# Altered DNA methylation indicates an oscillatory flow mediated epithelial-to-mesenchymal transition signature in ascending aorta of patients with bicuspid aortic valve

**DOI:** 10.1038/s41598-018-20642-4

**Published:** 2018-02-09

**Authors:** Hanna M. Björck, Lei Du, Silvia Pulignani, Valentina Paloschi, Karin Lundströmer, Alexandra S. Kostina, Cecilia Österholm, Anna Malashicheva, Anna Kostareva, Arturo Evangelista, Gisela Teixidó-Tura, Shohreh Maleki, Anders Franco-Cereceda, Per Eriksson, Mechanistic Interrogation of Bicuspid Aortic Valve associated Aortopathy (MIBAVA) Leducq Consortium, Harry C. Dietz, Bart Loeys, Lut Van Laer, Andrew S. McCallion, Luc Mertens, Seema Mital, Salah A. Mohamed, Gregor Andelfinger

**Affiliations:** 1 Cardiovascular Medicine Unit, Center for Molecular Medicine, Department of Medicine, Karolinska Institutet, Stockholm, Sweden; 2 Institute of Clinical Physiology, National Research Council, Pisa, Italy; 3 Almazov Federal Medical Research Centre, St.Petersburg, Russia; 4 ITMO University, Institute of Translational Medicine, St.Petersburg, Russia; 5 Saint-Petersburg State University, St.Petersburg, Russia; 6 Cell Therapy Institute, Nova Southeastern University, Fort Lauderdale, FL USA; 7 Cardiothoracic Surgery Unit, Department of Molecular Medicine and Surgery, Karolinska Institutet, Stockholm, Sweden; 8 Department of Cardiology, Hospital Universitary Vall d’Hebron, Barcelona and CIBERCV, Spain; 9 McKusick-Nathans Institute of Genetic Medicine, Johns Hopkins University School of Medicine, Baltimore, USA; 10 Howard Hughes Medical Institute, Baltimore, USA; 11 Department of Medical Genetics, University of Antwerp and Antwerp University Hospital, Antwerp, Belgium; 12 Cardiology, The Hospital for Sick Children, University of Toronto, Toronto, ON Canada; 13 Hospital for Sick Children, University of Toronto, Toronto, ON Canada; 14 Department of Cardio and Thoracic Vascular Surgery, Universitaetsklinikum Schleswig-Holstein, Campus Luebeck, Luebeck, Germany; 15 Sainte Justine University Hospital Research Center, Université de Montréal, Montréal, QC Canada

## Abstract

Disturbed flow has been suggested to contribute to aneurysm susceptibility in bicuspid aortic valve (BAV) patients. Lately, flow has emerged as an important modulator of DNA methylation. Hear we combined global methylation analysis with *in vitro* studies of flow-sensitive methylation to identify biological processes associated with BAV-aortopathy and the potential contribution of flow. Biopsies from non-dilated and dilated ascending aortas were collected from BAV (n = 21) and tricuspid aortic valve (TAV) patients (n = 23). DNA methylation and gene expression was measured in aortic intima-media tissue samples, and in EA.hy926 and primary aortic endothelial cells (ECs) isolated from BAV and TAV exposed to oscillatory (±12 dynes/cm^2^) or laminar (12 dynes/cm^2^) flow. We show methylation changes related to epithelial-mesenchymal-transition (EMT) in the non-dilated BAV aorta, associated with oscillatory flow related to endocytosis. The results indicate that the flow-response in BAV ECs involves hypomethylation and increased expression of WNT/β-catenin genes, as opposed to an angiogenic profile in TAV ECs. The EMT-signature was exasperated in dilated BAV aortas. Aberrant EMT in BAV aortic walls could contribute to increased aneurysm susceptibility, and may be due to disturbed flow-exposure. Perturbations during the spatiotemporally related embryonic development of ascending aorta and semilunar valves can however not be excluded.

## Introduction

Bicuspid aortic valve (BAV) is the most common congenital heart malformation, present in 1–2% of the population. Compared with individuals having a normal tricuspid aortic valve (TAV), BAV patients are at increased risk of ascending aortic dilatation^1^. The molecular mechanism underlying the increased susceptibility is not known, although we and others have shown that aneurysm formation in BAV and TAV patients is clearly distinct. The concurrence of a BAV and ascending aortic dilatation has been suggested to be the consequence of a genetic defect arising at early embryonic development^2^, or due to an increased hemodynamic burden with high oscillatory shear stress rendering the aortic wall dysfunctional^3^. Indeed, the importance of valve-related hemodynamics in BAV aortopathy has been addressed in a recent study in humans, showing a link between proteolytic dysregulation, elastic fiber degeneration and increased regional wall shear stress^4^.

As yet, there is no adequate BAV animal model targeting the molecular mechanisms behind the increased aneurysm susceptibility. First, with the exception of inbred Syrian hamsters, BAV animal models rarely develop BAV type I (fusion of the right and left coronary cusps), which is the most common type of BAV morphotype associated with a higher propensity for aneurysm formation in humans^5^. Second, most BAV animal studies so far exclusively focuses on the development of the bicuspid valve without addressing the state of the ascending aorta, which is a major concern in adult BAV patients. Lastly, the majority of BAV patients develop ascending aortic dilatation in adulthood, and molecular and histological analysis of BAV aorta implies that aneurysm formation in BAV is a gradual process with changes accumulating over a considerable period of time^6,7^. Thus, patient-based studies of adult BAV and TAV aorta still remain a valid alternative for unravelling molecular processes associated with BAV aortopathy.

DNA methylation is an important regulator of transcription and aberrant methylation have been described in numerous human diseases^8^. We have previously shown that DNMT1 and TET3, two key enzymes of the methylation machinery, are differentially expressed in the dilated aorta of BAV and TAV patients^7^, and that the protein expression of DNMT3A is changed already prior to dilatation^9^. Recently, several studies have demonstrated the importance of hemodynamics in the regulation of DNA methylation^10^, and we have previously shown that DNMTs and TETs are flow-responsive in the rat aortic arch^11^. Given the complex interaction between hemodynamics and genetic factors in the pathology of BAV aortopathy, DNA methylation may be of particular importance, mediating hemodynamic and genetic risks, thereby favoring aneurysm formation.

In the present study, we combined global DNA methylation analysis of ascending aortic biopsies from BAV and TAV patients with *in vitro* studies of flow-sensitive endothelial methylation to delineate biological processes associated with BAV aortopathy and the potential contribution of disturbed flow.

## Results

### The non-dilated BAV aorta shows a methylation signature associated with cell transformation and differentiation

To identify biological processes potentially contributing to the increased aneurysm susceptibility in BAV patients, we measured DNA methylation in the aortic intima-media portion of the ascending aorta of BAV and TAV patients operated due to valve disease, hence with normal ascending aortic dimensions. Due to the age difference between non-dilated BAV and TAV patients (Supplementary Table S1), methylation levels were corrected for age prior to analysis. To exclude cellular heterogeneity between BAV and TAV non-dilated intima-media samples, which may interfere with data interpretation, we analyzed the expression level of several cell type markers. As shown in Supplementary Table S3, there was no difference in the expression of VSMC- or EC-specific genes between BAV and TAV non-dilated aortas, nor was there any difference in inflammatory markers such as *VCAM1, TNF* and *NFKB*, or the macrophage markers *CD68* and *CD163*.

Then, analyzing the pattern of differential DNA methylation between BAV-ND and TAV-ND, we found no individual CpG that passed the strict correction for multiple testing. However, a total of 681 genomic clusters of differentially methylated CpGs, i.e. differentially methylated regions (DMRs) were identified, which were mapped to genes using GREAT (n = 894). In an attempt to capture relevant biological processes, we performed pathway and ontology analyses only on genes with a methylation fold change of ±10% (in total n = 540, of which n = 154 were hypermethylated in BAV and n = 398 were hypomethylated in BAV, Supplementary Table S4). Hallmark analysis showed that response to Estrogen was the most highly enriched differentially methylated genes (FDR q = 7.21e-8), followed by TNF signaling via NFKB (FDR q = 2.82e-7), hypoxia (FDR q = 1.37e-6), and epithelial mesenchymal transition (EMT) (FDR q = 3.48e-5) (Table 1). Estrogens^12^, NFKB-signaling^13^ and hypoxia^14^ are known stimulators of the EMT process. The differential methylation pattern was further evaluated by KEGG pathway analysis showing a similar molecular signature related to cell transformation (Table 1), including associations to Pathways in cancer (FDR q = 1.72e-6), WNT-signaling (FDR q = 3.54e-4), regulation of actin cytoskeleton (FDR q = 1.25e-3), endocytosis (FDR q = 1.33e-3), focal adhesion (FDR q = 2.35e-3), and calcium signaling (FDR q = 3.93e-3). Other ontology terms included insulin signaling (FDR q = 5.59e-6), hypertrophic cardiomyopathy (FDR q = 3.38E-5) and adipocytokine signaling (FDR q = 2.35e-3). Interestingly, GREAT analysis of differentially hypomethylated genes demonstrated an association with myosin light chain kinase (MYLK) activity (FDR q = 1.72e-2) (Supplementary Fig. S1). MYLK is one of the major RHOA kinases, involved in stress fiber formation and cytoskeleton rearrangements^15^ and we have previously shown higher expression of this protein in BAV-ND^9^.

**Table 1:** Hallmark and KEGG pathway analysis of BAV vs. TAV hypomethylated DMR-associated genes, non-dilated aorta. Age-corrected methylation values.

Gene Set Name		FDR q
**Hallmark Analysis**		
Estrogen response, late	Genes defining late response to estrogen.	7.21E-8
TNF signaling via NFKB	Genes regulated by NF-kB in response to TNF.	2.82E-7
Hypoxia	Genes up-regulated in response to low oxygen levels.	1.37E-6
Estrogen response, early	Genes defining early response to estrogen.	6.95E-6
Epithelial mesenchymal transition (EMT)	Genes defining EMT as in wound healing, fibrosis and metastasis.	3.48E-5
TGFβ signaling	Genes up-regulated in response to TGFB1.	2.77E-4
Apoptosis	Genes mediating programmed cell death by activation of caspases.	6.54E-4
IL2/STAT5 signaling	Genes up-regulated by STAT5 in response to IL2 stimulation.	6.54E-4
Peroxisome	Genes encoding components of peroxisome.	1.05E-3
Reactive oxygen species pathway	Genes up-regulated by reactive oxygen species.	1.15E-3
**KEGG pathway Analysis**		
Pathways in cancer	—	1.72E-6
Insulin signaling pathway	—	5.59E-6
Hypertrophic cardiomyopathy	—	3.38E-5
Chronic myeloid leukemia	—	8.36E-5
WNT signaling pathway	—	3.54E-4
Regulation of actin cytoskeleton	—	1.25E-3
Endocytosis	—	1.33E-3
Focal adhesion	—	2.35E-3
Adipocytokine signaling pathway		2.35E-3
Calcium signaling pathway	—	3.93E-3

### Methylation changes in the non-dilated BAV aorta are associated with an oscillatory flow profile, specifically related to endocytosis and WNT/β-catenin signaling

Disturbed hemodynamics, characterized by high shear stress and flow reversal has been described in the BAV ascending aorta and suggested to aggravate aneurysm development in BAV individuals^3^. Indeed, perturbed flow renders the endothelium dysfunctional, thereby influencing underlying smooth muscle cell phenotype and function. In order to test whether disturbed hemodynamics may be a contributing factor to the BAV-related methylation signature by promoting methylation changes in the endothelium, we exposed EA.hy926 cells to uniform (12 dynes/cm^2^ laminar) and bidirectional (±12 dynes/cm^2^ oscillatory) flow for 48 hours and identified flow-sensitive DNA methylation associated with laminar and oscillatory flow, respectively. We then investigated the overlap between genes differentially methylated between BAV-ND and TAV-ND (n = 894) and genes changing their methylation pattern in response to the two different types of flow, based on DMR analysis. This showed that the differential methylation profile observed in the non-dilated BAV aorta was significantly associated with an oscillatory flow methylation profile (P = 0.01). Specifically, 23% (n = 207, Supplementary Table S5) of differentially methylated genes in non-dilated aorta overlapped the oscillatory flow profile, as opposed to 18% (n = 163) overlapping the laminar flow profile. A full list of oscillatory flow-responsive hypo- (n = 1644) and hypermethylated (n = 169) DMRs and associated genes can be found in Supplementary Table S6. Further, to delineate which biological processes that may be related to oscillatory flow in BAV-ND, KEGG pathway analysis of the 207 overlapping genes was performed, showing enrichment for Endocytosis (FDR q = 1.48e-3) and regulation of actin cytoskeleton (FDR q = 2.84e-3). Endocytic activity has been reported to play an important role in the initiation of cellular programs involved in the onset and progression of EMT^16^, and we have previously shown that endocytosis is activated in the non-dilated BAV aorta^9^. Type I diabetes was the top enriched pathway (FDR q = 2.49e-4), and when looking at specific genes covered by the ontology term (i.e. *HLA-B*, *HLA-DMB, HLA-DPA1, GAD1* and *CPE*) we found that *HLA* genes were down-regulated in BAV-ND at mRNA level (*HLA-B*, P = 0.011; *HLA-DMB*, P = 0.033; *HLA-DPA1*, P = 0.010), which may be indicative of a decrease in immune reactivity. Of note, the associations remained after excluding DMR-genes altered also in the internal thoracic artery of the same patients (data not shown), supporting that the changes may be flow-related and aorta-specific. We further noted that several key transcription factors for EMT, such as ZEB1, SNAI2 and TWIST1 changed their methylation pattern in EA.hy926 cells in response to oscillatory flow, becoming hypomethylated (Supplementary Table S6). Moreover, to further verify the profile associated with perturbed flow, we analyzed global gene expression in EA.hy926 exposed to oscillatory flow and performed Hallmark analysis on differentially expressed genes. Indeed, this confirmed that EMT was the most highly enriched hallmark of genes changing their expression in response to oscillatory flow (FDR q = 2.35e-16). Other enriched hallmarks included e.g. TNF-signaling via NFKB, apoptosis and mTORC1-signaling (Supplementary Fig. 2). In total, 1321 genes were differentially expressed (FDR 0.01), 65% (n = 867) of which were upregulated in response to oscillatory flow.

To gain further insight into the possible role of disturbed flow to BAV aortopathy, we exposed primary aortic endothelial cells isolated from BAV and TAV aneurysmal patients to oscillatory flow for 6 hours and measured changes in DNA methylation in comparison to static conditions. Interestingly, substantially fewer genes changed their methylation pattern in response to flow in BAV cells compared to TAV cells, suggesting a defective flow-response of the BAV aneurysmal endothelium. Specifically, n = 4707 flow-sensitive DMR-genes were identified in BAV vs. n = 5999 flow-sensitive DMR-genes in TAV. In order to investigate the flow-associated phenotype, we analyzed expression levels of all identified DMR-genes in the same samples. In total, n = 400 genes in BAV and n = 1251 genes in TAV changed their expression in response to bi-directional oscillatory flow (FDR10%) (Supplementary Table S7). In BAV cells, 83% (n = 333) of the genes were up-regulated in oscillatory flow conditions, compared to only 10% (n = 135) up-regulated genes in TAV ECs, indicating that BAV and TAV ECs respond to perturbed flow in opposite directions. Further, KEGG pathway analysis of up-regulated genes showed that endocytosis (FDR q = 1.44e-5), adherence junctions (FDR q = 1.85e-5) and TGF-beta signaling (FDR q = 3.94e-5) were enriched in BAV ECs, compared to focal adhesion (FDR q = 8.42e-4), Leishmania infection (FDR q = 1.67e-3), among others, in TAV ECs (Supplementary Table S8). Shared ontology terms between BAV and TAV included pathways in cancer and MAPK signaling. However, when looking at specific genes covered by the GO terms, we found that the cancer-related phenotype was clearly distinct between BAV and TAV (Supplementary Table S9). Interestingly, in BAV ECs, several genes involved in WNT/β-catenin and KRAS signaling were up-regulated in response to oscillatory flow, suggesting that these pathways may be of more importance for the development of BAV-related aneurysm. In flowed TAV ECs, on the other hand, the cancer-related profile was primarily associated with angiogenesis and inflammation. MAPK signaling was more similar between BAV and TAV (Supplementary Table S9). BAV-specific genes included e.g. ATF4, RAP1B, PPP3CC and SRF.

Collectively, our results suggest that oscillatory flow may be one of the factors contributing to changes in DNA methylation associated with EMT-related mechanisms in BAV, thereby adding to the susceptibility of aortopathy in BAV individuals.

### DNA methylation is inversely related to gene expression

**Figure 1:**
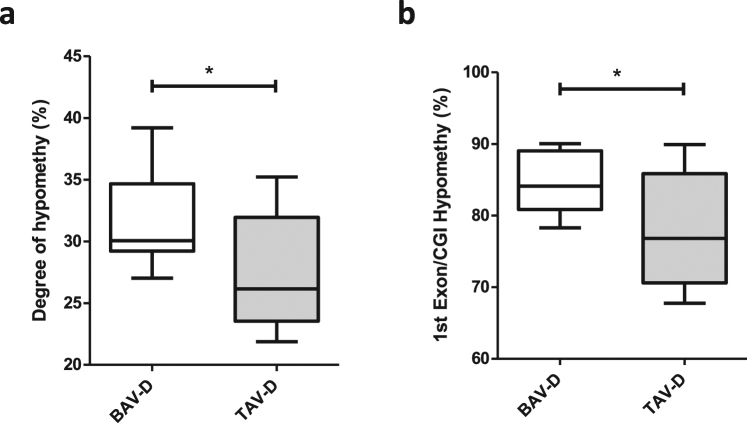
Hypomethylation in BAV and TAV dilated (D) aortas. Degree of (**a**) hypomethylation and (**b**) hypomethylation of CpG-island within the 1^st^ exon. N = 14 BAV and N = 13 TAV (t-test). Data is expressed as (%) ± standard deviation. *P ≤ 0.05.

**Figure 2:**
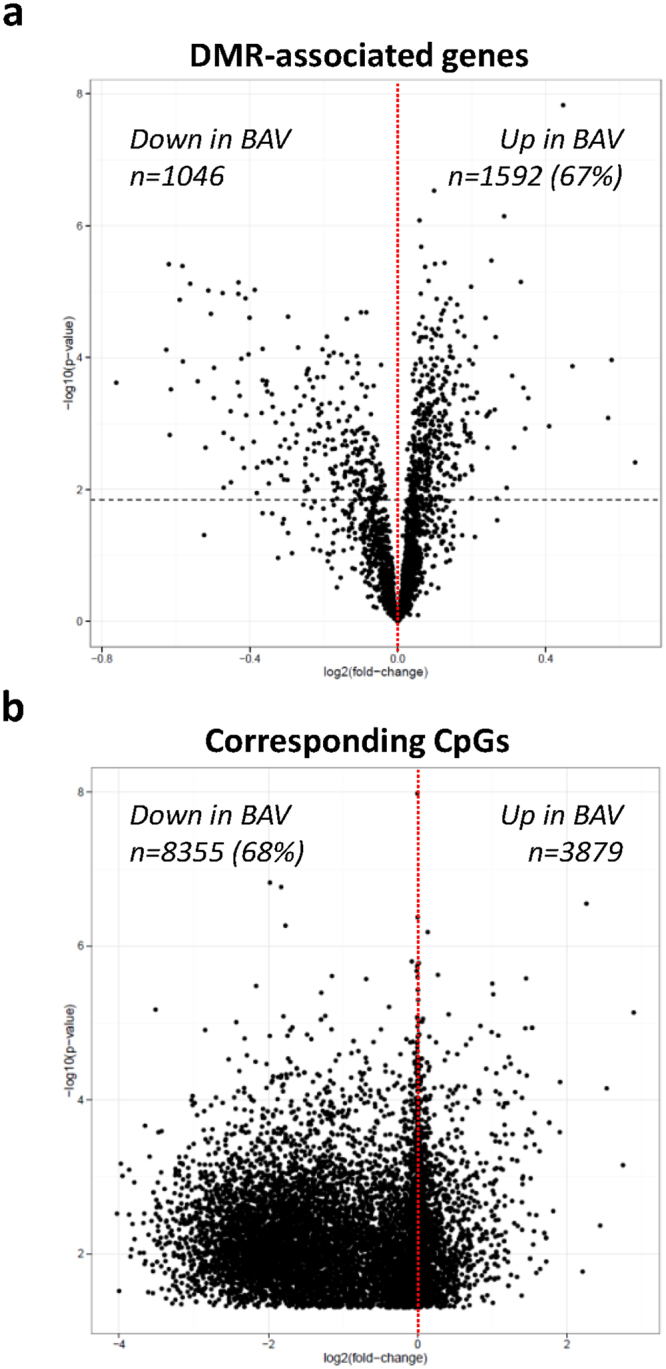
Relationship between gene expression and DNA methylation, BAV and TAV dilated aortas. Volcano plots of −log10 (P-value) against log2 (fold-change) of (**a**) gene expression and (**b**) DNA methylation beta values, of DMR-associated genes and their corresponding CpGs. Dashed horizontal line represents the Bonferroni-adjusted threshold for statistical significance (t-test). N = 14 BAV and N = 13 TAV.

**Figure 3:**
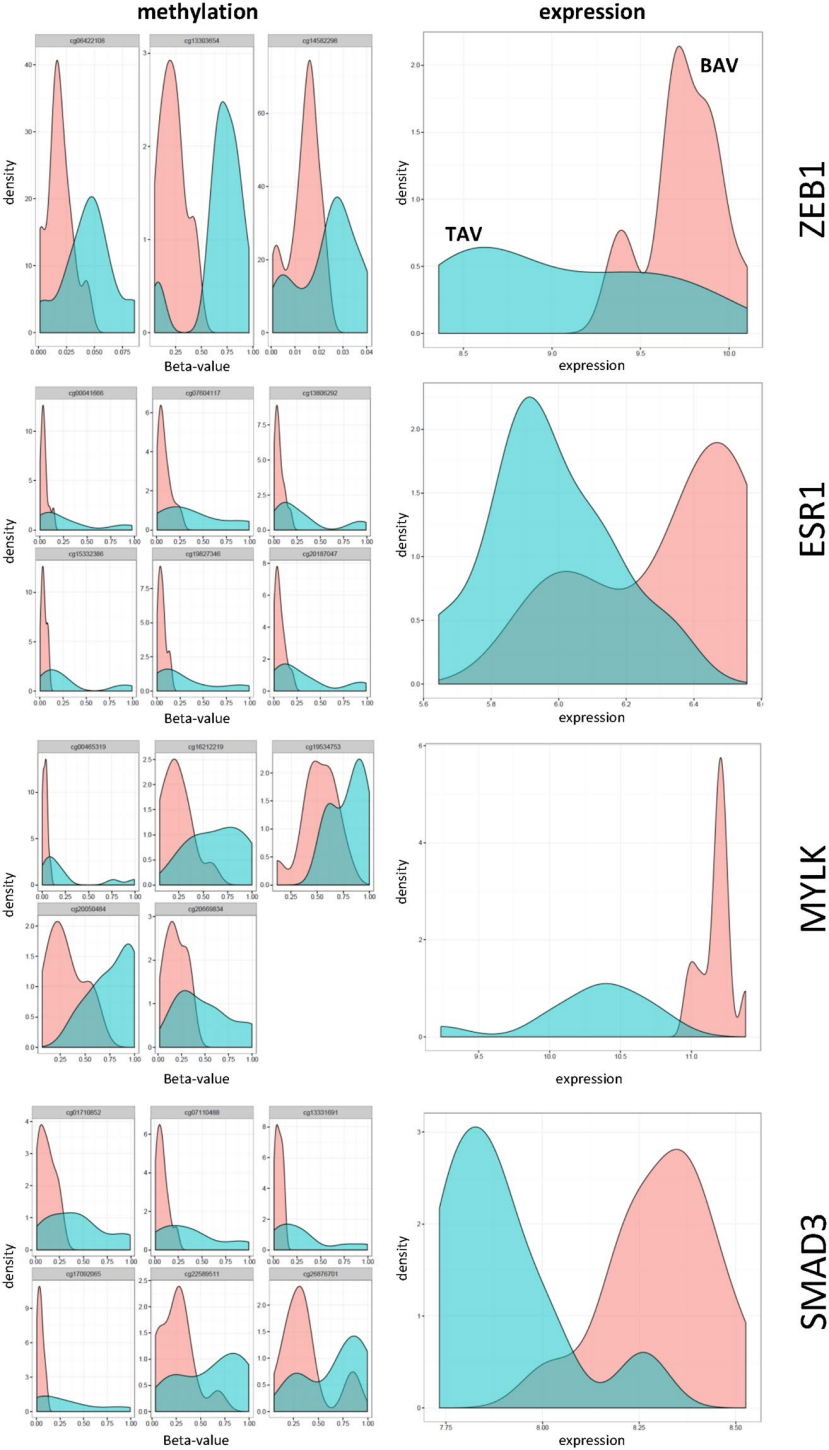
DNA methylation and gene expression density distribution plots of EMT related genes, BAV and TAV dilated aortas. DNA methylation and gene expression levels are measured in the same ascending aortic biopsy. N = 12 BAV (pink) and N = 12 TAV (blue). Methylation data is presented as beta values.

DNA methylation plays an important role in regulation of gene expression. High levels of gene expression often associate with low levels of DNA methylation (hypomethylation) within the promoter region of a gene. To investigate the importance of differential methylation in relation to gene expression we measured gene expression and DNA methylation in the same aortic biopsy. Of note, due to the small size of non-dilated aortic biopsies, this analysis was performed only on dilated aortic samples. First, the degree of hypomethylated CpGs (here defined as CpGs with <5% methylation) was assessed in BAV and TAV dilated aortas. This showed that the degree of hypomethylation was significantly higher in BAV-D than in TAV-D aorta (P = 0.026) (Fig. 1a), which was also true for CpGs located inside CpG-islands within first exons, i.e. regions tightly linked to transcriptional control^17^ (P = 0.018) (Fig. 1b). This suggested a more transcriptionally active profile in BAV-D, which was confirmed by analyzing the differential mRNA expression (DE) between BAV-D and TAV-D (in total 2514 DE genes, FDR 10%; n = 1561 up-regulated in BAV-D, n = 953 up-regulated in TAV-D). To elucidate this further, we identified DMRs between BAV-D and TAV-D, based on the same criteria as for the non-dilated aorta, and selected all DMRs overlapping RefSeq genes (n = 3980 DMRs, covering 3207 genes). DMRs covering non-expressed genes, based on the expression level of Y-chromosome genes in women, were filtered out resulting in 3170 DMRs, harboring 12 234 CpGs and covering 2638 genes. Of these CpGs, 68% (n = 8355) were less methylated in BAV-D, and accordingly, 67% (n = 1592) of the DMR-associated genes had a higher expression in BAV-D. This further indicated an inverse relationship between methylation and gene expression in BAV-D (Fig. 2). Detailed methylation and gene expression plots for selected EMT-related genes are shown in Fig. 3. Additional gene/methylation plots are presented in Supplementary Fig. S3.

### The EMT-signature is more pronounced in the dilated BAV aorta

**Figure 4:**
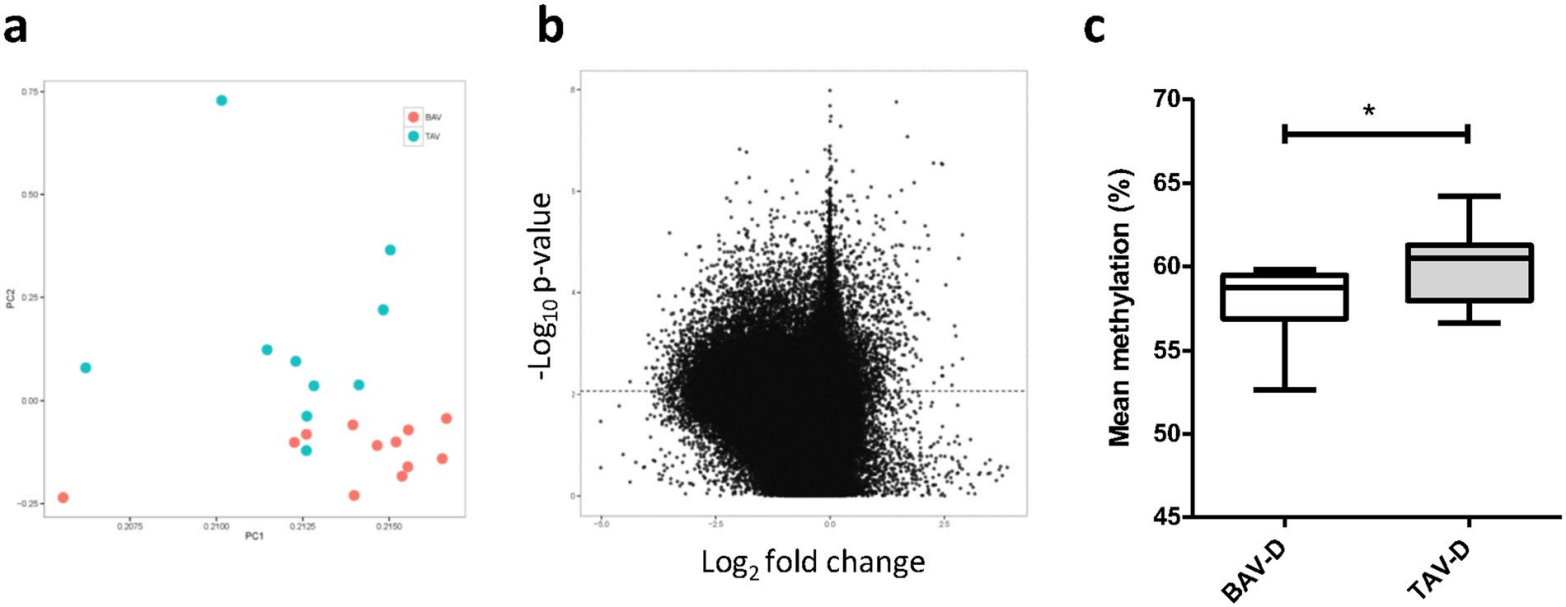
Differential DNA methylation, BAV and TAV dilated (D) ascending aortas. (**a**) Principle component analysis of BAV (pink) and TAV (blue) DNA methylation using M-values; (**b**) Volcano plot of −log10 (P-value) against log2 (fold-change) of beta values, representing differential methylation between BAV and TAV (t-test). Dashed line represents the Bonferroni-adjusted threshold for statistical significance; (**c**) mean DNA methylation, presented as (%) ± standard deviation (t-test). *P ≤ 0.05. N = 14 BAV and N = 13 TAV.

The pattern of global DNA methylation was further analyzed in dilated aortic intima-media samples of BAV and TAV patients. As there was no difference in age between the two groups (Supplementary Table S1), raw methylation values were used. Principal component analysis (PCA) indicated that characteristics of aortic dilatation in BAV and TAV patients was clearly distinct (Fig. 4a). Specifically, 39 984 individual CpGs were differentially methylated between BAV-D and TAV-D after correction for multiple testing (FDR 10%, P ≤ 0.0085), the majority of which were more methylated in TAV-D (Fig. 4b). This is in agreement with the lower level of mean methylation observed in TAV-D aorta (P = 0.031) (Fig. 4c). Importantly, bisulfite pyrosequencing of selected individual CpGs confirmed the Illumina 450 k methylation data (Supplementary Fig. S4). Moreover, the observed methylation differences were specific for the aorta as no difference in global methylation or mean methylation was observed in the intima-media of internal thoracic arteries of the same patients (Supplementary Fig. S5).

Furthermore, a total of 4913 DMRs were identified between BAV-D and TAV-D, the functional relevance of which was investigated using GREAT and GSEA. The analyses were restricted to DMRs with a fold change of ±10% (n = 3147 DMRs; n = 2673 hypomethylated in BAV and n = 474 hypermethylated in BAV). Hypomethylated BAV-D DMRs were associated with 3052 genes (Supplementary Table S10), and Hallmark analysis revealed that these genes were related to EMT (FDR q = 2.91e-29), smooth muscle myogenesis (FDR q = 2.97e-25) and hypoxia (FDR q = 1.35e-20) (Table 2). This was further confirmed using GREAT showing association with biological processes related to actin filament bundles (P = 7.09e-12), stress fibers (P = 1.72e-11), focal adhesion (P = 1.06e-8) and adherence junctions (P = 2.97e-8) (Supplementary Fig. S6). KEGG pathway analysis showed strong association with pathways in cancer (FDR q = 1.78e-22), MAPK-signaling (FDR q = 8.66e-19), focal adhesion (FDR q = 6.8e-17), WNT-signaling (FDR q = 3.56e-14), and regulation of actin cytoskeleton (FDR q = 2.26e-12) (Table 2). Interestingly, hypomethylated genes were also associated with negative regulation of the TGFβ-signaling (P = 6.84e-12) (Supplementary Fig. S6), which is in line with previous reports by our group^18^. Hallmark and KEGG pathway analysis of hypermethylated DMR-genes (n = 581, Supplementary Table S10) further strengthened the BAV-D EMT signature (Table 2).

**Table 2:** Hallmark and KEGG pathway analysis of BAV hypo- and hypermethylated DMR-associated genes, dilated aorta.

Gene Set Name	Description	FDR q	Gene Set Name	FDR q
HALLMARK			KEGG PATHWAY	
**Hypomethylated DMR-genes**				
EPITHELIAL MESENCHYMAL TRANSITION (EMT)	Genes defining EMT, as in wound healing, fibrosis and metastasis.	3.10E-29	PATHWAYS IN CANCER	1.78E-22
MYOGENESIS	Genes involved in development of skeletal muscle.	3.14E-25	MAPK-SIGNALING	8.66E-19
HYPOXIA	Genes up-regulated in response to low oxygen levels	1.42E-20	FOCAL ADHESION	6.80E-17
ESTROGEN RESPONSE-EARLY	Genes defining early response to estrogen.	1.99E-18	WNT-SIGNALING	3.56E-14
KRAS-SIGNALING	Genes up-regulated by KRAS activation.	8.66E-18	REGULATION ACTIN CYTOSKELETON	2.26E-12
ADIPOGENESIS	Genes up-regulated during adipocyte differentiation	1.97E-14	DILATED CARDIOCARDITIS	5.88E-12
MITOTIC SPINDLE	Genes important for mitotic spindle assembly.	1.97E-14	VIRAL MYOCARDITIS	2.15E-10
APOPTOSIS	Genes mediating programmed cell death by activation of caspases.	1.37E-12	CALCIUM-SIGNALING	6.28E-10
APICAL JUNCTION	Genes encoding components of apical junction complex.	1.40E-12	HYPERTROPHIC CARDIOMYOPATHY	9.71E-10
KRAS-SIGNALING	Genes up-regulated by KRAS activation.	4.08E-11	PATHWAYS IN CANCER	3.04E-08
ALLOGRAFT REJECTION	Genes up-regulated during transplant rejection.	1.37E-08	TYPE I DIABETES MELLITUS	9.38E-05
TNFA-SIGNALING VIA NFKB	Genes regulated by NF-kB in response to TNF.	5.10E-07	JAK_STAT-SIGNALING	2.06E-04
EPITHELIAL MESENCHYMAL TRANSITION	Genes defining EMT, as in wound healing, fibrosis and metastasis.	1.72E-06	CHEMOKINE SIGNALING	2.06E-04
ESTROGEN RESPONSE-LATE	Genes defining late response to estrogen.	1.72E-06	CYTOKINE**-**CYTOKINE REC INTERACTION	2.35E-04
INTERFERON γ RESPONSE	Genes up-regulated in response to IFNG.	1.72E-06	WNT-SIGNALING	5.67E-04
ESTROGEN RESPONSE-EARLY	Genes defining early response to estrogen.	8.07E-06	INSULIN-SIGNALING	1.35E-03
HYPOXIA	Genes up-regulated in response to low oxygen levels.	8.07E-06	ACUTE MYELOID LEUKEMIA	1.96E-03
STAT5-SIGNALING	Genes up-regulated by STAT5 in response to IL2 stimulation.	4.15E-05	SNARE INTERACTIONS IN VESICULAR TRANSPORT	1.96E-03
NOTCH-SIGNALING	Genes up-regulated by Notch signaling.	2.04E-04		

To further investigate the EMT-like state associated with BAV-D we specifically looked at differential methylation of key EMT transcription factors. Interestingly, ZEB1, SNAI2 and TWIST2 were hypomethylated in BAV-D compared to TAV-D (Supplementary Table S11). ZEB1 is one of the key transcription factors of EMT/EndMT^19^, and as shown in Fig. 3, there was an inverse relationship between *ZEB1* gene expression and DNA methylation, i.e. *ZEB1* mRNA was up-regulated in BAV-D compared to TAV-D aorta (P = 0.001)^9^. DMRs associated with TWIST2 were located in the 5′UTR, 1^st^ exon and TSS, that is regions linked to transcriptional control, as well as within the gene body. The DMR associated with SNAI2 was located in the TSS. Collectively this data suggests a more pronounced EMT-state in BAV dilated aortas.

To address the possible effect of oscillatory flow on the differential methylation pattern in BAV-D, we performed KEGG pathway analysis on overlapping DMR-genes that were differentially methylated in BAV aortic ECs exposed to disturbed flow for 6 hours and in BAV-D aorta (n = 1268), with exclusion of DMR-genes being differentially methylated also in the internal thoracic artery of the same patients. Interestingly, shared genes were enriched for cancer-related pathways (FDR q = 2.02e-13) and several other EMT-related processes, including regulation of actin cytoskeleton, focal adhesion and MAPK- WNT- and TGFβ-signaling (Supplementary Table S12). Genes common between the internal thoracic artery and the aorta that were not altered by oscillatory flow, (n = 191) were related to hypoxia (FDR q = 8.73e-3).

### Core cardiac transcription factors are hypermethylated in the BAV aorta

**Figure 5:**
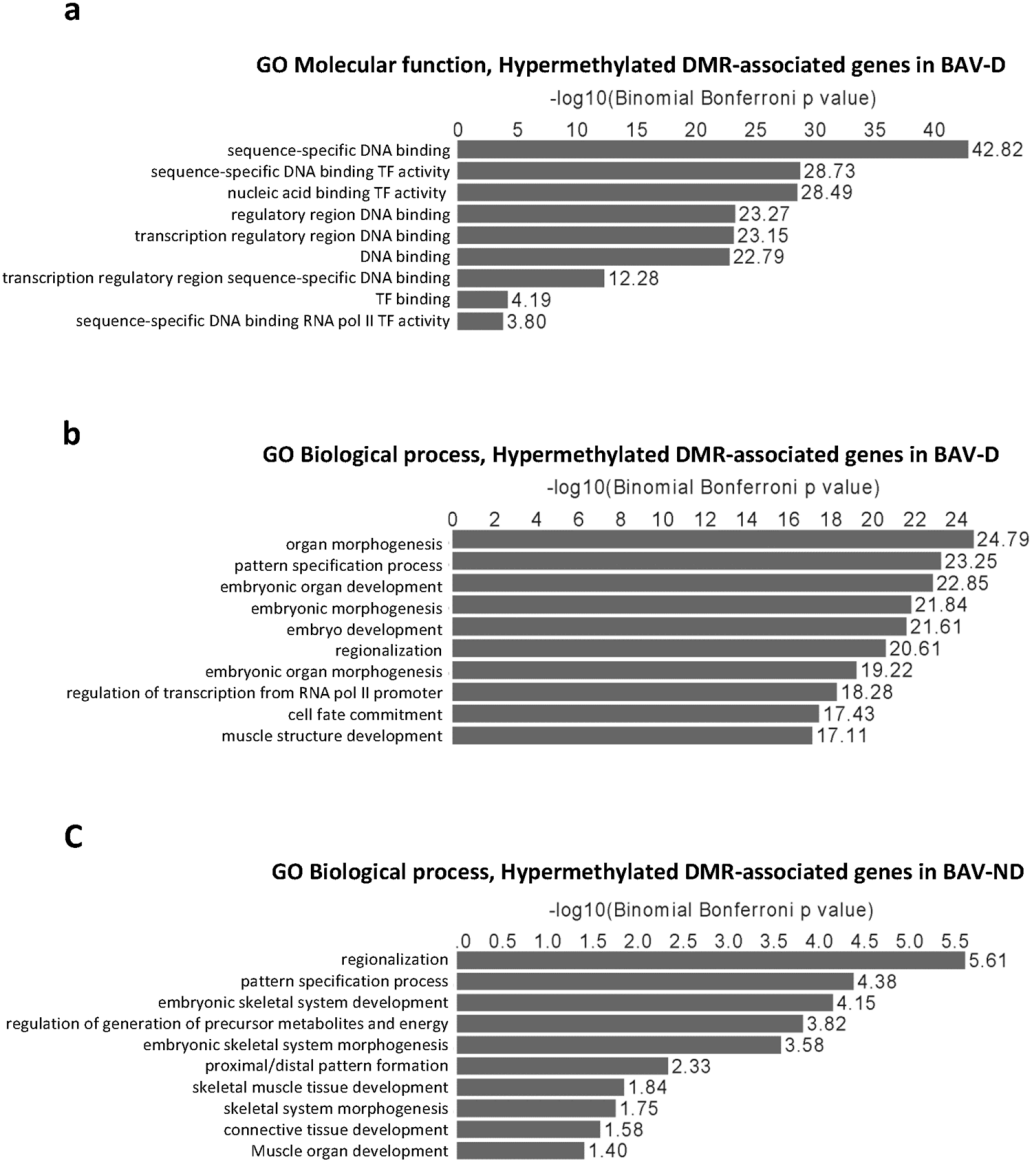
Gene ontology analysis of BAV hypermethylated DMR-associated genes. (**a**) Hypermethylated DMR-associated genes in BAV dilated (D) aorta, Molecular function; (**b**) Hypermethylated DMR- associated genes in BAV-D aorta, Biological processes; (**c**) Hypermethylated DMR- associated genes in BAV non-dilated (ND) aorta, Biological processes. N = 14 BAV-D; N = 13 TAV-D; N = 7 BAV-ND; N = 10 TAV-ND. −Log10 Binominal Bonferroni P-values are presented.

Lastly, GREAT analysis revealed that hypermethylated genes in BAV-D were strongly associated with sequence-specific DNA binding (P = 1.51e-43) and TF activity (P = 9.28e-29) (Fig. 5a), and among the 153 genes belonging to significant MF terms, numerous Homeobox genes (*HoxA13, HoxB1, HoxB4*, *HoxB5, HoxC4, HoxC5, HoxC6, HoxC8, HoxC9, HoxC11, HoxC12* and *HoxD8*) and SIX Homeobox genes (*Six2* and *Six3*) were observed. *Hox* genes play important roles during cardiac neural crest patterning by modulating EMT/EndMT^20^. Accordingly, hypermethylated DMR-genes were highly associated with organ morphogenesis (P = 1.63e-25), pattern specification (P = 5.56e-24) and cell fate commitment (FDR q = 3.67e-18) (Fig. 5b). Other significant BP-terms included Heart and mesenchyme development, outflow tract morphogenesis, and cell migration during heart development. In addition, several core cardiac TFs controlling heart development and vascular homeostasis (e.g. *GATA4, Nkx2-5, Nkx2-6, Tbx2, Tbx3, Tbx5* and *Isl1)* were hypermethylated in BAV-D (supplementary Table S10). A similar pattern of hypermethylation was observed in BAV non-dilated aortas (Fig. 5c).

## Discussion

Bicuspid aortic valves predispose to ascending aortic dilatation with increasing age. The underlying molecular mechanism is not known, however it has been shown that aortic dilatation in BAV-patients is clearly distinct from aortic dilatation in patients with a normal TAV^7^. A genetic defect and/or valve-related flow disturbances have been discussed as contributing factors. In the present study, we undertook an unbiased global DNA methylation approach of BAV and TAV aortic tissue, combined with *in vitro* analysis of flow-sensitive endothelial methylation using EA.hy926 and primary aortic ECs from BAV and TAV patients, to discover novel biological processes specific for BAV-associated aortopathy and dissect potential flow-related changes. We show that the non-dilated BAV aortic intima-media was associated with methylation changes related to EMT-like biological processes, such as pathways in cancer, regulation of actin cytoskeleton and endocytosis, and that the observed differential methylation signature was associated with an oscillatory flow profile specifically related to endocytosis. Our results further indicate that the response to perturbed flow in BAV cells involves hypomethylation, combined with an increased expression of WNT/β-catenin genes, as opposed to an angiogenic profile observed in TAV ECs. In addition, primary BAV aneurysmal ECs show an attenuated response to flow in comparison to TAV cells. Moreover, the EMT signature was more pronounced in the BAV-D aorta, associated with more structurally related processes, including stress fiber formation and junctional integrity. In accordance with our previous observations^9^, we propose that the observed differential methylation signature signifies aberrant EMT in the adult BAV aortic intima-media, and that this predispose BAV individuals to aortic complications. Moreover, perturbed flow may be contributing to changes in DNA methylation towards promotion of a mesenchymal phenotype. However, perturbations of embryonic development causing aberrant EMT in the adult BAV aorta remain a possibility.

We have previously shown a molecular signature associated with endothelial junction instability and activation of the RHOA pathway in the intima-media in the BAV ascending aorta prior to dilatation^9^. Here, DNA methylation profiling of BAV and TAV non-dilated aortas further strengthened our previous observations, specifically showing a BAV-ND methylation signature related to Pathways in cancer, WNT/β-catenin signaling, regulation of actin cytoskeleton, endocytosis and focal adhesion, all of which are biological processes related to EMT. EMT, or endothelial-mesenchymal transition (EndMT), is initiated during specific stages of embryonic development, including aortic valve leaflet formation and septation of the outflow tract into the ascending aorta and the pulmonary trunk. EMT/EndMT can also be re-activated by transcriptional reprogramming in adult life under certain pathological conditions, such as cancer, wound healing and fibrotic disease^21^. Recent studies have demonstrated that changes in the epigenetic signature play an important role during acquisition of a mesenchymal phenotype in cancer^21^, as well as during phenotypic switch of SMC^22,23^. Induction of EMT/EndMT in aortic SMCs results in reorganization of actin filaments and disassembly of cell-cell contact, favoring the mesenchymal VSMC phenotype and vascular remodeling. To our knowledge, the specific molecular phenotype of BAV ascending aortic SMCs has not been fully explored, however, a maturation defect of the non-dilated BAV aortic wall characterized by de-differentiated SMCs and intimal thinning has been suggested^24^. In line with this, we have previously shown an increased SMC proliferation in the non-dilated BAV aorta compared with TAV-ND, noted by an increased expression of Ki67^9^. Likewise, ZEB1 was increased at protein level in the non-dilated intima-medial of BAV patients, which agrees well with the hypomethylation of ZEB1 reported here. In line with this, disturbed EndMT has previously been demonstrated in BAV patients with aortic aneurysm^25^.

When analyzing the differential methylation signature in the non-dilated aorta we found that differentially methylated genes were related to insulin- and adipokine signaling, and hypertrophic cardiomyopathy, which may seem surprising. However, specific genes belonging to the ontology terms included several AMP-activated protein kinases (AMPKs), such as PRKAG2, PRKAG3 and PRKAB1, as well as calcium channel genes (CACNA2D1 and CACNB2) and genes related to cytoskeletal reorganization (SH2B2^26^, SOCS1^27^, RAPTOR^28^, ITGA6^29^ etc.). AMPKs have previously been shown to play an important role in regulation of cell polarity via remodeling of the actin cytoskeleton^30^. Additionally, the association with insulin signaling and diabetes may signify a link to endothelial dysfunction, which has previously been described in BAV patients^31,32^.

The pattern and degree of DNA methylation can be modified by environmental factors, and recently, perturbed flow has emerged as an important regulator of DNA methylation^10^. Interestingly, impaired flow has been discussed as a causal factor for BAV aortopathy, affecting endothelial control of underlying SMCs phenotype and function^33^. Indeed, the importance of valve-related hemodynamics in BAV aortic disease has been addressed in humans showing a link between ECM proteolytic dysregulation, elastic fiber degeneration and increased regional wall shear stress^4^. Also, EndMT has previously been shown to contribute to vascular disease by inducing endothelial differentiation in a shear dependent manner^34^. Therefore, to investigate the potential contribution of different types of flow to the differential methylation pattern observed between the BAV and TAV aortic intima-media, we exposed endothelial cells (EA.hy926) to unidirectional laminar flow and bi-directional oscillatory flow for 48 h. Interestingly, the non-dilated EMT methylation pattern was significantly associated with an oscillatory flow profile, and several key transcription factors for EMT/EndMT were hypomethylated in response to oscillatory flow. Further, the oscillatory flow related methylation pattern in BAV was specifically related to endocytosis. Endocytosis has emerged as an important circuitry in the onset and progression of EMT/EndMT, enabling membrane trafficking, receptor recycling and degradation, ultimately promoting mesenchymal features, such as changes in cell polarity, cell-cell contact and cell migration^16^. Endocytic activity in endothelial cells has previously been shown to be regulated by shear stress^35,36^, and we have recently demonstrated an increased endocytic activity in the BAV-ND endothelium associated with an increased accumulation of degrading material^9^. These results are in line with previous studies investigating the effect of oscillatory shear stress on EMT/EndMT using different experimental setups, including flow systems and type of endothelial cells^37–39^.

The flow-related mesenchymal phenotype was further dissected using primary endothelial cells isolated from BAV and TAV dilated aortas, in which matched differential methylation and gene expression was measured under perturbed flow conditions. Of note, due to the source of primary endothelial cells, i.e. patient aneurysmal aorta, cells could unfortunately not resist exposure to high oscillatory shear stress for more than 6 hours. This showed that the flow-related association with endocytic activity was specific for BAV ECs, and that BAV cells up-regulated cancer/EMT-related genes involved in WNT/β-catenin signaling. TAV cells, on the other hand, demonstrated a more angiogenic and inflammatory cancer/EMT-like phenotype in response to flow. These BAV and TAV differential phenotypes are in line with previous studies showing involvement of WNT in BAV aneurysm formation^40^, as well as a lower expression of VEGF-A in BAV aneurysms compared to TAV^9,41^. Also, we have previously shown that aneurysm formation in TAV, but not BAV, is accompanied by increased levels of collagen and fibrosis^42,43^. In addition, although both BAV and TAV cells were enriched for genes related to MAPK-signaling, certain differences between them was observed. For example, several genes related to endothelial integrity and cell motility, such as ATF4 and SRF, were up-regulated in BAV but not in TAV. SRF is a known regulator of numerous genes controlling EMT processes^44^, and the binding of SRF to MYOCD, and hence regulation of SMC-specific genes, has been demonstrated to be epigenetically controlled^45^. ATF4 has previously been shown to induce EMT in neural crest cells^46^.

Collectively, these results suggest that aneurysm initiation and formation in BAV patients involves endocytic activity and WNT/β-catenin signaling, and that perturbed flow, at least in part, may contribute to this phenotype. The importance of WNT/β-catenin signaling in BAV aneurysms, however, needs to be further elucidated.

The EMT/EndMT methylation signature was more pronounced in the dilated BAV aorta, as indicated by the association with additional pathways involved in structural changes required for EMT/EndMT, as well as a stronger statistical significance. Thus, we speculate that EMT/EndMT-related changes commenced in the non-dilated BAV aorta during embryonic development or in post-natal life could accumulate over time, eventually contributing to aortic dilatation. Moreover, the dilated BAV aorta was hypomethylated, a state which has been associated with various human diseases^8,47^ Oscillatory flow exposure resulted in a drastic loss of methylation (hypomethylation) in both EA.hy926 cells and primary aortic ECs, raising the possibility that life-long exposure to perturbed flow may contribute to, and exasperate, alterations in DNA methylation, thereby contributing to BAV aneurysm development. Accordingly, loss of methylation in BAV ECs exposed to oscillatory flow was associated with an up-regulation of gene expression. Further, compared to TAV ECs, BAV ECs showed an attenuated response to oscillatory flow, suggesting that the response to shear may be compromised in diseased BAV cells, possibly due to continuous and long-term exposure to perturbed flow.

Alternatively, the EMT/EndMT-like signature observed in the BAV aorta may be due to inherent properties resulting from defective crosstalk between cardiac progenitors at a stage critical for the inter-related embryogenesis of aortic valves and the ascending aorta^48^. Indeed, genes implicated in BAV disease have recently been shown to be more frequently differentially methylated in BAV patients compared to TAVs^49^. Here we show that differentially methylated genes common between BAV-D ascending aortas and internal thoracic arteries that were not responsive to flow were related to hypoxia. Elevated oxidative stress has previously been demonstrated in the non-aneurysmal BAV aorta^50^. Another possibility is that abnormal hemodynamic interacts with intrinsic/genetic factors, as in many human complex diseases, resulting in abnormal EMT/EndMT and disease development.

Noteworthy is that BAV-D hypomethylated DMRs were associated with genes such as *PRDM16*, *SKI* and *PMEPAI1*, which are involved in negative regulation of TGFβ-signaling. This is in line with the increased sequestration of TGFβ in the extracellular space of BAV-patients, shown previously^18^. Accordingly, the mRNA expression of *PRDM16* and *PMEPAI1* was higher in BAV-D (P = 0.05 for both) compared with TAV-D. This collectively suggests that other signaling cascades, such as MAPK and/or WNT, rather than canonical TGFβ, may be more relevant for aneurysm development in BAV.

Lastly, several core transcription factors important for proper cardiac development were hypermethylated in non-dilated and dilated BAV ascending aortas. Epigenetic mechanisms are known to play important roles during cardiac morphogenesis, and *de novo* mutations in genes regulating histone methylation have been implicated in congenital heart defects^51^. Importantly, several putative disease-causing variants in various cardiac transcription factors have been identified in BAV patients^40^ suggesting a polygenic etiology of BAV aortopathy. However, as disruption of instructive signals between cardiac and vascular progenitors during embryonic development of the outflow tract has been postulated to be the link between BAV formation and concomitant susceptibility to ascending aortic disease^48^, the observed differential methylation of cardiac transcription factors may also relate to this disruption, manifested by aortic disease in adult BAV patients.

In summary, *in vivo* analysis of BAV and TAV aortic intima-media DNA methylation, combined with identification of flow-sensitive BAV endothelial DNA methylation by the use of an *in vitro* flow system, identified an EMT/EndMT-like methylation signature in the non-dilated BAV aorta, which was even more pronounced in the dilated state, and suggested that disturbed flow may be a contributing factor to the EMT/EndMT phenotype. Defective EMT/EndMT during embryonic development, or an interplay between intrinsic genetic and flow factors, can however not be ruled out. Aberrant EMT/EndMT of the ascending aorta may be a cause for the increased risk for aneurysm development in BAV individuals.

### Limitations of study

Firstly, the type of flow present in the BAV ascending aorta is highly complex and most likely characterized by helical flow and flow turbulences. Thus, flow-related methylation changes detected here, associated with high magnitudes of bi-directional oscillatory flow, may not be directly applicable to BAV-related flow. Also, the duration of flow exposure when using primary aortic endothelial cells isolated from dilated aortas of BAV and TAV patients were only 6 hours. Although longer exposure times would have been ideal, this was not achievable due to lack of resistance of aneurysmal cells to high oscillatory shear exposure in our flow system. Secondly, EA.hy926 cells were used in for investigation of flow-sensitive DNA methylation and its potential contribution to the non-dilated aortic methylation signature. Ideally, primary endothelial cells isolated from BAV and TAV non-dilated aorta should be used, but due to the small size of non-dilated aortic biopsies, isolation of sufficient number of endothelial cells is not possible. However, EA.hy926 cells have been shown to retain most of the features of HUVECs, including expression of adhesion molecules, and express vWF with the same morphological distribution as primary endothelial cells. Also, EA.hy926 cells show the same shear stress-induced characteristics as primary endothelial cells^52^ and in comparison to endothelial cells isolate from dilated aorta of BAV and TAV patients, which were used here, 40% of the identified genes were shared. Lastly, biopsies taken from dilated ascending aortas of BAV and TAV patients may differ due to the inflammatory cell composition, showing enrichment of inflammatory cells in dilated TAV. However, infiltration of inflammatory cells is most likely predominantly present in the adventitia layer of the aortic wall, which in this study is removed prior to methylation and gene expression analysis.

## Methods

### Patients

Samples of ascending aorta and internal thoracic artery were collected from the Advanced Study of Aortic Pathology (ASAP) biobank. The ASAP study includes 600 patients undergoing elective open-heart surgery for aortic valve and/or ascending aortic disease at the Karolinska University Hospital, Stockholm, Sweden. A detailed description of the study population can be found elsewhere^53^. Patients were classified according to aortic valve cuspidity and aortic dilatation. Aortic diameters of >45 mm were considered dilated (D), and aortas <40 mm were classified as non-dilated (ND). Patients with syndromic aortic pathologies, dissection and/or significant coronary artery disease (according to angiography) were excluded. The study was approved by the Human Research Ethics Committee at Karolinska Institutet (application number 2006/784-31/1), Stockholm, Sweden. Written informed consent was obtained from all patients according to the declaration of Helsinki, and methods were carried out in accordance with relevant guidelines.

Biopsies were taken from the anterior part of the ascending aorta, at the site of aortotomy a few cm above the aortic valve, and from the proximal portion of the right internal thoracic artery. The intima-medial was separated from the adventitia. Global DNA methylation was measured in 21 BAV (7 BAV-ND, 14 BAV-D) and 23 TAV (10 TAV-ND, 13 TAV-D). Gene expression was measure using Human Transcriptome Array 2.0 (Affymetrix), according to manufacturer’s instructions, or Affymetrix GeneChip Human Exon 1.0 ST array and protocols, as previously described^7^. Characteristics of patients are shown in Supplementary Table S1. Samples for primary endothelial cell isolation were taken from the outer curvature of BAV and TAV aneurysmal thoracic aortas during aortic surgery at the Almazov Federal Medical Research Center, St Petersburg, Russia. The specific clinical research protocol was approved by the local Ethics Committee of the Almazov Federal Medical Research Center (ethical permit number 12.26/2014).

### Cell culture and *in vitro* flow exposure

Human aortic endothelial cells (HAEC) were isolated from aneurysmal aorta of BAV and TAV patients, as described previously^54^, and cultured in Endothelial Cell Basal media with growth supplements, including 10% fetal calf serum and penicillin/streptomycin (PromoCell). Human EA.hy926^55^ cells were cultured in Dulbecco’s modified Eagle’s high glucose medium (DMEM) supplemented with 10% fetal calf serum and penicillin/streptomycin. For flow experiments, cells were plated on gelatin-coated teflon-bordered cell culture slides (75 × 25 × 10 mm, Flexcell International Corp.) and cultured for 40 hours with 5% CO^2^ at 37 °C. Culture slides were then inserted into a parallel plate Streamer device (Flexcell International Corp., Hillsborough, NC, US) and exposed to bidirectional oscillatory shear stress of ±12 dynes/cm^2^ or unidirectional laminar flow of 12 dynes/cm^2^ for 48 hours with 5% CO^2^ at 37 °C to identify changes in DNA methylation induced by disturbed and physiological flow, respectively. Of note, cells from BAV and TAV patients were flowed for 6 hours due to primary cells. BAV n = 6, TAV n = 7, and EA.hy926 n = 9. Flow was generated by a Masterflex L/S peristaltic pump, and the frequency of oscillation was determined by the Osci-Flow flow controller (Flexcell International Corp.). Corresponding BAV, TAV and EA.hy926 cells cultured under static conditions for the same period of time were used as controls. Total DNA was extracted.

### Illumina 450 K and EPIC methylation assay and data preprocessing

Genomic DNA was isolated using QIAamp DNA Mini Kit (QIAGEN), according to manufacturer’s instructions, and quantified using NanoDrop ND-1000 (NanoDrop Technologies). For each sample, 500 ng of DNA was bisulfite converted using EZ-96 DNA Methylation™ Kit (ZYMO Research, Orange, CA) according to manufacturer’s recommendations. DNA methylation was detected by Illumina Infinium Human Methylation 450, and Infinium Methylation EPIC BeadChip. Methylation levels were calculated and extracted by Illumina GenomeStudio® software. All samples were pre-processed and analyzed together using R^56^. Only CpG sites located on autosomes were selected; probes with low detection p-value, probes measuring SNPs, and non-CpG probes were excluded.

### Identification of differentially methylated CpGs and genomic regions

As recommended by Du *et al*.^57^, M-value were used to conduct differential methylation analysis. Welch’s t-test was carried out to detect individual differentially methylated probes (DMPs). To correct for multiple testing, P-values were adjusted via false discovery rate (FDR) estimation 10%. A genomic region is considered as a differentially methylated region (DMR) if they satisfy: 1) covers at least three CpG DMPs (P-value ≤ 0.05); 2) within a maximal inter-site distance of 1 KB. Scripts for detecting DMRs were adapted from^58^.

### Genomic Regions Enrichment of Annotation Tool and Gene Ontology Analysis

Genomic Regions Enrichment of Annotations Tool^59^ (GREAT, version 3.0, Stanford University, CA, US) was used to map DMRs to genes and analyze predicted functions. GREAT was performed against a whole genome background, using the ‘basal plus extension’ rule for assigning regulatory domains to genes with the following settings, Proximal 5 kb upstream and 1 kb downstream from the transcription start site (TSS), plus Distal up to 1000 kb. Curated regulatory domains were included. Gene Set Enrichment Analysis (GSEA) and the Molecular Signatures Database (MSigDB) resource v5.0^60^ were used for further investigation DMR-associated genes.

### Validation of Illumina 450 k by bisulfite Pyrosequencing

Methylation levels of selected CpGs were detected by a PyroMark Q96 ID System (Qiagen, Hilden, Germany) using the PyroGold SQA™ Reagent Kit (Qiagen, Hilden, Germany), according to manufacturer’s instructions. Quantification of methylation levels were performed using the methylation Software Pyro Q-CpG™.

### Statistical analysis

PCA, DMP and DMR-analyses were performed using R^56^. Differential gene expression was investigated using Student’s t-test assuming unequal variance. Differences in methylation levels assessed by bisulfite pyrosequencing were analyzed by Mann-Whitney U-test. Data expressed as mean ± standard deviation, unless otherwise stated. A P-value of P < 0.05 was considered statistically significant. Statistical significance for GREAT and GSEA/MSigDB analyses is presented as Binominal Bonferroni P-value and FDR 10%, respectively. For identification of flow-sensitive methylation changes, flow conditions were compared to static controls in BAV and TAV cells, respectively.

## Electronic supplementary material

Supplementary Information

## References

[1] Cotrufo M, Della Corte A (2009). The association of bicuspid aortic valve disease with asymmetric dilatation of the tubular ascending aorta: identification of a definite syndrome. J Cardiovasc Med.

[2] Laforest B, Nemer M (2012). Genetic insights into bicuspid aortic valve formation. Cardiology research and practice.

[3] Burris NS, Hope MD (2015). Bicuspid valve-related aortic disease: flow assessment with conventional phase-contrast MRI. Academic radiology.

[4] Guzzardi DG (2015). Valve-Related Hemodynamics Mediate Human Bicuspid Aortopathy: Insights From Wall Shear Stress Mapping. Journal of the American College of Cardiology.

[5] Merritt BA (2014). Association between leaflet fusion pattern and thoracic aorta morphology in patients with bicuspid aortic valve. Journal of magnetic resonance imaging: JMRI.

[6] Kjellqvist S (2013). *A combined proteomic and transcriptomic* approach shows diverging molecular mechanisms in thoracic aortic aneurysm development in patients with tricuspid- and bicuspid aortic valve. Molecular & cellular proteomics: MCP.

[7] Folkersen L (2011). Unraveling the divergent gene expression profiles in bicuspid and tricuspid aortic valve patients with thoracic aortic dilatation - the ASAP study. Mol Med.

[8] Aavik E (2015). Global DNA methylation analysis of human atherosclerotic plaques reveals extensive genomic hypomethylation and reactivation at imprinted locus 14q32 involving induction of a miRNA cluster. European heart journal.

[9] Maleki S (2016). Mesenchymal state of intimal cells may explain higher propensity to ascending aortic aneurysm in bicuspid aortic valves. Scientific reports.

[10] Jiang YZ, Manduchi E, Jimenez JM, Davies PF (2015). Endothelial epigenetics in biomechanical stress: disturbed flow-mediated epigenomic plasticity *in vivo* and *in vitro*. Arteriosclerosis, thrombosis, and vascular biology.

[11] Bjorck HM (2012). Characterization of shear-sensitive genes in the normal rat aorta identifies Hand2 as a major flow-responsive transcription factor. PloS one.

[12] Voutsadakis, I. A. Epithelial-Mesenchymal Transition (EMT) and Regulation of EMT Factors by Steroid Nuclear Receptors in Breast Cancer: A Review and in Silico Investigation. *Journal of clinical medicine* **5**, 10.3390/jcm5010011 (2016). 10.3390/jcm5010011 PMC4730136 26797644

[13] Min C, Eddy SF, Sherr DH, Sonenshein GE (2008). NF-kappaB and epithelial to mesenchymal transition of cancer. Journal of cellular biochemistry.

[14] Zhang L (2013). Hypoxia induces epithelial-mesenchymal transition via activation of SNAI1 by hypoxia-inducible factor -1alpha in hepatocellular carcinoma. BMC cancer.

[15] Sakabe M (2006). Rho kinases regulate endothelial invasion and migration during valvuloseptal endocardial cushion tissue formation. Developmental dynamics: an official publication of the American Association of Anatomists.

[16] Corallino S, Malabarba MG, Zobel M, Di Fiore PP, Scita G (2015). Epithelial-to-Mesenchymal Plasticity Harnesses Endocytic Circuitries. Frontiers in oncology.

[17] Brenet F (2011). DNA methylation of the first exon is tightly linked to transcriptional silencing. PloS one.

[18] Paloschi V (2015). Aneurysm development in patients with a bicuspid aortic valve is not associated with transforming growth factor-beta activation. Arteriosclerosis, thrombosis, and vascular biology.

[19] Sanchez-Tillo E (2012). EMT-activating transcription factors in cancer: beyond EMT and tumor invasiveness. Cellular and molecular life sciences: CMLS.

[20] Gouti M, Briscoe J, Gavalas A (2011). Anterior Hox genes interact with components of the neural crest specification network to induce neural crest fates. Stem cells.

[21] Lamouille S, Xu J, Derynck R (2014). Molecular mechanisms of epithelial-mesenchymal transition. Nature reviews. Molecular cell biology.

[22] Connelly JJ (2013). Epigenetic regulation of COL15A1 in smooth muscle cell replicative aging and atherosclerosis. Human molecular genetics.

[23] Mousa AA (2012). Preeclampsia is associated with alterations in DNA methylation of genes involved in collagen metabolism. The American journal of pathology.

[24] Grewal N (2014). Ascending aorta dilation in association with bicuspid aortic valve: a maturation defect of the aortic wall. The Journal of thoracic and cardiovascular surgery.

[25] Kostina AS (2016). Notch-dependent EMT is attenuated in patients with aortic aneurysm and bicuspid aortic valve. Biochimica et biophysica acta.

[26] Yabana N, Shibuya M (2002). Adaptor protein APS binds the NH2-terminal autoinhibitory domain of guanine nucleotide exchange factor Vav3 and augments its activity. Oncogene.

[27] Rico-Bautista E, Negrin-Martinez C, Novoa-Mogollon J, Fernandez-Perez L, Flores-Morales A (2004). Downregulation of the growth hormone-induced Janus kinase 2/signal transducer and activator of transcription 5 signaling pathway requires an intact actin cytoskeleton. Experimental cell research.

[28] Wang S (2015). Regulation of endothelial cell proliferation and vascular assembly through distinct mTORC2 signaling pathways. Molecular and cellular biology.

[29] Chambers KF (2011). Stromal upregulation of lateral epithelial adhesions: gene expression analysis of signalling pathways in prostate epithelium. Journal of biomedical science.

[30] Hardie DG (2007). AMP-activated/SNF1 protein kinases: conserved guardians of cellular energy. Nature reviews. Molecular cell biology.

[31] Ali OA (2014). Interactions between inflammatory activation and endothelial dysfunction selectively modulate valve disease progression in patients with bicuspid aortic valve. Heart.

[32] Tzemos N (2010). Endothelial function, carotid-femoral stiffness, and plasma matrix metalloproteinase-2 in men with bicuspid aortic valve and dilated aorta. Journal of the American College of Cardiology.

[33] Nackman GB, Fillinger MF, Shafritz R, Wei T, Graham AM (1998). Flow modulates endothelial regulation of smooth muscle cell proliferation: a new model. Surgery.

[34] Moonen JR (2015). Endothelial-to-mesenchymal transition contributes to fibro-proliferative vascular disease and is modulated by fluid shear stress. Cardiovascular research.

[35] Mantilidewi KI (2014). Shear stress-induced redistribution of vascular endothelial-protein-tyrosine phosphatase (VE-PTP) in endothelial cells and its role in cell elongation. The Journal of biological chemistry.

[36] Han J (2015). Flow shear stress differentially regulates endothelial uptake of nanocarriers targeted to distinct epitopes of PECAM-1. Journal of controlled release: official journal of the Controlled Release Society.

[37] Mahler GJ, Frendl CM, Cao Q, Butcher JT (2014). Effects of shear stress pattern and magnitude on mesenchymal transformation and invasion of aortic valve endothelial cells. Biotechnology and bioengineering.

[38] Egorova AD (2011). Lack of primary cilia primes shear-induced endothelial-to-mesenchymal transition. Circulation research.

[39] Mahmoud MM (2017). Shear stress induces endothelial-to-mesenchymal transition via the transcription factor Snail. Scientific reports.

[40] Bonachea EM (2014). Use of a targeted, combinatorial next-generation sequencing approach for the study of bicuspid aortic valve. BMC medical genomics.

[41] Kessler K (2014). Angiogenesis and remodelling in human thoracic aortic aneurysms. Cardiovascular research.

[42] Paloschi V (2011). Impaired splicing of fibronectin is associated with thoracic aortic aneurysm formation in patients with bicuspid aortic valve. Arteriosclerosis, thrombosis, and vascular biology.

[43] Wågsater D (2013). Impaired collagen biosynthesis and cross-linking in aorta of patients with bicuspid aortic valve. Journal of the American Heart Association.

[44] Franco CA, Li Z (2009). *SRF* in angiogenesis: branching the vascular system. Cell adhesion & migration.

[45] Manabe I, Owens GK (2001). Recruitment of serum response factor and hyperacetylation of histones at smooth muscle-specific regulatory regions during differentiation of a novel P19-derived *in vitro* smooth muscle differentiation system. Circulation research.

[46] Suzuki T, Osumi N, Wakamatsu Y (2010). Stabilization of ATF4 protein is required for the regulation of epithelial-mesenchymal transition of the avian neural crest. Developmental biology.

[47] Ehrlich M (2009). DNA hypomethylation in cancer cells. Epigenomics.

[48] de la Pompa JL, Epstein JA (2012). Coordinating tissue interactions: Notch signaling in cardiac development and disease. Developmental cell.

[49] Pan S (2017). DNA methylome analysis reveals distinct epigenetic patterns of ascending aortic dissection and bicuspid aortic valve. Cardiovascular research.

[50] Billaud M (2017). Elevated oxidative stress in the aortic media of patients with bicuspid aortic valve. The Journal of thoracic and cardiovascular surgery.

[51] Zaidi S (2013). De novo mutations in histone-modifying genes in congenital heart disease. Nature.

[52] Huang X (2013). *Rac1 mediates laminar shear* stress-induced vascular endothelial cell migration. Cell adhesion & migration.

[53] Jackson V (2011). Bicuspid aortic valve leaflet morphology in relation to aortic root morphology: a study of 300 patients undergoing open-heart surgery. European journal of cardio-thoracic surgery: official journal of the European Association for Cardio-thoracic Surgery.

[54] Malashicheva A (2016). Phenotypic and Functional Changes of Endothelial and Smooth Muscle Cells in Thoracic Aortic Aneurysms. International journal of vascular medicine.

[55] Edgell CJ, McDonald CC, Graham JB (1983). Permanent cell line expressing human factor VIII-related antigen established by hybridization. Proceedings of the National Academy of Sciences of the United States of America.

[56] R: A language and environment for statistical computing (R Foundation for Statistical Computing, 2015).

[57] Du P (2010). Comparison of Beta-value and M-value methods for quantifying methylation levels by microarray analysis. BMC bioinformatics.

[58] Slieker RC (2013). *Identification and systema*tic annotation of tissue-specific differentially methylated regions using the Illumina 450 k array. Epigenetics & chromatin.

[59] McLean CY (2010). GREAT improves functional interpretation of cis-regulatory regions. Nature biotechnology.

[60] Subramanian A (2005). Gene set enrichment analysis: a knowledge-based approach for interpreting genome-wide expression profiles. Proceedings of the National Academy of Sciences of the United States of America.

