# Supplementary material for: Altered DNA methylation indicates an oscillatory flow mediated epithelial-to-mesenchymal transition signature in ascending aorta of patients with bicuspid aortic valve
Source: Sci Rep. 2018 Feb 9;8:2777. doi: 10.1038/s41598-018-20642-4 (PMC5807320; doi:10.1038/s41598-018-20642-4)
Supplement: Supplementary file 1 — Supplementary Information [file 41598_2018_20642_MOESM1_ESM.pdf]

Supplementary materials

Altered DNA methylation indicates an oscillatory flow mediated epithelial-to-mesenchymal transition signature in ascending aorta of patients with bicuspid aortic valve

Short title: BAV, DNA methylation and EndMT/EMT

Hanna M. Björck, Lei Du, Silvia Pulignani, Valentina Paloschi, Karin Lundströmer, Alexandra S. Kostina, Cecilia Österholm, Anna Malashicheva, Anna Kostareva, MIBAVA Leducq consortium, Arturo Evangelista, Gisela Teixidó-Tura, Shohreh Maleki, Anders Franco-Cereceda, Per Eriksson

**Page**	**Content**
2	Detailed Material and Methods
5	Supplementary Table S1, Patient characteristics
6	Supplementary Table S2, Pyro primer sequences
6	Supplementary Table S3, Gene expression cell type markers, BAV-ND vs. TAV-ND
6	Supplementary Table S4, DMRs and associated genes, BAV-ND vs. TAV-ND
7	Supplementary Table S5, Genes differentially methylated in nonDIL aorta and in response to bi-directional oscillatory flow
8	Supplementary Table S6, Hypo- and hypermethylated DMRs and associated genes in ECs exposed to bi-directional oscillatory flow
8	Supplementary Table S7, Differentially expressed DMR-genes, static vs. bi-directional oscillatory flow, BAV and TAV ECs
8	Supplementary Table S8, KEGG pathway analysis, DMR-genes up-regulated in response to bi-directional oscillatory flow, BAV and TAV ECs
9	Supplementary Table S9, Genes included in KEGG-terms ‘Pathways in cancer’ and ‘MAPK-signaling’, BAV and TAV ECs
10	Supplementary Table S10, Hypo- and hypermethylated DMRs and associated genes, dilated BAV aorta
10	Supplementary Table S11, Hypomethylated EMT/EndMT transcription factors, BAV dilated aorta
10	Supplementary Table S12, KEGG pathway analysis, DMR-genes overlapping dilated BAV and BAV ECs exposed to bi-directional oscillatory flow
11	Supplementary Fig. S1, Onotolgy analysis DMR-genes, non-dilated aorta
11	Supplementary Fig. S2, Hallmark analysis, differentially expressed genes, Ea.hy926 cells exposed to oscillatory flow
12	Supplementary Fig. S3, DNA methylation and gene expression density distribution plots
13	Supplementary Fig. S4, Validation DNA methylation levels, pyrosequencing
13	Supplementary Fig. S5, DNA methylation, internal thoracic arteries
14	Supplementary Fig. S6, Onotolgy analysis DMR-genes, dilated aorta
15	References

Detailed Material and Methods

Patients and tissue samples

Samples of ascending aorta and internal thoracic artery were collected from the Advanced Study of Aortic Pathology (ASAP) biobank. The ASAP study includes 600 patients undergoing elective open-heart surgery for aortic valve and/or ascending aortic disease at the Karolinska University Hospital, Stockholm, Sweden. A detailed description of the study population can be found elsewhere.[1] Patients were classified according to aortic valve cuspidity and aortic dilatation based on visual inspection and transesophagal diameter measurements of the ascending aorta, respectively, during surgery. Diameter measurements were obtained from the point where the ascending aorta showed maximal dilatation. Aortic diameters of >45 mm were considered dilated (D), and aortas <40 mm were classified as non-dilated (ND). Patients with ND aorta were operated due to valve disease, leaving the ascending aorta intact. Patients with syndromic aortic pathologies, dissection and/or significant coronary artery disease (according to angiography) were excluded. The study was approved by the Human Research Ethics Committee at Karolinska Institutet (application number 2006/784-31/1), Stockholm, Sweden. Written informed consent was obtained from all patients according to the declaration of Helsinki, and methods were carried out in accordance with relevant guidelines.

Biopsies were taken from the anterior part of the ascending aorta, at the site of aortotomy, a few cm above the aortic valve, and from the proximal portion of the right internal thoracic artery. The intima-medial was separated from the adventitia. Biopsies for DNA isolation were snap frozen on dry ice at the site of surgery and stored at -80°C pending extraction. RNA was extracted from the intima-medial of dilated aorta (n=14 BAV-D and n=13 TAV-D) and used for expression profiling using Affymetrix GeneChip® Human Exon 1.0 ST array and protocols, as previously described.[2] The Human Transcriptome Array 2.0 (Affymetrix) was used for gene expression profiling of flowed primary aortic endothelial cells and Ea.hy926 cells, according to manufacturers instruction. Global DNA methylation was measured in 21 BAV (7 BAV-ND, 14 BAV-D) and 23 TAV (10 TAV-ND, 13 TAV-D). Characteristics of patients are shown in Supplementary Table S1.

Samples for primary endothelial cell isolation were taken from the outer curvature of BAV and TAV aneurysmal thoracic aortas during aortic surgery at the Almazov Federal Medical Research Center, St Petersburg, Russia. The specific clinical research protocol was approved by the local Ethics Committee of the Almazov Federal Medical Research Center (ethical permit number 12.26/2014).

Cell culture and in vitro flow exposure

The human EC line EA.hy926[3] was routinely cultured in Dulbecco's modified Eagle's high glucose medium (DMEM) supplemented with 10% fetal calf serum and penicillin/streptomycin. Human aortic endothelial cells (HAEC) were isolated from dilated aorta of BAV and TAV patients, as described previously[4], and cultured in Endothelial Cell Basal media with growth supplements, including 10% fetal calf serum and penicillin/streptomycin (PromoCell). Cells (from passage 3-5) were plated on gelatin-coated teflon-bordered cell culture slides (75x25x10 mm, Flexcell International Corp.) and cultured for 40 hours with 5% CO2 at 37°C to allow confluency. Culture slips were then inserted into a parallel plate Streamer device (Flexcell International Corp., Hillsborough, NC, US), incorporated into a closed loop containing culture medium, and exposed to bidirectional oscillatory shear stress of ±12 dynes/cm^2 or unidirectional laminar flow of 12 dynes/cm^2 for 48 hours with 5% CO2 at 37°C to identify changes in DNA methylation induced by disturbed and and physiological flow, respectively. The flow was generated by a Masterflex L/S peristaltic pump and the frequency of oscillation was determined by the Osci-Flow flow controller (Flexcell International Corp.). Of note, cells from BAV and TAV patients were flowed for 6 hours due to primary cells. BAV n=6, TAV n=7, and EAHy.926 n=9. Corresponding BAV, TAV and EaHy.926 cells cultured under static conditions were used as controls. Total DNA was extracted from and analyzed for global DNA methylation.

Illumina 450K methylation assay and data preprocessing

Genomic DNA was isolated using QIAamp DNA Mini Kit (QIAGEN), according to manufacturer's instructions, and quantified using NanoDrop ND-1000 (NanoDrop Technologies). For each sample, 500 ng of DNA was bisulfite converted using EZ-96 DNA Methylation™ Kit (ZYMO Research, Orange, CA) according to manufacturer's recommendations. DNA methylation was measured using Infinium HumanMethylation 450, and Infinium Methylation EPIC BeadChips (Illumina Inc.), covering 99% of RefSeq genes. All samples were converted, fragmented, hybridized to array and further processed at the core facility for Bioinformatics and Expression Analysis at the Department of Biosciences and Nutrition, Novum, KI Huddinge. To avoid sampling bias, samples were randomized prior to conversion and subsequent analysis.

Methylation levels (beta values) were calculated and extracted by Illumina GenomeStudio® software. All samples were pre-processed and analyzed together using R[5] scripts including packages lumi[6-9] and methylumi[10]. For the quality control purpose, only CpG sites that are located on autosomes are selected; we also excluded probes with low detection p-value, and probes measuring SNPs. Non-CpG probes were also removed from further analyses. A total of 467 904 CpGs remained after quality control and filtering. The majority of CpGs were located within promoter regions (38.3%), defined here as the first exon of RefSeq genes, the 5'UTR or up 1.5kb upstream of TSS, or within the gene body (33.4%). Remaining CpGs were located at the 3'UTR (3.5%) or were not annotated to a specific gene region (24.8%).

Identification of differentially methylated CpGs and genomic regions

As recommended by Du *et al.,*[7] we used M-value to conduct differential methylation analysis. Welch's t-test was carried out to detect individual differentially methylated probes (DMPs). To correct for multiple testing, P-values were adjusted via false discovery rate (FDR) estimation 10%. A genomic region is considered as a differentially methylated region (DMR) if they satisfy 1) covers at least three DMPs (P-value ≤0.05), and 2) within a maximal inter-site distance of 1KB. Scripts for detecting DMRs were adapted from[11]. It is worth noting that this setting is stringent; each P-value is simplified and multiple comparisons within a region are not considered. Detection strategies that incorporate this information are included in our future research.

Genomic Regions Enrichment of Annotation Tool and Gene Ontology Analysis

Genomic Regions Enrichment of Annotations Tool[12] (GREAT, version 3.0, Stanford University, CA, US) was used to analyze the predicted function of the identified DMRs. In GREAT, this is done as follows. Each gene is first assigned a regulatory domain based on the association rule of choice. Then each genomic region is associated with all genes whose regulatory domain it overlaps. In our analysis, GREAT was performed against a whole genome background, using the 'basal plus extension' rule for assigning regulatory domains to genes with the following settings, Proximal 5 kb upstream and 1 kb downstream from the transcription start site, plus Distal up to 1000 kb. Curated regulatory domains were included. Gene ontology terms related to biological process (BP), molecular function (MF) and cellular compartment (CC) were considered. Gene Set Enrichment Analysis (GSEA) and the Molecular Signatures Database (MSigDB) resource v5.0[13] was used to investigate genes associated with DMRs. The Kyoto Encyclopedia of Genes and Genomes (KEGG) pathways database was used for identification of pathways, and Hallmark analysis identified specific and well-defined biological processes based on their coordinate expression.

Validation of Illumina 450k by bisulfite Pyrosequencing

Methylation levels of selected CpGs were measured by pyrosequencing technology to validate the Illumina 450k Array. From each sample, 400 ng DNA was bisulfite converted using the EZ DNA Methylation-Gold™ Kit (Zymo research), according to manufacturer's instructions. Specific genomic regions were then amplified by PCR, in which one primer was tagged with biotin. Biotinylated PCR products were immobilized on streptavidin-coated beads, followed by separation of the two DNA strands by denaturation in NaOH. Single immobilized DNA strands were washed, neutralized, and

annealed with the sequencing primer (sequences in Supplementary Table S2). Methylation levels were detected by a PyroMark Q96 ID System (Qiagen, Hilden, Germany) using the PyroGold SQA™ Reagent Kit (Qiagen, Hilden, Germany), according to manufacturer's instructions. Quantification of methylation levels were performed using the methylation Software Pyro Q-CpG™.

Statistical analysis

PCA, DMP and DMR analyses were performed using R (2.13.0)[5]. Differential gene expression was investigated using Student's t-test assuming unequal variance. Differences in methylation levels assessed by bisulfite pyrosequencing were analyzed by Mann-Whitney U-test (GraphPad Prism 5, La Jolla, CA); data expressed as mean ± standard deviation, unless otherwise stated. A P-value of $P<0.05$ was considered statistically significant. Statistical significance for GREAT and GSEA/MSigDB analyses is presented as Binominal Bonferroni P-value and FDR 10%, respectively. For identification of flow-sensitive methylation changes, flow conditions were compared to static controls in BAV and TAV cells, respectively.

Supplementary Table S1. Patient characteristics

**Patient characteristics**						
	**BAV-ND**	**TAV-ND**	***P***	**BAV-D**	**TAV-D**	***P***
**N**	7	10		14	13	
**Age, years**	60 (3)	72 (3)	0.028	61 (3)	63 (4)	0.61
**Male gender**	4 (57)	6 (60)	1.0	11 (79)	7 (54)	0.24
**Smoking, current smokers**	2 (29)	2 (20)	1.0	1 (7)	2 (15)	0.60
**BMI, Kg/m^2**	28.6 (2.4)	26.3 (1.3)	0.40	25.6 (1.1)	26.0 (1.0)	0.76
**Triglycerides, mmol/L**	1.18 (0.24)	0.90 (0.12)	0.28	1.29 (0.20)	1.24 (0.17)	0.86
**Cholesterol, mmol/L**	4.94 (0.21)	4.93 (0.34)	0.98	5.05 (0.27)	4.79 (0.30)	0.52
**hsCRP, mg/L**	2.61 (0.92)	2.16 (0.60)	0.68	2.09 (0.65)	9.06 (5.95)	0.24
**Systolic BP, mmHg**	123 (4)	138 (6)	0.08	136 (7)	150 (5)	0.11
**Diastolic BP, mmHg**	80 (4)	77 (4)	0.65	83 (5)	76 (4)	0.31
**Diabetes**	1 (14)	2 (20)	1.0	2 (14)	0 (0)	0.481
**Aortic aneurysm and valve disease**						
**Ascending aortic diameter, mm**	36.6 (1.2)	32.6 (1.4)	0.07	49.3 (0.72)	54.3 (1.85)	0.016
**Aortic valve stenosis**	5 (71)	9 (90)	0.54	9 (64)	0 (0)	<0.001
**Aortic valve regurgitation**	3 (43)	1 (10)	0.25	6 (43)	9 (69)	0.25
**Medication**						
**Beta blockers**	3 (43)	4 (40)	1.0	4 (29)	8 (62)	0.13
**Ca antagonists**	1 (14)	3 (30)	0.60	1 (7)	1 (8)	1.0
**ACE inhibitors**	1 (14)	1 (10)	1.0	5 (36)	5 (38)	1.0
**Statins**	1 (14)	5 (50)	0.30	5 (36)	5 (38)	1.0

BMI, body mass index; hsCRP, high-sensitive C-reactive protein; BP, blood pressure; ACE, Angiotensin-converting enzyme. Diabetes was recorded via questionnaire of medical history. Continuous variables are presented as mean (SD) and ordinal variables are presented as n (%). *P* indicates significance for differences between groups using Student's t-test for continuous variables and a Fisher's exact test for nominal variables. Non-dilated (ND) aorta <40mm; Dilated (D) aorta >45mm.

Supplementary Table S2. Pyro primer sequences

Pyro primer sequences				
**CpG-site**	**Gene**	**Chr**	**Pyro Sequencing primer 5' > 3'**	**Amplicon size**
1_cg04912273	SLC9A1	1	TTGCGGTCGTTTATTTTGGT	250
2_cg04987335	SLC1A2	11	GGAAGGAATATATGATAGTAGTTAAGAAA	184
3_cg06225294	ZBTB20	3	TGGAGAAAAGATTTTTTGTG	148
4_cg06420129	TNRC6A	16	AGTTAATGTTTAGTATAGAGAGGTAGGTA	179
5_cg12830671	CDH23	10	TTATAGTGTGTAGGTTTGTAGGGG	200
6_cg20906621	AGTR1	3	CCGATATAACTCTACAAAACG	138

Supplementary Table S3. Gene expression cell type markers, BAV (n=31) vs. TAV (n=23), non-dilated aorta.

Gene	FDR q
Endothelial cells	
PECAM1	0.629
VWF	0.915
Vascular smooth muscle cells	
MYH11	0.629
ACTA2	0.388
CALD1	0.222
SMTN	0.629
TAGLN	0.629
MYOCD	0.222
VIM	0.506
Macrophages	
CD83	0.431
CD68	0.222
CD163	0.222
Inflammation	
VCAM1	0.629
NFKB	0.915
TNF	0.222

FDR q indicates significance for differences between groups using Student's t-test.

Supplementary Table S4. Hyper- and hypomethylated DMRs, and associated genes, non-dilated BAV vs. TAV aorta (fold change ±10%).

See separate pdf-file.

Supplementary Table S5. Genes differentially methylated in nonDIL aorta and in response to bi-directional oscillatory flow.

Genes differentially methylated in NonDIL aorta and in repsonse to oscillatory flow					
AATK	CUX1	GSE1	NKX3-2	PUSL1	WDR46
ABCB5	CXXC5	GTF2H4	NKX6-2	PXDC1	WDR60
ACAP3	CYP26B1	HCK	NOTCH4	PXDN	WRAP73
ACSS1	DDR1	HLA-B	NPVF	RHCG	WSCD2
ADARB2	DIO3	HLA-DMB	NR2F1	RING1	ZBTB16
ADRA2C	DIP2C	HLA-DPA1	NUP210	RPP21	ZFHX3
AGAP1	DLX6	IDI1	NXN	RPTOR	ZMYND11
AGAP3	DNM3	IGF2	OTOP1	RTKN2	ZNF236
AMPD3	DPYSL4	JAKMIP3	P2RX2	SCARB1	ZNF32
ARHGAP44	DYSF	KALRN	PARP10	SDK1	ZNF423
ARHGEF10	ELFN1	KCNQ1	PARP8	SH2B2	ZNF469
ARHGEF16	EN1	KEAP1	PDCD1	SIX3	ZNF664
ARHGEF7	ENSG00000171282	KIF7	PER1	SORCS2	
ARPC1B	ENSG00000269375	LDB3	PEX5	SOX1	
ATM	EOMES	LSMEM2	PFDN6	SP5	
BAG6	EPHB3	LY6G6C	PFKP	SP8	
BAIAP2	ESR1	MAD1L1	PGBD5	SPACA7	
BANP	F7	MAMSTR	PHLDA2	SPTB	
BCL6	FAM105B	MAP1LC3B2	PIGC	SUPT3H	
BNIP3	FAM53B	MARCH10	PITRM1	SYNE1	
C1QTNF2	FAM65B	MBP	PLEC	TANC2	
C6orf25	FBLN2	MCF2L	PMEPA1	TBX2	
CACNB2	FGF18	MED13L	PMFBP1	TBX3	
CALCOCO1	FGFR4	MEGF6	POLG	TFAP2A	
CAMKMT	FOXK1	MGMT	POU3F2	THTPA	
CCDC92	FOXN3	MOB2	POU5F2	TLE3	
CCM2L	FREM2	MRPL23	PPT2-EGFL8	TMEM105	
CD3EAP	FZD5	MTRNR2L8	PRDM1	TMEM184A	
CDKN1C	GAD1	MUC8	PRDM6	TMEM53	
CECR5	GALNT9	MYH11	PRIMA1	TPCN2	
CHFR	GATA6	MYLK	PRKAG2	TPO	
CHMP6	GBX1	MYOM2	PRKAR1B	TPRG1L	
CHRM1	GBX2	NADK	PRRC2A	TRIO	
CLIC5	GLOD4	NCOR2	PRRT1	TTC8	
COL11A2	GLRX3	NEU4	PSAPL1	TUBB2B	
CPE	GNAI2	NEUROG1	PSD2	VAX2	
CRYBB2	GOLGA3	NFE2L3	PSMB9	VIPR2	
CTAGE1	GPR123	NKD2	PTP4A3	VPS52	
CUTA	GPSM3	NKX3-1	PTPLB	VWA7	

Supplementary Table S6a-b. Hypo- and hypermethylated DMRs and associated genes, ECs exposed to bi-directional oscillatory flow

See separate pdf-file.

Supplementary Table S7. Differentially expressed DMR-genes, static vs. bi-directional oscillatory flow, BAV and TAV ECs (FDR10%)

See separate pdf-file.

Supplementary Table S8. KEGG pathway analysis, DMR-genes up-regulated in response to bi-directional oscillatory flow, BAV and TAV ECs

Genes up-regulated in BAV		Genes up-regulated in TAV	
**Description**	**FDR q**	**Description**	**FDR q**
MAPK signaling pathway	3.61E-9	Pathways in cancer	1.71E-7
Pathways in cancer	3.21E-7	MAPK signaling pathway	2.93E-6
Endocytosis	1.44E-5	Focal adhesion	8.42E-4
Long-term potentiation	1.44E-5	Leishmania infection	1.67E-3
Adherens junction	1.85E-5	Small cell lung cancer	2.45E-3
TGF-beta signaling pathway	3.94E-5	Dilated cardiomyopathy	2.91E-3
Prostate cancer	4.26E-5	mTOR signaling pathway	8.62E-3
GnRH signaling pathway	8.73E-5	Systemic lupus erythematosus	1.01E-2
Melanogenesis	9.85E-4	Acute myeloid leukemia	1.01E-2

BAV, n=333 genes; TAV, n=135 genes

Supplementary Table S9. Genes included in KEGG-terms 'Pathways in cancer' and 'MAPK-signaling', BAV and TAV ECs, respectively

BAV

Pathways in cancer		MAPK-signaling	
**Gene**	**Description**	**Gene**	**Description**
TRAF6	TNF receptor-associated factor 6	TRAF6	TNF receptor-associated factor 6
KRAS	v-Ki-ras2 Kirsten rat sarcoma viral oncogene homolog	KRAS	v-Ki-ras2 Kirsten rat sarcoma viral oncogene homolog
BRAF	v-raf murine sarcoma viral oncogene homolog B1	BRAF	v-raf murine sarcoma viral oncogene homolog B1
FGFR1	fibroblast growth factor receptor 1	FGFR1	fibroblast growth factor receptor 1
CBL	Cas-Br-M (murine) ecotropic retroviral transforming sequence	ATF4	activating transcription factor 4 (tax-responsive enhancer element B67)
EP300	E1A binding protein p300	PPP3CC	protein phosphatase 3, catalytic subunit, gamma isozyme
CTNNB1	catenin (cadherin-associated protein), beta 1, 88kDa	RAP1B	RAP1B, member of RAS oncogene family
CTNNA1	catenin (cadherin-associated protein), alpha 1, 102kDa	NLK	nemo-like kinase
BMP2	bone morphogenetic protein 2	MAP2K4	mitogen-activated protein kinase kinase 4
E2F3	E2F transcription factor 3	MAP2K3	mitogen-activated protein kinase kinase 3
FZD8	frizzled family receptor 8	TAB2	TGF-beta activated kinase 1/MAP3K7 binding protein 2
ITGB1	integrin, beta 1 (fibronectin receptor, beta polypeptide, antigen CD29 includes MDF2, MSK12)	GADD45B	growth arrest and DNA-damage-inducible, beta
PTGS2	prostaglandin-endoperoxide synthase 2 (prostaglandin G/H synthase and cyclooxygenase)	NR4A1	nuclear receptor subfamily 4, group A, member 1
JAK1	Janus kinase 1	TAOK1	TAO kinase 1
BIRC3	baculoviral IAP repeat containing 3	SRF	serum response factor (c-fos serum response element-binding transcription factor)
		DUSP16	dual specificity phosphatase 16

TAV

Pathways in cancer		MAPK-signaling	
**Gene**	**Description**	**Gene**	**Description**
PDGFA	platelet-derived growth factor alpha polypeptide	PDGFA	platelet-derived growth factor alpha polypeptide
NFKB1	nuclear factor of kappa light polypeptide gene enhancer in B-cells 1	NFKB1	nuclear factor of kappa light polypeptide gene enhancer in B-cells 1
NFKB2	nuclear factor of kappa light polypeptide gene enhancer in B-cells 2 (p49/p100)	NFKB2	nuclear factor of kappa light polypeptide gene enhancer in B-cells 2 (p49/p100)
ITGB1	integrin, beta 1 (fibronectin receptor, beta polypeptide, antigen CD29 includes MDF2, MSK12)	TAB2	TGF-beta activated kinase 1/MAP3K7 binding protein 2
BIRC2	baculoviral IAP repeat containing 2	NLK	nemo-like kinase
VEGFA	vascular endothelial growth factor A	MAPKAPK2	mitogen-activated protein kinase-activated protein kinase 2
PGF	placental growth factor	GADD45B	growth arrest and DNA-damage-inducible, beta
PTGS2	prostaglandin-endoperoxide synthase 2 (prostaglandin G/H synthase and cyclooxygenase)	RASGRP3	RAS guanyl releasing protein 3 (calcium and DAG-regulated)
TCF7	transcription factor 7 (T-cell specific, HMG-box)	DUSP16	dual specificity phosphatase 16
RUNX1	runt-related transcription factor 1		
KITLG	KIT ligand		

Supplementary Table S10a-b. Hypo- and hypermethylated DMRs and associated genes, dilated BAV aorta (fold change ±10%)

See separate pdf-file.

Supplementary Table S11. Key EMT/EndMT transcription factors hypomethylated in dilated BAV aorta

Gene	BAV-D vs. TAV-D	Gene location
TWIST1	no DMR	-
TWIST2	R.818 (-412076)	Body
	R.820 (+305304)	Body
	R.823 (+439751)	TSS1500
	R.824 (+456295)	5'UTR. 1stExon. TSS200
SNAI1	no DMR	-
SNAI2	R.2304 (+50945)	TSS1500
ZEB1	R.2507 (+2323)	Body
ZEB1	R.2508 (+608094)	Body
ZEB2	no DMR	-

Supplementary Table S12. KEGG pathway analysis, DMR-genes overlapping BAV-D and BAV ECs exposed to bi-directional oscillatory flow

Gene Set Name	FDR q
Pathways in cancer	2.02e-13
Small cell lung cancer	1.17e-8
Regulation of actin cytoskeleton	2.89e-8
MAPK signaling pathway	7.32e-8
Wnt signaling pathway	1.4e-7
TGF-beta signaling pathway	3.78e-6
Focal adhesion	1.02e-5
ErbB signaling pathway	2.38e-5
Prostate cancer	2.71e-5
Phosphatidylinositol signaling system	3.29e-5

Supplementary Figure S1

Ontology analysis (GO term molecular function) of hypomethylated DMR-genes in the non-dilated BAV aorta. N=7 BAV-ND and N=10 TAV-ND. Methylation values corrected for age.

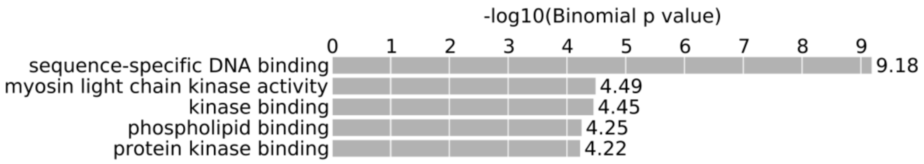

Supplementary Figure S2

Hallmark analysis of differentially expressed genes in Ea.hy926 cells exposed to oscillatory flow compared to static conditions. Red line indicates raw P-value=0.05; Green line indicates FDR q=0.05.

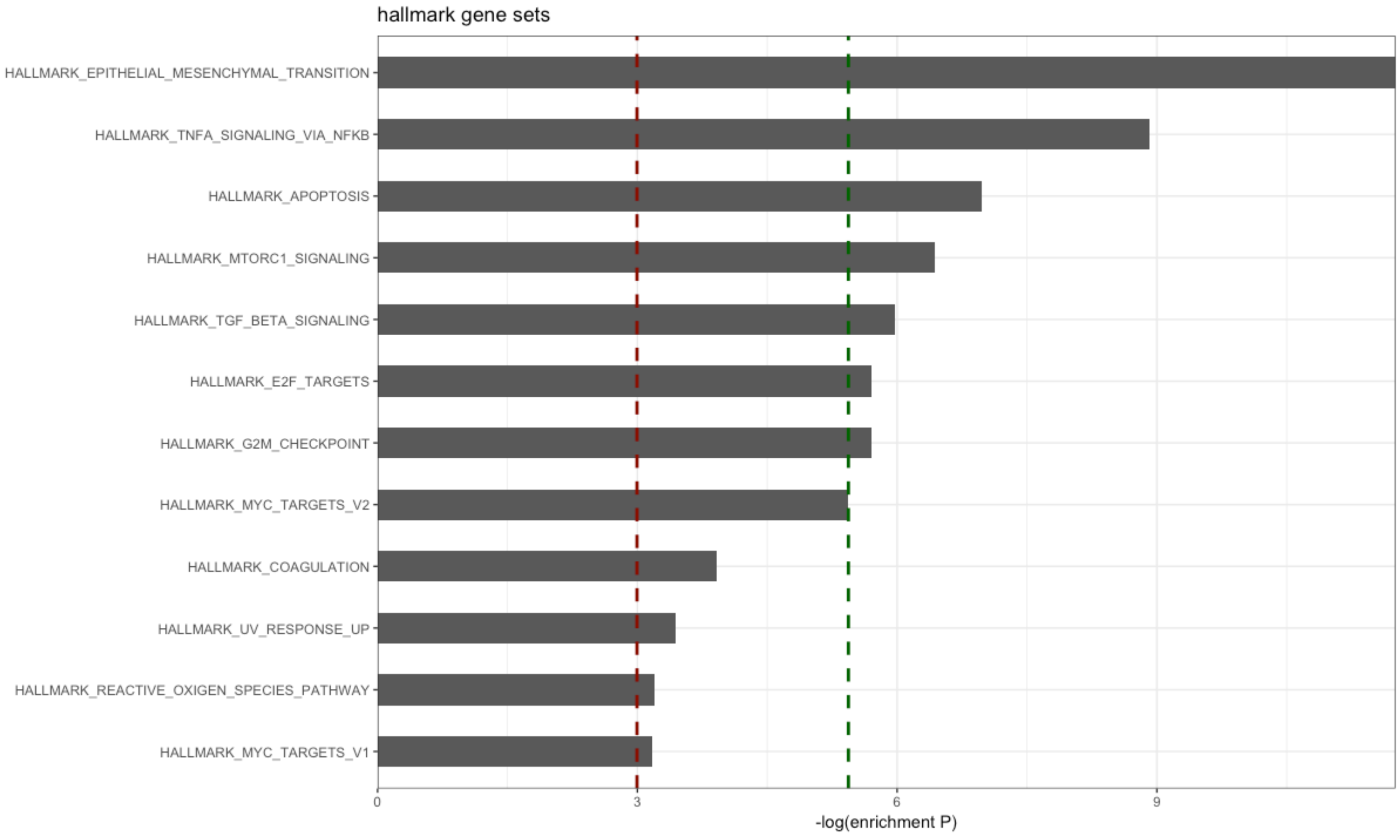

Supplementary Figure S3

DNA methylation and gene expression density distribution plots. Ascending aortic DNA methylation and gene expression levels are measured in the same aortic biopsy. N=14 BAV (pink) and N=13 TAV (blue).

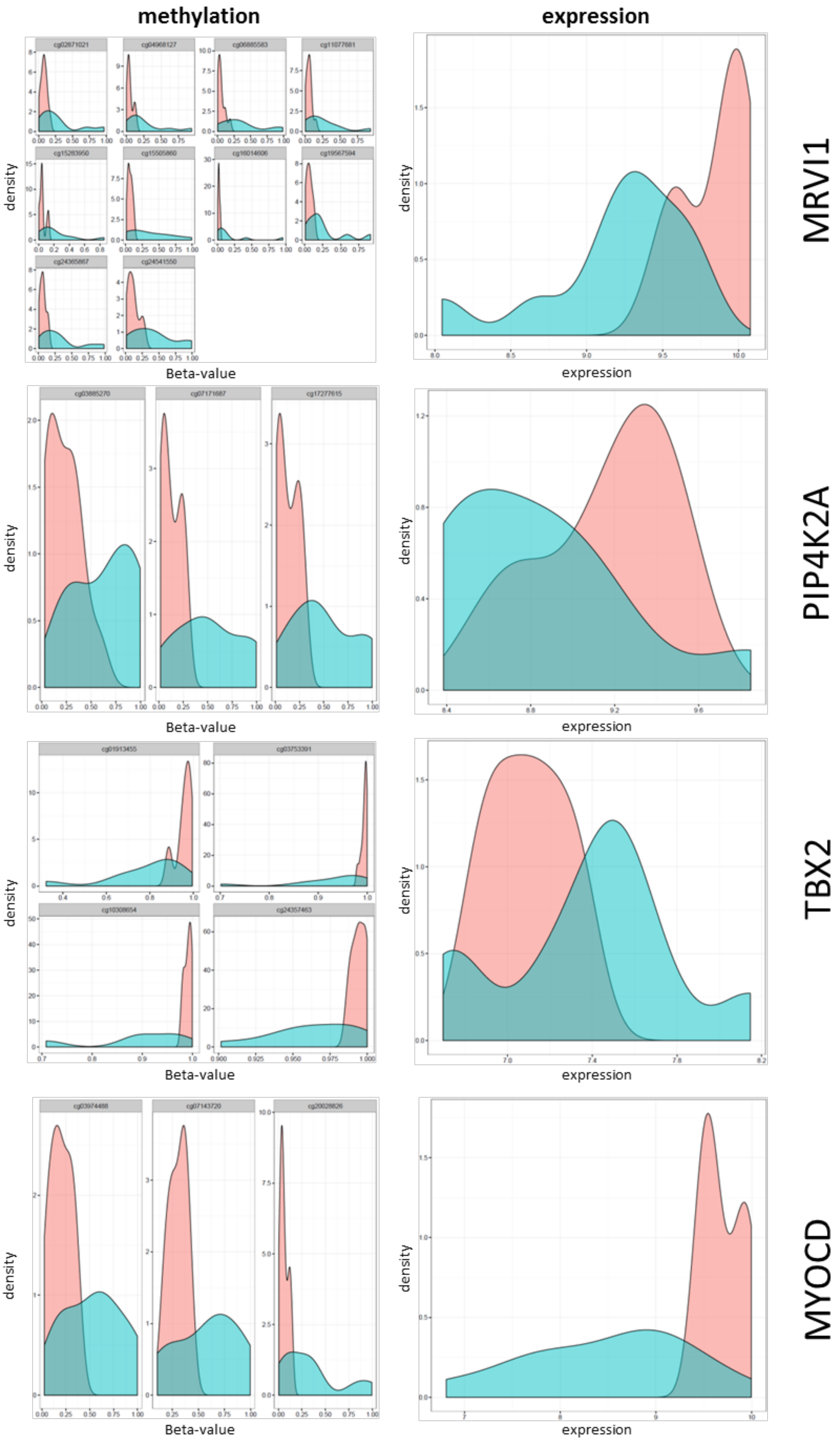

Supplementary Figure S4

DNA methylation levels of six selected CpGs measured using pyrosequencing. DNA methylation measured in the dilated intima-media portion of the ascending aorta of BAV and TAV patients. Significance of differences between groups is calculated using Mann-Whitney U-test. N=7 BAV and N=7 TAV. CpGs are 1-6. according to Supplementary Table S6. Data is expressed as mean methylation ± standard error of the mean. *. *P*≤0.05; **. *P*≤0.01; ***. *P*≤0.001.

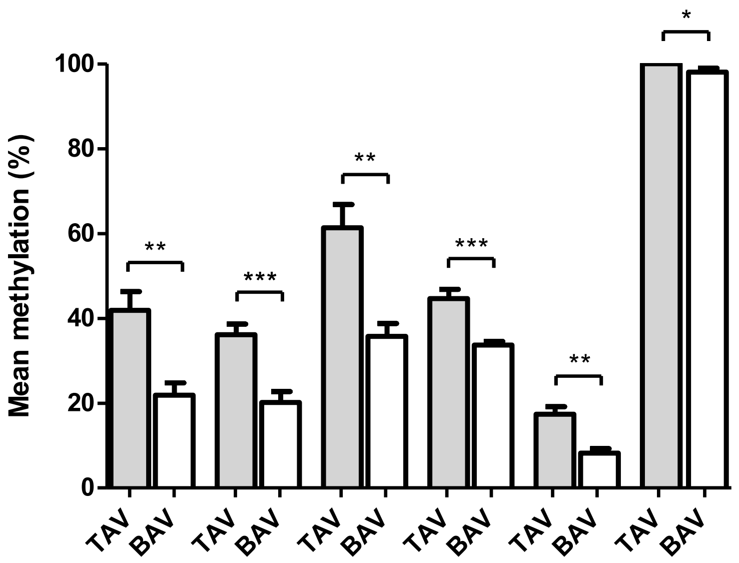

Supplementary Figure S5

DNA methylation of the internal thoracic artery. (a) Principal component analysis of BAV (pink) and TAV (blue) DNA methylation using M-values; (b) mean methylation levels. BAV vs. TAV (Student's t-test). N=14 BAV and N=13 TAV. Data is expressed as mean methylation ± standard deviation.

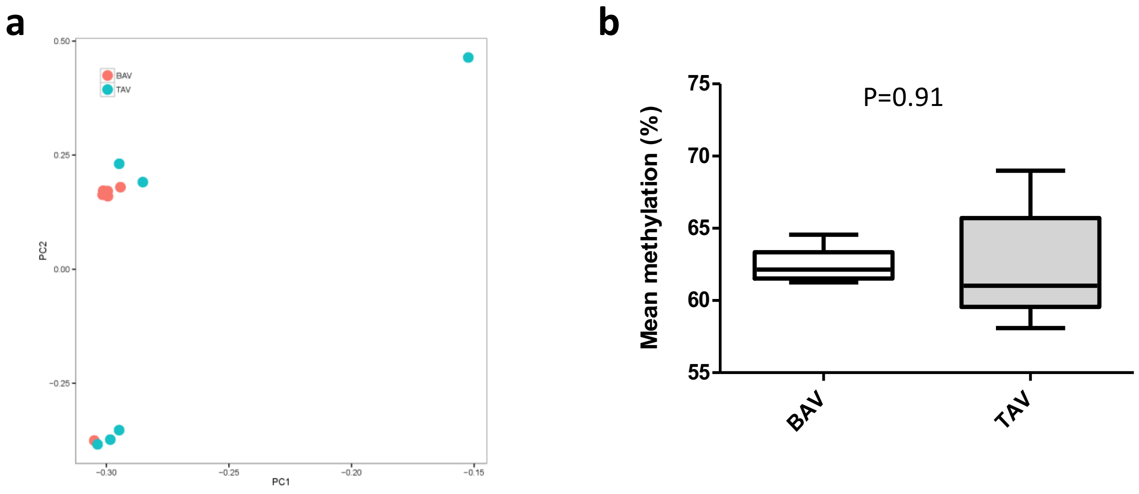

Supplementary Figure S6

Ontology analysis of DMR-genes. dilated BAV vs. TAV aorta. (a) Hypomethylated DMR-genes in BAV-D, Cellular Compartment; (b) Hypomethylated DMR-genes in BAV-D, Biological Processes. N=14 BAV-D and N=13 TAV-D.

a **GO Cellular compartment, Hypomethylated DMR-associated genes in BAV-D**

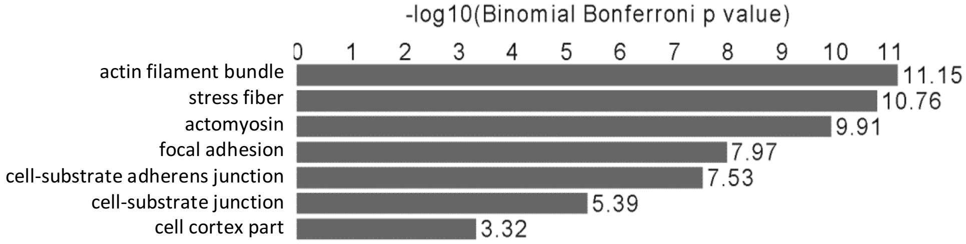

b- **GO Biological process, Hypomethylated DMR-associated genes in BAV-D**

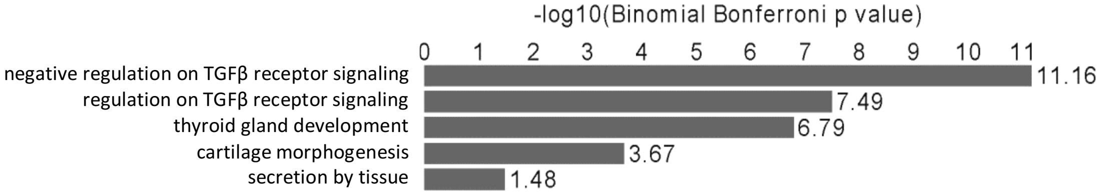

References

1. Jackson V, Petrini J, Caidahl K, Eriksson MJ, Liska J, Eriksson P, Franco-Cereceda A. Bicuspid aortic valve leaflet morphology in relation to aortic root morphology: A study of 300 patients undergoing open-heart surgery. *European journal of cardio-thoracic surgery : official journal of the European Association for Cardio-thoracic Surgery*. 2011;**40**:e118-124
2. Folkersen L, Wagsater D, Paloschi V, Jackson V, Petrini J, Kurtovic S, Maleki S, Eriksson MJ, Caidahl K, Hamsten A, Michel JB, Liska J, Gabrielsen A, Franco-Cereceda A, Eriksson P. Unraveling the divergent gene expression profiles in bicuspid and tricuspid aortic valve patients with thoracic aortic dilatation - the asap study. *Mol Med*. 2011;**17**:1365-1373
3. Edgell CJ, McDonald CC, Graham JB. Permanent cell line expressing human factor viii-related antigen established by hybridization. *Proceedings of the National Academy of Sciences of the United States of America*. 1983;**80**:3734-3737
4. Malashicheva A, Kostina D, Kostina A, Irtyuga O, Voronkina I, Smagina L, Ignatieva E, Gavriliuk N, Uspensky V, Moiseeva O, Vaage J, Kostareva A. Phenotypic and functional changes of endothelial and smooth muscle cells in thoracic aortic aneurysms. *International journal of vascular medicine*. 2016;**2016**:3107879
5. R Development Core Team. R: A language and environment for statistical computing. 2015
6. Du P, Kibbe WA, Lin SM. Lumi: A pipeline for processing illumina microarray. *Bioinformatics*. 2008;**24**:1547-1548
7. Du P, Zhang X, Huang CC, Jafari N, Kibbe WA, Hou L, Lin SM. Comparison of beta-value and m-value methods for quantifying methylation levels by microarray analysis. *BMC bioinformatics*. 2010;**11**:587
8. Lin SM, Du P, Huber W, Kibbe WA. Model-based variance-stabilizing transformation for illumina microarray data. *Nucleic acids research*. 2008;**36**:e11
9. Du P, Kibbe WA, Lin SM. Nuid: A universal naming scheme of oligonucleotides for illumina, affymetrix, and other microarrays. *Biology direct*. 2007;**2**:16
10. Davis S, Du P, Bilke S, Triche JT, Bootwalla M. Methylumi: Handle illumina methylation data. 2015
11. Slieker RC, Bos SD, Goeman JJ, Bovee JV, Talens RP, van der Breggen R, Suchiman HE, Lameijer EW, Putter H, van den Akker EB, Zhang Y, Jukema JW, Slagboom PE, Meulenbelt I, Heijmans BT. Identification and systematic annotation of tissue-specific differentially methylated regions using the illumina 450k array. *Epigenetics & chromatin*. 2013;**6**:26
12. McLean CY, Bristor D, Hiller M, Clarke SL, Schaar BT, Lowe CB, Wenger AM, Bejerano G. Great improves functional interpretation of cis-regulatory regions. *Nature biotechnology*. 2010;**28**:495-501
13. Subramanian A, Tamayo P, Mootha VK, Mukherjee S, Ebert BL, Gillette MA, Paulovich A, Pomeroy SL, Golub TR, Lander ES, Mesirov JP. Gene set enrichment analysis: A knowledge-based approach for interpreting genome-wide expression profiles. *Proceedings of the National Academy of Sciences of the United States of America*. 2005;**102**:15545-15550

Supplementary Table 4. DMRs BAV-ND vs. TAV-ND, and associated genes				
HYPERMETHYLATED in BAV				
**Region**	**DMR start**	**DMR end**	**Chr**	**Gene**
R.26	18958324	18958823	chr1	PAX7 (+1074), TAS1R2 (+227602)
R.27	18959891	18960672	chr1	PAX7 (+2782), TAS1R2 (+225894)
R.28	18963670	18964400	chr1	PAX7 (+6535), TAS1R2 (+222141)
R.29	19051735	19052235	chr1	PAX7 (+94485), TAS1R2 (+134191)
R.39	47695138	47695475	chr1	TAL1 (+136)
R.47	161008644	161008811	chr1	ENSG00000270149 (+6), TSTD1 (+52)
R.58	1895191	1895643	chr2	PXDN (-147139), MYT1L (+439471)
R.64	45157296	45158117	chr2	SIX3 (-11195), CAMKMT (+568604)
R.65	45164720	45165193	chr2	SIX3 (-3945)
R.68	63285491	63285846	chr2	OTX1 (+7732), WDPCP (+530264)
R.69	71123688	71124531	chr2	VAX2 (-3610)
R.76	105273376	105273432	chr2	POU3F3 (-198565)
R.77	105274698	105275613	chr2	POU3F3 (-196813)
R.78	105461870	105462313	chr2	POU3F3 (-9877)
R.79	105473969	105474472	chr2	MRPS9 (-180220), POU3F3 (+2252)
R.80	105483497	105484357	chr2	MRPS9 (-170514), POU3F3 (+11958)
R.81	105484508	105484823	chr2	MRPS9 (-169775), POU3F3 (+12697)
R.85	119607379	119607573	chr2	EN1 (-2222)
R.87	162272687	162273152	chr2	TBR1 (+315)
R.90	171569144	171569282	chr2	SP5 (-2648)
R.91	171671648	171671795	chr2	GAD1 (-1350)
R.92	171676306	171676925	chr2	GORASP2 (-108420), GAD1 (+3544)
R.93	172946193	172947075	chr2	DLX1 (-3320)
R.94	172951553	172952136	chr2	DLX1 (+1891), DLX2 (+15783)
R.95	172953032	172953630	chr2	DLX1 (+3377), DLX2 (+14297)
R.96	172955512	172955930	chr2	DLX1 (+5767), DLX2 (+11907)
R.97	172965650	172966428	chr2	DLX2 (+1589), DLX1 (+16085)
R.98	172973195	172974138	chr2	ITGA6 (-318850), DLX2 (-6039)
R.100	176949630	176950007	chr2	HOXD10 (-31488), HOXD11 (-22195), HOXD12 (-14639), HOXD13 (-7800), EVX2 (-1178)
R.101	176968052	176968920	chr2	HOXD10 (-12821), HOXD11 (-3528)
R.102	176973835	176974060	chr2	HOXD10 (-7359), HOXD11 (+1934)
R.103	176978893	176979788	chr2	HOXD10 (-1966)
R.104	176993643	176994142	chr2	HOXD8 (-575)
R.105	177012117	177013071	chr2	HOXD4 (-3356), HOXD3 (-2528)
R.106	177014555	177014685	chr2	HOXD4 (-1330), HOXD3 (-502)
R.107	177014959	177015125	chr2	HOXD4 (-908), HOXD3 (-80)
R.108	177022285	177023183	chr2	HOXD1 (-30573), HOXD4 (+6784)
R.109	177023667	177024502	chr2	HOXD1 (-29222), HOXD4 (+8135)
R.110	177036809	177037364	chr2	HOXD1 (-16220), HOXD4 (+21137)
R.111	200326591	200326725	chr2	SATB2 (-3839)
R.113	215675429	215675637	chr2	BARD1 (-1105)
R.124	242802009	242802099	chr2	PDCD1 (-994)
R.128	13590419	13590439	chr3	FBLN2 (-202)
R.131	27764816	27765484	chr3	EOMES (-1161)
R.152	11652441	11652505	chr4	HS3ST1 (-221084)
R.154	41875470	41875784	chr4	PHOX2B (-124640), TMEM33 (-61510)

R.156	85401758	85402497	chr4	NKX6-1 (+17475), AGPAT9 (+944867)
R.157	85414147	85414486	chr4	NKX6-1 (+5286), AGPAT9 (+957056)
R.164	186732124	186733060	chr4	PDLIM3 (-275930), SORBS2 (+145214)
R.169	1800542	1800782	chr5	NDUFS6 (-852), MRPL36 (-653)
R.170	2094296	2094524	chr5	IRX4 (-207078), IRX2 (+657366)
R.178	59064378	59064517	chr5	PDE4D (+125177)
R.180	76936361	76936636	chr5	OTP (-986)
R.182	92904875	92905860	chr5	NR2F1 (-13675)
R.184	92929319	92930052	chr5	NR2F1 (+10643), POU5F2 (+147657)
R.187	134880436	134880895	chr5	NEUROG1 (-9027), CXCL14 (+34303)
R.190	143978290	143978420	chr5	KCTD16 (+393515)
R.206	10417385	10418121	chr6	TFAP2A (-2283)
R.217	31325455	31326324	chr6	HLA-B (-926)
R.221	31691035	31691227	chr6	LY6G6C (-1509), C6orf25 (-30)
R.240	32805398	32805759	chr6	TAP2 (+925)
R.252	99273568	99273679	chr6	POU3F2 (-8956)
R.253	106545984	106546747	chr6	PRDM1 (+12171), ATG5 (+227300)
R.255	117923860	117923992	chr6	GOPC (-399)
R.258	137812735	137813648	chr6	IFNGR1 (-272606), OLIG3 (+2339)
R.275	20816695	20817530	chr7	SP8 (+9392), ABCB5 (+162283)
R.276	20827117	20827825	chr7	SP8 (-966)
R.277	20838523	20838581	chr7	SP4 (-629100), SP8 (-12047)
R.278	22767384	22767571	chr7	IL6 (+1975), TOMM7 (+94992)
R.281	26897253	26897612	chr7	SKAP2 (+6929), SNX10 (+565890)
R.282	27134109	27134369	chr7	SKAP2 (-229877), HOXA1 (+1354)
R.290	27187553	27187560	chr7	HOXA5 (-4270), HOXA6 (-164)
R.291	27224954	27225078	chr7	HOXA11 (-181)
R.292	27229028	27229163	chr7	HOXA11 (-4261)
R.293	27231204	27232150	chr7	HOXA11 (-6842), HOXA13 (+8048)
R.294	27252541	27253143	chr7	EVX1 (-29322), HOXA13 (-13117)
R.295	27260102	27260285	chr7	EVX1 (-21970), HOXA13 (-20469)
R.302	96627253	96627634	chr7	SHFM1 (-288241), DLX6 (-7416)
R.318	1651101	1651197	chr8	CLN8 (-60779), DLGAP2 (+201617)
R.322	9764602	9765601	chr8	MSRA (-146676), TNKS (+351678)
R.324	24858570	24859367	chr8	DOCK5 (-183411), NEFM (+88444)
R.335	972520	973198	chr9	DMRT3 (-4105)
R.336	974533	975059	chr9	DMRT3 (-2168)
R.354	18947857	18948058	chr10	ARL5B (-376)
R.355	21783091	21783445	chr10	NEBL (-596737), SKIDA1 (+31343)
R.356	22541995	22542145	chr10	DNAJC1 (-249416), COMMD3 (-62833)
R.361	44788627	44789589	chr10	ZNF32 (-644956), CXCL12 (+91389)
R.366	77035993	77036584	chr10	COMTD1 (-40520), ZNF503 (+125375)
R.379	134498830	134498960	chr10	NKX6-2 (+100661), INPP5A (+147571)
R.400	14994130	14994198	chr11	CALCA (-264)
R.401	14994381	14994561	chr11	CALCA (-571)
R.402	14996355	14996490	chr11	CALCA (-2523)
R.403	34460318	34460351	chr11	CAT (-137)
R.409	62693039	62693396	chr11	CHRM1 (-4065)
R.413	68147880	68148079	chr11	PPP6R3 (-80258), LRP5 (+67903)
R.414	68621969	68622263	chr11	CPT1A (-12732), MRPL21 (+49172)

R.432	125496181	125496752	chr11	CHEK1 (+231)
R.436	6485537	6486123	chr12	SCNN1A (-1440)
R.439	7260877	7261710	chr12	C1RL (+575)
R.440	7341252	7341580	chr12	PEX5 (-867)
R.448	95867190	95867348	chr12	METAP2 (-458)
R.463	130529394	130530121	chr12	TMEM132D (-141547), FZD10 (-117246)
R.471	28544760	28545214	chr13	CDX2 (+289)
R.474	45149661	45149706	chr13	TSC22D1 (+1017), SERP2 (+201706)
R.482	112714024	112714769	chr13	SOX1 (-7516), TEX29 (+741382)
R.491	38065152	38065303	chr14	FOXA1 (-989)
R.492	38725536	38725750	chr14	CLEC14A (-69)
R.493	54423807	54423845	chr14	BMP4 (-297)
R.507	102554826	102554977	chr14	HSP90AA1 (+51121), DYNC1H1 (+124037)
R.521	89952135	89952299	chr15	POLG (-74139), RHCG (+87627)
R.524	96868857	96868989	chr15	NR2F2 (-5023)
R.525	96896940	96897190	chr15	NR2F2 (+23119)
R.527	333104	333155	chr16	PDIA2 (-22)
R.543	54965895	54966471	chr16	IRX6 (-391489), IRX5 (+1409)
R.544	56677634	56677855	chr16	MT1B (-8066), MT1A (+5167)
R.545	56703257	56703551	chr16	MT1G (-1427), MT1H (-322)
R.586	38574364	38574566	chr17	TOP2A (-263)
R.590	41832753	41833161	chr17	MEOX1 (-93635), SOST (+3199)
R.592	43863000	43863356	chr17	SPPL2C (-59078), CRHR1 (+1267)
R.593	59488116	59488443	chr17	TBX4 (-45527), TBX2 (+11023)
R.611	80187886	80188598	chr17	SLC16A3 (-3321)
R.612	80189645	80189973	chr17	SLC16A3 (-1754)
R.613	80190054	80190154	chr17	SLC16A3 (-1459)
R.630	10624868	10625429	chr19	KEAP1 (-10732), S1PR5 (+3458)
R.632	10958952	10959101	chr19	C19orf38 (-51)
R.636	42636811	42636978	chr19	POU2F2 (-316)
R.638	44285333	44285594	chr19	KCNN4 (-55)
R.642	45905621	45906156	chr19	CD3EAP (-3578)
R.655	4202151	4202764	chr20	ADRA1D (+27263), SMOX (+73032)
R.658	30309466	30309717	chr20	BCL2L1 (+1109), COX4I2 (+83901)
R.669	46128908	46129392	chr21	TSPEAR (+2345), KRTAP10-12 (+12063)
R.681	50523761	50524374	chr22	MOV10L1 (-4367), MLC1 (+263)
**HYPOMETHYLATED in BAV**				
**Region**	**DMR start**	**DMR end**	**Chr**	**Gene**
R.5	2254199	2254582	chr1	RER1 (-68881), SKI (+94257)
R.6	2792637	2792744	chr1	MMEL1 (-228262), ACTRT2 (-145355)
R.7	3010896	3011304	chr1	ARHGEF16 (-359890), PRDM16 (+25325)
R.8	3077835	3078524	chr1	ARHGEF16 (-292810), PRDM16 (+92405)
R.9	3104999	3105252	chr1	ARHGEF16 (-265864), PRDM16 (+119351)
R.10	3133763	3134162	chr1	ARHGEF16 (-237027), PRDM16 (+148188)
R.11	3141992	3142925	chr1	ARHGEF16 (-228531), PRDM16 (+156684)
R.12	3157287	3157480	chr1	ARHGEF16 (-213606), PRDM16 (+171609)
R.13	3228966	3229579	chr1	ARHGEF16 (-141717), PRDM16 (+243498)
R.19	6580308	6581295	chr1	PLEKHG5 (-23646), NOL9 (+33793)
R.20	12703864	12704007	chr1	AADACL4 (-630)
R.22	16346120	16346434	chr1	CLCNKA (-2270), HSPB7 (-992)

R.23	17746286	17746491	chr1	RCC2 (+19831), PADI4 (+111697)
R.30	19614429	19615080	chr1	AKR7A3 (+989)
R.31	26394083	26394211	chr1	TRIM63 (-23)
R.33	36026194	36026611	chr1	KIAA0319L (-3389), NCDN (+2994)
R.37	45083079	45083207	chr1	TMEM53 (+57084), RNF220 (+212277)
R.41	86048479	86048585	chr1	CYR61 (+2088), ZNHIT6 (+125569)
R.43	153519020	153519850	chr1	S100A4 (-1074)
R.44	153520801	153521758	chr1	S100A4 (-2919), S100A3 (+568)
R.45	154929762	154930232	chr1	PBXIP1 (-1417)
R.46	160084833	160085581	chr1	ATP1A2 (-356)
R.48	172113506	172114068	chr1	PIGC (+299439), DNM3 (+303166)
R.49	201708888	201709390	chr1	IPO9 (-89130), NAV1 (+91762)
R.51	230272306	230272656	chr1	GALNT2 (+69525), PGBD5 (+240910)
R.52	234667087	234667500	chr1	TARBP1 (-52445), IRF2BP2 (+77977)
R.53	193256	193509	chr2	FAM110C (-146998), SH3YL1 (+70682)
R.56	1796076	1796228	chr2	PXDN (-47874), MYT1L (+538736)
R.57	1827732	1828377	chr2	PXDN (-79777), MYT1L (+506833)
R.59	2773427	2773984	chr2	TRAPPC12 (-609751), MYT1L (-438818)
R.61	19553422	19554237	chr2	NT5C1B-RDH14 (-783002), OSR1 (+2847)
R.62	23746926	23747197	chr2	ATAD2B (+402922)
R.71	72079413	72079609	chr2	CYP26B1 (+295656), DYSF (+385679)
R.72	74119781	74120274	chr2	ACTG2 (-107)
R.74	100722584	100723240	chr2	AFF3 (-867)
R.83	109934728	109934860	chr2	SH3RF3 (+188797), SEPT10 (+436989)
R.112	208689522	208689903	chr2	FZD5 (-55426), PLEKHM3 (+200571)
R.115	219696472	219697159	chr2	PRKAG3 (-7)
R.116	223916502	223916687	chr2	KCNE4 (-267)
R.117	223916861	223917590	chr2	KCNE4 (+364)
R.118	236773924	236774164	chr2	GBX2 (+302968), AGAP1 (+371293)
R.119	240061601	240062353	chr2	HDAC4 (+260666), TWIST2 (+305304)
R.122	241936844	241937034	chr2	SNED1 (-1316)
R.126	11178652	11178745	chr3	HRH1 (-115686), SLC6A1 (+144289)
R.127	13060741	13060974	chr3	IQSEC1 (-51690), NUP210 (+400951)
R.129	14443428	14443473	chr3	SLC6A6 (-625)
R.130	16215248	16216094	chr3	GALNT15 (-485)
R.132	45077369	45077765	chr3	CLEC3B (+9892), CDCP1 (+110347)
R.134	50306107	50306456	chr3	LSMEM2 (-10176), GNAI2 (+32898)
R.135	123339334	123339568	chr3	PTPLB (-35419), MYLK (+263698)
R.139	184209224	184209412	chr3	EPHB3 (-70254), CHRD (+111457)
R.140	187896277	187897193	chr3	BCL6 (-433220), LPP (-33986)
R.141	187929592	187930530	chr3	LPP (-660)
R.145	1148456	1148569	chr4	RNF212 (-41199), SPON2 (+18148)
R.146	1188421	1189037	chr4	SPON2 (-22068), CTBP1 (+54196)
R.147	3773933	3773996	chr4	ADRA2C (+5840), OTOP1 (+454651)
R.148	7287492	7287737	chr4	SORCS2 (+93350), PSAPL1 (+149085)
R.149	7338730	7338837	chr4	PSAPL1 (+97916), SORCS2 (+144519)
R.150	8395941	8396163	chr4	ACOX3 (+46386), HTRA3 (+124560)
R.151	8702279	8702475	chr4	CPZ (+107990), HMX1 (+171166)
R.153	13543985	13544632	chr4	RAB28 (-58320), NKX3-2 (+2365)
R.158	114213914	114214093	chr4	ANK2 (+243172), CAMK2D (+468220)

R.161	166130172	166131163	chr4	KLHL2 (-377)
R.163	186559938	186560403	chr4	PDLIM3 (-103509), SORBS2 (+317635)
R.167	1553051	1553224	chr5	LPCAT1 (-29046), MRPL36 (+246871)
R.171	2169371	2170036	chr5	IRX4 (-282372), IRX2 (+582072)
R.172	3181925	3182108	chr5	IRX1 (-414151), C5orf38 (+429755)
R.174	34043333	34043481	chr5	C1QTNF3 (+530)
R.176	50260045	50260240	chr5	ISL1 (-418778), PARP8 (+297371)
R.177	50695224	50695720	chr5	ISL1 (+16551)
R.186	122423190	122424091	chr5	PRDM6 (-1175)
R.188	139056667	139057496	chr5	PSD2 (-118324), CXXC5 (+29205)
R.189	141191429	141191557	chr5	ARAP3 (-129705), PCDH1 (+66482)
R.191	149980486	149980674	chr5	SYNPO (-62)
R.193	170877840	170878209	chr5	C5orf50 (-334851), FGF18 (+31365)
R.194	175108269	175108315	chr5	CPLX2 (-115021), HRH2 (+23259)
R.197	1604134	1604212	chr6	FOXC1 (-6508), FOXF2 (+214104)
R.198	1607507	1608500	chr6	FOXC1 (-2677)
R.199	1618814	1619162	chr6	FOXC1 (+8307), GMDS (+626938)
R.200	1620137	1621093	chr6	FOXC1 (+9934), GMDS (+625311)
R.201	2382649	2382777	chr6	GMDS (-136787), MYLK4 (+368487)
R.203	3724407	3724690	chr6	SLC22A23 (-267756), PXDC1 (+27711)
R.204	3737830	3737956	chr6	SLC22A23 (-281100), PXDC1 (+14367)
R.207	25232780	25232874	chr6	FAM65B (-321632), LRRC16A (-46829)
R.209	25732239	25732956	chr6	SLC17A4 (-22329), HIST1H2BA (+5461)
R.211	30095269	30095281	chr6	TRIM31 (-14392), TRIM40 (-9512)
R.223	31734471	31734580	chr6	VWA7 (+10545), MSH5 (+26728)
R.229	32026299	32026610	chr6	TNXB (-12550), ATF6B (+69562)
R.231	32038747	32038958	chr6	TNXB (-24948), ATF6B (+57164)
R.233	32044104	32044404	chr6	TNXB (-30349), ATF6B (+51763)
R.235	32120773	32120826	chr6	PPT2-EGFL8 (-1199), PRRT1 (-1071), PPT2 (-500)
R.237	32137141	32137188	chr6	EGFL8 (+4783), AGPAT1 (+7692)
R.249	36645500	36645648	chr6	CDKN1A (-913)
R.251	88031886	88032061	chr6	SMIM8 (-332)
R.254	116381609	116382179	chr6	FRK (+27)
R.259	152702365	152702444	chr6	SYNE1 (+256129), ESR1 (+690774)
R.260	158404061	158404121	chr6	SYNJ2 (+1172), SERAC1 (+185211)
R.261	158464296	158464852	chr6	SYNJ2 (+61655), SERAC1 (+124728)
R.262	160023581	160023689	chr6	SOD2 (+90725), FNDC1 (+433206)
R.263	553693	553810	chr7	PDGFA (+5279), FAM20C (+360783)
R.264	597568	597596	chr7	PDGFA (-38551), PRKAR1B (+155262)
R.265	640338	641028	chr7	PDGFA (-81652), PRKAR1B (+112161)
R.266	1135747	1136023	chr7	GPER (+9442), ZFAND2A (+63929)
R.268	1686764	1687244	chr7	TMEM184A (-90938), ELFN1 (-40751)
R.271	2678208	2678263	chr7	AMZ1 (-40920), TTYH3 (+6651)
R.273	4065931	4066127	chr7	FOXK1 (-655911), SDK1 (+724949)
R.284	27150262	27150598	chr7	HOXA2 (-8000), HOXA3 (+8784)
R.285	27152583	27153580	chr7	HOXA2 (-10652), HOXA3 (+6132)
R.286	27154264	27154911	chr7	HOXA2 (-12158), HOXA3 (+4626)
R.287	27160520	27160960	chr7	HOXA3 (-1526)
R.296	41745737	41746034	chr7	INHBA (-3180)
R.299	73389575	73389660	chr7	ELN (-52501), WBSCR28 (+114129)

R.300	75596021	75596055	chr7	POR (+51641), STYXL1 (+81283)
R.301	82073751	82074180	chr7	CACNA2D1 (-852)
R.304	98990837	98991418	chr7	BUD31 (-15136), ARPC1B (+18775)
R.305	101768610	101768874	chr7	SH2B2 (-159663), CUX1 (+309451)
R.306	114561988	114562063	chr7	MDFIC (-183)
R.307	135433715	135433790	chr7	FAM180A (-159)
R.311	151433020	151433326	chr7	RHEB (-216163), PRKAG2 (+141037)
R.313	158885581	158886120	chr7	VIPR2 (+51798), WDR60 (+236582)
R.315	1365049	1365906	chr8	TDRP (-869697), DLGAP2 (-84054)
R.316	1404023	1404347	chr8	TDRP (-908404), DLGAP2 (-45347)
R.320	1906312	1906498	chr8	MYOM2 (-86750), ARHGEF10 (+134263)
R.323	23559839	23560319	chr8	NKX3-1 (-19639), NKX2-6 (+4032)
R.325	37005558	37005619	chr8	ZNF703 (-547680), KCNU1 (+363697)
R.326	52722140	52722185	chr8	PXDNL (-158)
R.327	61834943	61835175	chr8	CLVS1 (-365455), CHD7 (+243722)
R.328	141057427	141057827	chr8	C8orf17 (+114211), TRAPPC9 (+411051)
R.331	145033310	145033622	chr8	PLEC (-8422), PARP10 (+27156)
R.332	145047469	145048137	chr8	PLEC (-22759), PARP10 (+12819)
R.334	145728501	145729106	chr8	GPT (-661)
R.338	101568975	101569334	chr9	GALNT12 (-826)
R.340	129433708	129433969	chr9	ZBTB43 (-133455), LMX1B (+57117)
R.341	134699146	134699710	chr9	RAPGEF1 (-114199), MED27 (+255867)
R.345	384506	384652	chr10	ZMYND11 (+204155), DIP2C (+351027)
R.346	711556	712442	chr10	DIP2C (+23607), ZMYND11 (+531575)
R.347	729479	729956	chr10	DIP2C (+5888), ZMYND11 (+549294)
R.349	1558762	1559249	chr10	IDI1 (-463896), ADARB2 (+220664)
R.350	3168685	3169516	chr10	PITRM1 (+45902), PFKP (+59389)
R.351	3789537	3789974	chr10	PITRM1 (-574753), KLF6 (+37711)
R.353	18689378	18689471	chr10	NSUN6 (+251126), CACNB2 (+259819)
R.357	22843607	22843938	chr10	PIP4K2A (+159711), SPAG6 (+209374)
R.358	29811530	29811659	chr10	SVIL (+213135), LYZL1 (+233605)
R.359	30316933	30317872	chr10	SVIL (-292673), KIAA1462 (+31050)
R.360	31107995	31108190	chr10	LYZL2 (-189402), ZNF438 (+212773)
R.362	50506875	50506896	chr10	C10orf128 (-110482), DRGX (+93021)
R.363	63809073	63809170	chr10	ARID5B (+148063), RTKN2 (+219344)
R.365	75631588	75632066	chr10	NDST2 (-60485), CAMK2G (+2516)
R.367	88427400	88428147	chr10	LDB3 (-432)
R.368	88428228	88428295	chr10	LDB3 (+56)
R.369	97175272	97175479	chr10	PDLIM1 (-124595), SORBS1 (+145759)
R.370	102893925	102894148	chr10	TLX1 (+4780), LBX1 (+95514)
R.371	102899285	102899983	chr10	TLX1 (+10377), LBX1 (+89917)
R.372	126278272	126278502	chr10	LHPP (+127983), FAM53B (+154232)
R.373	126703991	126704259	chr10	CTBP2 (+12334), ZRANB1 (+73433)
R.374	126782326	126782397	chr10	C10orf137 (-625783), CTBP2 (-65903)
R.377	133775416	133775551	chr10	BNIP3 (+19951), PPP2R2D (+27529)
R.378	133929429	133929519	chr10	DPYSL4 (-70930), JAKMIP3 (+11299)
R.380	134801360	134801775	chr10	NKX6-2 (-202012), GPR123 (-99841)
R.385	1507119	1507321	chr11	MOB2 (+756)
R.388	2162363	2162536	chr11	IGF2 (+18)
R.390	2295090	2295191	chr11	ASCL2 (-2959)

R.392	2890670	2890710	chr11	CDKN1C (+16421), KCNQ1 (+424469)
R.394	3167407	3167827	chr11	CARS (-88950), OSBPL5 (+18967)
R.397	7597402	7597983	chr11	PPFIBP2 (+62692), CYB5R2 (+97746)
R.399	10715221	10715445	chr11	MRVI1 (-34)
R.410	64703312	64703510	chr11	GPHA2 (-51)
R.411	64808081	64808285	chr11	SAC3D1 (-193)
R.415	68779553	68779826	chr11	MRGPRD (-31235), MRGPRF (+1187)
R.416	68781976	68782211	chr11	MRGPRF (-1217)
R.417	68924746	68925191	chr11	CCND1 (-530886), TPCN2 (+108604)
R.418	68970629	68971452	chr11	CCND1 (-484814), TPCN2 (+154676)
R.422	72533295	72533664	chr11	ATG16L2 (+8127), FCHSD2 (+319826)
R.423	77774867	77775044	chr11	THRSP (+49)
R.424	85430070	85430657	chr11	CCDC89 (-33044), SYTL2 (+7147)
R.425	93754288	93754390	chr11	HEPHL1 (-188)
R.428	114127924	114128517	chr11	NNMT (-332)
R.430	118781778	118781978	chr11	BCL9L (-265)
R.431	120038521	120038992	chr11	POU2F3 (-68592), TRIM29 (-29596)
R.433	128559244	128559701	chr11	FLI1 (-4417)
R.435	2113119	2113435	chr12	DCP1B (+400)
R.441	52437299	52437571	chr12	C12orf44 (-26320), NR4A1 (+20819)
R.443	53448085	53448180	chr12	IGFBP6 (-43096), TENC1 (+5400)
R.445	54785003	54785055	chr12	ZNF385A (+53)
R.446	56113808	56114269	chr12	RDH5 (-143)
R.449	107974557	107974897	chr12	PWP1 (-104782), BTBD11 (+262537)
R.452	111620199	111620313	chr12	CUX2 (+148428), FAM109A (+186669)
R.455	116756805	116756948	chr12	MAP1LC3B2 (-240309), MED13L (-41734)
R.456	120241760	120242090	chr12	CIT (+73170), PRKAB1 (+136367)
R.457	121416315	121416796	chr12	HNF1A (+210)
R.461	124773103	124773668	chr12	NCOR2 (+206412), ZNF664 (+315716)
R.462	125242977	125243251	chr12	NCOR2 (-263316), SCARB1 (+105279)
R.466	132920348	132920884	chr12	GALNT9 (-230043), MUC8 (+130110)
R.472	36050844	36050930	chr13	MAB21L1 (-55)
R.475	46426202	46426263	chr13	SIAH3 (-362)
R.476	102104848	102104991	chr13	ITGBL1 (-46)
R.478	110521956	110522265	chr13	IRS2 (-83196), COL4A1 (+437385)
R.479	110874267	110874326	chr13	IRS2 (-435382), COL4A1 (+85199)
R.480	110885345	110885926	chr13	IRS2 (-446721), COL4A1 (+73860)
R.481	111839104	111839335	chr13	TEX29 (-133795), ARHGEF7 (+71596)
R.486	114797047	114797383	chr13	GAS6 (-230175), RASA3 (+100871)
R.489	24027960	24028894	chr14	THTPA (+3196), AP1G2 (+8852)
R.494	65172103	65172436	chr14	PLEKHG3 (+1116), SPTB (+117596)
R.495	75530906	75531782	chr14	ZC2HC1C (-4639), ACYP1 (-592)
R.496	76445988	76446681	chr14	TGFB3 (+1199), TTLL5 (+318714)
R.497	90042201	90042222	chr14	FOXN3 (+43262), TTC8 (+751206)
R.498	93419016	93419554	chr14	CHGA (+29860), ITPK1 (+162863)
R.499	93698053	93698416	chr14	UNC79 (-101330), UBR7 (+24834)
R.501	94462309	94462337	chr14	OTUB2 (-30352), ASB2 (-19186)
R.502	95693784	95693881	chr14	DICER1 (-69486), CLMN (+92410)
R.503	98444151	98444513	chr14	NONE
R.506	101830869	101831160	chr14	DIO3 (-196673), ENSG00000269375 (+471750)

R.509	103416122	103416389	chr14	AMN (+27263), CDC42BPB (+107543)
R.512	31516111	31516481	chr15	KLF13 (-102762), TRPM1 (-62820)
R.514	67228722	67228986	chr15	SMAD3 (-129329), SMAD6 (+234288)
R.515	67458370	67458522	chr15	AAGAB (+88628), SMAD3 (+100263)
R.516	69841201	69841259	chr15	RPLP1 (+96107), TLE3 (+549285)
R.523	90190637	90191154	chr15	KIF7 (+7786), TICRR (+72183)
R.526	101728653	101729391	chr15	CHSY1 (+63115), LRRK1 (+269602)
R.529	1033074	1034059	chr16	SSTR5 (-95214), SOX8 (+1759)
R.531	1583883	1584050	chr16	TMEM204 (+393)
R.535	11348611	11348684	chr16	SOCS1 (+1388), CIITA (+377593)
R.541	46823935	46824143	chr16	MYLK3 (-41818), C16orf87 (+41284)
R.542	49732224	49732567	chr16	CBLN1 (-416654), ZNF423 (+124254)
R.546	57405979	57406074	chr16	CX3CL1 (-343)
R.547	67977865	67978182	chr16	LCAT (+10)
R.549	72981203	72981599	chr16	PMFBP1 (-775247), ZFHX3 (+100873)
R.550	73125573	73126120	chr16	ZFHX3 (-43573)
R.551	73126608	73127462	chr16	ZFHX3 (-44761)
R.552	73206310	73206624	chr16	ZFHX3 (-124193)
R.554	85320518	85320882	chr16	GSE1 (-326122), KIAA0513 (+223882)
R.556	85479264	85479803	chr16	GSE1 (-167288), KIAA0513 (+382716)
R.557	85619974	85620872	chr16	GSE1 (-26399), KIAA0513 (+523605)
R.559	86598792	86599189	chr16	FOXC2 (-1866)
R.560	86609656	86610434	chr16	FOXL1 (-2070)
R.561	86611690	86612451	chr16	FOXL1 (-44)
R.562	86715152	86715554	chr16	FOXL1 (+103238), FBXO31 (+702010)
R.566	88976160	88976453	chr16	PABPN1L (-43281), CBFA2T3 (+67305)
R.568	800084	800717	chr17	GLOD4 (-114820), NXN (+82609)
R.577	7491548	7492524	chr17	SOX15 (+1452), MPDU1 (+5071)
R.580	8736136	8736293	chr17	MFSD6L (-33548), PIK3R6 (+34779)
R.585	38084377	38084459	chr17	ORMDL3 (-1324)
R.587	38708399	38708638	chr17	TNS4 (-50670), CCR7 (+13205)
R.588	41003272	41003399	chr17	AOC3 (+135)
R.591	42994008	42994269	chr17	GFAP (-1277)
R.594	60886038	60886284	chr17	MARCH10 (-466)
R.598	75373327	75373457	chr17	TNRC6C (-627745), SEPT9 (+95900)
R.600	76719591	76719640	chr17	DNAH17 (-146140), CYTH1 (+58760)
R.605	78755379	78755841	chr17	CHMP6 (-210031), RPTOR (+236542)
R.607	79138873	79139112	chr17	AATK (+824)
R.610	79377665	79378207	chr17	ENSG00000171282 (+4396), ACTG1 (+101871)
R.616	8367425	8367691	chr18	RAB12 (-241885), PTPRM (+800778)
R.617	19756877	19757468	chr18	GATA6 (+7769), CTAGE1 (+240705)
R.621	1356269	1356304	chr19	NDUFS7 (-27343), EFNA2 (+70134)
R.623	2494236	2494595	chr19	GADD45B (+18291), GNG7 (+208291)
R.624	2525231	2525634	chr19	GADD45B (+49308), GNG7 (+177274)
R.627	7580209	7580275	chr19	ZNF358 (-762)
R.631	10928211	10928696	chr19	TMED1 (+18512), DNM2 (+99647)
R.633	13113455	13113893	chr19	NFIX (+7252), LYL1 (+100007)
R.635	35629356	35629603	chr19	FXYD7 (-4674), LGI4 (-3376), FXYD1 (-1446)
R.639	45260935	45261808	chr19	CBLC (-19754), BCL3 (+9568)
R.641	45737610	45737792	chr19	EXOC3L2 (-232)

R.647	49223814	49224454	chr19	MAMSTR (-1156)
R.654	3687817	3688314	chr20	SIGLEC1 (-291)
R.656	17674195	17674250	chr20	BANF2 (-94)
R.657	25039707	25039812	chr20	ACSS1 (-942)
R.659	30406997	30407388	chr20	MYLK2 (+82)
R.660	30433409	30433673	chr20	FOXS1 (-121)
R.661	30605656	30605916	chr20	HCK (-34278), CCM2L (+7533)
R.664	56287004	56287532	chr20	PMEPA1 (-2310)
R.666	33749529	33749986	chr21	URB1 (+15577), MRAP (+85634)
R.670	17680531	17680706	chr22	CECR5 (-40452), CECR1 (+22260)
R.672	24402088	24402725	chr22	GSTT1 (-18123), CABIN1 (-5461)
R.674	35695157	35695301	chr22	TOM1 (-568)
R.676	41956861	41957443	chr22	CSDC2 (+385)
R.678	43166139	43166347	chr22	A4GALT (-75251), ARFGAP3 (+87165)
R.680	46458783	46459327	chr22	PPARA (-87444), WNT7B (-86046)

Supplementary Table 6a. Hypomethylated DMRs under disturbed flow, and associated genes				
**Region**	**DMR start**	**DMR end**	**Chr**	**Gene**
R.1	762232	762882	chr1	OR4F16 (-140504), SAMD11 (-98561)
R.2	894056	894207	chr1	KLHL17 (-1835), NOC2L (+538)
R.3	896226	896316	chr1	NOC2L (-1601), KLHL17 (+304)
R.4	934197	934662	chr1	HES4 (+1122), PLEKHN1 (+32553)
R.5	1208426	1208709	chr1	UBE2J2 (+645)
R.6	1245040	1245184	chr1	ACAP3 (-1714), PUSL1 (+1165)
R.7	1259676	1259698	chr1	GLTPD1 (-460), CPSF3L (+302)
R.8	1294006	1294140	chr1	MXRA8 (-158)
R.9	1335424	1335601	chr1	CCNL2 (-821)
R.10	1710229	1710265	chr1	NADK (-338)
R.11	2121349	2121521	chr1	C1orf86 (+4778), PRKCZ (+139526)
R.12	3387913	3388196	chr1	ARHGEF16 (+17065), MEGF6 (+140004)
R.13	3540077	3540901	chr1	TPRG1L (-1077)
R.14	3568669	3569046	chr1	WRAP73 (-2221), TP73 (-226)
R.15	3689178	3689519	chr1	SMIM1 (-3)
R.16	6639738	6639762	chr1	ZBTB48 (-358)
R.17	7764642	7765369	chr1	VAMP3 (-66323), CAMTA1 (+919622)
R.18	9747263	9748107	chr1	PIK3CD (+35882), CLSTN1 (+136899)
R.19	11714873	11715197	chr1	FBXO2 (-296), FBXO44 (+121)
R.20	12226928	12227131	chr1	TNFRSF1B (-30)
R.21	12655992	12656315	chr1	DHRS3 (+21583), TNFRSF1B (+429094)
R.22	14026584	14026817	chr1	PRDM2 (-4649)
R.23	19971172	19971709	chr1	HTR6 (-20339), NBL1 (+1715)
R.24	24069514	24069711	chr1	TCEB3 (-32)
R.25	25558575	25558918	chr1	SYF2 (+237)
R.26	25566331	25567240	chr1	RHD (-32098), SYF2 (-7802)
R.27	25943862	25943891	chr1	MAN1C1 (-82)
R.28	26147252	26147299	chr1	SEPN1 (+20609), AUNIP (+38627)
R.29	26496421	26497107	chr1	ZNF593 (+402)
R.30	27226971	27227008	chr1	GPATCH3 (-33)
R.31	27339314	27339435	chr1	TRNP1 (+19177), SLC9A1 (+142026)
R.32	27339612	27339786	chr1	TRNP1 (+19501), SLC9A1 (+141702)
R.33	27986522	27986807	chr1	FGR (-24877), IFI6 (+12024)
R.34	29450643	29450836	chr1	TMEM200B (-1727)
R.35	32687567	32687590	chr1	EIF3I (-392), TMEM234 (+347)
R.36	33351946	33352269	chr1	HPCA (+72)
R.37	34628784	34629098	chr1	CSMD2 (+2502), HMGB4 (+302840)
R.38	34630864	34630944	chr1	CSMD2 (+539)
R.39	36690068	36690560	chr1	THRAP3 (+297)
R.40	38471474	38471572	chr1	FHL3 (-346)
R.41	38512250	38512595	chr1	POU3F1 (+27)
R.42	39249425	39249604	chr1	POU3F1 (-737065), RRAGC (+75980)
R.43	40105667	40105706	chr1	HEYL (-70)
R.44	40157086	40157125	chr1	HPCAL4 (+255)
R.45	40254529	40254859	chr1	BMP8B (-161)

R.46	40562564	40562678	chr1	PPT1 (+754)
R.47	40915518	40915896	chr1	ZFP69B (-72)
R.48	40942785	40943211	chr1	ZFP69 (+111)
R.49	42928798	42929392	chr1	PPIH (-195001), PPCS (+6865)
R.50	43281678	43282382	chr1	ERMAP (-772), CCDC23 (+893)
R.52	43613433	43613494	chr1	SLC2A1 (-188963), EBNA1BP2 (+24777)
R.53	44440160	44440242	chr1	ATP6V0B (-119)
R.54	44497012	44497352	chr1	KLF17 (-87340), SLC6A9 (-14185)
R.55	45140440	45140869	chr1	TMEM53 (-428)
R.56	45671659	45672383	chr1	ZSWIM5 (+207)
R.57	45956646	45956843	chr1	TESK2 (+93)
R.58	46668822	46669345	chr1	LURAP1 (+78)
R.59	48937445	48937753	chr1	SPATA6 (+246)
R.60	57110722	57111387	chr1	PRKAA2 (+60)
R.61	61542620	61543011	chr1	NFIA (-4718)
R.62	62207966	62208645	chr1	INADL (+157)
R.63	63783070	63783375	chr1	FOXD3 (-5507), ATG4C (+533417)
R.64	63788255	63788429	chr1	FOXD3 (-388)
R.65	64971188	64971752	chr1	RAVER2 (-239308), CACHD1 (+34995)
R.66	65432439	65432742	chr1	JAK1 (-404)
R.67	65613383	65613405	chr1	AK4 (-119)
R.68	65613819	65614322	chr1	AK4 (+558)
R.69	68696709	68696988	chr1	DIRAS3 (-179535), WLS (+1379)
R.70	75198211	75198582	chr1	TYW3 (-443), CRYZ (+695)
R.71	85462885	85463364	chr1	MCOLN2 (-502)
R.72	85514226	85514340	chr1	MCOLN3 (-154)
R.73	85527754	85527879	chr1	MCOLN3 (-13688), SYDE2 (+138912)
R.75	91300387	91300446	chr1	BARHL2 (-117623), ZNF644 (+186613)
R.76	91869782	91870014	chr1	HFM1 (+528)
R.77	91870362	91870450	chr1	HFM1 (+20)
R.78	94344944	94345009	chr1	DNTTIP2 (-215)
R.79	95392682	95393115	chr1	CNN3 (-65)
R.80	99126913	99127069	chr1	SNX7 (-288)
R.81	100435258	100435430	chr1	SLC35A3 (-1)
R.82	100597416	100598159	chr1	TRMT13 (-931), SASS6 (+723)
R.83	107599246	107600091	chr1	PRMT6 (+368)
R.84	107683187	107683677	chr1	NTNG1 (-10)
R.85	107684339	107684751	chr1	NTNG1 (+1103), VAV3 (+823221)
R.86	109420034	109420722	chr1	GPSM2 (+2406), CLCC1 (+85733)
R.87	110052352	110052490	chr1	AMIGO1 (-117)
R.88	110453002	110453144	chr1	CSF1 (-182)
R.89	111022774	111023201	chr1	PROK1 (+29166), KCNA10 (+38809)
R.90	115212834	115212901	chr1	DENND2C (-212)
R.91	115632262	115632570	chr1	TSPAN2 (-331)
R.92	116961131	116961233	chr1	ATP1A1 (+44693), CD58 (+152479)
R.93	117909022	117909175	chr1	MAN1A2 (-972)
R.94	120838320	120838387	chr1	NOTCH2 (-226114), FCGR1B (+97583)
R.95	143743628	143743955	chr1	PPIAL4G (+24089)
R.96	144340103	144340251	chr1	PPIAL4B (+24069), NBPF8 (+193365)

R.97	144521081	144521462	chr1	NBPF9 (-290476), PPIAL4B (-157026)
R.98	147142184	147142653	chr1	ACP6 (+199)
R.99	150122500	150122690	chr1	PLEKHO1 (+425)
R.100	150266158	150266313	chr1	MRPS21 (-104)
R.101	150947566	150947589	chr1	CERS2 (-138)
R.102	151372572	151372652	chr1	PSMB4 (+602)
R.103	151762605	151762942	chr1	MRPL9 (-26734), TDRKH (+1118)
R.104	152635200	152635878	chr1	LCE2D (-333)
R.105	152814831	152815158	chr1	LCE6A (-349)
R.106	155294935	155295093	chr1	RUSC1 (+4296), ASH1L (+237309)
R.107	156594229	156594688	chr1	BCAN (-17281), HAPLN2 (+5373)
R.108	156696768	156697624	chr1	RRNAD1 (-1071), ISG20L2 (+1031)
R.109	159824086	159824538	chr1	C1orf204 (+825)
R.110	161067769	161067927	chr1	NIT1 (-20043), PVRL4 (-8459)
R.111	171810468	171810570	chr1	DNM3 (-102)
R.112	171811299	171811529	chr1	DNM3 (+793)
R.113	172113506	172114047	chr1	PIGC (+299449), DNM3 (+303156)
R.114	175162044	175162052	chr1	TNN (+125054), TNR (+550858)
R.115	177150789	177150934	chr1	FAM5B (+10229), SEC16B (+788188)
R.116	178693969	178694150	chr1	RALGPS2 (-240)
R.118	182584068	182584193	chr1	RGS16 (-10588), RGS8 (+57918)
R.119	182758815	182759062	chr1	NPL (-2455)
R.120	184006216	184006496	chr1	COLGALT2 (+507)
R.121	185703485	185703688	chr1	HMCN1 (-96)
R.122	186649877	186649985	chr1	PTGS2 (-372)
R.123	190447232	190448126	chr1	FAM5C (-920)
R.124	200992604	200992902	chr1	KIF21B (+75)
R.125	201123225	201123562	chr1	TMEM9 (+174)
R.126	202612633	202612948	chr1	SYT2 (-210)
R.127	202776680	202777459	chr1	SYT2 (-164489), KDM5B (+1528)
R.128	205538293	205538427	chr1	MFSD4 (+255)
R.129	205649794	205649807	chr1	SLC45A3 (-214)
R.131	211500034	211500571	chr1	TRAF5 (+124)
R.132	211589678	211590292	chr1	RD3 (+76274), TRAF5 (+89806)
R.133	212872426	212872986	chr1	BATF3 (+621)
R.134	213122829	213123520	chr1	VASH2 (-802)
R.135	213124685	213125130	chr1	VASH2 (+931)
R.136	215256254	215256451	chr1	KCNK2 (-226)
R.137	220701321	220701817	chr1	MARK1 (-330)
R.138	221916662	221916861	chr1	DUSP10 (-1244)
R.139	223316668	223316927	chr1	TLR5 (-6169), SUSD4 (+220603)
R.140	223537363	223538174	chr1	SUSD4 (-368)
R.141	224803837	224804149	chr1	CNIH3 (-2)
R.142	226309536	226310475	chr1	H3F3A (+58328), ACBD3 (+64425)
R.143	226497333	226497359	chr1	LIN9 (+88)
R.144	226737341	226737766	chr1	C1orf95 (+1053), ITPKB (+189310)
R.145	227915846	227915962	chr1	SNAP47 (-6793), ZNF678 (+164660)
R.146	227916287	227916426	chr1	SNAP47 (-6340), ZNF678 (+165113)
R.147	227922309	227922533	chr1	SNAP47 (-276)
R.148	228135758	228136256	chr1	WNT9A (-408)

R.150	228645045	228645634	chr1	HIST3H2BB (-468), HIST3H2A (+220)
R.151	228646224	228646970	chr1	HIST3H2A (-1037), HIST3H2BB (+789)
R.152	230561250	230561975	chr1	COG2 (-216625), PGBD5 (-48222)
R.153	231175275	231175978	chr1	TRIM67 (-123089), ARV1 (+60832)
R.154	231762664	231763026	chr1	DISC1 (+284)
R.155	235812840	235813452	chr1	GNG4 (+147)
R.156	236227521	236227653	chr1	NID1 (+875)
R.157	236227686	236228625	chr1	NID1 (+306)
R.158	239549600	239549798	chr1	CHRM3 (-242674)
R.159	242688081	242688226	chr1	PLD5 (-334)
R.160	245081988	245082320	chr1	HNRNPU (-54381), EFCAB2 (-51017)
R.161	245317950	245317980	chr1	KIF26B (-322)
R.164	247464072	247464677	chr1	ZNF124 (-129057), ZNF496 (+30670)
R.165	1746509	1746647	chr2	PXDN (+1700), TPO (+329345)
R.166	7005733	7005942	chr2	CMPK2 (-11)
R.167	7005960	7006339	chr2	CMPK2 (-323)
R.170	8825107	8825907	chr2	ID2 (+6532), KIDINS220 (+152248)
R.171	9144505	9144629	chr2	MBOAT2 (-625)
R.172	10861602	10862305	chr2	ATP6V1C2 (+179)
R.174	16080121	16080615	chr2	MYCN (-318)
R.175	17721984	17722068	chr2	VSNL1 (-401)
R.176	20190016	20190169	chr2	WDR35 (-201)
R.177	25565459	25565603	chr2	DNMT3A (-72)
R.178	27070750	27071255	chr2	DPYSL5 (+34)
R.179	27485780	27485967	chr2	SLC30A3 (+253)
R.180	27498234	27498334	chr2	DNAJC5G (-5)
R.181	29337946	29337988	chr2	C2orf71 (-40840), ALK (+806465)
R.182	29338258	29339076	chr2	C2orf71 (-41540), ALK (+805765)
R.183	30454146	30454279	chr2	LBH (-184)
R.184	31360693	31361104	chr2	GALNT14 (+114)
R.185	33171699	33172002	chr2	LTBP1 (-188)
R.186	33172444	33173163	chr2	LTBP1 (+765)
R.187	38303386	38303472	chr2	CYP1B1 (-106)
R.188	38303999	38304037	chr2	CYP1B1 (-695)
R.189	42795486	42795925	chr2	MTA3 (+49)
R.190	43864389	43864628	chr2	PLEKHH2 (+97)
R.194	46770281	46770357	chr2	RHOQ (+452)
R.195	47797590	47797963	chr2	KCNK12 (+301)
R.196	54014928	54015318	chr2	ERLEC1 (+906)
R.197	54682859	54683048	chr2	SPTBN1 (-468)
R.199	58654962	58655104	chr2	FANCL (-186548)
R.200	61404354	61404764	chr2	AHSA2 (-105)
R.201	62733114	62733617	chr2	TMEM17 (+110)
R.202	66803263	66803837	chr2	ETAA1 (-820901), MEIS1 (+141018)
R.203	70779764	70780723	chr2	TGFA (+903)
R.204	70780904	70781101	chr2	TGFA (+144)
R.205	71127565	71127779	chr2	VAX2 (-48)
R.206	72372162	72372426	chr2	CYP26B1 (+2873), DYSF (+678462)
R.207	72376574	72377174	chr2	CYP26B1 (-1707)
R.208	73520043	73520784	chr2	EGR4 (+415)

R.209	74055942	74055977	chr2	STAMBP (-187)
R.210	74056056	74056290	chr2	STAMBP (+26)
R.211	74709203	74709666	chr2	MRPL53 (-8986), LBX2 (+21008)
R.212	74942436	74942655	chr2	HK2 (-118562), SEMA4F (+61191)
R.213	75062130	75062663	chr2	POLE4 (-123222), HK2 (+1289)
R.214	80530948	80531500	chr2	LRRTM1 (+650)
R.215	85829320	85829672	chr2	TMEM150A (+43)
R.216	85830136	85830265	chr2	TMEM150A (-662)
R.217	85838641	85839032	chr2	USP39 (-4472)
R.218	86564436	86564659	chr2	REEP1 (+658)
R.219	88650305	88650823	chr2	TEX37 (-173605), THNSL2 (+179585)
R.220	91635085	91635595	chr2	NONE
R.221	95824277	95825271	chr2	ZNF514 (+578)
R.222	95831012	95831031	chr2	ZNF2 (-161)
R.223	95872892	95873465	chr2	PROM2 (-67022), ZNF2 (+41996)
R.224	96012328	96012595	chr2	FAHD2A (-56012), KCNIP3 (+49410)
R.225	96990858	96991327	chr2	ITPRIPL1 (-842)
R.226	97536097	97536287	chr2	SEMA4C (-484)
R.227	99552633	99553078	chr2	MGAT4A (-205267), TSGA10 (+218524)
R.228	102003778	102004252	chr2	CREG2 (+42)
R.230	112812751	112812775	chr2	TMEM87B (-37)
R.231	119532230	119532492	chr2	EN1 (+72893), INSIG2 (+686311)
R.232	119981334	119981960	chr2	STEAP3 (+240)
R.233	120188912	120189622	chr2	TMEM37 (-178)
R.234	120300987	120301835	chr2	ENSG00000163075 (-636)
R.235	122494667	122494691	chr2	MKI67IP (-180)
R.236	127863656	127864359	chr2	BIN1 (+923)
R.237	128615962	128616082	chr2	POLR2D (-291)
R.238	133428422	133428510	chr2	LYPD1 (-688)
R.239	150186634	150186923	chr2	LYPD6 (-272)
R.240	152955109	152955206	chr2	CACNB4 (+435)
R.241	152956036	152956135	chr2	CACNB4 (-493)
R.242	154335368	154335698	chr2	RPRM (-211)
R.243	158485309	158485436	chr2	ACVR1C (+144)
R.244	159824855	159825138	chr2	TANC1 (-186)
R.245	168149401	168149991	chr2	B3GALT1 (-525486), XIRP2 (+404699)
R.246	169312418	169312687	chr2	CERS6 (-206)
R.247	170218690	170219019	chr2	LRP2 (+340)
R.248	170220780	170221103	chr2	LRP2 (-1747)
R.249	171627721	171627790	chr2	GAD1 (-45316), SP5 (+55895)
R.250	171672205	171672899	chr2	GAD1 (-520)
R.251	171673547	171674437	chr2	GAD1 (+920)
R.252	173940432	173940582	chr2	ENSG00000091436 (-180)
R.253	175260612	175260842	chr2	CIR1 (-284), SCRN3 (+254)
R.255	175546916	175547399	chr2	WIPF1 (-47851), CHRNA1 (+82031)
R.256	179315948	179316083	chr2	DFNB59 (-147), PRKRA (-58)
R.257	180725637	180725691	chr2	ZNF385B (+568)
R.258	180725907	180726249	chr2	ZNF385B (+154)
R.259	187713876	187713964	chr2	ZSWIM2 (-23)

R.260	187714003	187714068	chr2	ZSWIM2 (-139)
R.261	189156425	189157348	chr2	GULP1 (-672)
R.262	189157361	189157566	chr2	GULP1 (-95)
R.263	191399046	191399880	chr2	TMEM194B (-15)
R.264	192109731	192109990	chr2	MYO1B (-254)
R.265	198669748	198670187	chr2	PLCL1 (+542)
R.266	200819837	200819881	chr2	C2orf47 (-181), TYW5 (+600)
R.267	202898016	202898349	chr2	FZD7 (-1127)
R.268	203736525	203736696	chr2	ICA1L (-246)
R.269	203879262	203879498	chr2	CYP20A1 (-224283), CARF (+102263)
R.270	207139431	207139445	chr2	ZDBF2 (+51)
R.271	207308087	207308375	chr2	ADAM23 (-32)
R.272	208634186	208634204	chr2	FZD5 (+92)
R.273	211036478	211036822	chr2	KANSL1L (-543)
R.274	213403212	213403734	chr2	ERBB4 (+92)
R.275	219265245	219266059	chr2	VIL1 (-18163), CTDSP1 (+1174)
R.276	219724404	219724891	chr2	WNT6 (+104)
R.277	219757464	219757724	chr2	CDK5R2 (-66783), WNT10A (+12509)
R.278	220252733	220253109	chr2	DNPEP (-184)
R.279	220407999	220408224	chr2	TMEM198 (-273), CHPF (+397)
R.280	222437040	222437130	chr2	EPHA4 (-75)
R.281	222437611	222438339	chr2	EPHA4 (-965)
R.282	223184510	223185049	chr2	SGPP2 (-104456), PAX3 (-21080)
R.283	230135879	230136150	chr2	PID1 (-34)
R.284	230785050	230785180	chr2	DNER (-205841), TRIP12 (+1610)
R.285	231729785	231730425	chr2	ITM2C (+490)
R.286	233497957	233498018	chr2	EFHD1 (+50)
R.287	233792732	233793120	chr2	C2orf82 (+58584), NGEF (+85056)
R.289	236403704	236404028	chr2	AGAP1 (+1115), GBX2 (+673146)
R.290	237087235	237087777	chr2	GBX2 (-10494), ASB18 (+85482)
R.291	241374411	241375382	chr2	GPC1 (-191)
R.292	241391930	241392617	chr2	GPC1 (+17186), ANKMY1 (+105106)
R.293	242642199	242642372	chr2	ING5 (+831)
R.295	8810980	8811092	chr3	OXTR (+267)
R.297	9291274	9291334	chr3	SRGAP3 (-241)
R.298	9774065	9774725	chr3	BRPF1 (+966)
R.299	9851598	9851847	chr3	TTLL3 (+51)
R.300	9851855	9852352	chr3	TTLL3 (+432)
R.301	12329166	12329223	chr3	TIMP4 (-128344), PPARG (-63776)
R.302	12329590	12330263	chr3	TIMP4 (-129076), PPARG (-63044)
R.304	13521120	13521176	chr3	HDAC11 (-517)
R.306	13921227	13921813	chr3	WNT7A (+98)
R.307	19189709	19189930	chr3	KCNH8 (-126)
R.308	23852449	23852775	chr3	RPL15 (-105424), UBE2E1 (+5218)
R.309	24536327	24536562	chr3	THRB (-179)
R.310	24562864	24563497	chr3	RARB (-906621), THRB (-26915)
R.311	27763266	27763865	chr3	EOMES (+423)
R.312	30936255	30936318	chr3	GADL1 (-30)
R.313	32509212	32509543	chr3	CMTM6 (+35522), CMTM7 (+76215)
R.314	37283665	37284460	chr3	GOLGA4 (-680)

R.315	37902741	37903684	chr3	CTDSPL (-452)
R.316	38071166	38071498	chr3	DLEC1 (-9364), PLCD1 (-5054)
R.317	39850621	39851007	chr3	MYRIP (-282)
R.318	40547336	40547368	chr3	ZNF620 (-178)
R.319	40547444	40547704	chr3	ZNF620 (+44)
R.320	44038250	44038484	chr3	TOPAZ1 (-245011), ABHD5 (+306005)
R.321	45729894	45730181	chr3	SACM1L (-716)
R.322	45883249	45883796	chr3	LZTFL1 (+135)
R.323	48936118	48936479	chr3	SLC25A20 (+127)
R.324	49157146	49157911	chr3	USP19 (+735)
R.325	49314539	49314636	chr3	USP4 (+62916), KLHDC8B (+105544)
R.326	49756904	49757438	chr3	AMIGO3 (+4178), RNF123 (+30239)
R.327	50275112	50275558	chr3	LSMEM2 (-41123), GNAI2 (+1951)
R.328	50383134	50383175	chr3	RASSF1 (-4883)
R.329	50388659	50388878	chr3	ZMYND10 (-4486), NPRL2 (-247), CYB561D2 (+480)
R.330	50540680	50541078	chr3	CACNA2D2 (-25)
R.331	50605003	50605156	chr3	HEMK1 (-1548), C3orf18 (+102)
R.332	50712233	50713087	chr3	DOCK3 (-12)
R.334	53080176	53080682	chr3	SFMBT1 (-345)
R.335	53880366	53880378	chr3	IL17RB (-235), CHDH (+45)
R.336	55521141	55521373	chr3	LRTM1 (-559156), WNT5A (+2716)
R.337	57542803	57543243	chr3	PDE12 (+1019), ARF4 (+40112)
R.338	58163493	58163614	chr3	DNASE1L3 (+36844), FLNB (+169427)
R.339	62358241	62358610	chr3	FEZF2 (+764)
R.340	64673301	64673501	chr3	ADAMTS9 (+275)
R.341	64673914	64674314	chr3	ADAMTS9 (-438)
R.342	66550735	66551541	chr3	LRIG1 (+218)
R.343	73674074	73674146	chr3	PDZRN3 (-19)
R.344	77088570	77089110	chr3	NONE
R.345	89163702	89164038	chr3	EPHA3 (+7196)
R.346	94656950	94657041	chr3	DHFRL1 (-874929)
R.348	112930675	112931126	chr3	BOC (+572)
R.349	115377360	115377939	chr3	GAP43 (+35293), LSAMP (+786728)
R.350	118753660	118754376	chr3	IGSF11 (-283)
R.352	120068640	120068700	chr3	GPR156 (-64729), FSTL1 (+101430)
R.353	120626881	120627406	chr3	STXBP5L (+225)
R.355	123603306	123603618	chr3	MYLK (-313)
R.356	123752504	123752573	chr3	CCDC14 (-71975), KALRN (-60989)
R.357	126075894	126076475	chr3	KLF15 (+100)
R.358	127347876	127347884	chr3	PODXL2 (-144)
R.359	128207255	128208037	chr3	DNAJB8 (-21835), GATA2 (+4382)
R.360	132756986	132757267	chr3	TMEM108 (-108)
R.361	133614790	133614804	chr3	RAB6B (-137)
R.362	134369828	134369939	chr3	KY (-20)
R.363	134513902	134514086	chr3	EPHB1 (-110)
R.364	134514881	134515422	chr3	EPHB1 (+1048)
R.365	138048335	138048402	chr3	NME9 (+359)
R.366	138170705	138170939	chr3	ESYT3 (+17367), CEP70 (+142376)
R.367	140770683	140770864	chr3	SPSB4 (+530)

R.368	141867862	141868459	chr3	TFDP2 (+202)
R.369	142607280	142607715	chr3	PCOLCE2 (+547)
R.370	142607939	142607981	chr3	PCOLCE2 (+85)
R.371	150803295	150804063	chr3	MED12L (-997)
R.372	153838788	153839028	chr3	ARHGEF26 (+116)
R.373	153839142	153839842	chr3	ARHGEF26 (+700)
R.374	158519410	158519645	chr3	MFSD1 (-126)
R.376	167098017	167098170	chr3	ZBBX (-516)
R.377	169755545	169755693	chr3	GPR160 (-98)
R.379	181429661	181429911	chr3	SOX2 (+64)
R.380	182400091	182400369	chr3	ATP11B (-111058), SOX2 (+970508)
R.381	183146412	183146663	chr3	MCF2L2 (-475)
R.382	183894761	183894778	chr3	ABCF3 (-9041), AP2M1 (+2127)
R.383	183978344	183978878	chr3	CAMK2N2 (+640)
R.384	184279509	184280384	chr3	EPHB3 (+375)
R.385	184530443	184530595	chr3	VPS8 (+588)
R.386	184870137	184870685	chr3	EHHADH (+101475), VPS8 (+340480)
R.387	187457128	187457588	chr3	RTP2 (-37013), BCL6 (+6157)
R.388	190580595	190580644	chr3	GMNC (-216)
R.389	192289245	192289293	chr3	HRASLS (-669645), FGF12 (-162431)
R.390	192634089	192634364	chr3	FGF12 (-507389), HRASLS (-324687)
R.391	194980766	194981274	chr3	LSG1 (-587814), XXYLT1 (+10876)
R.392	195634965	195635643	chr3	TNK2 (-12872), TFRC (+173756)
R.393	197476315	197476477	chr3	FYTTD1 (-225)
R.394	197687333	197687728	chr3	LMLN (+438)
R.395	197807975	197808147	chr3	LMLN (+120968)
R.396	331119	331685	chr4	ZNF141 (-222)
R.397	2956821	2957128	chr4	MFSD10 (-20790), NOP14 (+8137)
R.399	4108947	4109446	chr4	OTOP1 (+119419), ADRA2C (+341072)
R.400	4322607	4323265	chr4	ENSG00000168824 (-26931), ZBTB49 (+30958)
R.401	6201080	6201536	chr4	JAKMIP1 (+974)
R.402	6202379	6202553	chr4	JAKMIP1 (-184)
R.403	6989335	6990245	chr4	TADA2B (-55328), TBC1D14 (+78218)
R.404	7070051	7070632	chr4	GRPEL1 (-418)
R.406	8159729	8160379	chr4	ABLIM2 (+382)
R.407	13546157	13546316	chr4	NKX3-2 (+437)
R.408	24472254	24473145	chr4	PPARGC1A (-581000), DHX15 (+113473)
R.409	25032141	25032488	chr4	LGI2 (+186)
R.410	38665604	38665613	chr4	KLF3 (-208)
R.411	41362253	41362853	chr4	LIMCH1 (-251)
R.412	44450962	44451009	chr4	KCTD8 (-162)
R.413	48271826	48271923	chr4	TEC (+6)
R.414	48272059	48272120	chr4	TEC (-209)
R.415	53588038	53588397	chr4	USP46 (-62716), ERVMER34-1 (+29589)
R.416	55991701	55991943	chr4	KDR (-66)
R.417	56915329	56915900	chr4	CEP135 (+100468), AASDH (+338051)
R.418	62066539	62066990	chr4	LPHN3 (-1095)

R.419	66535203	66535403	chr4	EPHA5 (+758)
R.420	73434035	73434487	chr4	ADAMTS3 (+255)
R.421	77227621	77227631	chr4	FAM47E-STBD1 (+447)
R.422	78978565	78978708	chr4	FRAS1 (-87)
R.423	82393079	82393162	chr4	RASGEF1B (-52)
R.424	85504051	85504879	chr4	CDS1 (+333)
R.426	87515419	87515700	chr4	PTPN13 (+92)
R.427	87813277	87813585	chr4	SLC10A6 (-43015), AFF1 (-42723)
R.428	88928433	88928541	chr4	PKD2 (-333)
R.429	94749609	94750205	chr4	ATOH1 (-135)
R.430	95678939	95679705	chr4	BMPR1B (-293508), PDLIM5 (+302926)
R.431	96469343	96469634	chr4	UNC5C (+868)
R.432	96470584	96470887	chr4	UNC5C (-379)
R.434	105411974	105412536	chr4	TACR3 (-771282), CXXC4 (+3796)
R.435	109088779	109089770	chr4	LEF1 (+303)
R.436	109089890	109090828	chr4	LEF1 (-781)
R.437	110624749	110624915	chr4	CASP6 (-203)
R.438	111552353	111553161	chr4	PITX2 (-8498)
R.439	118006429	118006832	chr4	TRAM1L1 (+105)
R.440	119199621	119199731	chr4	PRSS12 (+74482), NDST3 (+244176)
R.441	121843717	121844192	chr4	PRDM5 (+49)
R.442	122872285	122872838	chr4	TRPC3 (+347)
R.444	140216130	140216277	chr4	MGARP (-14712), NDUFC1 (+6160)
R.445	142053623	142053970	chr4	RNF150 (+199)
R.446	142054417	142054743	chr4	RNF150 (-584)
R.447	146403942	146404134	chr4	SMAD1 (+75)
R.448	146856745	146857246	chr4	ZNF827 (+2627), MMAA (+317581)
R.449	152329364	152330215	chr4	PRSS48 (+131467), PET112 (+352385)
R.450	154170410	154170590	chr4	MND1 (-95301), TRIM2 (+44882)
R.451	157997086	157997367	chr4	GLRB (+18)
R.452	166300014	166300290	chr4	CPE (+58)
R.453	171011099	171011264	chr4	AADAT (-64)
R.454	175443569	175443797	chr4	HPGD (+366)
R.455	176986856	176987359	chr4	GPM6A (-63625), SPATA4 (+129714)
R.456	183065068	183065519	chr4	TENM3 (-99288)
R.457	183369754	183370138	chr4	TENM3 (+205364), DCTD (+468586)
R.458	184826421	184826704	chr4	STOX2 (+54)
R.459	186064512	186064648	chr4	SLC25A4 (+185)
R.460	187111940	187112145	chr4	CYP4V2 (-631)
R.461	187644620	187645448	chr4	FAT1 (-25)
R.462	190905990	190906107	chr4	FRG2 (+42363), FRG1 (+44106)
R.463	1008828	1009204	chr5	NKD2 (+72)
R.464	1385979	1386150	chr5	CLPTM1L (-40851), SLC6A3 (+59480)
R.465	5423192	5423457	chr5	ADAMTS16 (+282882), MED10 (+955382)
R.467	6411286	6411956	chr5	UBE2QL1 (-37115), MED10 (-32914)
R.468	7826972	7827133	chr5	FASTKD3 (+42062), ADCY2 (+430732)
R.469	9545207	9545976	chr5	SEMA5A (+595)
R.470	14581771	14582549	chr5	FAM105B (-82697), TRIO (+438349)
R.471	16616805	16617290	chr5	FAM134B (+119)

R.472	17216512	17217093	chr5	BASP1 (-866)
R.473	34915890	34916181	chr5	BRIX1 (+555), RAD1 (+835)
R.474	35229788	35229908	chr5	PRLR (+843)
R.475	36066944	36067091	chr5	UGT3A2 (-25)
R.476	41510801	41510851	chr5	PLCXD3 (-123)
R.477	41869963	41870316	chr5	OXCT1 (+481)
R.478	42423577	42423718	chr5	GHR (-231)
R.479	49962729	49963156	chr5	PARP8 (+171)
R.481	52856497	52856570	chr5	NDUFS4 (+69)
R.482	54469057	54469144	chr5	GPX8 (+13155), MCIDAS (+54042)
R.483	56246561	56247035	chr5	MIER3 (+1154), SETD9 (+41711)
R.484	63461566	63461803	chr5	RNF180 (-24)
R.485	63802106	63802280	chr5	RGS7BP (+67)
R.486	65891878	65892187	chr5	MAST4 (-156)
R.487	68788469	68789255	chr5	OCLN (+743)
R.488	72743968	72744581	chr5	FOXD1 (+77)
R.489	76326080	76326240	chr5	AGGF1 (-50)
R.490	78808852	78809348	chr5	HOMER1 (+940)
R.491	78809740	78810173	chr5	HOMER1 (+83)
R.492	79865808	79866099	chr5	ANKRD34B (+353)
R.493	81045966	81046758	chr5	SSBP2 (+710)
R.494	81147781	81148310	chr5	ATG10 (-119798), SSBP2 (-100974)
R.495	82767450	82767617	chr5	VCAN (+250)
R.496	92957059	92957400	chr5	NR2F1 (+38187), POU5F2 (+120113)
R.497	99870926	99870957	chr5	FAM174A (-67)
R.498	99871233	99871651	chr5	FAM174A (+433)
R.499	110559581	110560172	chr5	CAMK4 (+93)
R.500	111093475	111093927	chr5	STARD4 (-245493), NREP (+218927)
R.501	111755158	111755481	chr5	EPB41L4A (-307)
R.503	115910592	115911468	chr5	SEMA6A (-400)
R.504	119801142	119801431	chr5	FAM170A (+835994)
R.505	121647642	121648022	chr5	SNCAIP (+68)
R.506	122372327	122372722	chr5	PPIC (-89)
R.507	122424706	122425425	chr5	PRDM6 (+250)
R.508	125936257	125936553	chr5	PHAX (+445)
R.509	128430663	128431096	chr5	ISOC1 (+436)
R.510	131593106	131593261	chr5	PDLIM4 (-180)
R.511	131705035	131705742	chr5	SLC22A5 (-55)
R.512	131892515	131892956	chr5	RAD50 (+106)
R.513	133450017	133450281	chr5	TCF7 (-253)
R.514	134369116	134370051	chr5	PITX1 (+404)
R.515	134871658	134871805	chr5	NEUROG1 (-93)
R.516	135170208	135170281	chr5	SLC25A48 (-171)
R.517	137801011	137801165	chr5	EGR1 (-91)
R.518	138533428	138533871	chr5	SIL1 (+437)
R.519	138609379	138609566	chr5	SIL1 (-75386), MATR3 (-19864)
R.520	139088815	139089549	chr5	PSD2 (-86224), CXXC5 (+61305)
R.523	140306054	140306205	chr5	PCDHAC1 (-348)
R.524	140306249	140306458	chr5	PCDHAC1 (-124)
R.525	140346199	140346309	chr5	PCDHAC2 (+434)

R.526	140700576	140700638	chr5	TAF7 (-277)
R.527	140782948	140783647	chr5	PCDHGB6 (-4472), PCDHGA9 (+778)
R.529	141705411	141705539	chr5	SPRY4 (-909)
R.530	142149936	142150401	chr5	ARHGAP26 (+220)
R.531	146258512	146258546	chr5	GPR151 (-362776), PPP2R2B (+177008)
R.532	148651359	148651771	chr5	AFAP1L1 (+131)
R.533	150036071	150036581	chr5	MYOZ3 (-4118)
R.535	159739797	159740383	chr5	CCNJL (-483)
R.536	159797753	159797775	chr5	C1QTNF2 (-116)
R.537	167718124	167718524	chr5	WWC1 (-831)
R.538	167718982	167719548	chr5	WWC1 (+110)
R.539	168726889	168727752	chr5	SLIT3 (+812)
R.541	170846083	170846517	chr5	FGF18 (-360)
R.542	172068019	172068142	chr5	NEURL1B (-188)
R.543	173472645	173472732	chr5	ENSG00000170091 (+82)
R.544	176237381	176237996	chr5	UNC5A (+211)
R.545	176514005	176514432	chr5	FGFR4 (+332)
R.546	176873646	176873656	chr5	PRR7 (-145)
R.547	176900806	176901453	chr5	DBN1 (-931)
R.548	177099102	177099378	chr5	B4GALT7 (+72139), PROP1 (+324003)
R.549	178157346	178157550	chr5	ZNF354A (+255)
R.550	178157792	178157825	chr5	ZNF354A (-106)
R.551	178450758	178451078	chr5	ZNF879 (+165)
R.552	179563738	179563839	chr5	RNF130 (-64671), RASGEF1C (+1560)
R.553	179635731	179635981	chr5	RASGEF1C (-70507), MAPK9 (+83243)
R.554	180631604	180632555	chr5	TRIM7 (+92)
R.555	180644590	180645302	chr5	TRIM7 (-12774), TRIM41 (-5344)
R.556	3227741	3227981	chr6	TUBB2B (+108)
R.557	3752849	3753083	chr6	PXDC1 (-706)
R.558	6008937	6009298	chr6	NRN1 (-1918)
R.559	10413233	10413523	chr6	OFCC1 (-473826), TFAP2A (+2092)
R.560	10419400	10420035	chr6	TFAP2A (-4248)
R.561	13925505	13925567	chr6	RNF182 (+438)
R.562	16238667	16238691	chr6	GMPR (-132)
R.563	17393616	17394417	chr6	CAP2 (+570)
R.564	24403115	24403143	chr6	MRS2 (-53)
R.565	24403287	24403646	chr6	MRS2 (+285)
R.566	24646545	24646782	chr6	KIAA0319 (-281)
R.567	24911193	24911308	chr6	FAM65B (-56)
R.568	24911334	24911615	chr6	FAM65B (-280)
R.569	26043732	26043990	chr6	HIST1H3C (-1778), HIST1H2BB (+24)
R.570	26522136	26522904	chr6	HMGN4 (-16113), BTN1A1 (+21071)
R.571	27247586	27247970	chr6	ZNF391 (-108719), PRSS16 (+32276)
R.572	27798455	27798932	chr6	HIST1H4K (+611)
R.573	27833095	27833129	chr6	HIST1H2AL (+78)
R.574	27834606	27835363	chr6	HIST1H1B (+374)
R.575	27835400	27835460	chr6	HIST1H1B (-71)
R.576	27839850	27840105	chr6	HIST1H1B (-4619), HIST1H3I (+121)
R.577	28555213	28555324	chr6	SCAND3 (-157)

R.578	28574571	28574592	chr6	SCAND3 (-19470), TRIM27 (+317184)
R.580	28601417	28601440	chr6	SCAND3 (-46317), TRIM27 (+290337)
R.581	28603059	28603230	chr6	SCAND3 (-48033), TRIM27 (+288621)
R.582	28831713	28831743	chr6	SCAND3 (-276616), TRIM27 (+60038)
R.583	28831788	28831873	chr6	SCAND3 (-276719), TRIM27 (+59935)
R.584	28832107	28832121	chr6	SCAND3 (-277002), TRIM27 (+59652)
R.585	28832245	28832274	chr6	SCAND3 (-277148), TRIM27 (+59506)
R.586	28864178	28864188	chr6	SCAND3 (-309071), TRIM27 (+27583)
R.587	28979290	28979338	chr6	ZNF311 (-6277), OR2W1 (+33703)
R.588	28979424	28979445	chr6	ZNF311 (-6398), OR2W1 (+33582)
R.589	29596540	29596874	chr6	GABBR1 (+4255), OR2H2 (+41024)
R.590	29691135	29691168	chr6	HLA-F (-33)
R.591	29691888	29691899	chr6	HLA-F (+709)
R.592	29760495	29760659	chr6	HLA-G (-34179), HLA-F (+69392)
R.593	29760754	29760773	chr6	HLA-G (-33992), HLA-F (+69579)
R.594	29760823	29760864	chr6	HLA-G (-33912), HLA-F (+69659)
R.595	29856182	29856203	chr6	HLA-A (-52844), HLA-G (+61437)
R.596	29856278	29856363	chr6	HLA-A (-52716), HLA-G (+61565)
R.597	30028447	30028582	chr6	ZNRD1 (-516)
R.598	30227294	30227373	chr6	TRIM39 (-67287), TRIM26 (-46191)
R.599	30227783	30227825	chr6	TRIM39 (-66817), TRIM26 (-46661)
R.600	30228007	30228058	chr6	TRIM39 (-66588), TRIM26 (-46890)
R.601	30313495	30313546	chr6	RPP21 (+613)
R.602	30523242	30523350	chr6	PRR3 (-1367)
R.603	30523512	30523788	chr6	PRR3 (-1013)
R.604	30633339	30633353	chr6	DHX16 (+7468), C6orf136 (+18530)
R.605	30640154	30640219	chr6	DHX16 (+627)
R.606	30640270	30640347	chr6	DHX16 (+505)
R.607	30647034	30647104	chr6	DHX16 (-6255), PPP1R18 (+8603)
R.608	30851632	30851753	chr6	DDR1 (-168)
R.609	30852069	30852307	chr6	DDR1 (+327)
R.610	30852545	30852823	chr6	DDR1 (+823)
R.612	31093846	31094185	chr6	CDSN (-5793), PSORS1C2 (+13111)
R.613	31110473	31110647	chr6	PSORS1C2 (-3433)
R.615	31324259	31324512	chr6	HLA-B (+578)
R.617	31589593	31590513	chr6	PRRC2A (+1551), BAG6 (+30424)
R.618	31671064	31671159	chr6	LY6G6F (-3550), ABHD16A (+109)
R.619	31688160	31688428	chr6	C6orf25 (-2867), LY6G6C (+1328)
R.620	31762650	31762688	chr6	VWA7 (-17598), VARS (+1061)
R.621	32122436	32122484	chr6	PRRT1 (-2731), PPT2-EGFL8 (+461)
R.622	32153540	32153657	chr6	AGER (-1498)
R.623	32172007	32172027	chr6	GPSM3 (-11334), NOTCH4 (+19827)
R.624	32182002	32182174	chr6	GPSM3 (-21405), NOTCH4 (+9756)
R.625	32187918	32188404	chr6	GPSM3 (-27478), NOTCH4 (+3683)
R.626	32829025	32829208	chr6	PSMB9 (+7179), HLA-DMB (+79730)
R.627	33053576	33053608	chr6	HLA-DPA1 (-5105), COL11A2 (+106684)
R.628	33084814	33084840	chr6	HLA-DPA1 (-36340), COL11A2 (+75449)

R.629	33172388	33172467	chr6	RXRB (-4037), RING1 (-3844), HSD17B8 (+9)
R.630	33216612	33216663	chr6	VPS52 (+23033), RING1 (+40366)
R.631	33256555	33256621	chr6	PFDN6 (-491), WDR46 (+716)
R.632	33291519	33291586	chr6	DAXX (-762)
R.633	33385314	33385337	chr6	SYNGAP1 (-2615), CUTA (+768)
R.634	33395433	33395936	chr6	ZBTB9 (-26671), SYNGAP1 (+7744)
R.636	34111750	34111965	chr6	HMGA1 (-92792), GRM4 (-10415)
R.637	37137738	37137753	chr6	PIM1 (-233)
R.638	37665051	37665626	chr6	MDGA1 (+427)
R.639	39693159	39693360	chr6	KIF6 (-79)
R.641	45389860	45389871	chr6	SUPT3H (-44196), CLIC5 (+658266)
R.642	46097530	46097671	chr6	ENPP4 (-129)
R.643	46620647	46620666	chr6	CYP39A1 (-134), SLC25A27 (-21)
R.644	46703225	46703235	chr6	PLA2G7 (-151)
R.645	47277552	47277683	chr6	TNFRSF21 (+23)
R.646	53516423	53517075	chr6	GCLC (-106822), KLHL31 (+13757)
R.647	56820286	56820869	chr6	DST (-312784), KIAA1586 (-90769)
R.648	70577059	70577198	chr6	COL19A1 (+666)
R.649	71666803	71666858	chr6	B3GAT2 (-90)
R.650	72596472	72596566	chr6	RIMS1 (-208)
R.651	75914803	75915344	chr6	COL12A1 (+693)
R.652	75915787	75915885	chr6	COL12A1 (-69)
R.653	76059576	76059756	chr6	SENP6 (-252097), TMEM30A (-64982)
R.654	79577069	79577232	chr6	IRAK1BP1 (-38)
R.655	80656973	80657412	chr6	ELOVL4 (+104)
R.656	82461874	82462430	chr6	IBTK (+495319)
R.657	82462460	82462656	chr6	IBTK (+494913)
R.658	83072774	83073156	chr6	TPBG (-383)
R.659	84418433	84418724	chr6	SNAP91 (+548)
R.660	84562888	84563042	chr6	RIPPLY2 (-20)
R.661	87861586	87862264	chr6	ZNF292 (-3342)
R.662	90121670	90121836	chr6	RRAGD (+236)
R.663	90142885	90142892	chr6	ANKRD6 (-129231), RRAGD (-20900)
R.664	91005564	91006321	chr6	BACH2 (+518)
R.665	91006597	91006647	chr6	BACH2 (-161)
R.666	97372234	97372626	chr6	NDUFAF4 (-26673), MMS22L (+358622)
R.667	99282348	99283304	chr6	POU3F2 (+246)
R.668	99797222	99797842	chr6	FAXC (-1)
R.669	105584216	105585179	chr6	BVES (+351)
R.670	105628044	105628147	chr6	POPDC3 (-361)
R.671	106534117	106534239	chr6	PRDM1 (-17)
R.672	106959592	106960065	chr6	AIM1 (+99)
R.673	108454984	108455272	chr6	OSTM1 (-59187), NR2E1 (-32134)
R.674	109762382	109762635	chr6	PPIL6 (-135), SMPD2 (+543)
R.675	111408745	111408761	chr6	SLC16A10 (-28)
R.676	114663223	114663972	chr6	HS3ST5 (+611)
R.677	116691851	116692265	chr6	DSE (+537)
R.678	116937689	116937974	chr6	RSPH4A (+182)

R.679	117586538	117586666	chr6	VGLL2 (-135)
R.680	117586817	117587588	chr6	VGLL2 (+466)
R.681	119670533	119671260	chr6	MAN1A1 (+29)
R.682	124124875	124125002	chr6	NKAIN2 (-347)
R.683	126069645	126070385	chr6	HEY2 (-711)
R.684	133562087	133562193	chr6	EYA4 (-349)
R.685	133562479	133562492	chr6	EYA4 (-3)
R.686	135501674	135501969	chr6	MYB (-631)
R.687	137242738	137243410	chr6	SLC35D3 (-328)
R.688	144385609	144385771	chr6	PLAGL1 (-95575), SF3B5 (+31064)
R.689	146136083	146136563	chr6	FBXO30 (-434)
R.690	146348616	146348890	chr6	GRM1 (-29)
R.691	150311726	150312368	chr6	ULBP1 (+26904), RAET1L (+34560)
R.692	151562026	151562634	chr6	AKAP12 (+1196), ZBTB2 (+150353)
R.693	152128411	152128471	chr6	ESR1 (+116810), SYNE1 (+830093)
R.694	153451251	153451812	chr6	ENSG00000213121 (-100923), RGS17 (-86354)
R.695	153452233	153452732	chr6	ENSG00000213121 (-99972), RGS17 (-87305)
R.696	160182438	160182624	chr6	ACAT2 (+1171), TCP1 (+28250)
R.697	163148718	163148853	chr6	PARK2 (+17), PACRG (+622)
R.698	163149167	163149320	chr6	PARK2 (-441)
R.700	166400494	166400665	chr6	SDIM1 (-90855), T (+181527)
R.701	167275843	167276359	chr6	RPS6KA2 (-62)
R.702	167764620	167765110	chr6	TTLL2 (+26291), TCP10 (+33089)
R.706	170124933	170125241	chr6	PHF10 (-936)
R.707	170249394	170249553	chr6	C6orf70 (+97753), DLL1 (+350087)
R.710	170863845	170864351	chr6	PSMB1 (-1669), TBP (+677)
R.715	2154026	2154175	chr7	MAD1L1 (+118777), ELFN1 (+426346)
R.716	2354203	2354294	chr7	SNX8 (-150)
R.719	5466405	5467366	chr7	TNRC18 (-3709)
R.720	6745725	6746208	chr7	ZNF12 (+587)
R.722	12610175	12610384	chr7	SCIN (-32)
R.723	16793463	16793634	chr7	TSPAN13 (+389)
R.724	19157193	19157420	chr7	TWIST1 (-12)
R.725	20817296	20817927	chr7	SP8 (+8893), ABCB5 (+162782)
R.727	25990679	25991027	chr7	NPVF (-722748), NFE2L3 (-201007)
R.728	28219613	28220533	chr7	JAZF1 (+289)
R.729	29234470	29234993	chr7	CPVL (+160), CHN2 (+704)
R.730	29846259	29846620	chr7	WIPF3 (-27901), PRR15 (+243013)
R.731	31232764	31232861	chr7	ADCYAP1R1 (+130003), NEUROD6 (+147695)
R.732	33944415	33944635	chr7	BMPER (+2)
R.733	33944927	33945297	chr7	BMPER (+589)
R.735	36193062	36193344	chr7	EEPD1 (+445)
R.737	37488428	37488564	chr7	ELMO1 (+356)
R.738	37488858	37488936	chr7	ELMO1 (-45)
R.739	38670412	38670804	chr7	AMPH (+559)
R.740	38670957	38671001	chr7	AMPH (+188)
R.741	39453784	39454090	chr7	RALA (-209145), POU6F2 (+436339)

R.742	42533001	42533206	chr7	GLI3 (-256492), ENSG00000256646 (+438669)
R.743	43797898	43798100	chr7	BLVRA (-287)
R.744	43965618	43966260	chr7	UBE2D4 (-104)
R.745	44622302	44622423	chr7	TMED4 (-505)
R.746	44837033	44837140	chr7	PPIA (+808)
R.747	44924778	44925080	chr7	PURB (+31)
R.749	50861549	50861602	chr7	GRB10 (-61526), COBL (+522920)
R.750	51384621	51384931	chr7	COBL (-280)
R.751	64023591	64023661	chr7	ZNF680 (-142)
R.752	64254770	64255045	chr7	ZNF138 (+105)
R.753	64712379	64712896	chr7	ERV3-1 (-245607), ZNF92 (-126074)
R.754	65958983	65959203	chr7	KCTD7 (-246550), TPST1 (+288907)
R.755	69064884	69065351	chr7	AUTS2 (+799)
R.757	71217124	71217320	chr7	WBSCR17 (+620067), CALN1 (+660136)
R.758	72722304	72722951	chr7	NSUN5 (+185)
R.759	73867831	73867948	chr7	GTF2IRD1 (-410)
R.760	75114950	75115714	chr7	POM121C (+216)
R.761	75796215	75796528	chr7	HSPB1 (-135489), MDH2 (+118987)
R.762	76178544	76178636	chr7	UPK3B (+38845), POMZP3 (+77968)
R.763	79764176	79764387	chr7	GNAI1 (+178)
R.764	82792053	82792284	chr7	PCLO (+77)
R.766	86688919	86689099	chr7	KIAA1324L (+6)
R.767	87257416	87257547	chr7	ABCB4 (-152427), ABCB1 (+85082)
R.768	87563569	87563850	chr7	ADAM22 (+8)
R.769	90225159	90226043	chr7	CDK14 (-113111), CLDN12 (+192889)
R.770	91510405	91510597	chr7	MTERF (-467)
R.771	92466331	92466688	chr7	CDK6 (-3279)
R.772	94284411	94284439	chr7	PEG10 (-1212), SGCE (+1011)
R.773	94284526	94284750	chr7	PEG10 (-999), SGCE (+798)
R.774	94284893	94284927	chr7	PEG10 (-727), SGCE (+526)
R.776	95226035	95226193	chr7	PDK4 (-311)
R.777	95401131	95401838	chr7	DYNC1I1 (-417)
R.778	96634623	96634914	chr7	DLX6 (-91)
R.781	97601102	97601469	chr7	ASNS (-99432), OCM2 (+18222)
R.782	98971852	98972197	chr7	ARPC1B (-328)
R.783	99155739	99155971	chr7	ZNF655 (-593)
R.784	99156362	99156634	chr7	ZNF655 (+50)
R.785	100809451	100809985	chr7	VGF (-844)
R.786	101386679	101387242	chr7	MYL10 (-114385), CUX1 (-72330)
R.787	101398152	101398184	chr7	MYL10 (-125592), CUX1 (-61123)
R.788	101500014	101500514	chr7	SH2B2 (-428141), CUX1 (+40973)
R.789	102004098	102004775	chr7	PRKRIP1 (+93)
R.790	102789509	102790119	chr7	NAPEPLD (-185)
R.791	102984348	102984880	chr7	PSMC2 (-747), DNAJC2 (+706)
R.792	107643692	107644249	chr7	LAMB1 (-271)
R.793	117854511	117854765	chr7	ANKRD7 (-10092), NAA38 (+30552)
R.794	119913576	119913771	chr7	KCND2 (-48)
R.795	121512781	121513058	chr7	PTPRZ1 (-223)

R.796	127291658	127291928	chr7	SND1 (-441)
R.797	128470599	128471146	chr7	FLNC (+442)
R.798	129418470	129419139	chr7	NRF1 (+167250), UBE2H (+173984)
R.799	129691233	129691259	chr7	ZC3HC1 (+45)
R.801	130418324	130419064	chr7	KLF14 (+194)
R.802	130419614	130419754	chr7	KLF14 (-796)
R.803	134001208	134001556	chr7	SLC35B4 (+421)
R.805	138818186	138818325	chr7	TTC26 (-268)
R.806	139762256	139762806	chr7	PARP12 (+990)
R.807	140339480	140340155	chr7	DENND2A (+758)
R.808	140340980	140341335	chr7	DENND2A (-582)
R.809	140772810	140773578	chr7	TMEM178B (-838)
R.810	140774161	140774559	chr7	TMEM178B (+328)
R.811	149570992	149571373	chr7	ATP6V0E2 (+1126), ACTR3C (+449575)
R.812	150065170	150065678	chr7	REPIN1 (-465)
R.813	150102660	150102724	chr7	GIMAP8 (-45026), ZNF775 (+26268)
R.814	150672175	150672364	chr7	KCNH2 (+3133), AOC1 (+150555)
R.815	150675025	150675516	chr7	KCNH2 (+132)
R.817	150756408	150756491	chr7	CDK5 (-833)
R.818	150778201	150778818	chr7	FASTK (-579)
R.819	150778926	150779110	chr7	AGAP3 (-4811), FASTK (-1087)
R.820	150864974	150865291	chr7	GBX1 (-498)
R.821	151573694	151573913	chr7	PRKAG2 (+406)
R.822	151722461	151722536	chr7	GALNT11 (-48884), GALNTL5 (+69019)
R.823	153749738	153749759	chr7	DPP6 (-16)
R.824	155249941	155250220	chr7	EN2 (-743)
R.825	156803122	156803562	chr7	MNX1 (+3)
R.828	182402	182981	chr8	ZNF596 (+308)
R.830	356385	357061	chr8	FBXO25 (-232)
R.831	1797886	1798729	chr8	MYOM2 (-194847), ARHGEF10 (+26166)
R.832	6692148	6692544	chr8	DEFB1 (+43198), AGPAT5 (+126468)
R.833	10873155	10874016	chr8	PINX1 (-176200), XKR6 (+185289)
R.834	11057947	11058770	chr8	XKR6 (+516)
R.835	11059038	11059303	chr8	XKR6 (-296)
R.836	11204541	11204833	chr8	BLK (-146823), SLC35G5 (+16290)
R.838	12523148	12523225	chr8	DEFB130 (-347362), LONRF1 (+89812)
R.839	15397729	15398053	chr8	TUSC3 (+99)
R.840	17354715	17355239	chr8	MTMR7 (-84141), SLC7A2 (-41309)
R.841	18871817	18872143	chr8	PSD3 (-784)
R.842	19171706	19172126	chr8	SH2D4A (+788)
R.843	19615134	19615412	chr8	CSGALNACT1 (-155267), INTS10 (-59378)
R.844	19796561	19796844	chr8	LPL (+221)
R.845	21944986	21945413	chr8	NUDT18 (+21732), DMTN (+28483)
R.846	23539627	23539955	chr8	NKX3-1 (+649)
R.847	26724001	26724836	chr8	ADRA1A (-1497)
R.848	27850175	27850258	chr8	SCARA5 (+27)
R.849	29953364	29953601	chr8	LEPROTL1 (+487)
R.850	30769215	30769864	chr8	TEX15 (-62932), PURG (+121691)

R.851	30890617	30890632	chr8	WRN (-692), PURG (+606)
R.853	41166738	41167278	chr8	SFRP1 (+8)
R.854	49833948	49834643	chr8	SNAI2 (+3)
R.855	53478053	53478102	chr8	FAM150A (-11)
R.856	57069907	57070217	chr8	MOS (-43521), PLAG1 (+53776)
R.857	61193715	61194072	chr8	CA8 (+77)
R.858	63998327	63998667	chr8	TTPA (+115)
R.859	67088895	67089887	chr8	CRH (+1569), TRIM55 (+50260)
R.860	68864523	68864546	chr8	PREX2 (+182)
R.861	70746365	70747067	chr8	SLCO5A1 (+583)
R.862	70747337	70747440	chr8	SLCO5A1 (-90)
R.864	73449127	73449684	chr8	KCNB2 (-220)
R.865	75232650	75233613	chr8	JPH1 (+431)
R.866	80524467	80525214	chr8	STMN2 (+1454), HEY1 (+155251)
R.867	81599484	81599569	chr8	ZNF704 (+187489), ZBTB10 (+201625)
R.868	82023929	82024462	chr8	PAG1 (+107)
R.869	86375501	86376277	chr8	CA2 (-192)
R.870	93114294	93114951	chr8	RUNX1T1 (-6917), TRIQK (+863750)
R.871	97506180	97507079	chr8	CPQ (-150825), SDC2 (+1051)
R.872	98881351	98881842	chr8	MATN2 (-18608), LAPTM4B (+94312)
R.873	101170048	101170565	chr8	SPAG1 (-349)
R.874	104512317	104513083	chr8	RIMS2 (-415)
R.876	109799215	109799909	chr8	TMEM74 (+282)
R.877	110657174	110657184	chr8	SYBU (+46841), EBAG9 (+104773)
R.878	119964440	119964630	chr8	TNFRSF11B (-96)
R.879	120886151	120886450	chr8	DEPTOR (+344)
R.880	121824405	121824433	chr8	SNTB1 (+1094), MTBP (+366753)
R.881	122652023	122652162	chr8	SNTB1 (-826580), HAS2 (+1537)
R.882	124085299	124085669	chr8	TBC1D31 (+564)
R.883	124169368	124169819	chr8	TBC1D31 (+84674), ZHX1 (+117141)
R.884	124552839	124553600	chr8	FBXO32 (+226)
R.885	127569650	127570165	chr8	FAM84B (+730)
R.886	127570482	127570908	chr8	FAM84B (-57)
R.888	140718042	140718385	chr8	KCNK9 (-2915)
R.890	143695529	143695901	chr8	ARC (+1118), BAI1 (+164924)
R.891	144373008	144373277	chr8	ZNF696 (-416)
R.892	144512192	144512773	chr8	MAFA (+93)
R.893	144640378	144640755	chr8	GSDMD (+5190), NAPRT1 (+20216)
R.894	144798309	144798331	chr8	MAPK15 (-109)
R.895	144815967	144816217	chr8	FAM83H (-121)
R.896	145047979	145048235	chr8	PLEC (-23063), PARP10 (+12515)
R.897	146024629	146024786	chr8	ZNF517 (+447)
R.898	4299549	4299830	chr9	GLIS3 (+226)
R.899	78506874	78507533	chr9	PCSK5 (+1644), RFK (+502217)
R.900	86571432	86571904	chr9	C9orf64 (-36)
R.901	89561826	89562473	chr9	GAS1 (-46)
R.902	94710679	94711647	chr9	NFIL3 (-525019), ROR2 (+1281)
R.903	94711818	94712472	chr9	ROR2 (+299)
R.904	95570676	95571589	chr9	BICD2 (-44039), ZNF484 (+69085)
R.906	99381359	99382235	chr9	CDC14B (+315)

R.907	99801371	99801723	chr9	CTSV (+45)
R.908	100264063	100264289	chr9	TMOD1 (+714)
R.909	103235181	103236040	chr9	TMEFF1 (+216)
R.910	107526283	107526516	chr9	NIPSNAP3A (+16431), ABCA1 (+164118)
R.911	130516077	130516816	chr9	TOR2A (-18851), SH2D3C (+24573)
R.912	131872018	131872920	chr9	PPP2R4 (-1128), CRAT (+614)
R.913	134378512	134378745	chr9	POMT1 (+317)
R.914	136343843	136344026	chr9	SLC2A6 (+324)
R.915	140081032	140081972	chr9	SSNA1 (-1597), ANAPC2 (+1487)
R.916	140196785	140197048	chr9	NRARP (-214)
R.921	5708674	5708797	chr10	ASB13 (-195)
R.922	6214016	6214079	chr10	PFKFB3 (-30846), RBM17 (+82739)
R.923	6962135	6962295	chr10	PRKCQ (-339952), SFMBT2 (+489088)
R.925	8096633	8096650	chr10	GATA3 (-14)
R.926	11206813	11207527	chr10	CELF2 (+147277), USP6NL (+367104)
R.927	13481909	13482639	chr10	SEPHS1 (-91977), BEND7 (+62702)
R.928	15411709	15412027	chr10	NMT2 (-201176), FAM171A1 (+1190)
R.929	17271294	17271519	chr10	VIM (+1149), ST8SIA6 (+224922)
R.930	18429703	18430144	chr10	CACNB2 (+318)
R.931	27150106	27150228	chr10	ABI1 (-303)
R.932	28033840	28034669	chr10	MKX (+734)
R.933	28034856	28035208	chr10	MKX (-43)
R.934	31609891	31609960	chr10	ZEB1 (+1825), ARHGAP12 (+607809)
R.935	35415980	35416245	chr10	CREM (-275)
R.937	42863508	42863550	chr10	ZNF33B (+270463)
R.938	43277850	43277941	chr10	BMS1 (-353)
R.939	43724742	43725411	chr10	FXYD4 (-142013), RASGEF1A (-20445)
R.940	44143806	44144525	chr10	ZNF32 (-14)
R.941	46168551	46169183	chr10	ZFAND4 (-1071)
R.942	47151300	47151566	chr10	ANXA8L1 (+22607), NPY4R (+67899)
R.943	51488973	51489643	chr10	AGAP7 (-2981)
R.944	52833902	52834076	chr10	PRKG1 (+55)
R.945	52834121	52834850	chr10	PRKG1 (+552)
R.946	64028056	64028664	chr10	RTKN2 (+106)
R.947	64133812	64133896	chr10	ZNF365 (-97)
R.949	64576118	64576388	chr10	EGR2 (-138)
R.950	65280952	65280961	chr10	REEP3 (-166)
R.951	70320051	70320734	chr10	TET1 (-20)
R.952	70586994	70587181	chr10	STOX1 (-210)
R.953	70660528	70660701	chr10	DDX50 (-419)
R.955	72431919	72432482	chr10	ADAMTS14 (-358)
R.956	74020428	74021022	chr10	ASCC1 (-44692), DDIT4 (-12953)
R.957	74034644	74034667	chr10	DDIT4 (+978)
R.959	81741554	81742245	chr10	TMEM254 (-96526), SFTPD (-33041)
R.960	88126285	88126306	chr10	GRID1 (-61)
R.961	88471309	88471978	chr10	BMPR1A (-44763), LDB3 (+43438)
R.963	91294781	91295045	chr10	SLC16A12 (+400)
R.964	91295358	91295421	chr10	SLC16A12 (-77)
R.965	93170467	93171002	chr10	HECTD2 (+633)

R.966	94833520	94833626	chr10	CYP26A1 (-74)
R.967	97515222	97515377	chr10	ENTPD1 (-109)
R.968	98273188	98273924	chr10	TLL2 (+112)
R.969	99185264	99185307	chr10	PGAM1 (-631)
R.970	100154826	100155182	chr10	LOXL4 (-126997), PYROXD2 (+19937)
R.971	102107666	102107757	chr10	SCD (+831)
R.972	102416179	102416409	chr10	PAX2 (-89174), HIF1AN (+120678)
R.973	102822249	102822873	chr10	KAZALD1 (+963)
R.974	103880676	103880810	chr10	LDB1 (-533)
R.975	103990214	103991057	chr10	ELOVL3 (+4551), PITX3 (+10595)
R.976	112836780	112837240	chr10	ADRA2A (+220)
R.978	115803805	115804095	chr10	ADRB1 (+144)
R.979	115934411	115934468	chr10	TDRD1 (-4589)
R.980	118031654	118032355	chr10	GFRA1 (+974)
R.981	118032872	118032905	chr10	GFRA1 (+90)
R.982	118765174	118765255	chr10	KIAA1598 (-127)
R.983	118927664	118928025	chr10	VAX1 (-30278), KCNK18 (-29155)
R.984	120925349	120925452	chr10	SFXN4 (-222)
R.985	121485064	121485184	chr10	INPP5F (-485)
R.986	124220856	124221066	chr10	HTRA1 (-80)
R.987	125852011	125852693	chr10	CHST15 (-382)
R.988	125852964	125853893	chr10	CHST15 (-1459)
R.989	126106555	126106614	chr10	OAT (+920)
R.990	126431523	126432368	chr10	FAM53B (+673)
R.991	128076936	128077080	chr10	ADAM12 (+16)
R.996	133835858	133836792	chr10	JAKMIP3 (-81850), BNIP3 (-40890)
R.997	133849797	133850441	chr10	JAKMIP3 (-68056), BNIP3 (-54684)
R.999	134121577	134121596	chr10	STK32C (-143)
R.1000	134756164	134756306	chr10	NKX6-2 (-156679), GPR123 (-145174)
R.1001	135191928	135191962	chr10	ECHS1 (-4752), PAOX (-793)
R.1002	135192038	135192102	chr10	ECHS1 (-4877), PAOX (-668)
R.1003	830190	830320	chr11	CD151 (-2588)
R.1004	1567941	1568614	chr11	MOB2 (-60302), DUSP8 (+24872)
R.1005	2158555	2159122	chr11	IGF2 (+3629), MRPL23 (+190331)
R.1006	2160554	2160564	chr11	IGF2 (+1909), MRPL23 (+192051)
R.1009	2422391	2422607	chr11	TRPM5 (+21776), CD81 (+23975)
R.1011	2905554	2906285	chr11	CDKN1C (+1191), KCNQ1 (+439699)
R.1012	2950403	2950754	chr11	PHLDA2 (+106)
R.1013	3663491	3663644	chr11	ART1 (-2793), ART5 (-83)
R.1014	5247586	5248426	chr11	HBB (+421)
R.1015	5265155	5265992	chr11	HBB (-17147), HBD (-9696), HBG1 (+5548)
R.1016	6440482	6440731	chr11	APBB1 (-307)
R.1017	10476976	10477461	chr11	AMPD3 (+4991), MTRNR2L8 (+53504)
R.1018	12030268	12030289	chr11	DKK3 (+350)
R.1019	12030648	12031046	chr11	DKK3 (-218)
R.1020	15132656	15133087	chr11	INSC (-1098)
R.1021	16626476	16627098	chr11	C11orf58 (-133161), SOX6 (-128852)
R.1022	16634515	16635104	chr11	SOX6 (-136875), C11orf58 (-125138)
R.1023	18720358	18720729	chr11	TMEM86A (+206)

R.1025	20409647	20410216	chr11	PRMT3 (+856)
R.1027	26353605	26353811	chr11	ANO3 (+737)
R.1028	30607360	30607888	chr11	MPPED2 (-5581), DCDC1 (+783697)
R.1029	30607959	30608165	chr11	MPPED2 (-6019), DCDC1 (+783259)
R.1030	31832710	31832931	chr11	PAX6 (+6688), ELP4 (+301524)
R.1031	31833007	31833016	chr11	PAX6 (+6497), ELP4 (+301715)
R.1033	32456069	32456912	chr11	WT1 (+685)
R.1034	32457124	32457386	chr11	WT1 (-79)
R.1036	33061716	33061907	chr11	TCP11L1 (+849)
R.1037	40314771	40315337	chr11	LRRC4C (+610)
R.1038	43963845	43964150	chr11	ACCSL (-105533), ALKBH3 (+61609)
R.1039	44327015	44327572	chr11	ALX4 (+4422), EXT2 (+209547)
R.1040	44331108	44331577	chr11	ALX4 (+373)
R.1041	44971049	44971894	chr11	TP53I11 (+239)
R.1043	45907069	45907147	chr11	MAPK8IP1 (-94)
R.1044	45921959	45922184	chr11	C11orf94 (+6761), MAPK8IP1 (+14870)
R.1045	46402210	46402535	chr11	MDK (+67)
R.1046	46413816	46414452	chr11	CHRM4 (-6027), AMBRA1 (+198780)
R.1047	57249947	57250780	chr11	RTN4RL2 (+22342), SLC43A1 (+32895)
R.1048	58939797	58939953	chr11	DTX4 (-90)
R.1049	60048221	60049097	chr11	MS4A4A (+600)
R.1051	61101089	61102074	chr11	DDB1 (-744), DAK (+900)
R.1052	61276048	61276447	chr11	PPP1R32 (+27656), SYT7 (+72050)
R.1053	62439523	62439866	chr11	C11orf83 (+1950), UBXN1 (+6832)
R.1054	62476891	62477109	chr11	BSCL2 (+46)
R.1055	62559850	62559882	chr11	TMEM223 (-373)
R.1056	62648823	62649160	chr11	SLC3A2 (+25409), CHRM1 (+40161)
R.1057	63997715	63997740	chr11	VEGFB (-4282), TRPT1 (-4002), DNAJC4 (-22)
R.1058	64512197	64512829	chr11	RASGRP2 (+415)
R.1059	64901065	64901879	chr11	SYVN1 (+532)
R.1060	65308245	65308645	chr11	SCYL1 (+15897), LTBP3 (+17254)
R.1061	66024688	66024911	chr11	KLC2 (+35)
R.1062	66034896	66034926	chr11	RAB1B (-1093)
R.1063	66045718	66046083	chr11	CNIH2 (+231)
R.1064	66138996	66139221	chr11	SLC29A2 (+182)
R.1065	66139311	66139712	chr11	SLC29A2 (-221)
R.1066	67034249	67034540	chr11	ADRBK1 (+514)
R.1067	67195993	67196506	chr11	RPS6KB2 (+278)
R.1068	67722745	67723411	chr11	ALDH3B2 (-280976), UNC93B1 (+48515)
R.1069	67777770	67777952	chr11	NDUFS8 (-20233), ALDH3B1 (+1813)
R.1070	67889051	67889237	chr11	CHKA (-473)
R.1071	68816067	68816191	chr11	TPCN2 (-236)
R.1072	69632335	69632655	chr11	FGF4 (-42324), FGF3 (+1297)
R.1073	71159853	71160219	chr11	NADSYN1 (-4119), DHCR7 (-555)
R.1075	75947361	75947637	chr11	WNT11 (-29923), PRKRIR (+144487)
R.1076	77184925	77185492	chr11	PAK1 (-104)
R.1077	78286037	78286346	chr11	NARS2 (-283)
R.1078	79151188	79151611	chr11	TENM4 (+295)

R.1079	79151791	79151814	chr11	TENM4 (-108)
R.1081	94822733	94823603	chr11	ENDOD1 (+194)
R.1082	100557736	100557996	chr11	ARHGAP42 (-541)
R.1083	107461798	107462430	chr11	ELMOD1 (+158)
R.1084	108093335	108093386	chr11	ATM (-198), NPAT (+8)
R.1085	108464350	108464417	chr11	EXPH5 (-10)
R.1086	110583599	110583662	chr11	ARHGAP20 (-180)
R.1087	111411176	111411216	chr11	LAYN (-188)
R.1088	111956827	111956885	chr11	ENSG00000255292 (-771), SDHD (-641), TIMM8B (+666)
R.1089	112832159	112833009	chr11	NCAM1 (+426)
R.1090	113185079	113185166	chr11	ANKK1 (-73390), NCAM1 (+352965)
R.1091	113345325	113346183	chr11	DRD2 (+357)
R.1092	113931000	113931305	chr11	ZBTB16 (+838)
R.1093	115375703	115375718	chr11	CADM1 (-578)
R.1094	117747390	117747829	chr11	FXYD6 (+287)
R.1095	119187772	119187858	chr11	MCAM (+11)
R.1096	119234736	119234956	chr11	C1QTNF5 (-17463), USP2 (+17590)
R.1097	122753429	122753504	chr11	CRTAM (+44259), BSX (+98961)
R.1098	122855292	122855462	chr11	BSX (-2949)
R.1099	123300839	123300867	chr11	CLMP (-234864), GRAMD1B (-95677)
R.1100	123524938	123525662	chr11	SCN3B (+12)
R.1101	126080928	126081181	chr11	RPUSD4 (+532)
R.1102	129245309	129245568	chr11	BARX2 (-396)
R.1103	129245691	129245728	chr11	BARX2 (-125)
R.1104	129939049	129939665	chr11	APLP2 (-444)
R.1105	130297513	130298172	chr11	ZBTB44 (-113262), ADAMTS8 (+1045)
R.1106	130318594	130318703	chr11	ADAMTS15 (-220)
R.1108	133826329	133827193	chr11	IGSF9B (+119)
R.1109	134145940	134146075	chr11	GLB1L3 (-627)
R.1110	134146253	134146324	chr11	GLB1L3 (-346)
R.1112	2161640	2162232	chr12	CACNA1C (-793)
R.1113	4382425	4382985	chr12	CCND2 (-233)
R.1114	4384404	4384890	chr12	C12orf5 (-45724), CCND2 (+1709)
R.1115	5018805	5018984	chr12	KCNA1 (-176)
R.1116	6976279	6976432	chr12	TPI1 (+73)
R.1117	7282186	7282371	chr12	RBP5 (-741), CLSTN3 (-516)
R.1118	7282593	7282928	chr12	RBP5 (-1223), CLSTN3 (-34)
R.1119	7342621	7342888	chr12	PEX5 (+472)
R.1120	10366005	10366566	chr12	GABARAPL1 (+882)
R.1121	12503186	12503194	chr12	MANSC1 (+285)
R.1123	15374303	15374609	chr12	RERG (-105)
R.1124	15475690	15476085	chr12	PTPRO (+557)
R.1125	19282374	19283108	chr12	PLEKHA5 (+39)
R.1126	21810983	21811034	chr12	LDHB (-281)
R.1127	22487459	22488229	chr12	ST8SIA1 (-196)
R.1128	24715484	24716048	chr12	SOX5 (-613129), BCAT1 (+340243)
R.1129	26277706	26278677	chr12	BHLHE41 (-132)
R.1130	28122232	28123034	chr12	PTHLH (+2270), KLHL42 (+189680)
R.1131	28123063	28123086	chr12	PTHLH (+1828), KLHL42 (+190122)

R.1132	28343237	28343518	chr12	CCDC91 (-1)
R.1133	29302035	29302279	chr12	FAR2 (-74441), CCDC91 (+958778)
R.1134	30848063	30848360	chr12	IPO8 (+708)
R.1136	31881951	31882290	chr12	H3F3C (+63054), METTL20 (+69499)
R.1137	32552899	32553273	chr12	FGD4 (-102008), BICD1 (+292923)
R.1138	38532352	38532614	chr12	ALG10B (-177897)
R.1140	44229713	44230058	chr12	TMEM117 (-15)
R.1141	45269318	45269519	chr12	NELL2 (+1461)
R.1142	45444902	45445115	chr12	DBX2 (-127)
R.1143	50451395	50451907	chr12	ASIC1 (+320)
R.1144	51785820	51786489	chr12	SLC4A8 (-32400), GALNT6 (-8808)
R.1145	52214119	52214758	chr12	ANKRD33 (-67305), SCN8A (+229419)
R.1148	52400650	52400907	chr12	GRASP (+55)
R.1150	54120876	54121077	chr12	CALCOCO1 (+247)
R.1151	54384744	54385526	chr12	HOXC9 (-8768), HOXC10 (+6165)
R.1152	54410491	54410657	chr12	HOXC6 (-11568), HOXC8 (+7742)
R.1153	54412269	54413000	chr12	HOXC6 (-9507), HOXC8 (+9803)
R.1154	54450584	54450826	chr12	HOXC4 (+3044), SMUG1 (+132073)
R.1155	56101579	56102034	chr12	ITGA7 (-322)
R.1156	57483513	57483793	chr12	NAB2 (+976)
R.1157	57610491	57610705	chr12	NXPH4 (+20)
R.1158	58026010	58026476	chr12	B4GALNT1 (+895)
R.1159	58132093	58132733	chr12	AGAP2 (-384)
R.1160	63544923	63545523	chr12	AVPR1A (-501)
R.1161	65515031	65515276	chr12	WIF1 (+192)
R.1162	70759870	70759920	chr12	KCNMB4 (-161)
R.1163	71833515	71833613	chr12	LGR5 (+14)
R.1164	75904963	75905004	chr12	KRR1 (+417)
R.1166	82752718	82753120	chr12	CCDC59 (-352), METTL25 (+643)
R.1167	89747417	89747628	chr12	DUSP6 (-475)
R.1168	90101389	90102059	chr12	ATP2B1 (+884)
R.1169	90102575	90103243	chr12	ATP2B1 (-301)
R.1170	93323225	93323273	chr12	EEA1 (-142)
R.1171	94071294	94071778	chr12	CRADD (+43)
R.1172	94541943	94542778	chr12	PLXNC1 (-138)
R.1173	94543764	94543978	chr12	PLXNC1 (+1372), CCDC41 (+309857)
R.1174	96336121	96336499	chr12	AMDHD1 (-761)
R.1175	96882885	96883596	chr12	NEDD1 (-418003), CDK17 (-88903)
R.1176	100377821	100378258	chr12	ANKS1B (-25)
R.1177	103889655	103889789	chr12	STAB2 (-91329), ASCL1 (+538258)
R.1178	104443925	104444006	chr12	GLT8D2 (-51)
R.1179	106695932	106695998	chr12	POLR3B (-55471), CKAP4 (-54199)
R.1180	107349093	107349191	chr12	C12orf23 (-358)
R.1181	108523463	108523888	chr12	WSCD2 (-1852)
R.1182	109592525	109592662	chr12	ACACB (+38194), FOXN4 (+154431)
R.1183	109747000	109747333	chr12	FOXN4 (-142)
R.1184	112280887	112280971	chr12	MAPKAPK5 (+499)
R.1188	116971031	116971116	chr12	MED13L (-255931), MAP1LC3B2 (-26112)
R.1189	117319246	117319395	chr12	HRK (-75)

R.1190	118809325	118809648	chr12	SUDS3 (-4698), TAOK3 (+1263)
R.1191	120426529	120426618	chr12	CCDC64 (-1099)
R.1192	120806383	120807316	chr12	MSI1 (+133)
R.1193	120875539	120875584	chr12	COX6A1 (-331)
R.1194	121163418	121163597	chr12	ACADS (-30)
R.1195	122687951	122688708	chr12	B3GNT4 (+240)
R.1196	124155880	124156170	chr12	TCTN2 (+365)
R.1197	124456542	124457083	chr12	ZNF664 (-857), CCDC92 (+558)
R.1199	125478775	125478868	chr12	BRI3BP (+576)
R.1200	125670513	125671104	chr12	TMEM132B (-140353), AACS (+120884)
R.1201	126018024	126018298	chr12	TMEM132B (+206999)
R.1202	129309117	129309330	chr12	SLC15A4 (-696)
R.1203	129337674	129337871	chr12	GLT1D1 (-308)
R.1204	132413775	132413985	chr12	PUS1 (+112)
R.1206	133066651	133066682	chr12	P2RX2 (-128736), MUC8 (-15941)
R.1207	133293889	133294524	chr12	PGAM5 (+6802), ANKLE2 (+44267)
R.1211	133532874	133532998	chr12	ZNF605 (-44)
R.1212	133614159	133614475	chr12	ZNF84 (+151)
R.1213	133656876	133657153	chr12	ZNF140 (+591)
R.1214	133707169	133707316	chr12	ENSG00000256825 (-327), ZNF891 (-184), ZNF10 (+65)
R.1215	20533018	20533551	chr13	ZMYM2 (+475)
R.1216	24153521	24154139	chr13	TNFRSF19 (+331)
R.1217	25254512	25254867	chr13	ATP12A (+141)
R.1218	26760422	26760793	chr13	SHISA2 (-135439), RNF6 (+36183)
R.1219	27334259	27335082	chr13	GPR12 (+251)
R.1220	28494161	28494497	chr13	PDX1 (+172)
R.1221	29293196	29293279	chr13	SLC46A3 (-131)
R.1222	37006063	37006116	chr13	CCNA1 (-405)
R.1223	39260877	39261432	chr13	FREM2 (-111)
R.1224	42535296	42535460	chr13	VWA8 (-158)
R.1225	44359836	44360259	chr13	ENOX1 (+996)
R.1226	44361212	44361624	chr13	ENOX1 (-374)
R.1227	48807050	48807122	chr13	ITM2B (-208)
R.1228	50159138	50159768	chr13	RCBTB1 (+266)
R.1229	51483857	51484248	chr13	RNASEH2B (+239)
R.1230	52585813	52586135	chr13	ALG11 (-576), ATP7B (-344)
R.1231	52733373	52733664	chr13	NEK3 (-1899)
R.1233	72441146	72441184	chr13	DACH1 (+165)
R.1234	73634056	73634369	chr13	KLF5 (+1283), KLF12 (+934973)
R.1235	75149833	75150072	chr13	KLF12 (-580767), TBC1D4 (+906297)
R.1236	78494010	78494067	chr13	EDNRB (-136)
R.1237	88324193	88324879	chr13	SLITRK5 (-334)
R.1238	92050791	92050991	chr13	GPC5 (-38)
R.1239	95363371	95363811	chr13	SOX21 (+798)
R.1240	95364586	95364675	chr13	SOX21 (-242)
R.1241	95364928	95365509	chr13	SOX21 (-830)
R.1242	98795021	98795562	chr13	FARP1 (-213)
R.1243	100620447	100621000	chr13	ZIC5 (+3439), CLYBL (+361801)

R.1244	100622570	100623362	chr13	ZIC5 (+1197), CLYBL (+364043)
R.1245	103450891	103450963	chr13	BIVM (-472), KDELC1 (+430)
R.1247	107187177	107187681	chr13	EFNB2 (+33)
R.1248	108519880	108520252	chr13	FAM155A (-983)
R.1249	108520481	108520827	chr13	FAM155A (-1571)
R.1250	111767003	111767899	chr13	ARHGEF7 (-173)
R.1251	112838611	112838970	chr13	SPACA7 (-191842), SOX1 (+116878)
R.1252	112859484	112860420	chr13	SPACA7 (-170681), SOX1 (+138039)
R.1254	114018021	114018864	chr13	GRTP1 (-2)
R.1255	115000206	115000234	chr13	CDC16 (-142)
R.1256	21100888	21101308	chr14	RNASE12 (-42116), OR6S1 (+8752)
R.1257	21466306	21467273	chr14	SLC39A2 (-659)
R.1259	22191382	22191827	chr14	OR4E2 (+58308), DAD1 (+866570)
R.1260	23305548	23305835	chr14	MMP14 (-74)
R.1261	23305957	23306850	chr14	MMP14 (+638)
R.1263	23821435	23821570	chr14	SLC22A17 (+577)
R.1264	24020586	24020989	chr14	THTPA (-4443), ZFHX2 (+70)
R.1265	24836084	24836219	chr14	NFATC4 (+35)
R.1266	27065974	27066771	chr14	NOVA1 (+587)
R.1267	34420413	34420437	chr14	EGLN3 (-138)
R.1268	36002798	36003118	chr14	INSM2 (-290)
R.1269	36003443	36003826	chr14	INSM2 (+387)
R.1270	50088260	50088598	chr14	RPL36AL (-1026), MGAT2 (+940)
R.1271	51027569	51027964	chr14	ATL1 (+1024), SAV1 (+107282)
R.1272	51560874	51561562	chr14	PYGL (-149764), TRIM9 (+1561)
R.1273	51562026	51562486	chr14	TRIM9 (+523)
R.1274	52535964	52536066	chr14	NID2 (-303)
R.1275	53173891	53174064	chr14	PSMC6 (+88)
R.1276	60557893	60558163	chr14	PCNXL4 (-601)
R.1277	64932086	64932467	chr14	AKAP5 (-1548)
R.1278	65346490	65346875	chr14	SPTB (-56817), CHURC1 (-34396)
R.1279	65438707	65438743	chr14	RAB15 (+150)
R.1280	66974032	66974163	chr14	GPHN (-27)
R.1281	66975372	66976062	chr14	FAM71D (-680429), GPHN (+1592)
R.1282	68086242	68086880	chr14	ARG2 (+46)
R.1283	69620129	69620440	chr14	DCAF5 (-442)
R.1284	70041283	70041661	chr14	KIAA0247 (-36841), PLEKHD1 (+90001)
R.1285	70346082	70346221	chr14	SMOC1 (+19)
R.1286	71275530	71275911	chr14	MAP3K9 (+530)
R.1287	75348325	75348400	chr14	DLST (-233)
R.1288	75894209	75894309	chr14	JDP2 (-4578)
R.1289	77499307	77499547	chr14	IRF2BPL (-4393)
R.1290	77606990	77607975	chr14	ZDHHC22 (+651)
R.1291	79745403	79745579	chr14	NRXN3 (+875398), DIO2 (+932352)
R.1293	81421508	81421763	chr14	TSHR (+249)
R.1294	88792913	88793021	chr14	KCNK10 (-3374)
R.1295	89882896	89883534	chr14	FOXN3 (+202259), TTC8 (+592209)
R.1296	90167851	90168304	chr14	FOXN3 (-82604), EFCAB11 (+252955)
R.1297	90527387	90527917	chr14	KCNK13 (-457)
R.1298	92414253	92414425	chr14	FBLN5 (-172)

R.1299	94254726	94255493	chr14	PRIMA1 (-283)
R.1300	95236568	95236615	chr14	GSC (-30)
R.1301	96505296	96505502	chr14	C14orf132 (-262)
R.1302	96505722	96506401	chr14	C14orf132 (+401)
R.1304	96670911	96670960	chr14	BDKRB2 (-199)
R.1305	96671037	96671262	chr14	BDKRB2 (+15)
R.1308	103589028	103589780	chr14	TNFAIP2 (-394)
R.1310	104604177	104604893	chr14	KIF26A (-525)
R.1311	104640581	104641403	chr14	C14orf144 (-69549), KIF26A (+35932)
R.1313	105154689	105154858	chr14	INF2 (-1200)
R.1317	32163153	32163262	chr15	OTUD7A (-215666), CHRNA7 (-159483)
R.1318	34875825	34875937	chr15	GOLGA8B (-110)
R.1319	37394166	37394754	chr15	MEIS2 (-2374)
R.1320	40330586	40330739	chr15	SRP14 (+726)
R.1321	40650133	40650169	chr15	DISP2 (-285)
R.1322	40697959	40698367	chr15	IVD (+167)
R.1323	41166042	41166443	chr15	RHOV (+244)
R.1324	41220951	41221939	chr15	DLL4 (-146)
R.1325	41576691	41576837	chr15	OIP5 (+48055), CHP1 (+53423)
R.1326	43621948	43622808	chr15	ADAL (-494), LCMT2 (+425)
R.1327	48009623	48009656	chr15	SEMA6D (-1046)
R.1328	52043486	52043553	chr15	TMOD2 (-238)
R.1329	52043684	52043951	chr15	TMOD2 (+60)
R.1330	52264133	52264396	chr15	LEO1 (-262)
R.1331	53082249	53082902	chr15	ONECUT1 (-367)
R.1332	59730071	59730655	chr15	GCNT3 (-173620), MYO1E (-65264)
R.1333	60884719	60884785	chr15	NARG2 (-113408), RORA (+34910)
R.1334	64443709	64443892	chr15	SNX22 (-115)
R.1335	64443970	64444268	chr15	SNX22 (+203)
R.1336	65067696	65068371	chr15	RBPMS2 (-248)
R.1337	65669563	65670304	chr15	IGDCC3 (+444)
R.1338	66545863	66546648	chr15	MEGF11 (-171)
R.1339	66585582	66585646	chr15	DIS3L (-271)
R.1340	68176973	68177334	chr15	PIAS1 (-169363), SKOR1 (+65112)
R.1341	68569728	68569993	chr15	FEM1B (-280)
R.1342	68870979	68871866	chr15	CORO2B (-150)
R.1343	69452462	69452723	chr15	GLCE (-380)
R.1344	69591148	69591194	chr15	PAQR5 (-15571), GLCE (+138198)
R.1345	70392497	70392993	chr15	TLE3 (-2230)
R.1346	72612125	72612853	chr15	CELF6 (-19)
R.1347	72667883	72668275	chr15	HEXA (+738)
R.1348	72668413	72668657	chr15	HEXA (+282)
R.1349	73075372	73075587	chr15	ADPGK (+646)
R.1350	73089234	73089643	chr15	NEO1 (-254612), ADPGK (-13313)
R.1351	74045075	74045705	chr15	TBC1D21 (-120578), CD276 (+68836)
R.1352	74725593	74726139	chr15	SEMA7A (+942)
R.1353	74753869	74753978	chr15	ARID3B (-79594), SEMA7A (-27116)
R.1354	75199472	75199493	chr15	MPI (+17135), COX5A (+31026)
R.1355	75287824	75287854	chr15	SCAMP5 (-100)

R.1356	76351361	76351942	chr15	C15orf27 (-526)
R.1357	78423758	78424141	chr15	CIB2 (-64)
R.1358	79383385	79383632	chr15	RASGRF1 (-394)
R.1359	80189322	80189782	chr15	MTHFS (-161)
R.1360	80215376	80216052	chr15	ST20-MTHFS (+372)
R.1361	80216276	80216491	chr15	ST20-MTHFS (-298)
R.1362	80543983	80544661	chr15	ARNT2 (-152371), FAH (+99200)
R.1363	81072152	81072933	chr15	KIAA1199 (+831)
R.1364	82337202	82337812	chr15	MEX3B (+975)
R.1365	83378827	83378895	chr15	AP3B2 (-195)
R.1366	83952345	83952722	chr15	BNC1 (+932)
R.1367	84115932	84116151	chr15	SH3GL3 (+62)
R.1368	84322323	84322594	chr15	ADAMTSL3 (-379)
R.1369	85923708	85923739	chr15	AKAP13 (-236)
R.1370	89902526	89903506	chr15	POLG (-24938), RHCG (+136828)
R.1371	90197924	90198503	chr15	KIF7 (+468)
R.1372	90233762	90233889	chr15	WDR93 (-202), PEX11A (+187)
R.1373	90234026	90234207	chr15	PEX11A (-104), WDR93 (+89)
R.1374	90727995	90728020	chr15	IDH2 (-82272), SEMA4B (-16543)
R.1375	90728140	90728569	chr15	IDH2 (-82619), SEMA4B (-16196)
R.1376	91643036	91643135	chr15	SV2B (-429)
R.1377	92396240	92396808	chr15	SLCO3A1 (-401)
R.1378	93122918	93123359	chr15	FAM174B (+76049), ST8SIA2 (+186081)
R.1379	93615146	93615718	chr15	RGMA (+1660), CHD2 (+172374)
R.1380	98447577	98447696	chr15	ARRDC4 (-56291)
R.1381	98503725	98503803	chr15	ARRDC4 (-164)
R.1382	100882231	100882540	chr15	ADAMTS17 (-176)
R.1383	100913842	100913948	chr15	ADAMTS17 (-31685), CERS3 (+171030)
R.1384	101835872	101836060	chr15	SNRPA1 (-510)
R.1385	433439	433641	chr16	TMEM8A (-1558)
R.1386	677526	677895	chr16	WFIKKN1 (-3221)
R.1387	710674	711015	chr16	RHOT2 (-7241), WFIKKN1 (+29913)
R.1388	729354	729763	chr16	STUB1 (-717)
R.1389	1203586	1203595	chr16	CACNA1H (+350)
R.1390	1821559	1822289	chr16	EME2 (-1284), NME3 (-193)
R.1391	1831722	1832493	chr16	NUBP2 (-794), SPSB3 (+713)
R.1392	1833420	1833616	chr16	SPSB3 (-697), NUBP2 (+616)
R.1393	1876114	1876867	chr16	FAHD1 (-477), HAGH (+704)
R.1394	1993481	1993659	chr16	MSRB1 (-243)
R.1395	2012763	2013573	chr16	RNF151 (-3707), RPS2 (+1693)
R.1396	2040017	2040627	chr16	SYNGR3 (+376)
R.1397	2478353	2478800	chr16	CCNF (-818)
R.1398	2770814	2770901	chr16	PRSS27 (-642)
R.1399	3233117	3233912	chr16	OR1F1 (-20732), CASP16 (+39271)
R.1400	3313947	3314612	chr16	ZNF263 (-18663), MEFV (-7653)
R.1401	3355079	3355553	chr16	ZNF75A (-170), TIGD7 (+329)
R.1403	4421603	4422228	chr16	VASN (+67)
R.1404	10276799	10277017	chr16	GRIN2A (-297)
R.1405	11680284	11680980	chr16	LITAF (+787)

R.1406	15950840	15951230	chr16	MYH11 (-167)
R.1407	17564920	17565189	chr16	XYLT1 (-317)
R.1408	19179231	19179258	chr16	SYT17 (-326)
R.1409	20817602	20817639	chr16	ENSG00000005189 (-200), ERI2 (+174)
R.1410	21289767	21289818	chr16	CRYM (+24579), ANKS4B (+44807)
R.1411	23464705	23464715	chr16	COG7 (-209)
R.1412	28074974	28075269	chr16	GSG1L (-292)
R.1413	29820119	29820460	chr16	PRRT2 (-3222)
R.1414	29973024	29973158	chr16	TMEM219 (-907)
R.1415	30006300	30007069	chr16	INO80E (+70)
R.1416	30103511	30103624	chr16	TBX6 (-363)
R.1417	30772531	30772838	chr16	RNF40 (-633)
R.1418	31884589	31884740	chr16	ZNF267 (-414)
R.1419	48278654	48279428	chr16	LONP2 (+834)
R.1420	49891857	49892496	chr16	CNEP1R1 (-166144), ZNF423 (-35527)
R.1421	50100517	50100815	chr16	PAPD5 (-86163), CNEP1R1 (+42345)
R.1422	50581723	50582056	chr16	NKD1 (-351)
R.1423	54319242	54320230	chr16	IRX3 (+939)
R.1424	54320430	54320674	chr16	IRX3 (+123)
R.1425	55512806	55513148	chr16	MMP2 (+94)
R.1426	55514269	55514470	chr16	LPCAT2 (-28540), MMP2 (+1487)
R.1427	57318603	57318709	chr16	PLLP (-57)
R.1428	57570661	57570777	chr16	DOK4 (-50312), GPR114 (-5882)
R.1430	58059152	58059321	chr16	MMP15 (-233)
R.1431	58060336	58060865	chr16	MMP15 (+1131), C16orf80 (+102753)
R.1432	58497714	58497815	chr16	NDRG4 (-163)
R.1433	58548873	58549679	chr16	SETD6 (-116)
R.1434	65155141	65155841	chr16	CDH11 (+610)
R.1435	67193015	67193218	chr16	HSF4 (-4171), FBXL8 (-717)
R.1436	67876451	67877413	chr16	NUTF2 (-3703), THAP11 (+719)
R.1437	67918680	67918813	chr16	NRN1L (+39)
R.1438	68056774	68056849	chr16	DUS2L (-47), DDX28 (+958)
R.1439	68278871	68278969	chr16	PLA2G15 (-327)
R.1440	68482637	68482821	chr16	SMPD3 (-138)
R.1441	68572937	68573240	chr16	ZFP90 (-27)
R.1442	72698400	72698852	chr16	PMFBP1 (-492472), ZFHX3 (+383648)
R.1443	72699054	72699106	chr16	PMFBP1 (-492926), ZFHX3 (+383194)
R.1444	74808678	74808761	chr16	FA2H (+3)
R.1445	81111074	81111226	chr16	C16orf46 (-301)
R.1446	82068702	82068980	chr16	HSD17B2 (+4)
R.1447	82204167	82204401	chr16	MPHOSPH6 (-453)
R.1448	84732969	84733199	chr16	USP10 (-500)
R.1449	85646973	85647585	chr16	GSE1 (+457)
R.1450	85832271	85832335	chr16	COX4I1 (-996), EMC8 (+843)
R.1451	86543092	86543567	chr16	FOXF1 (-803)
R.1452	87635674	87636539	chr16	JPH3 (-404)
R.1455	88636102	88636366	chr16	ZC3H18 (-555)
R.1456	88729968	88730440	chr16	MVD (-686)
R.1457	88753010	88753119	chr16	SNAI3 (-164)
R.1458	89557737	89557752	chr16	ANKRD11 (-776)

R.1459	89642413	89642613	chr16	CPNE7 (+337)
R.1460	89724782	89725091	chr16	CHMP1A (-684), SPATA33 (+727)
R.1461	89752555	89752879	chr16	CDK10 (-359)
R.1462	89983766	89984268	chr16	TUBB3 (-1556), MC1R (-270)
R.1463	881647	881949	chr17	GLOD4 (-196217), NXN (+1212)
R.1464	1928794	1929555	chr17	DPH1 (-4229), RTN4RL1 (-997)
R.1465	1945076	1945138	chr17	OVCA2 (-220)
R.1466	3540328	3540440	chr17	SHPK (-768), CTNS (+611)
R.1467	3867794	3867847	chr17	ATP2A3 (-236)
R.1468	4401911	4402132	chr17	SPNS2 (-111)
R.1469	4642905	4643257	chr17	ZMYND15 (-733), CXCL16 (+33)
R.1470	5404330	5404581	chr17	MIS12 (+14209), NLRP1 (+82821)
R.1471	6917393	6917757	chr17	ENSG00000215067 (-1939), RNASEK-C17orf49 (-498), C17orf49 (-239)
R.1473	7197987	7198000	chr17	YBX2 (-111)
R.1474	7209803	7210162	chr17	EIF5A (-335)
R.1475	8055733	8055834	chr17	PER1 (-31)
R.1476	8534404	8534932	chr17	MYH10 (-589)
R.1477	8924776	8925203	chr17	NTN1 (+131)
R.1478	9862873	9862900	chr17	RCVRN (-53949), GAS7 (+238981)
R.1479	11924870	11925041	chr17	MAP2K4 (+815)
R.1480	12569095	12569114	chr17	MYOCD (-201)
R.1481	12569213	12569334	chr17	MYOCD (-32)
R.1482	12692673	12692900	chr17	ARHGAP44 (-69)
R.1483	14204310	14204485	chr17	HS3ST3B1 (-2)
R.1484	15602714	15602880	chr17	ENSG00000187607 (-294), ZNF286A (-258)
R.1485	15848072	15848828	chr17	ADORA2B (+219)
R.1486	16472440	16472693	chr17	ZNF287 (-84)
R.1488	20811255	20812114	chr17	USP22 (+134667), CDRT15L2 (+328648)
R.1489	21118194	21118261	chr17	TMEM11 (-291)
R.1490	27894761	27895566	chr17	ABHD15 (-1009), TP53I13 (-498)
R.1491	28706427	28706715	chr17	CPD (+648)
R.1492	30592657	30593452	chr17	RHBDL3 (-140)
R.1493	31254875	31255268	chr17	TMEM98 (+117)
R.1494	31619592	31620378	chr17	ASIC2 (+21)
R.1495	32907636	32907705	chr17	TMEM132E (-97)
R.1496	33289325	33289464	chr17	CCT6B (-867), ZNF830 (+846)
R.1497	33701321	33701529	chr17	SLFN11 (-786)
R.1498	35060323	35060467	chr17	LHX1 (-233689), MRM1 (+102394)
R.1499	35294481	35294740	chr17	LHX1 (+527)
R.1500	35295726	35296219	chr17	AATF (-10202), LHX1 (+1889)
R.1501	37763000	37763735	chr17	NEUROD2 (+828)
R.1502	37764250	37764293	chr17	NEUROD2 (-76)
R.1503	39890581	39891007	chr17	HAP1 (+102)
R.1504	39942812	39942981	chr17	JUP (+53)
R.1505	42084612	42084953	chr17	PYY (-2946), NAGS (+2869)
R.1506	42298359	42298664	chr17	UBTF (+482)
R.1507	42385525	42385946	chr17	RUNDC3A (-191)

R.1508	46623780	46624268	chr17	HOXB2 (-583)
R.1509	46673910	46674395	chr17	HOXB5 (-2830)
R.1510	46703122	46703908	chr17	HOXB9 (+324)
R.1511	46724237	46724775	chr17	HOXB9 (-20667), PRAC (+75378)
R.1512	47572610	47573230	chr17	NGFR (+265)
R.1513	47652804	47653212	chr17	NXPH3 (-212)
R.1514	47925364	47926239	chr17	TAC4 (-423)
R.1515	48046663	48046859	chr17	DLX4 (+223)
R.1516	48637649	48637818	chr17	CACNA1G (-1087)
R.1517	52977867	52978583	chr17	TOM1L1 (+351)
R.1518	53342602	53342740	chr17	HLF (+298)
R.1519	56245848	56246650	chr17	OR4D2 (-768)
R.1520	56609082	56609582	chr17	MTMR4 (-14066), SEPT4 (+8847)
R.1521	56833425	56834061	chr17	PPM1E (+513)
R.1522	59477019	59477081	chr17	TBX2 (-207)
R.1523	59477266	59477564	chr17	TBX2 (+158)
R.1524	60704543	60705058	chr17	MRC2 (+39)
R.1525	61042898	61043343	chr17	MARCH10 (-157426), TANC2 (-43796)
R.1527	67410790	67410806	chr17	MAP2K6 (-41)
R.1528	71640338	71640353	chr17	SDK2 (-118)
R.1529	72209156	72209469	chr17	TTYH2 (-340)
R.1531	72450232	72450692	chr17	CD300A (-12093), GPRC5C (+23410)
R.1532	72732955	72733163	chr17	RAB37 (+3)
R.1533	73127197	73127468	chr17	NT5C (+557)
R.1534	73257286	73257657	chr17	MRPS7 (-283), GGA3 (+413)
R.1537	74137336	74137522	chr17	FOXJ1 (-49)
R.1538	74721824	74721931	chr17	METTL23 (-1072), JMJD6 (+883)
R.1539	74928737	74928833	chr17	SEC14L1 (-156046), MGAT5B (+60056)
R.1541	76921829	76921948	chr17	TIMP2 (-420)
R.1542	77751069	77751135	chr17	CBX2 (-829)
R.1543	77773808	77774124	chr17	CBX8 (-3051)
R.1544	78518857	78519192	chr17	RPTOR (-43)
R.1545	78803474	78804436	chr17	CHMP6 (-161686), RPTOR (+284887)
R.1546	79042364	79043264	chr17	BAIAP2 (+33852), AATK (+97003)
R.1549	79317339	79317944	chr17	ENSG00000171282 (-55898), TMEM105 (-13168)
R.1550	80710066	80710673	chr17	TBCD (+430)
R.1551	5628468	5629371	chr18	EPB41L3 (-84679), TMEM200C (+267034)
R.1552	5629683	5630016	chr18	EPB41L3 (-85609), TMEM200C (+266104)
R.1553	5891948	5892213	chr18	EPB41L3 (-347840), TMEM200C (+3873)
R.1554	6413908	6414446	chr18	L3MBTL4 (+733)
R.1555	11689206	11689613	chr18	GNAL (+455)
R.1556	13801143	13801271	chr18	MC5R (-24336), RNMT (+74547)
R.1557	18822123	18822817	chr18	ROCK1 (-130658), GREB1L (-121084)
R.1559	20139442	20139913	chr18	RBBP8 (-374111), CTAGE1 (-141800)
R.1560	21718893	21718979	chr18	CABYR (-6)
R.1561	24443262	24443344	chr18	KCTD1 (-313904), AQP4 (+2479)

R.1562	28681962	28682408	chr18	DSC2 (+193)
R.1563	30050326	30050818	chr18	GAREM (-177)
R.1564	30051012	30051183	chr18	GAREM (-703)
R.1565	31158497	31158549	chr18	ASXL3 (-73)
R.1566	32556760	32557266	chr18	MAPRE2 (-64311), DTNA (+383710)
R.1567	32956850	32957337	chr18	ZNF396 (+207)
R.1568	33877806	33878230	chr18	FHOD3 (+341)
R.1569	35145535	35146046	chr18	CELF4 (+209)
R.1571	42260110	42260134	chr18	SETBP1 (-741)
R.1572	43754049	43755000	chr18	C18orf25 (+532)
R.1573	48086162	48086580	chr18	MAPK4 (-77)
R.1574	56530106	56530420	chr18	ZNF532 (+431)
R.1575	60988017	60988058	chr18	BCL2 (-677)
R.1577	67955861	67955882	chr18	SOCS6 (-265)
R.1578	70211774	70211916	chr18	CBLN2 (-71)
R.1579	74202887	74203484	chr18	ZNF516 (+3960)
R.1580	74206892	74207551	chr18	ZNF516 (-76)
R.1581	74843361	74844034	chr18	MBP (+1027), ZNF236 (+307582)
R.1582	75612056	75612223	chr18	GALR1 (+649635)
R.1583	77154869	77155145	chr18	NFATC1 (-5329), ATP9B (+325613)
R.1584	77160030	77160758	chr18	NFATC1 (+58)
R.1585	78004728	78005237	chr18	PARD6G (+446)
R.1586	78005607	78005665	chr18	PARD6G (-207)
R.1587	408683	409510	chr19	THEG (-33084), SHC2 (+51899)
R.1588	460135	460934	chr19	SHC2 (+461)
R.1589	639566	639808	chr19	FGF22 (-208)
R.1590	821865	822195	chr19	ENSG00000129951 (-78)
R.1591	860565	861071	chr19	CFD (+1365), MED16 (+32400)
R.1592	1028379	1028780	chr19	ABCA7 (-11522), CNN2 (+2282)
R.1593	3606835	3606974	chr19	TBXA2R (-247)
R.1595	4172961	4173482	chr19	SIRT6 (+9379), CREB3L3 (+19624)
R.1596	4769531	4769690	chr19	DPP9 (-45736), FEM1A (-22117)
R.1597	5048426	5048814	chr19	KDM4B (+79488), PTPRS (+292194)
R.1598	8273917	8274460	chr19	CERS4 (-48)
R.1599	9546252	9546371	chr19	ZNF266 (-14891), ZNF560 (+62971)
R.1600	9879317	9879603	chr19	ZNF846 (-167)
R.1601	10047034	10047209	chr19	OLFM2 (+106)
R.1602	10381393	10381454	chr19	ICAM1 (-87)
R.1603	10527199	10527588	chr19	PDE4A (-3937)
R.1604	10530736	10531434	chr19	PDE4A (-246)
R.1605	10589815	10590009	chr19	KEAP1 (+24505), PDE4A (+58581)
R.1606	11266792	11267029	chr19	SPC24 (-427)
R.1607	11805298	11805899	chr19	ZNF823 (+44189), ZNF627 (+97364)
R.1608	11877721	11878248	chr19	ZNF441 (+78)
R.1609	11925087	11925456	chr19	ZNF440 (+173)
R.1610	12035959	12036118	chr19	ENSG00000267179 (+149), ZNF700 (+156)
R.1611	12146629	12146696	chr19	ZNF433 (-138)
R.1612	12267676	12267734	chr19	ZNF625 (-161), ZNF625-ZNF20 (-159)
R.1613	12273814	12274129	chr19	ZNF136 (+93)

R.1614	12405427	12405570	chr19	ZNF44 (+129)
R.1615	12405748	12405873	chr19	ZNF44 (-183)
R.1616	12444273	12444484	chr19	ZNF563 (+123)
R.1617	12511563	12512226	chr19	ZNF799 (+190), ENSG00000268744 (+193)
R.1618	12551140	12551931	chr19	ENSG00000268870 (+360), ZNF443 (+390)
R.1619	12595615	12595661	chr19	ENSG00000269755 (-8), ZNF709 (+5)
R.1620	12780830	12781085	chr19	MAN2B1 (-3402), ENSG00000269590 (-747)
R.1622	13002357	13002493	chr19	KLF1 (-4430), GCDH (+585)
R.1624	15490804	15490914	chr19	AKAP8 (-256)
R.1625	15619224	15619355	chr19	CYP4F22 (-14)
R.1626	16682354	16682861	chr19	SLC35E1 (+585)
R.1627	17326059	17326432	chr19	USE1 (+78)
R.1628	17414337	17414379	chr19	MRPL34 (-2119), ABHD8 (-76)
R.1629	17448497	17449042	chr19	ANO8 (-3132), GTPBP3 (+406)
R.1630	18118079	18118880	chr19	ARRDC2 (-497)
R.1631	18547358	18548015	chr19	ISYNA1 (+1424), SSBP4 (+17448)
R.1632	18717638	18717742	chr19	TMEM59L (-550), CRLF1 (-30)
R.1633	18746981	18746992	chr19	CRTC1 (-47501), TMEM59L (+28747)
R.1634	19322563	19322676	chr19	NCAN (-218)
R.1635	19335919	19336240	chr19	NCAN (+13242), HAPLN4 (+37525)
R.1636	19843456	19843934	chr19	ZNF14 (+211)
R.1637	19887246	19887486	chr19	ZNF14 (-43460), ZNF506 (+45194)
R.1638	21106053	21106370	chr19	ZNF85 (+118)
R.1639	21579787	21579862	chr19	ZNF493 (-106)
R.1640	21949968	21950216	chr19	ZNF100 (+338)
R.1641	23578063	23578292	chr19	ZNF91 (+184)
R.1642	23869502	23870151	chr19	ZNF675 (+177)
R.1643	28285128	28285395	chr19	NONE
R.1644	31840737	31841161	chr19	TSHZ3 (-496)
R.1645	33864279	33864421	chr19	CEBPG (+114)
R.1646	34113214	34113449	chr19	PEPD (-100632), CHST8 (-62102)
R.1647	35263701	35264069	chr19	ZNF599 (+235)
R.1648	35264158	35264189	chr19	ZNF599 (-54)
R.1649	35521622	35521856	chr19	SCN1B (+14)
R.1650	36048757	36049123	chr19	ATP4A (+5620), TMEM147 (+12443)
R.1651	36347626	36347919	chr19	KIRREL2 (-51)
R.1652	36486437	36486874	chr19	SDHAF1 (+566)
R.1653	36822549	36822672	chr19	ZNF565 (-116628), ZFP14 (+47490)
R.1654	36980503	36980699	chr19	ZNF566 (-138)
R.1655	37019165	37019327	chr19	ZNF260 (+316)
R.1656	37064156	37064171	chr19	ZNF529 (-10)
R.1657	37157565	37157721	chr19	ZNF461 (+96)
R.1658	37178174	37178489	chr19	ZNF567 (-23810), ZNF461 (-20593)
R.1659	37329366	37329431	chr19	ZNF790 (-113)
R.1660	37329475	37329500	chr19	ZNF790 (-202)
R.1661	37568953	37569236	chr19	ZNF420 (-287)
R.1662	37708675	37709434	chr19	ZNF383 (-127)

R.1663	37861707	37862220	chr19	ZNF527 (-99)
R.1664	37957995	37958444	chr19	ZNF570 (-1762), ZNF569 (+119)
R.1665	38085706	38085757	chr19	ZNF571 (-60)
R.1666	38865049	38865080	chr19	PSMD8 (-111)
R.1667	41036236	41036303	chr19	SHKBP1 (-46487), SPTBN4 (+64122)
R.1669	44099044	44099413	chr19	ZNF576 (-1528)
R.1670	44143644	44144149	chr19	CADM4 (+94)
R.1671	45908262	45908440	chr19	CD3EAP (-1116)
R.1672	46405119	46405552	chr19	MYPOP (+526)
R.1673	46580441	46580807	chr19	IGFL4 (-36350), IGFL3 (+47307)
R.1674	48897005	48897863	chr19	KDELR1 (-2624), GRIN2D (-698)
R.1675	49016928	49017364	chr19	LMTK3 (-700)
R.1676	49224033	49224454	chr19	MAMSTR (-1266)
R.1677	49250180	49250561	chr19	IZUMO1 (-205)
R.1678	49436756	49436967	chr19	DHDH (-77)
R.1679	49944488	49944642	chr19	SLC17A7 (+243)
R.1680	50922190	50922484	chr19	SPIB (+139), SPIB (+142)
R.1681	52207353	52207653	chr19	SIGLEC14 (-57449), HAS1 (+19718)
R.1682	52430334	52430492	chr19	ZNF613 (-60)
R.1683	52511370	52511504	chr19	ZNF615 (+12)
R.1684	52511513	52511591	chr19	ZNF615 (-103)
R.1685	53606340	53607009	chr19	ZNF160 (+12)
R.1686	53836802	53836866	chr19	ZNF845 (-168)
R.1687	53837180	53837561	chr19	ZNF845 (+369)
R.1688	55691175	55691742	chr19	SYT5 (+350)
R.1689	55880593	55880984	chr19	IL11 (+952)
R.1690	56061334	56061348	chr19	ENSG00000231274 (-4432)
R.1691	56154165	56154382	chr19	ZNF580 (+856)
R.1692	56915522	56915658	chr19	ZNF583 (-111)
R.1693	57702793	57703364	chr19	ZNF264 (+211)
R.1694	57792200	57792257	chr19	ZNF460 (+376)
R.1695	57862442	57862484	chr19	ZNF304 (-212)
R.1696	57862627	57863057	chr19	ZNF304 (+167)
R.1697	57901421	57901745	chr19	ENSG00000269533 (+207), ZNF548 (+318)
R.1699	58090222	58090287	chr19	ZNF416 (+40)
R.1700	58090397	58090465	chr19	ZNF416 (-136)
R.1701	58111128	58111480	chr19	ZNF530 (+51)
R.1702	58125438	58125867	chr19	ZNF134 (+34)
R.1703	58193144	58193323	chr19	ENSG00000269026 (-179), ZNF551 (-113)
R.1704	58193370	58193630	chr19	ENSG00000269026 (+87), ZNF551 (+153)
R.1706	58257853	58258092	chr19	ZNF776 (-191)
R.1707	58258172	58258646	chr19	ZNF776 (+245)
R.1708	58361140	58361533	chr19	ZNF587 (+112)
R.1709	58427757	58428106	chr19	ZNF417 (+12)
R.1711	58789915	58790422	chr19	ZNF8 (-148)
R.1712	58874386	58874501	chr19	ZNF497 (-324)
R.1713	59086638	59086703	chr19	MZF1 (-1729)

R.1714	327607	328035	chr20	NRSN2 (+153)
R.1715	825155	825268	chr20	FAM110A (+10792), ANGPT4 (+71748)
R.1716	1757780	1758016	chr20	SIRPG (-119473), SIRPA (-118044)
R.1717	3154266	3154899	chr20	ENSG00000088899 (-5376), DDRGK1 (+30748)
R.1718	5093988	5094433	chr20	TMEM230 (-478)
R.1719	10199536	10200189	chr20	SNAP25 (+385)
R.1720	10413855	10414372	chr20	SLX4IP (-1837), MKKS (-1544)
R.1721	13201353	13201844	chr20	ISM1 (-819)
R.1722	13202225	13202702	chr20	ISM1 (+46)
R.1723	16553772	16554047	chr20	KIF16B (+168)
R.1724	16554249	16554259	chr20	KIF16B (-176)
R.1725	16555519	16555778	chr20	KIF16B (-1571)
R.1726	17207246	17207562	chr20	PCSK2 (-232)
R.1727	18037540	18038510	chr20	OVOL2 (+496)
R.1728	19193060	19193824	chr20	SLC24A3 (+152)
R.1729	19955436	19955868	chr20	NAA20 (-42108), RIN2 (+88487)
R.1730	20349241	20349621	chr20	INSM1 (+666)
R.1731	25038391	25038795	chr20	ACSS1 (+225)
R.1732	25176806	25177027	chr20	ENTPD6 (+561)
R.1733	25565460	25566336	chr20	NINL (+255)
R.1734	30605868	30606095	chr20	HCK (-34082), CCM2L (+7729)
R.1735	32254706	32255491	chr20	NECAB3 (+7115), CBFA2T2 (+104928)
R.1736	33104204	33104384	chr20	DYNLRB1 (+80)
R.1737	33460474	33460724	chr20	ACSS2 (-3818), GGT7 (+64)
R.1738	33865349	33865990	chr20	EIF6 (+7118), MMP24 (+51213)
R.1739	33871836	33872408	chr20	EIF6 (+666)
R.1740	34203707	34203797	chr20	SPAG4 (-62)
R.1741	35490815	35491355	chr20	DSN1 (-88931), SOGA1 (+1004)
R.1742	36531399	36531652	chr20	VSTM2L (+20)
R.1744	37590070	37590555	chr20	DHX35 (-665)
R.1745	39318705	39319326	chr20	MAFB (-1136)
R.1746	42543129	42543646	chr20	TOX2 (-104)
R.1747	43438809	43439400	chr20	RIMS4 (-193)
R.1748	43538527	43538769	chr20	PABPC1L (-55)
R.1749	44451667	44451783	chr20	TNNC2 (+4255), UBE2C (+10510)
R.1750	44803246	44803686	chr20	CD40 (+56555), CDH22 (+76868)
R.1751	44993182	44993233	chr20	SLC35C2 (-1395)
R.1752	45034743	45035259	chr20	ELMO2 (+255)
R.1754	51588785	51588860	chr20	TSHZ2 (-123)
R.1755	54919037	54919239	chr20	FAM210B (-14833), MC3R (+95350)
R.1756	55204593	55205571	chr20	TFAP2C (+724)
R.1757	55841342	55841888	chr20	BMP7 (+69)
R.1758	55841898	55842871	chr20	BMP7 (-701)
R.1759	56285388	56285701	chr20	PMEPA1 (-587)
R.1760	56964453	56964831	chr20	VAPB (+464)
R.1761	57616897	57617431	chr20	SLMO2 (+800)
R.1763	60795156	60795465	chr20	HRH3 (0)
R.1764	61733863	61734520	chr20	BIRC7 (-133043), BHLHE23 (-95805)
R.1765	62130111	62130512	chr20	EEF1A2 (+193)

R.1767	62289358	62289599	chr20	STMN3 (-4699), RTEL1-TNFRSF6B (-1277), RTEL1 (-165)
R.1768	62711129	62711523	chr20	RGS19 (-481)
R.1769	16437288	16437351	chr21	NRIP1 (+1)
R.1770	18884876	18885132	chr21	CXADR (+304)
R.1771	22370733	22370864	chr21	NCAM2 (+166)
R.1772	27011462	27012313	chr21	JAM2 (+304)
R.1773	33245091	33245908	chr21	HUNK (-128)
R.1774	33942142	33942312	chr21	TCP10L (+15616), EVA1C (+157538)
R.1775	34442377	34443010	chr21	OLIG1 (+244)
R.1776	34444245	34444382	chr21	IFNAR2 (-157965), OLIG1 (+1864)
R.1777	37692294	37692453	chr21	MORC3 (-113)
R.1778	40757899	40758325	chr21	SH3BGR (-65644), WRB (+5942)
R.1779	40759534	40759686	chr21	SH3BGR (-64146), WRB (+7440)
R.1780	43098516	43098901	chr21	TMPRSS2 (-218717), RIPK4 (+88557)
R.1781	43916159	43916444	chr21	RSPH1 (+162)
R.1782	45148332	45148694	chr21	PDXK (+9520), CSTB (+47813)
R.1783	47518068	47518998	chr21	COL6A2 (+522)
R.1784	17601381	17602259	chr22	IL17RA (+35971), CECR5 (+38347)
R.1785	20306749	20307397	chr22	DGCR6L (+530)
R.1786	22011682	22012035	chr22	PPIL2 (-8414), SDF2L1 (+15309)
R.1787	24256011	24256358	chr22	MIF (+19994), GSTT2B (+47188)
R.1788	24989054	24989335	chr22	GGT1 (-9973), SNRPD3 (+37724)
R.1789	25615317	25615355	chr22	CRYBB2 (-153)
R.1790	29601763	29601862	chr22	EMID1 (-27)
R.1791	31064067	31064577	chr22	DUSP18 (-445)
R.1792	35935982	35936722	chr22	RASD2 (-563)
R.1793	36424782	36425210	chr22	RBFOX2 (-523)
R.1794	38239793	38240259	chr22	EIF3L (-4849), ANKRD54 (+412)
R.1795	38851884	38852154	chr22	KCNJ4 (-814)
R.1797	39548131	39548389	chr22	CBX7 (+395)
R.1798	39746048	39746550	chr22	SYNGR1 (+298)
R.1800	42949810	42949946	chr22	SERHL2 (-47)
R.1801	43506465	43506531	chr22	BIK (-256)
R.1802	43506649	43506786	chr22	BIK (-36)
R.1803	44319424	44320173	chr22	PNPLA3 (+180)
R.1804	45064086	45065052	chr22	KIAA1644 (-355838), PRR5 (-8396)
R.1805	45097940	45097955	chr22	ARHGAP8 (-434), PRR5-ARHGAP8 (-407)
R.1806	45405333	45405621	chr22	PHF21B (+104)
R.1807	45636317	45636559	chr22	UPK3A (-44425), NUP50 (+76716)
R.1808	46663545	46663740	chr22	PKDREJ (-4424)
R.1810	50689915	50690152	chr22	HDAC10 (-220)
R.1811	50919938	50920013	chr22	ADM2 (-9)
R.1812	51038720	51039000	chr22	MAPK8IP2 (-271)
R.1813	51221675	51221736	chr22	RABL2B (+364)

Supplementary Table 6b. Hypermethylated DMRs under disturbed flow, and associated genes				
**Region**	**DMR start**	**DMR end**	**Chr**	**Gene**
R.51	43472373	43473030	chr1	SLC2A1 (-48201), EBNA1BP2 (+165539)
R.74	87994406	87995129	chr1	LMO4 (+200617)
R.117	179557960	179558954	chr1	TDRD5 (-2568)
R.130	210856989	210857205	chr1	HHAT (+354847), KCNH1 (+450218)
R.149	228473873	228473980	chr1	OBSCN (+78096), TRIM11 (+120614)
R.162	245836446	245836835	chr1	KIF26B (+518354), SMYD3 (+833878)
R.163	247276096	247276647	chr1	ZNF669 (-8698), ZNF124 (+58946)
R.168	8301459	8301539	chr2	ID2 (-517476)
R.169	8784813	8785728	chr2	ID2 (-33704)
R.173	11679605	11679879	chr2	GREB1 (+5500), NTSR2 (+130548)
R.191	44065791	44065893	chr2	ABCG8 (-261), ABCG5 (+116)
R.192	45029004	45029543	chr2	SIX3 (-139628), CAMKMT (+440171)
R.193	45164495	45164723	chr2	SIX3 (-4293)
R.198	56412380	56412924	chr2	EFEMP1 (-261378)
R.229	102768919	102769768	chr2	IL1RL2 (-34089), IL1R1 (+10098)
R.254	175499639	175500583	chr2	WIPF1 (-804)
R.288	234847247	234847683	chr2	SPP2 (-111878), TRPM8 (+21422)
R.294	242785116	242785270	chr2	PDCD1 (+15867), NEU4 (+33098)
R.296	9289747	9289893	chr3	RAD18 (-284634), SRGAP3 (+1243)
R.303	13462145	13462496	chr3	NUP210 (-512)
R.305	13679471	13679636	chr3	FBLN2 (+88923), WNT7A (+242064)
R.333	51976667	51977385	chr3	RRP9 (-1069), PARP3 (+651)
R.347	112013130	112013357	chr3	SLC9C1 (-139)
R.351	118864952	118865050	chr3	IGSF11 (-111266), UPK1B (-27364)
R.354	123339918	123340814	chr3	PTPLB (-36334), MYLK (+262783)
R.375	158786187	158786242	chr3	IQCJ-SCHIP1 (-838)
R.378	169780688	169781404	chr3	GPR160 (+25329), PHC3 (+118489)
R.398	3685577	3685875	chr4	LRPAP1 (-151440), ADRA2C (-82399)
R.405	7282819	7283184	chr4	SORCS2 (+88737), PSAPL1 (+153698)
R.425	87468529	87468672	chr4	MAPK10 (-187385), PTPN13 (-46867)
R.433	100737821	100738011	chr4	DAPP1 (-87)
R.443	133045114	133045178	chr4	NONE
R.466	5652502	5652728	chr5	ADAMTS16 (+512172), MED10 (+726092)
R.480	51813680	51813703	chr5	ITGA1 (-270038)
R.502	112769885	112770203	chr5	TSSK1B (+684)
R.521	139283051	139283199	chr5	PSD2 (+107719), NRG2 (+139754)
R.522	140166568	140167213	chr5	PCDHA2 (-7659), PCDHA1 (+1015)
R.528	140787430	140787507	chr5	PCDHGB6 (-301)
R.534	153852934	153853154	chr5	HAND1 (+4780), SAP30L (+27527)
R.540	169659845	169659863	chr5	LCP2 (+65377), FOXI1 (+126953)
R.579	28601324	28601365	chr6	SCAND3 (-46233), TRIM27 (+290421)
R.611	30859855	30860014	chr6	GTF2H4 (-16026), DDR1 (+8074)
R.614	31130261	31130273	chr6	CCHCR1 (-4701), TCF19 (+3948)
R.616	31549563	31549631	chr6	LTB (+605)
R.635	33868438	33869169	chr6	MLN (-97016), GRM4 (+232639)
R.640	41374806	41375153	chr6	FOXP4 (-139184), NCR2 (+71453)

R.699	166100605	166100693	chr6	PDE10A (-25092), SDIM1 (+209076)
R.703	168416740	168417126	chr6	KIF25 (+17161), FRMD1 (+62906)
R.704	168443212	168443958	chr6	FRMD1 (+36254), KIF25 (+43813)
R.705	168765896	168766243	chr6	SMOC2 (-75761), DACT2 (-45668)
R.708	170467425	170467790	chr6	DLL1 (+131953), C6orf70 (+315887)
R.709	170562191	170562522	chr6	DLL1 (+37204), C6orf70 (+410636)
R.711	752800	753007	chr7	PRKAR1B (-60)
R.712	1684706	1685134	chr7	TMEM184A (-88854), ELFN1 (-42835)
R.713	1936732	1937120	chr7	ELFN1 (+209171), MAD1L1 (+335952)
R.714	2100029	2100357	chr7	MAD1L1 (+172685), ELFN1 (+372438)
R.717	3083333	3083541	chr7	CARD11 (+142)
R.718	3991014	3991287	chr7	FOXK1 (-730789), SDK1 (+650071)
R.721	8475484	8476128	chr7	NXPH1 (+2221)
R.726	23749639	23749722	chr7	STK31 (-105)
R.734	36124843	36124944	chr7	EEPD1 (-67864), SEPT7 (+284267)
R.736	36765142	36765609	chr7	AOAH (-1223)
R.748	50443901	50444317	chr7	FIGNL1 (+73313), IKZF1 (+95791)
R.756	70254810	70255332	chr7	WBSCR17 (-342084)
R.765	84569142	84569394	chr7	SEMA3A (-745051), SEMA3D (+181983)
R.775	95026181	95026211	chr7	PON3 (-523)
R.779	96636616	96637152	chr7	DLX6 (+2024), DLX5 (+17525)
R.780	96655044	96655444	chr7	DLX5 (-835)
R.800	130126153	130126513	chr7	CEP41 (-45255), MEST (-5601)
R.804	138602025	138602695	chr7	ATP6V0A4 (-119419), KIAA1549 (+63704)
R.816	150689889	150690881	chr7	NOS3 (+2302), ATG9B (+31201)
R.826	158819531	158819822	chr7	VIPR2 (+117972), WDR60 (+170408)
R.827	158893862	158894694	chr7	VIPR2 (+43371), WDR60 (+245009)
R.829	337367	337566	chr8	FBXO25 (-19488), ZNF596 (+155083)
R.837	11549521	11549859	chr8	GATA4 (-12023), BLK (+198180)
R.852	39695788	39695862	chr8	ADAM2 (-17)
R.863	72469553	72469987	chr8	EYA1 (-195303), MSC (+286933)
R.875	109143682	109143860	chr8	RSPO2 (-47895), EIF3E (+117223)
R.887	140712424	140712499	chr8	COL22A1 (-786226), KCNK9 (+2837)
R.889	142439489	142440046	chr8	PTP4A3 (+37675)
R.905	96009985	96010290	chr9	FAM120A (-203866), WNK2 (+62926)
R.917	390328	390961	chr10	ZMYND11 (+210221), DIP2C (+344961)
R.918	1401818	1402253	chr10	IDI1 (-306926), ADARB2 (+377634)
R.919	1410519	1410671	chr10	IDI1 (-315485), ADARB2 (+369075)
R.920	3149673	3149852	chr10	PFKP (+40051), PITRM1 (+65240)
R.924	8085029	8085558	chr10	GATA3 (-11362), TAF3 (+224827)
R.936	35651873	35652116	chr10	GJD4 (-242343), CCNY (+26193)
R.948	64396659	64397487	chr10	ADO (-167443), ZNF365 (+263122)
R.954	72360348	72360448	chr10	PRF1 (+2133), PALD1 (+121821)
R.958	81154150	81154192	chr10	PPIF (+46937), ZCCHC24 (+51212)
R.962	88717364	88717623	chr10	SNCG (-919), MMRN2 (-122)
R.977	114135978	114136053	chr10	ACSL5 (+60)
R.992	130422176	130422945	chr10	MGMT (-842887), MKI67 (-497912)
R.993	131263962	131264145	chr10	MGMT (-1394)
R.994	131713829	131713890	chr10	EBF3 (+48245), MGMT (+448412)

R.995	131812518	131812878	chr10	GLRX3 (-121965), EBF3 (-50593)
R.998	133945044	133945935	chr10	DPYSL4 (-54914), JAKMIP3 (+27315)
R.1007	2367787	2368637	chr11	CD81 (-30312), TSPAN32 (+44969)
R.1008	2397576	2397614	chr11	CD81 (-929)
R.1010	2860989	2861204	chr11	CDKN1C (+46014), KCNQ1 (+394876)
R.1024	18747919	18748167	chr11	TMEM86A (+27705), PTPN5 (+65346)
R.1026	23752138	23752469	chr11	SVIP (-900893), LUZP2 (-766420)
R.1032	32454718	32454840	chr11	WT1 (+2397), RCN1 (+342329)
R.1035	32459428	32459954	chr11	WT1 (-2515)
R.1042	45737711	45737771	chr11	SLC35C1 (-88216), CHST1 (-50569)
R.1050	60738995	60739019	chr11	CD6 (-145)
R.1074	73359662	73359987	chr11	PLEKHB1 (+2602), RAB6A (+112357)
R.1080	86383696	86383804	chr11	ME3 (-72)
R.1107	133770764	133771191	chr11	SPATA19 (-55582), IGSF9B (+55902)
R.1111	149486	149764	chr12	IQSEC3 (-26306)
R.1122	15114393	15114710	chr12	ARHGDIB (+51)
R.1135	30976001	30976349	chr12	TSPAN11 (-103717), CAPRIN2 (-68727)
R.1139	40019919	40020078	chr12	ABCD2 (-6446), SLC2A13 (+479662)
R.1146	52301580	52301970	chr12	ACVRL1 (+573)
R.1147	52305328	52305401	chr12	ACVR1B (-40120), ACVRL1 (+4163)
R.1149	52799999	52800261	chr12	KRT82 (+9)
R.1165	81102486	81102749	chr12	MYF5 (-8073), MYF6 (+1341)
R.1185	115124973	115125549	chr12	TBX3 (-3866)
R.1186	115134886	115134918	chr12	TBX3 (-13507)
R.1187	115172502	115172748	chr12	TBX3 (-51230)
R.1198	125197665	125198295	chr12	NCOR2 (-218182), SCARB1 (+150413)
R.1205	133019880	133020030	chr12	GALNT9 (-329382), MUC8 (+30771)
R.1208	133409657	133409818	chr12	GOLGA3 (-4450)
R.1209	133414462	133415266	chr12	GOLGA3 (-9576), CHFR (+49330)
R.1210	133424420	133424655	chr12	GOLGA3 (-19250), CHFR (+39656)
R.1232	70682628	70683127	chr13	KLHL1 (-287)
R.1246	106912494	106912912	chr13	EFNB2 (+274759), DAOA (+794111)
R.1253	113642703	113642784	chr13	F7 (-117377), MCF2L (+19209)
R.1258	21503521	21504190	chr14	RNASE13 (-912)
R.1262	23816088	23816988	chr14	SLC22A17 (+5542), PABPN1 (+26040)
R.1292	80327885	80328189	chr14	DIO2 (+349806)
R.1303	96509376	96510362	chr14	BDKRB2 (-161266), C14orf132 (+4208)
R.1306	96730545	96730780	chr14	GSKIP (-99231), BDKRB1 (+8502)
R.1307	101509249	101509391	chr14	DIO3 (-518368), ENSG00000269375 (+150055)
R.1309	104569519	104569581	chr14	KIF26A (-35510), ASPG (+17534)
R.1312	104682880	104683315	chr14	C14orf144 (-27443), KIF26A (+78038)
R.1314	25329168	25329447	chr15	SNURF (+129173), UBE3A (+324487)
R.1315	29213626	29213748	chr15	APBA2 (+82567), NDNL2 (+348346)
R.1316	29410245	29410508	chr15	NDNL2 (+151656), APBA2 (+279257)
R.1402	4292559	4293524	chr16	SRL (-961)
R.1429	57576285	57576394	chr16	GPR114 (-261)
R.1453	87946341	87947130	chr16	SLC7A5 (-43642), CA5A (+23386)
R.1454	88238503	88239242	chr16	ZNF469 (-255006), BANP (+235249)
R.1472	6926003	6926414	chr17	BCL6B (-130)

R.1487	17061915	17062185	chr17	PLD6 (+47579), MPRIP (+115976)
R.1526	61926188	61926700	chr17	SMARCD2 (-6144), CSH2 (+24682)
R.1530	72258164	72258231	chr17	DNAI2 (-19453), TTYH2 (+48545)
R.1535	73506046	73506230	chr17	CASKIN2 (+5526), KIAA0195 (+53593)
R.1536	73642547	73642628	chr17	SMIM6 (+71)
R.1540	76126449	76126702	chr17	TMC6 (-1798), TMC8 (-291)
R.1547	79134486	79135071	chr17	AATK (+5038), BAIAP2 (+125817)
R.1548	79166676	79167630	chr17	AATK (-27336), AZI1 (+29577)
R.1558	19780644	19780878	chr18	GATA6 (+31357), CTAGE1 (+217117)
R.1570	37332566	37333028	chr18	NONE
R.1576	67136810	67137030	chr18	DOK6 (+68629), CD226 (+487312)
R.1594	3869525	3869879	chr19	ZFR2 (-676)
R.1621	12998099	12998435	chr19	GCDH (-3573), KLF1 (-272)
R.1623	14550846	14550997	chr19	PKN1 (-150)
R.1668	41073603	41074006	chr19	SHKBP1 (-8952), SPTBN4 (+101657)
R.1698	58037382	58038065	chr19	ZNF549 (-1037)
R.1705	58220773	58220837	chr19	ZNF154 (-269)
R.1710	58456148	58456963	chr19	ZNF418 (-9816), ZNF256 (+2483)
R.1743	37433745	37434158	chr20	PPP1R16B (-396)
R.1753	45337784	45338083	chr20	SLC2A10 (-192)
R.1762	58630954	58631038	chr20	CDH26 (+97514)
R.1766	62209634	62209865	chr20	HELZ2 (-5942), GMEB2 (+41479)
R.1796	39352174	39352432	chr22	APOBEC3B (-26111), APOBEC3A (+3547)
R.1799	42353589	42353933	chr22	SEPT3 (-19053), CENPM (-10593)
R.1809	49697565	49697802	chr22	BRD1 (+520768), FAM19A5 (+812412)

Supplementary Table S7. Differentially expressed DMR-genes BAV (FDR 10%)

Gene symbol	tc	P-value	FDR q
SNAI1	TC20000395,hg,1	0,004329004	0,064964119
PTGS2	TC01003638,hg,1	0,002164502	0,045444383
BIRC3	TC11000956,hg,1	0,002164502	0,045444383
ATF3	TC01001777,hg,1	0,002164502	0,045444383
GADD45B	TC19001938,hg,1	0,002164502	0,045444383
SMAD6	TC15000616,hg,1	0,002164502	0,045444383
SMAD6	TC15002247,hg,1	0,002164502	0,045444383
GADD45B	TC19000055,hg,1	0,002164502	0,045444383
JAG1	TC20001421,hg,1	0,002164502	0,045444383
PMEPA1	TC20001671,hg,1	0,008658009	0,096796537
SAV1	TC14001122,hg,1	0,002164502	0,045444383
TMEM39A	TC03002488,hg,1	0,002164502	0,045444383
MAP2K3	TC17002123,hg,1	0,002164502	0,045444383
PPAP2B	TC01002701,hg,1	0,002164502	0,045444383
TMEM217	TC06001682,hg,1	0,004329004	0,064964119
DUSP16	TC12001255,hg,1	0,002164502	0,045444383
RNF217	TC06003033,hg,1	0,002164502	0,045444383
ZBTB2	TC06002233,hg,1	0,002164502	0,045444383
JAG1	TC20000621,hg,1	0,002164502	0,045444383
SAMD4A	TC14000319,hg,1	0,004329004	0,064964119
GABARAPL1	TC12000159,hg,1	0,002164502	0,045444383
SMIM13	TC06002580,hg,1	0,002164502	0,045444383
ENC1	TC05001481,hg,1	0,002164502	0,045444383
SESN2	TC01000377,hg,1	0,008658009	0,096796537
MMD	TC17001711,hg,1	0,004329004	0,064964119
RNF217	TC06000956,hg,1	0,002164502	0,045444383
PIM1	TC06000541,hg,1	0,002164502	0,045444383
LRP12	TC08001511,hg,1	0,002164502	0,045444383
SPAG9	TC17001700,hg,1	0,002164502	0,045444383
EPC1	TC10001161,hg,1	0,002164502	0,045444383
PMEPA1	TC20000980,hg,1	0,004329004	0,064964119
ARID5A	TC02000583,hg,1	0,002164502	0,045444383
CDYL2	TC16001294,hg,1	0,008658009	0,096796537
CHMP1B	TC18000059,hg,1	0,002164502	0,045444383
MYO1E	TC15001492,hg,1	0,004329004	0,064964119
CPEB4	TC05000967,hg,1	0,002164502	0,045444383
CRY1	TC12001925,hg,1	0,002164502	0,045444383
MEF2A	TC15002404,hg,1	0,002164502	0,045444383
MAP2K3	TC17000297,hg,1	0,002164502	0,045444383
NEXN	TC01000789,hg,1	0,004329004	0,064964119
ELL2	TC05001610,hg,1	0,002164502	0,045444383
FZD8	TC10001179,hg,1	0,002164502	0,045444383
MEF2A	TC15000955,hg,1	0,002164502	0,045444383
LRP12	TC08002465,hg,1	0,002164502	0,045444383
NEDD4L	TC18000202,hg,1	0,002164502	0,045444383
BTG1	TC12001807,hg,1	0,002164502	0,045444383
ARID5B	TC10002092,hg,1	0,004329004	0,064964119
MXD1	TC02004970,hg,1	0,004329004	0,064964119

UNC5B	TC10000438,hg,1	0,002164502	0,045444383
FOSL2	TC02000183,hg,1	0,004329004	0,064964119
PBX3	TC09002258,hg,1	0,002164502	0,045444383
REST	TC04000335,hg,1	0,002164502	0,045444383
STXBP5	TC06001071,hg,1	0,002164502	0,045444383
NUP153	TC06002607,hg,1	0,008658009	0,096796537
JAK1	TC01002742,hg,1	0,002164502	0,045444383
MED13L	TC12002004,hg,1	0,002164502	0,045444383
TAPT1	TC04001061,hg,1	0,002164502	0,045444383
CSF1	TC01000955,hg,1	0,002164502	0,045444383
MYO1E	TC15002559,hg,1	0,002164502	0,045444383
ADCY4	TC14002316,hg,1	0,008658009	0,096796537
PBX3	TC09000652,hg,1	0,002164502	0,045444383
SOX4	TC06002615,hg,1	0,002164502	0,045444383
SMIM13	TC06000078,hg,1	0,002164502	0,045444383
SGK1	TC06002126,hg,1	0,004329004	0,064964119
SLC19A2	TC01003497,hg,1	0,008658009	0,096796537
SLC20A2	TC08001179,hg,1	0,004329004	0,064964119
SPRY2	TC13000763,hg,1	0,002164502	0,045444383
PHLPP1	TC18000224,hg,1	0,002164502	0,045444383
KDM6B	TC17000117,hg,1	0,002164502	0,045444383
ACOX3	TC04001018,hg,1	0,002164502	0,045444383
CYLD	TC16000443,hg,1	0,002164502	0,045444383
ACVR1	TC02002455,hg,1	0,002164502	0,045444383
APPL2	TC12001913,hg,1	0,008658009	0,096796537
ARHGAP31	TC03000607,hg,1	0,002164502	0,045444383
KLF3	TC04000221,hg,1	0,002164502	0,045444383
NRIP1	TC21000288,hg,1	0,002164502	0,045444383
CPEB2	TC04000140,hg,1	0,002164502	0,045444383
ZSCAN9	TC06000279,hg,1	0,004329004	0,064964119
TTL	TC02000706,hg,1	0,002164502	0,045444383
IER2	TC19000237,hg,1	0,004329004	0,064964119
FBXL20	TC17002612,hg,1	0,004329004	0,064964119
SMURF1	TC07001639,hg,1	0,002164502	0,045444383
SPOPL	TC02000894,hg,1	0,002164502	0,045444383
TNIP1	TC05003310,hg,1	0,002164502	0,045444383
BRAF	TC07003197,hg,1	0,002164502	0,045444383
NR4A1	TC12000414,hg,1	0,002164502	0,045444383
RGPD3	TC02000672,hg,1	0,002164502	0,045444383
SPOCD1	TC01002459,hg,1	0,008658009	0,096796537
AGO2	TC08001683,hg,1	0,002164502	0,045444383
XBP1	TC22000627,hg,1	0,008658009	0,096796537
PHLPP2	TC16001250,hg,1	0,002164502	0,045444383
UBTF	TC17002655,hg,1	0,004329004	0,064964119
CEBPG	TC19000437,hg,1	0,004329004	0,064964119
KLHL21	TC01002155,hg,1	0,002164502	0,045444383
RGPD3	TC02002203,hg,1	0,002164502	0,045444383
TPM1	TC15000578,hg,1	0,002164502	0,045444383
TNIP1	TC05001946,hg,1	0,002164502	0,045444383
LNX2	TC13000511,hg,1	0,002164502	0,045444383

LRRC8A	TC09000704,hg,1	0,002164502	0,045444383
SHB	TC09002893,hg,1	0,004329004	0,064964119
SPSB1	TC01000115,hg,1	0,002164502	0,045444383
ARHGAP21	TC10001109,hg,1	0,008658009	0,096796537
INPP5A	TC10000946,hg,1	0,002164502	0,045444383
LARP6	TC15001615,hg,1	0,004329004	0,064964119
PTPN21	TC14001423,hg,1	0,002164502	0,045444383
KLF13	TC15000198,hg,1	0,004329004	0,064964119
KANSL1L	TC02002730,hg,1	0,002164502	0,045444383
PIP5K1C	TC19001048,hg,1	0,004329004	0,064964119
HOOK3	TC08001897,hg,1	0,002164502	0,045444383
BRAF	TC07001926,hg,1	0,002164502	0,045444383
FBXL20	TC17001448,hg,1	0,002164502	0,045444383
SRGAP1	TC12000564,hg,1	0,008658009	0,096796537
MED13	TC17001772,hg,1	0,002164502	0,045444383
ZNF432	TC19001794,hg,1	0,002164502	0,045444383
SRF	TC06000596,hg,1	0,008658009	0,096796537
STX12	TC01000368,hg,1	0,008658009	0,096796537
ABL2	TC01003566,hg,1	0,002164502	0,045444383
TBC1D9	TC04001592,hg,1	0,008658009	0,096796537
NABP1	TC02001128,hg,1	0,008658009	0,096796537
NLK	TC17000315,hg,1	0,004329004	0,064964119
FRMD8	TC11000627,hg,1	0,002164502	0,045444383
PCNX	TC14000429,hg,1	0,002164502	0,045444383
DENND3	TC08000793,hg,1	0,002164502	0,045444383
SATB1	TC03003338,hg,1	0,004329004	0,064964119
WBP4	TC13000141,hg,1	0,002164502	0,045444383
DPYSL3	TC05001909,hg,1	0,004329004	0,064964119
RGPD3	TC02002172,hg,1	0,002164502	0,045444383
ZNF92	TC07000395,hg,1	0,008658009	0,096796537
BMP2	TC20000067,hg,1	0,002164502	0,045444383
PDE8A	TC15000824,hg,1	0,002164502	0,045444383
CCNL1	TC03001939,hg,1	0,002164502	0,045444383
SMOX	TC20000048,hg,1	0,004329004	0,064964119
GPR176	TC15001225,hg,1	0,002164502	0,045444383
HABP4	TC09000480,hg,1	0,002164502	0,045444383
CHKA	TC11002002,hg,1	0,008658009	0,096796537
TAP1	TC06001574,hg,1	0,004329004	0,064964119
SPIRE1	TC18000374,hg,1	0,002164502	0,045444383
CDC42SE1	TC01003218,hg,1	0,002164502	0,045444383
ID3	TC01002347,hg,1	0,008658009	0,096796537
PURB	TC07001347,hg,1	0,008658009	0,096796537
TRAF6	TC11001560,hg,1	0,004329004	0,064964119
HES1	TC03002732,hg,1	0,008658009	0,096796537
HIVEP1	TC06000086,hg,1	0,004329004	0,064964119
HDAC5	TC17001572,hg,1	0,002164502	0,045444383
ZNFX1	TC20000924,hg,1	0,002164502	0,045444383
CUX1	TC07002501,hg,1	0,008658009	0,096796537
CAMSAP1	TC09001720,hg,1	0,004329004	0,064964119
PURB	TC07002923,hg,1	0,002164502	0,045444383

IRF2BP2	TC01003985,hg,1	0,004329004	0,064964119
TNFAIP1	TC17000320,hg,1	0,002164502	0,045444383
SIRT1	TC10000400,hg,1	0,002164502	0,045444383
CMTM3	TC16001601,hg,1	0,002164502	0,045444383
ZCCHC14	TC16001332,hg,1	0,002164502	0,045444383
PLEKHA1	TC10000886,hg,1	0,002164502	0,045444383
ABHD5	TC03000231,hg,1	0,002164502	0,045444383
DNAJC1	TC10001097,hg,1	0,002164502	0,045444383
RNF115	TC01001098,hg,1	0,002164502	0,045444383
AGO2	TC08001684,hg,1	0,008658009	0,096796537
FERMT2	TC14001144,hg,1	0,002164502	0,045444383
EZH2	TC07001993,hg,1	0,004329004	0,064964119
DNAJC18	TC05003430,hg,1	0,004329004	0,064964119
PPP1R13L	TC19001619,hg,1	0,008658009	0,096796537
OSGIN2	TC08000546,hg,1	0,008658009	0,096796537
ZNF460	TC19000937,hg,1	0,008658009	0,096796537
UBTF	TC17001576,hg,1	0,002164502	0,045444383
RB1CC1	TC08001210,hg,1	0,002164502	0,045444383
RNPC3	TC01004581,hg,1	0,002164502	0,045444383
CYTH2	TC19000704,hg,1	0,002164502	0,045444383
JAM2	TC21000077,hg,1	0,002164502	0,045444383
B3GNT2	TC02000357,hg,1	0,008658009	0,096796537
PBLD	TC10001345,hg,1	0,004329004	0,064964119
FBXO30	TC06002196,hg,1	0,004329004	0,064964119
ZNF345	TC19000508,hg,1	0,002164502	0,045444383
MAP2K4	TC17002052,hg,1	0,002164502	0,045444383
RIT1	TC01003324,hg,1	0,002164502	0,045444383
TMEM87A	TC15001263,hg,1	0,002164502	0,045444383
CYTH1	TC17001920,hg,1	0,004329004	0,064964119
ANTXR2	TC04001328,hg,1	0,002164502	0,045444383
TAOK1	TC17002149,hg,1	0,008658009	0,096796537
AP3M2	TC08000324,hg,1	0,008658009	0,096796537
SCAF11	TC12001425,hg,1	0,002164502	0,045444383
BRAF	TC07001927,hg,1	0,002164502	0,045444383
ARPC5	TC01003618,hg,1	0,002164502	0,045444383
ZFYVE1	TC14001282,hg,1	0,002164502	0,045444383
GNA13	TC17001808,hg,1	0,002164502	0,045444383
LRRC49	TC15000647,hg,1	0,002164502	0,045444383
ATF4	TC22000317,hg,1	0,008658009	0,096796537
RHEB	TC07002040,hg,1	0,002164502	0,045444383
HOOK3	TC08002600,hg,1	0,002164502	0,045444383
ANKRD12	TC18000042,hg,1	0,002164502	0,045444383
PTPDC1	TC09000455,hg,1	0,004329004	0,064964119
CUX1	TC07000650,hg,1	0,004329004	0,064964119
GLIS2	TC16000128,hg,1	0,008658009	0,096796537
SERTAD3	TC19001541,hg,1	0,002164502	0,045444383
STIM2	TC04000184,hg,1	0,008658009	0,096796537
FBRS	TC16000355,hg,1	0,002164502	0,045444383
OPTN	TC10000097,hg,1	0,004329004	0,064964119
TNFAIP8	TC05000565,hg,1	0,008658009	0,096796537

CBL	TC11001089,hg,1	0,004329004	0,064964119
GCC2	TC02003486,hg,1	0,002164502	0,045444383
TRNP1	TC01000356,hg,1	0,008658009	0,096796537
DNAJC6	TC01000729,hg,1	0,004329004	0,064964119
MEX3C	TC18000517,hg,1	0,002164502	0,045444383
AFF4	TC05001776,hg,1	0,002164502	0,045444383
ZNF229	TC19001606,hg,1	0,008658009	0,096796537
BAZ2B	TC02005063,hg,1	0,008658009	0,096796537
AKAP12	TC06001100,hg,1	0,004329004	0,064964119
STRN	TC02001736,hg,1	0,002164502	0,045444383
MTPN	TC07003185,hg,1	0,002164502	0,045444383
NIPBL	TC05000166,hg,1	0,002164502	0,045444383
RANBP2	TC02000668,hg,1	0,002164502	0,045444383
OSBPL8	TC12001754,hg,1	0,002164502	0,045444383
C8orf44-SGK3	TC08002607,hg,1	0,002164502	0,045444383
SGK3	TC08002607,hg,1	0,002164502	0,045444383
DYRK1A	TC21000164,hg,1	0,002164502	0,045444383
KMT2C	TC07002045,hg,1	0,002164502	0,045444383
CMTM3	TC16000520,hg,1	0,002164502	0,045444383
RCC2	TC01002289,hg,1	0,004329004	0,064964119
CHMP4B	TC20000235,hg,1	0,004329004	0,064964119
RNPC3	TC01000917,hg,1	0,002164502	0,045444383
GJA1	TC06000945,hg,1	0,002164502	0,045444383
CMTM4	TC16001171,hg,1	0,008658009	0,096796537
GTF2A1	TC14001368,hg,1	0,008658009	0,096796537
ZNF140	TC12001069,hg,1	0,004329004	0,064964119
PLCB1	TC20000072,hg,1	0,008658009	0,096796537
ADAM17	TC02001558,hg,1	0,002164502	0,045444383
SLC3A2	TC11000567,hg,1	0,004329004	0,064964119
TGIF1	TC18000018,hg,1	0,004329004	0,064964119
FXR2	TC17001092,hg,1	0,004329004	0,064964119
TYRO3	TC15000310,hg,1	0,008658009	0,096796537
ZC2HC1A	TC08000506,hg,1	0,002164502	0,045444383
ATP11B	TC03000976,hg,1	0,002164502	0,045444383
RNF111	TC15000448,hg,1	0,004329004	0,064964119
USP7	TC16000852,hg,1	0,002164502	0,045444383
BCL2L2	TC14001595,hg,1	0,008658009	0,096796537
STX1A	TC07001504,hg,1	0,002164502	0,045444383
WAC	TC10000196,hg,1	0,002164502	0,045444383
STX3	TC11000499,hg,1	0,002164502	0,045444383
MMP14	TC14000133,hg,1	0,002164502	0,045444383
PDLIM5	TC04000501,hg,1	0,002164502	0,045444383
CTAGE5	TC14002296,hg,1	0,002164502	0,045444383
KIAA0232	TC04000074,hg,1	0,008658009	0,096796537
CCDC92	TC12002094,hg,1	0,002164502	0,045444383
RRAGC	TC01006351,hg,1	0,002164502	0,045444383
ARID4B	TC01006389,hg,1	0,002164502	0,045444383
TBC1D20	TC20000541,hg,1	0,002164502	0,045444383
PPFIA1	TC11000724,hg,1	0,004329004	0,064964119
PICALM	TC11002165,hg,1	0,002164502	0,045444383

ACVR2A	TC02000923,hg,1	0,004329004	0,064964119
TRPM7	TC15001357,hg,1	0,002164502	0,045444383
IST1	TC16000598,hg,1	0,002164502	0,045444383
E2F3	TC06000128,hg,1	0,008658009	0,096796537
TSTD2	TC09001395,hg,1	0,004329004	0,064964119
SPPL2A	TC15001358,hg,1	0,002164502	0,045444383
AP5Z1	TC07000045,hg,1	0,002164502	0,045444383
ATG13	TC11000399,hg,1	0,002164502	0,045444383
UFM1	TC13000130,hg,1	0,004329004	0,064964119
ZMYND11	TC10000002,hg,1	0,002164502	0,045444383
SLC12A6	TC15001192,hg,1	0,008658009	0,096796537
SLC35F2	TC11002262,hg,1	0,002164502	0,045444383
PAN3	TC13000085,hg,1	0,004329004	0,064964119
DESI2	TC01001989,hg,1	0,002164502	0,045444383
INTS6	TC13000678,hg,1	0,002164502	0,045444383
EP300	TC22000332,hg,1	0,002164502	0,045444383
GCC2	TC02000666,hg,1	0,002164502	0,045444383
IL17RA	TC22000019,hg,1	0,008658009	0,096796537
TMEM136	TC11001101,hg,1	0,008658009	0,096796537
MITD1	TC02002132,hg,1	0,008658009	0,096796537
SHOC2	TC10000809,hg,1	0,002164502	0,045444383
FBXO11	TC02001826,hg,1	0,002164502	0,045444383
SLC3A2	TC11002708,hg,1	0,004329004	0,064964119
UBE3A	TC15001076,hg,1	0,002164502	0,045444383
FAM76B	TC11002208,hg,1	0,008658009	0,096796537
RASAL2	TC01001539,hg,1	0,008658009	0,096796537
CTNNB1	TC03000214,hg,1	0,008658009	0,096796537
WAC	TC10001962,hg,1	0,002164502	0,045444383
DENND1B	TC01003665,hg,1	0,008658009	0,096796537
CD276	TC15000667,hg,1	0,007795827	0,096796537
ZNF776	TC19002668,hg,1	0,004329004	0,064964119
MAP2K4	TC17000155,hg,1	0,008658009	0,096796537
TAB2	TC06004102,hg,1	0,002164502	0,045444383
ATG14	TC14001159,hg,1	0,004329004	0,064964119
ABHD12	TC20000732,hg,1	0,002164502	0,045444383
POGZ	TC01003227,hg,1	0,008658009	0,096796537
EIF1	TC17000514,hg,1	0,008658009	0,096796537
TSG101	TC11001466,hg,1	0,004329004	0,064964119
C1orf198	TC01003959,hg,1	0,004329004	0,064964119
FER	TC05000509,hg,1	0,004329004	0,064964119
GPR161	TC01003485,hg,1	0,002164502	0,045444383
MAP4K5	TC14001120,hg,1	0,002164502	0,045444383
AEBP2	TC12000222,hg,1	0,002164502	0,045444383
PTPN12	TC07000495,hg,1	0,002164502	0,045444383
FGFR1	TC08001147,hg,1	0,008658009	0,096796537
ZNF503	TC10002967,hg,1	0,008658009	0,096796537
BRAP	TC12001974,hg,1	0,008658009	0,096796537
CXXC5	TC05000715,hg,1	0,002164502	0,045444383
PCGF1	TC02004929,hg,1	0,008658009	0,096796537
TPBG	TC06000766,hg,1	0,008658009	0,096796537

ZNF706	TC08001489,hg,1	0,002164502	0,045444383
PPP3CC	TC08000172,hg,1	0,004329004	0,064964119
ZNF821	TC16001254,hg,1	0,002164502	0,045444383
DDX5	TC17001800,hg,1	0,004329004	0,064964119
KRAS	TC12001314,hg,1	0,008658009	0,096796537
TRA2A	TC07001197,hg,1	0,008658009	0,096796537
CD81	TC11000063,hg,1	0,004329004	0,064964119
MICAL3	TC22000478,hg,1	0,008658009	0,096796537
VMP1	TC17000727,hg,1	0,008658009	0,096796537
CAB39	TC02001379,hg,1	0,008658009	0,096796537
PTPN12	TC07002426,hg,1	0,004329004	0,064964119
SERPINB8	TC18001001,hg,1	0,008658009	0,096796537
RAP1B	TC12000601,hg,1	0,002164502	0,045444383
LENG1	TC19001835,hg,1	0,004329004	0,064964119
TRIM39	TC06004068,hg,1	0,008658009	0,096796537
TRIM39-RPP21	TC06004068,hg,1	0,008658009	0,096796537
RING1	TC06000415,hg,1	0,004329004	0,064964119
CSNK1G1	TC15002806,hg,1	0,008658009	0,096796537
WLS	TC01002763,hg,1	0,008658009	0,096796537
EAF1	TC03000096,hg,1	0,008658009	0,096796537
C6orf62	TC06001328,hg,1	0,002164502	0,045444383
ZNF664	TC12003263,hg,1	0,002164502	0,045444383
GNL1	TC06001501,hg,1	0,008658009	0,096796537
ZNF821	TC16001950,hg,1	0,008658009	0,096796537
CTNNA1	TC05000702,hg,1	0,004329004	0,064964119
HLA-E	TC06000338,hg,1	0,004329004	0,064964119
MTPN	TC07001891,hg,1	0,004329004	0,064964119
JOSD1	TC22000796,hg,1	0,004329004	0,064964119
ZFYVE27	TC10000703,hg,1	0,008658009	0,096796537
TCIRG1	TC11000704,hg,1	0,008658009	0,096796537
JAK1	TC01002741,hg,1	0,002164502	0,045444383
GTPBP1	TC22000304,hg,1	0,008658009	0,096796537
GPBP1L1	TC01002613,hg,1	0,004329004	0,064964119
ARID3B	TC15000682,hg,1	0,008127043	0,096796537
ITGB1	TC10001163,hg,1	0,008658009	0,096796537
ZSCAN18	TC19001911,hg,1	0,008658009	0,096796537
MTDH	TC08000594,hg,1	0,008658009	0,096796537
ZNF331	TC19000812,hg,1	0,008658009	0,096796537
IFFO1	TC12001141,hg,1	0,008658009	0,096796537
SYDE1	TC19000269,hg,1	0,004329004	0,064964119
PKM	TC15002776,hg,1	0,008658009	0,096796537
EIF5A	TC17000088,hg,1	0,008658009	0,096796537
CAV1	TC07003339,hg,1	0,007795827	0,096796537
DDX11	TC12001201,hg,1	0,004329004	0,064964119
CALM1	TC19000665,hg,1	0,008658009	0,096796537
PKM	TC15002610,hg,1	0,008658009	0,096796537
CARD10	TC22001492,hg,1	0,002164502	0,045444383
ZFP41	TC08002608,hg,1	0,004329004	0,064964119
CCDC134	TC22000345,hg,1	0,004329004	0,064964119
DPH2	TC01000554,hg,1	0,008658009	0,096796537

HMGN2	TC01000346,hg,1	0,002164502	0,045444383
NPRL2	TC03001431,hg,1	0,002164502	0,045444383
C6orf1	TC06001653,hg,1	0,008658009	0,096796537
ERG	TC21000450,hg,1	0,006392269	0,095606108
PSME3	TC17000538,hg,1	0,008658009	0,096796537
PRR3	TC06000340,hg,1	0,008658009	0,096796537
CNOT7	TC08001011,hg,1	0,008658009	0,096796537
DYNLL1	TC12000946,hg,1	0,004329004	0,064964119
AKAP1	TC17000704,hg,1	0,002164502	0,045444383
TUBB	TC06000347,hg,1	0,004329004	0,064964119
RAB11A	TC15000609,hg,1	0,008658009	0,096796537
TUBA1C	TC12000367,hg,1	0,002164502	0,045444383
SNX2	TC05000576,hg,1	0,008658009	0,096796537
CCDC88C	TC14001436,hg,1	0,004329004	0,064964119
ITSN1	TC21000681,hg,1	0,002164502	0,045444383
TFAP4	TC16001756,hg,1	0,004329004	0,064964119
TFAP4	TC16000827,hg,1	0,008658009	0,096796537
CSRP2BP	TC20000117,hg,1	0,002164502	0,045444383
PET117	TC20000117,hg,1	0,002164502	0,045444383
ZFP64	TC20000947,hg,1	0,002164502	0,045444383
NR1D1	TC17001459,hg,1	0,002164502	0,045444383
ADAT2	TC06002186,hg,1	0,008658009	0,096796537
PGAP1	TC02002644,hg,1	0,008658009	0,096796537
ANP32A	TC15001594,hg,1	0,008658009	0,096796537
ZHX3	TC20000848,hg,1	0,004329004	0,064964119
PIK3R1	TC05000291,hg,1	0,008658009	0,096796537
ALDH3A2	TC17000246,hg,1	0,002164502	0,045444383
SH2D3C	TC09001608,hg,1	0,002164502	0,045444383
SLC25A30	TC13000634,hg,1	0,002164502	0,045444383
TOB1	TC17001698,hg,1	0,002164502	0,045444383
NR1D1	TC17002615,hg,1	0,002164502	0,045444383
TRIM25	TC17001716,hg,1	0,008658009	0,096796537
CHAC2	TC02000310,hg,1	0,004329004	0,064964119
AMD1	TC06000883,hg,1	0,002164502	0,045444383
NFIA	TC01000702,hg,1	0,002164502	0,045444383
CITED2	TC06002168,hg,1	0,002164502	0,045444383
SKP2	TC05000161,hg,1	0,004329004	0,064964119
EGFL7	TC09002355,hg,1	0,008658009	0,096796537
BCL2L1	TC20000756,hg,1	0,002164502	0,045444383
PRICKLE1	TC12001405,hg,1	0,004329004	0,064964119
ETV1	TC07001152,hg,1	0,008658009	0,096796537
DUSP6	TC12001796,hg,1	0,002164502	0,045444383
PLK2	TC05001385,hg,1	0,002164502	0,045444383
CDC42EP3	TC02001746,hg,1	0,004329004	0,064964119
BCL2L1	TC20001531,hg,1	0,002164502	0,045444383
SNRK	TC03000229,hg,1	0,002164502	0,045444383
PIK3CG	TC07000687,hg,1	0,002164502	0,045444383
PTGER4	TC05000184,hg,1	0,004329004	0,064964119
SPRY1	TC04000632,hg,1	0,002164502	0,045444383
PTX3	TC03000862,hg,1	0,002164502	0,045444383

PRICKLE1	TC12002841,hg,1	0,002164502	0,045444383
SDPR	TC02002627,hg,1	0,004329004	0,064964119

Differentially expressed DMR-genes TAV (FDR 10%)

Gene symbol	tc	P-value	FDR q
PTGS2	TC01003638.hg.1	0,000582751	0,010863718
SLC7A2	TC08001816.hg.1	0,000582751	0,010863718
NOG	TC17000698.hg.1	0,000582751	0,010863718
SLC7A2	TC08000127.hg.1	0,000582751	0,010863718
SNAI1	TC20000395.hg.1	0,011072261	0,058833921
GADD45B	TC19001938.hg.1	0,002331002	0,022134595
JHDM1D	TC07001917.hg.1	0,000582751	0,010863718
GADD45B	TC19000055.hg.1	0,000582751	0,010863718
ATF3	TC01001777.hg.1	0,000582751	0,010863718
MT1G	TC16001135.hg.1	0,011072261	0,058833921
JAG1	TC20000621.hg.1	0,000582751	0,010863718
JAG1	TC20001421.hg.1	0,001165501	0,015073094
HEY2	TC06000960.hg.1	0,000582751	0,010863718
SMAD6	TC15000616.hg.1	0,002331002	0,022134595
SGK1	TC06002126.hg.1	0,000582751	0,010863718
SAV1	TC14001122.hg.1	0,011072261	0,058833921
BHLHE40	TC03000015.hg.1	0,001165501	0,015073094
E2F7	TC12002970.hg.1	0,001165501	0,015073094
NFKB1	TC04000526.hg.1	0,000582751	0,010863718
E2F7	TC12001756.hg.1	0,001165501	0,015073094
SMAD6	TC15002247.hg.1	0,002331002	0,022134595
KITLG	TC12001792.hg.1	0,002331002	0,022134595
WEE1	TC11000174.hg.1	0,000582751	0,010863718
ENC1	TC05001481.hg.1	0,000582751	0,010863718
TMC7	TC16000207.hg.1	0,001165501	0,015073094
MMD	TC17001711.hg.1	0,002331002	0,022134595
PGF	TC14001313.hg.1	0,001165501	0,015073094
BACH1	TC21000097.hg.1	0,000582751	0,010863718
RNF217	TC06003033.hg.1	0,004079254	0,031319232
BACH1	TC21000651.hg.1	0,002331002	0,022134595
TPM1	TC15000578.hg.1	0,000582751	0,010863718
DUSP16	TC12001255.hg.1	0,004079254	0,031319232
NR3C1	TC05001887.hg.1	0,001165501	0,015073094
PDGFA	TC07001073.hg.1	0,000582751	0,010863718
RHOB	TC02000116.hg.1	0,000582751	0,010863718
GABARAPL1	TC12000159.hg.1	0,001165501	0,015073094
BIRC2	TC11000957.hg.1	0,001165501	0,015073094
RFX2	TC19001088.hg.1	0,004079254	0,031319232
NFKB2	TC10000753.hg.1	0,000582751	0,010863718
TIPARP	TC03000853.hg.1	0,000582751	0,010863718
NDRG1	TC08001659.hg.1	0,001165501	0,015073094
SAMD4A	TC14000319.hg.1	0,011072261	0,058833921
ENKUR	TC10001111.hg.1	0,006993007	0,044547453
PGF	TC14002090.hg.1	0,001165501	0,015073094
UACA	TC15001612.hg.1	0,000582751	0,010863718

KLF10	TC08002455.hg.1	0,004079254	0,031319232
SOX4	TC06002615.hg.1	0,000582751	0,010863718
FLT1	TC13001387.hg.1	0,017482517	0,079419358
SERTAD1	TC19001540.hg.1	0,000582751	0,010863718
RNF217	TC06000956.hg.1	0,017482517	0,079419358
PAWR	TC12001765.hg.1	0,000582751	0,010863718
GATA6	TC18000094.hg.1	0,011072261	0,058833921
PITPNC1	TC17002321.hg.1	0,000582751	0,010863718
TAPT1	TC04001061.hg.1	0,002331002	0,022134595
SPRY2	TC13000763.hg.1	0,006993007	0,044547453
CHMP1B	TC18000059.hg.1	0,011072261	0,058833921
CEBPG	TC19000437.hg.1	0,002331002	0,022134595
RND3	TC02002419.hg.1	0,011072261	0,058833921
SPAG9	TC17001700.hg.1	0,006993007	0,044547453
VEGFA	TC06000608.hg.1	0,000582751	0,010863718
RASGRP3	TC02000221.hg.1	0,011072261	0,058833921
CD58	TC01005732.hg.1	0,002331002	0,022134595
GABARAPL3	TC15001847.hg.1	0,000582751	0,010863718
PLEKHO1	TC01001178.hg.1	0,001165501	0,015073094
SOX4	TC06000135.hg.1	0,017482517	0,079419358
CD58	TC01003029.hg.1	0,002331002	0,022134595
HIVEP1	TC06000086.hg.1	0,000582751	0,010863718
SLC20A2	TC08001179.hg.1	0,011072261	0,058833921
ADCY4	TC14002316.hg.1	0,017482517	0,079419358
MEF2A	TC15000955.hg.1	0,017482517	0,079419358
OSGIN2	TC08000546.hg.1	0,017482517	0,079419358
OSBPL8	TC12001754.hg.1	0,006993007	0,044547453
WTIP	TC19000445.hg.1	0,002331002	0,022134595
KLF10	TC08001500.hg.1	0,001165501	0,015073094
VEGFA	TC06002799.hg.1	0,017482517	0,079419358
FERMT2	TC14001144.hg.1	0,001165501	0,015073094
LRRC4	TC07001842.hg.1	0,011072261	0,058833921
TCF7	TC05002628.hg.1	0,002331002	0,022134595
ARID2	TC12000331.hg.1	0,017482517	0,079419358
LIMS1	TC02000667.hg.1	0,000582751	0,010863718
NAB2	TC12000520.hg.1	0,001165501	0,015073094
JAZF1	TC07001232.hg.1	0,001165501	0,015073094
PLEKHA1	TC10000886.hg.1	0,001165501	0,015073094
EPN2	TC17001235.hg.1	0,004079254	0,031319232
PDE8A	TC15000824.hg.1	0,004079254	0,031319232
CTU2	TC16002023.hg.1	0,001165501	0,015073094
RUNX1	TC21000423.hg.1	0,002331002	0,022134595
EPN2	TC17000233.hg.1	0,004079254	0,031319232
NLK	TC17000315.hg.1	0,004079254	0,031319232
TNFAIP8	TC05000565.hg.1	0,006993007	0,044547453
ITGB1	TC10000229.hg.1	0,006993007	0,044547453
RHEB	TC07002040.hg.1	0,017482517	0,079419358
CYB5R2	TC11001372.hg.1	0,017482517	0,079419358
LIMS1	TC02002201.hg.1	0,011072261	0,058833921
PICALM	TC11002165.hg.1	0,000582751	0,010863718

SPPL2A	TC15001358.hg.1	0,017482517	0,079419358
VMP1	TC17000727.hg.1	0,002331002	0,022134595
UBTF	TC17001576.hg.1	0,017482517	0,079419358
MYADM	TC19000865.hg.1	0,006993007	0,044547453
SLC38A2	TC12001427.hg.1	0,000582751	0,010863718
GNB4	TC03002038.hg.1	0,017482517	0,079419358
STARD10	TC11003492.hg.1	0,006993007	0,044547453
RANBP2	TC02000668.hg.1	0,000582751	0,010863718
SERPINB8	TC18001001.hg.1	0,011072261	0,058833921
CMTM3	TC16000520.hg.1	0,004079254	0,031319232
FAM110A	TC20000012.hg.1	0,011072261	0,058833921
PODXL	TC07001874.hg.1	0,010517951	0,058833921
SIAH1	TC16001095.hg.1	0,004079254	0,031319232
VSTM2L	TC20000277.hg.1	0,011072261	0,058833921
TAB2	TC06004102.hg.1	0,004079254	0,031319232
PSMD14	TC02000970.hg.1	0,017482517	0,079419358
PRR24	TC19000675.hg.1	0,011072261	0,058833921
NOTUM	TC17001968.hg.1	0,001165501	0,015073094
MAPKAPK2	TC01001727.hg.1	0,017482517	0,079419358
BMPR2	TC02001192.hg.1	0,001165501	0,015073094
NLRC5	TC16000482.hg.1	0,002331002	0,022134595
DSTN	TC20000109.hg.1	0,017482517	0,079419358
AP5Z1	TC07000045.hg.1	0,000582751	0,010863718
IGF2	TC11001274.hg.1	0,006993007	0,044547453
HIST1H4D	TC06000172.hg.1	0,017482517	0,079419358
HIST1H4J	TC06000172.hg.1	0,017482517	0,079419358
HIST1H4K	TC06000172.hg.1	0,017482517	0,079419358
HIST4H4	TC06000172.hg.1	0,017482517	0,079419358
TMEM243	TC07001575.hg.1	0,017482517	0,079419358
ZNF821	TC16001950.hg.1	0,017482517	0,079419358
YTHDF3	TC08000424.hg.1	0,017482517	0,079419358
CXXC5	TC05000715.hg.1	0,001165501	0,015073094
ZNF821	TC16001254.hg.1	0,006993007	0,044547453
ORMDL3	TC17002852.hg.1	0,002331002	0,022134595
MFSD12	TC19002294.hg.1	0,006993007	0,044547453
ZNF48	TC16000346.hg.1	0,017482517	0,079419358
GSE1	TC16000664.hg.1	0,017482517	0,079419358
HES4	TC01002080.hg.1	0,002331002	0,022134595
TPM4	TC19000316.hg.1	0,017482517	0,079419358
STK11IP	TC02001333.hg.1	0,009504354	0,058833921
SLC19A1	TC21000549.hg.1	0,004079254	0,031319232
HSP90AB1	TC06000615.hg.1	0,017482517	0,079419358
FTL	TC19000717.hg.1	0,017482517	0,079419358
PDE4A	TC19000177.hg.1	0,010263769	0,058833921
CC2D1A	TC19000247.hg.1	0,014749453	0,078001379
ZNRF3	TC22000189.hg.1	0,00214071	0,022134595
LZTS2	TC10000730.hg.1	0,004079254	0,031319232
TTC7A	TC02000289.hg.1	0,020880821	0,093862657
RAB43	TC03003393.hg.1	0,004079254	0,031319232
TUBGCP6	TC22000908.hg.1	0,021307711	0,094939392

ITM2B	TC13000189.hg.1	0,017482517	0,079419358
AKT2	TC19001532.hg.1	0,015082035	0,079010584
ZSCAN25	TC07000599.hg.1	0,020037002	0,090361045
TBC1D10A	TC22000649.hg.1	0,010517951	0,058833921
CACNB1	TC17001446.hg.1	0,017482517	0,079419358
MKS1	TC17001726.hg.1	0,004079254	0,031319232
ZNF331	TC19000812.hg.1	0,007226222	0,045616039
MPV17	TC02001681.hg.1	0,006993007	0,044547453
TRMT61A	TC14001887.hg.1	0,017482517	0,079419358
ARFGAP1	TC20000511.hg.1	0,017482517	0,079419358
MGAT1	TC05002151.hg.1	0,021307711	0,094939392
EML2	TC19001625.hg.1	0,021307711	0,094939392
ZNF160	TC19001811.hg.1	0,010517951	0,058833921
NDUFAF6	TC08000579.hg.1	0,017482517	0,079419358
RGS19	TC20001046.hg.1	0,006993007	0,044547453
CHP1	TC15000304.hg.1	0,017482517	0,079419358
NFKBID	TC19001457.hg.1	0,006993007	0,044547453
SKIV2L	TC06000389.hg.1	0,001165501	0,015073094
DAK	TC11000529.hg.1	0,008734438	0,054888112
AES	TC19002293.hg.1	0,011072261	0,058833921
PQLC2	TC01000246.hg.1	0,004079254	0,031319232
TMEM219	TC16000334.hg.1	0,011072261	0,058833921
PVRL1	TC11002372.hg.1	0,021307711	0,094939392
SGSM2	TC17000023.hg.1	0,006993007	0,044547453
UNK	TC17000862.hg.1	0,017482517	0,079419358
INO80E	TC16000336.hg.1	0,017482517	0,079419358
AES	TC19001041.hg.1	0,004079254	0,031319232
KLHL22	TC22001484.hg.1	0,011072261	0,058833921
QTRT1	TC19002622.hg.1	0,017482517	0,079419358
PPP2R4	TC09000709.hg.1	0,004000668	0,031319232
GNAI2	TC03000307.hg.1	0,002331002	0,022134595
NQO1	TC16001225.hg.1	0,017482517	0,079419358
CTSA	TC20000361.hg.1	0,017482517	0,079419358
NTMT1	TC09000721.hg.1	0,006993007	0,044547453
ABCC10	TC06000600.hg.1	0,000582751	0,010863718
SH3PXD2A	TC10001635.hg.1	0,014749453	0,078001379
GAS8	TC16000711.hg.1	0,001165501	0,015073094
KIAA0195	TC17000853.hg.1	0,006993007	0,044547453
GCAT	TC22000289.hg.1	0,017482517	0,079419358
ASCC1	TC10001377.hg.1	0,015082035	0,079010584
TOP3B	TC22001434.hg.1	0,000582751	0,010863718
SSH3	TC11000684.hg.1	0,004079254	0,031319232
MYO18A	TC17001315.hg.1	0,007035599	0,044614821
CIC	TC19000596.hg.1	0,011072261	0,058833921
SERGEF	TC11003435.hg.1	0,004079254	0,031319232
IRF3	TC19001722.hg.1	0,006993007	0,044547453
ZNF343	TC20001745.hg.1	0,006993007	0,044547453
B4GALT2	TC01000556.hg.1	0,010517951	0,058833921
FAM189B	TC01003309.hg.1	0,004079254	0,031319232
MBD2	TC18000521.hg.1	0,011072261	0,058833921

MPI	TC15000687.hg.1	0,006993007	0,044547453
UBAP2L	TC01004756.hg.1	0,000582751	0,010863718
HMOX2	TC16000131.hg.1	0,001165501	0,015073094
CABIN1	TC22000144.hg.1	0,006993007	0,044547453
NCL	TC02002859.hg.1	0,002331002	0,022134595
POLM	TC07001333.hg.1	0,004079254	0,031319232
ARHGEF18	TC19000125.hg.1	0,021307711	0,094939392
UNC45A	TC15000873.hg.1	0,006993007	0,044547453
RAP1GAP2	TC17000026.hg.1	0,002331002	0,022134595
MTDH	TC08000594.hg.1	0,011072261	0,058833921
SETD1A	TC16000364.hg.1	0,005956016	0,044547453
MAPKBP1	TC15000313.hg.1	0,000582751	0,010863718
OMA1	TC01006360.hg.1	0,007226222	0,045616039
ZNF527	TC19000515.hg.1	0,011072261	0,058833921
CTIF	TC18000173.hg.1	0,012618408	0,06685837
PRR12	TC19000741.hg.1	0,021307711	0,094939392
PSME3	TC17000538.hg.1	0,017482517	0,079419358
TMEM159	TC16000227.hg.1	0,004079254	0,031319232
FLYWCH1	TC16000089.hg.1	0,006993007	0,044547453
CHMP4A	TC14002341.hg.1	0,007035599	0,044614821
RANBP10	TC16001196.hg.1	0,004079254	0,031319232
CDIPT	TC16001011.hg.1	0,017482517	0,079419358
SARS2	TC19002700.hg.1	0,015082035	0,079010584
LEPREL4	TC17001517.hg.1	0,006993007	0,044547453
NEU1	TC06001548.hg.1	0,017482517	0,079419358
MNT	TC17000994.hg.1	0,008734438	0,054888112
TUBB	TC06000347.hg.1	0,011072261	0,058833921
YBX3	TC12001227.hg.1	0,011072261	0,058833921
DYNLL1	TC12000946.hg.1	0,011072261	0,058833921
EFEMP1	TC02001867.hg.1	0,011072261	0,058833921
TRAF4	TC17000330.hg.1	0,004079254	0,031319232
PISD	TC22001489.hg.1	0,006993007	0,044547453
BRD9	TC05003404.hg.1	0,002068779	0,022134595
SZT2	TC01000547.hg.1	0,006993007	0,044547453
CASC4	TC15000338.hg.1	0,011072261	0,058833921
TRIM26	TC06001499.hg.1	0,006993007	0,044547453
BLCAP	TC20000826.hg.1	0,017482517	0,079419358
TSC2	TC16000059.hg.1	0,017959832	0,081059195
DVL2	TC17001079.hg.1	0,000582751	0,010863718
WBSCR16	TC07001515.hg.1	0,017482517	0,079419358
FOXRED1	TC11001170.hg.1	0,001165501	0,015073094
RTN3	TC11000575.hg.1	0,002331002	0,022134595
ALG12	TC22000904.hg.1	0,004079254	0,031319232
C1QBP	TC17001056.hg.1	0,011072261	0,058833921
SNX3	TC06001994.hg.1	0,004079254	0,031319232
UROS	TC10001739.hg.1	0,003158907	0,02989431
PIGV	TC01000350.hg.1	0,011072261	0,058833921
PCYOX1L	TC05000819.hg.1	0,004079254	0,031319232
ANKS6	TC09001408.hg.1	0,011072261	0,058833921
SDF2L1	TC22000095.hg.1	0,011072261	0,058833921

RAB8A	TC19002017.hg.1	0,017482517	0,079419358
ZKSCAN4	TC06001437.hg.1	0,017482517	0,079419358
SLC4A8	TC12000406.hg.1	0,008734438	0,054888112
ERBB2	TC17000480.hg.1	0,006993007	0,044547453
ARPP19	TC15001457.hg.1	0,002331002	0,022134595
GNS	TC12001678.hg.1	0,002331002	0,022134595
CHEK2	TC22000626.hg.1	0,001165501	0,015073094
YIF1B	TC19001497.hg.1	0,017482517	0,079419358
ATXN10	TC22000385.hg.1	0,011072261	0,058833921
CHCHD4	TC03001193.hg.1	0,017482517	0,079419358
IFFO1	TC12001141.hg.1	0,001165501	0,015073094
SLC12A2	TC05000612.hg.1	0,011072261	0,058833921
GFOD2	TC16001195.hg.1	0,004079254	0,031319232
KIAA0556	TC16000295.hg.1	0,004079254	0,031319232
KLF16	TC19001015.hg.1	0,001165501	0,015073094
EML2	TC19002522.hg.1	0,002331002	0,022134595
TASP1	TC20000636.hg.1	0,006993007	0,044547453
LIPE	TC19001573.hg.1	0,006993007	0,044547453
ARRB2	TC17000047.hg.1	0,020880821	0,093862657
TMEM110	TC03003414.hg.1	0,000582751	0,010863718
DDRGK1	TC20000570.hg.1	0,001165501	0,015073094
TDRP	TC08000880.hg.1	0,017482517	0,079419358
SLC25A25	TC09000675.hg.1	0,001165501	0,015073094
TNRC6C	TC17002389.hg.1	0,002331002	0,022134595
OCIAD1	TC04000281.hg.1	0,004000668	0,031319232
CEP250	TC20000253.hg.1	0,001165501	0,015073094
FANCC	TC09001361.hg.1	0,006993007	0,044547453
METTL1	TC12001635.hg.1	0,004079254	0,031319232
EVI5L	TC19000138.hg.1	0,002331002	0,022134595
COX10	TC17000162.hg.1	0,011072261	0,058833921
HADH	TC04000556.hg.1	0,017482517	0,079419358
LAMP1	TC13000421.hg.1	0,011072261	0,058833921
HNRNPA3	TC02001062.hg.1	0,017482517	0,079419358
DHX37	TC12002105.hg.1	0,011072261	0,058833921
TEX264	TC03003354.hg.1	0,000582751	0,010863718
ZNF639	TC03000952.hg.1	0,004079254	0,031319232
FITM2	TC20000862.hg.1	0,017482517	0,079419358
SLC11A2	TC12001499.hg.1	0,015082035	0,079010584
C7orf55	TC07000872.hg.1	0,00214071	0,022134595
C7orf55-LUC7L2	TC07000872.hg.1	0,00214071	0,022134595
LUC7L2	TC07000872.hg.1	0,00214071	0,022134595
GAS8	TC16001722.hg.1	0,000582751	0,010863718
MRPL20	TC01002096.hg.1	0,017959832	0,081059195
ZNF782	TC09001379.hg.1	0,001165501	0,015073094
ZNF790	TC19001472.hg.1	0,000582751	0,010863718
IRF9	TC14000161.hg.1	0,000582751	0,010863718
RNF31	TC14000161.hg.1	0,000582751	0,010863718
C19orf12	TC19001381.hg.1	0,006993007	0,044547453
STX10	TC19001219.hg.1	0,017959832	0,081059195
TPK1	TC07001982.hg.1	0,017482517	0,079419358

TERF1	TC08000479.hg.1	0,002331002	0,022134595
ZADH2	TC18000578.hg.1	0,011072261	0,058833921
RBBP4	TC01000433.hg.1	0,000582751	0,010863718
RAB40B	TC17001985.hg.1	0,020880821	0,093862657
IFT122	TC03000692.hg.1	0,007035599	0,044614821
CCS	TC11000672.hg.1	0,004079254	0,031319232
PRKRIR	TC11002110.hg.1	0,017482517	0,079419358
PILRB	TC07003313.hg.1	0,003260294	0,030697427
NR2F6	TC19001270.hg.1	0,010517951	0,058833921
ENTPD5	TC14001302.hg.1	0,015082035	0,079010584
GRSF1	TC04001272.hg.1	0,017482517	0,079419358
TBC1D22A	TC22000399.hg.1	0,006993007	0,044547453
SLC36A1	TC05000840.hg.1	0,004000668	0,031319232
CALM1	TC19000665.hg.1	0,000582751	0,010863718
METTL21B	TC12003249.hg.1	0,011072261	0,058833921
C17orf80	TC17000823.hg.1	0,021307711	0,094939392
PACS2	TC14000834.hg.1	0,017482517	0,079419358
RAD51D	TC17002897.hg.1	0,004079254	0,031319232
USP30	TC12000841.hg.1	0,000582751	0,010863718
LYAR	TC04000989.hg.1	0,017482517	0,079419358
ZNF445	TC03001337.hg.1	0,001165501	0,015073094
DEPDC5	TC22000232.hg.1	0,000582751	0,010863718
FUZ	TC19002546.hg.1	0,011072261	0,058833921
ZNF619	TC03000210.hg.1	0,004079254	0,031319232
NUFIP2	TC17001316.hg.1	0,011072261	0,058833921
RGL2	TC06001583.hg.1	0,005956016	0,044547453
SUPT20H	TC13000571.hg.1	0,004079254	0,031319232
WDR46	TC06001582.hg.1	0,006993007	0,044547453
USP36	TC17001922.hg.1	0,004079254	0,031319232
TOP2B	TC03001247.hg.1	0,011072261	0,058833921
RING1	TC06000415.hg.1	0,011072261	0,058833921
LARP1	TC05000855.hg.1	0,004079254	0,031319232
PROCR	TC20000248.hg.1	0,017482517	0,079419358
ZNF774	TC15000863.hg.1	0,017959832	0,081059195
UBAP2L	TC01001267.hg.1	0,001165501	0,015073094
PDIA4	TC07001995.hg.1	0,017482517	0,079419358
NCBP2	TC03002170.hg.1	0,006993007	0,044547453
DNAJC16	TC01000190.hg.1	0,004079254	0,031319232
CEP170	TC01004038.hg.1	0,006993007	0,044547453
EFCAB14	TC01006356.hg.1	0,011072261	0,058833921
FAHD1	TC16000049.hg.1	0,011072261	0,058833921
SMARCA5	TC04000701.hg.1	0,002331002	0,022134595
BCAT2	TC19001697.hg.1	0,017482517	0,079419358
ABHD11	TC07001505.hg.1	0,000582751	0,010863718
SCAP	TC03001367.hg.1	0,010263769	0,058833921
TMCC1	TC03001773.hg.1	0,017959832	0,081059195
RCN2	TC15000709.hg.1	0,017482517	0,079419358
DUSP7	TC03001445.hg.1	0,002331002	0,022134595
CAMKK2	TC12002061.hg.1	0,011072261	0,058833921
AK9	TC06002005.hg.1	0,015082035	0,079010584

MOGS	TC02001997.hg.1	0,011072261	0,058833921
MED27	TC09001671.hg.1	0,017482517	0,079419358
SLC9A3R1	TC17000834.hg.1	0,017482517	0,079419358
AKAP8	TC19001253.hg.1	0,017482517	0,079419358
POLRMT	TC19000987.hg.1	0,002646872	0,02509127
LSM14B	TC20000482.hg.1	0,017959832	0,081059195
KPNB1	TC17000611.hg.1	0,017482517	0,079419358
LSM2	TC06001545.hg.1	0,011072261	0,058833921
NDUFS2	TC01001381.hg.1	0,002331002	0,022134595
MIF4GD	TC17001875.hg.1	0,011072261	0,058833921
VARS	TC06001543.hg.1	0,006993007	0,044547453
PQLC3	TC02000069.hg.1	0,002331002	0,022134595
ZNF512B	TC20001043.hg.1	0,000582751	0,010863718
CEP170	TC04000603.hg.1	0,011072261	0,058833921
BTBD9	TC06001690.hg.1	0,001165501	0,015073094
ARHGEF7	TC13000398.hg.1	0,001165501	0,015073094
CNOT7	TC08001011.hg.1	0,017482517	0,079419358
DDB1	TC11001851.hg.1	0,004079254	0,031319232
TIAL1	TC10001705.hg.1	0,011072261	0,058833921
EIF2B4	TC02001683.hg.1	0,001165501	0,015073094
CEP19	TC03002165.hg.1	0,017482517	0,079419358
PIP5K1A	TC01001204.hg.1	0,011072261	0,058833921
GANC	TC15002792.hg.1	0,011072261	0,058833921
PTPN14	TC01003817.hg.1	0,017482517	0,079419358
TP53BP1	TC15002510.hg.1	0,002331002	0,022134595
TFDP2	TC03001849.hg.1	0,017482517	0,079419358
PHF20	TC20000260.hg.1	0,011072261	0,058833921
UBE3D	TC06001910.hg.1	0,004750313	0,036421244
PIGL	TC17000186.hg.1	0,006993007	0,044547453
BRE	TC02004954.hg.1	0,017482517	0,079419358
MKKS	TC20000619.hg.1	0,011072261	0,058833921
GPI	TC19000442.hg.1	0,006993007	0,044547453
ZSWIM7	TC17001170.hg.1	0,001165501	0,015073094
DHFRL1	TC03001592.hg.1	0,011072261	0,058833921
PPP1R26	TC09000795.hg.1	0,000582751	0,010863718
RNF4	TC04002910.hg.1	0,017482517	0,079419358
BAX	TC19000716.hg.1	0,006993007	0,044547453
L3MBTL2	TC22000334.hg.1	0,017482517	0,079419358
MKNK1	TC01006353.hg.1	0,00214071	0,022134595
NDUFV3	TC21001063.hg.1	0,011072261	0,058833921
RBM19	TC12001993.hg.1	0,004079254	0,031319232
BCAR1	TC16001276.hg.1	0,017482517	0,079419358
TMEM254	TC10000575.hg.1	0,011072261	0,058833921
UBE2V1	TC20001751.hg.1	0,004079254	0,031319232
TUSC2	TC03003330.hg.1	0,002331002	0,022134595
ZDHHC14	TC06001124.hg.1	0,006993007	0,044547453
HNRNPUL1	TC19000577.hg.1	0,002331002	0,022134595
LAMB1	TC07001747.hg.1	0,002331002	0,022134595
ATF7IP	TC12000193.hg.1	0,002331002	0,022134595
PPIL2	TC22000096.hg.1	0,000582751	0,010863718

PHF12	TC17001313.hg.1	0,011072261	0,058833921
RNF216	TC07001121.hg.1	0,002331002	0,022134595
ARL4D	TC17000551.hg.1	0,004079254	0,031319232
ZNF619	TC03002299.hg.1	0,006993007	0,044547453
ZC3H4	TC19001660.hg.1	0,000582751	0,010863718
PDE4A	TC19001980.hg.1	0,017482517	0,079419358
PRR13	TC12003241.hg.1	0,011072261	0,058833921
POLDIP2	TC17001295.hg.1	0,011072261	0,058833921
DCAF8	TC01006277.hg.1	0,011072261	0,058833921
ZNF846	TC19001153.hg.1	0,017482517	0,079419358
NGLY1	TC03001248.hg.1	0,001165501	0,015073094
DTD1	TC20000127.hg.1	0,011072261	0,058833921
ERCC6L2	TC09000475.hg.1	0,017482517	0,079419358
SNAPC3	TC09000075.hg.1	0,017482517	0,079419358
ALG14	TC01002892.hg.1	0,011072261	0,058833921
NKIRAS1	TC03001239.hg.1	0,002331002	0,022134595
SON	TC21000130.hg.1	0,017482517	0,079419358
SDCCAG3	TC09001730.hg.1	0,006993007	0,044547453
F2R	TC05000370.hg.1	0,004079254	0,031319232
ARMC10	TC07000671.hg.1	0,017482517	0,079419358
HSD17B4	TC05000567.hg.1	0,006993007	0,044547453
ZNF625	TC19002683.hg.1	0,017482517	0,079419358
ZNF625-ZNF20	TC19002683.hg.1	0,017482517	0,079419358
MBNL1	TC03000832.hg.1	0,002331002	0,022134595
EID1	TC15000381.hg.1	0,002331002	0,022134595
NAA40	TC11000579.hg.1	0,004079254	0,031319232
ICA1	TC07001143.hg.1	0,017482517	0,079419358
SPG20	TC13000566.hg.1	0,004079254	0,031319232
CAMKMT	TC02000276.hg.1	0,006993007	0,044547453
GTF3A	TC13000077.hg.1	0,004079254	0,031319232
TMEM241	TC18000412.hg.1	0,004079254	0,031319232
GPATCH4	TC01003344.hg.1	0,017482517	0,079419358
SPA17	TC11001142.hg.1	0,006993007	0,044547453
UBE2Q1	TC01003293.hg.1	0,000582751	0,010863718
ADD1	TC04000039.hg.1	0,002331002	0,022134595
RBM25	TC14000438.hg.1	0,010517951	0,058833921
XPNPEP3	TC22000327.hg.1	0,011072261	0,058833921
NIF3L1	TC02001168.hg.1	0,017482517	0,079419358
ZNF248	TC10001187.hg.1	0,002331002	0,022134595
ATP6V1H	TC08001215.hg.1	0,017482517	0,079419358
EIF2B3	TC01002603.hg.1	0,006993007	0,044547453
ACAP2	TC03002141.hg.1	0,000582751	0,010863718
ANAPC10	TC04001608.hg.1	0,011072261	0,058833921
METTL16	TC17000996.hg.1	0,006993007	0,044547453
SERPINB6	TC06001221.hg.1	0,002331002	0,022134595
PPIL3	TC02002668.hg.1	0,021307711	0,094939392
TXNDC15	TC05000669.hg.1	0,017482517	0,079419358
KIF2A	TC05003396.hg.1	0,001165501	0,015073094
CCDC134	TC22000345.hg.1	0,006993007	0,044547453
FKBP15	TC09002916.hg.1	0,011072261	0,058833921

REPIN1	TC07003295.hg.1	0,00214071	0,022134595
PMPCA	TC09000812.hg.1	0,011072261	0,058833921
SNRNP70	TC19000724.hg.1	0,001165501	0,015073094
UGGT1	TC02000791.hg.1	0,002331002	0,022134595
SLC25A11	TC17001035.hg.1	0,006993007	0,044547453
SLC35E3	TC12000604.hg.1	0,001165501	0,015073094
FAM213A	TC10000581.hg.1	0,002331002	0,022134595
LIG3	TC17000391.hg.1	0,000582751	0,010863718
XXYLT1	TC03002140.hg.1	0,006993007	0,044547453
ZNF554	TC19000058.hg.1	0,000582751	0,010863718
GLOD4	TC17000968.hg.1	0,017482517	0,079419358
NPRL3	TC16000722.hg.1	0,002331002	0,022134595
SEC61A2	TC10000090.hg.1	0,000582751	0,010863718
COPG1	TC03000689.hg.1	0,017482517	0,079419358
CHTF8	TC16001218.hg.1	0,011072261	0,058833921
MED4	TC13000653.hg.1	0,011072261	0,058833921
ST3GAL3	TC01000550.hg.1	0,000582751	0,010863718
TRIM14	TC09001403.hg.1	0,017482517	0,079419358
NEIL1	TC15000695.hg.1	0,011072261	0,058833921
SNX27	TC01001214.hg.1	0,006993007	0,044547453
DGCR2	TC22000490.hg.1	0,017482517	0,079419358
PRR3	TC06000340.hg.1	0,000582751	0,010863718
RALB	TC02000760.hg.1	0,001165501	0,015073094
UHMK1	TC01001421.hg.1	0,006993007	0,044547453
NUDT7	TC16000628.hg.1	0,002331002	0,022134595
MIPEP	TC13000484.hg.1	0,004079254	0,031319232
VWA9	TC15001570.hg.1	0,017482517	0,079419358
CABLES2	TC20001009.hg.1	0,002331002	0,022134595
MON2	TC12000556.hg.1	0,017482517	0,079419358
SHPK	TC17002848.hg.1	0,001165501	0,015073094
IARS2	TC01001814.hg.1	0,006993007	0,044547453
AMACR	TC05003422.hg.1	0,011072261	0,058833921
MRPS18B	TC06000343.hg.1	0,017482517	0,079419358
ZBTB16	TC11001026.hg.1	0,017482517	0,079419358
RBM27	TC05000797.hg.1	0,006993007	0,044547453
TTC19	TC17000181.hg.1	0,000582751	0,010863718
KLHL22	TC22001224.hg.1	0,001165501	0,015073094
CSPP1	TC08000447.hg.1	0,004079254	0,031319232
TARS2	TC01001187.hg.1	0,011072261	0,058833921
VTI1A	TC10000814.hg.1	0,017482517	0,079419358
FLII	TC17001199.hg.1	0,011072261	0,058833921
MARCH2	TC19000150.hg.1	0,011072261	0,058833921
KDM1A	TC01000292.hg.1	0,001165501	0,015073094
FBXO31	TC16001330.hg.1	0,017482517	0,079419358
UGGT1	TC02003566.hg.1	0,002331002	0,022134595
ABI2	TC02001198.hg.1	0,002331002	0,022134595
SH3PXD2B	TC05002062.hg.1	0,002331002	0,022134595
EXOC7	TC17001893.hg.1	0,011072261	0,058833921
RANBP3	TC19001087.hg.1	0,000582751	0,010863718
ZMYM1	TC01000454.hg.1	0,001165501	0,015073094

IFT74	TC09000119.hg.1	0,006993007	0,044547453
ACOT1	TC14002200.hg.1	0,006993007	0,044547453
SRSF4	TC01002432.hg.1	0,000582751	0,010863718
TBC1D14	TC04000075.hg.1	0,000582751	0,010863718
ZNF616	TC19001796.hg.1	0,017482517	0,079419358
DCLRE1A	TC10001673.hg.1	0,000582751	0,010863718
ASXL2	TC02001663.hg.1	0,004079254	0,031319232
ZBTB49	TC04000057.hg.1	0,004079254	0,031319232
NHP2L1	TC22000831.hg.1	0,00214071	0,022134595
TBC1D31	TC08000712.hg.1	0,017482517	0,079419358
DBNL	TC07003393.hg.1	0,002331002	0,022134595
NUDC	TC01000355.hg.1	0,004890624	0,03744552
SMYD4	TC17000984.hg.1	0,001165501	0,015073094
ALKBH5	TC17000207.hg.1	0,017482517	0,079419358
BBX	TC03000543.hg.1	0,002331002	0,022134595
AP3B1	TC05001513.hg.1	0,011072261	0,058833921
ZNF106	TC15001264.hg.1	0,017482517	0,079419358
TLDC1	TC16001313.hg.1	0,006993007	0,044547453
ZNF8	TC19002670.hg.1	0,017482517	0,079419358
MFSD8	TC04001535.hg.1	0,002331002	0,022134595
RETSAT	TC02002037.hg.1	0,006993007	0,044547453
KIAA1586	TC06000682.hg.1	0,000582751	0,010863718
PAGR1	TC16002070.hg.1	0,000582751	0,010863718
INPP4A	TC02000599.hg.1	0,006993007	0,044547453
LASP1	TC17000469.hg.1	0,002331002	0,022134595
PIM3	TC22000416.hg.1	0,017482517	0,079419358
METTL17	TC14000074.hg.1	0,011072261	0,058833921
RBL2	TC16000448.hg.1	0,011072261	0,058833921
MTIF2	TC02001859.hg.1	0,011072261	0,058833921
PRKACB	TC01000808.hg.1	0,004079254	0,031319232
EIF2AK1	TC07001126.hg.1	0,000582751	0,010863718
PFN2	TC03003395.hg.1	0,006993007	0,044547453
MMS19	TC10001567.hg.1	0,017959832	0,081059195
FAM57A	TC17000003.hg.1	0,011072261	0,058833921
SNX2	TC05000576.hg.1	0,006993007	0,044547453
NAA16	TC13000145.hg.1	0,017482517	0,079419358
GRB2	TC17001877.hg.1	0,011072261	0,058833921
RNF20	TC09000519.hg.1	0,021307711	0,094939392
MLYCD	TC16000651.hg.1	0,017482517	0,079419358
ILF3	TC19000182.hg.1	0,017482517	0,079419358
CLPX	TC15001561.hg.1	0,000582751	0,010863718
RNPEP	TC01001669.hg.1	0,011072261	0,058833921
SLC25A23	TC19001097.hg.1	0,004079254	0,031319232
ZHX1	TC08002619.hg.1	0,017482517	0,079419358
BCAS3	TC17000740.hg.1	0,017482517	0,079419358
CNOT11	TC02000615.hg.1	0,001165501	0,015073094
ZNF347	TC19001813.hg.1	0,011072261	0,058833921
LMLN	TC03003369.hg.1	0,006993007	0,044547453
B4GALT5	TC20000928.hg.1	0,001165501	0,015073094
CLCN3	TC04000845.hg.1	0,011072261	0,058833921

TINF2	TC14000970.hg.1	0,017482517	0,079419358
ICK	TC06001809.hg.1	0,011072261	0,058833921
SGSH	TC17001934.hg.1	0,002331002	0,022134595
ZNF740	TC12000432.hg.1	0,017482517	0,079419358
TIMM10B	TC11003443.hg.1	0,011072261	0,058833921
MED20	TC06001726.hg.1	0,004079254	0,031319232
ARHGAP26	TC05000782.hg.1	0,002331002	0,022134595
ORC4	TC02002408.hg.1	0,012618408	0,06685837
DHDDS	TC01000344.hg.1	0,001165501	0,015073094
CAPN7	TC03000092.hg.1	0,015082035	0,079010584
LMAN2L	TC02002111.hg.1	0,017482517	0,079419358
PARL	TC03002062.hg.1	0,017482517	0,079419358
CACYBP	TC01001526.hg.1	0,017482517	0,079419358
SLC4A1AP	TC02000180.hg.1	0,011072261	0,058833921
UTRN	TC06001059.hg.1	0,002331002	0,022134595
SHISA5	TC03001380.hg.1	0,000582751	0,010863718
PRPSAP2	TC17000223.hg.1	0,000582751	0,010863718
DDX20	TC01000981.hg.1	0,006993007	0,044547453
RMDN1	TC08001391.hg.1	0,004079254	0,031319232
NARG2	TC15001506.hg.1	0,006993007	0,044547453
BRD9	TC05002805.hg.1	0,006993007	0,044547453
CRYZL1	TC21001057.hg.1	0,004079254	0,031319232
SMAP2	TC01000510.hg.1	0,006993007	0,044547453
STXBP1	TC09000668.hg.1	0,020880821	0,093862657
ACP6	TC01003128.hg.1	0,011072261	0,058833921
TTBK2	TC15001270.hg.1	0,006993007	0,044547453
TCFL5	TC20001020.hg.1	0,000582751	0,010863718
WDR59	TC16001272.hg.1	0,004079254	0,031319232
TOX2	TC20000323.hg.1	0,006993007	0,044547453
OTUD6B	TC08000553.hg.1	0,017482517	0,079419358
RERE	TC01005249.hg.1	0,004079254	0,031319232
SCAP	TC03002894.hg.1	0,011072261	0,058833921
C16orf62	TC16000216.hg.1	0,017482517	0,079419358
TRAPPC12	TC02000019.hg.1	0,000582751	0,010863718
ERCC5	TC13001721.hg.1	0,004079254	0,031319232
PXN	TC12002037.hg.1	0,011072261	0,058833921
YLPM1	TC14000463.hg.1	0,002331002	0,022134595
AKAP2	TC09000550.hg.1	0,000582751	0,010863718
PALM2	TC09000550.hg.1	0,000582751	0,010863718
PALM2-AKAP2	TC09000550.hg.1	0,000582751	0,010863718
TMEM107	TC17001107.hg.1	0,002331002	0,022134595
PRPF8	TC17000981.hg.1	0,011072261	0,058833921
WDR26	TC01003883.hg.1	0,002331002	0,022134595
ANKS1A	TC06000510.hg.1	0,001165501	0,015073094
CRLS1	TC20000065.hg.1	0,006993007	0,044547453
PFAS	TC17000136.hg.1	0,006993007	0,044547453
BRI3BP	TC12001002.hg.1	0,001165501	0,015073094
ZNF652	TC17001662.hg.1	0,017959832	0,081059195
CDC16	TC13000435.hg.1	0,000582751	0,010863718
RHOT1	TC17000373.hg.1	0,017482517	0,079419358

NCBP1	TC09000492.hg.1	0,011072261	0,058833921
DDX49	TC19000366.hg.1	0,006993007	0,044547453
HOXB3	TC17002909.hg.1	0,000582751	0,010863718
HOXB4	TC17002909.hg.1	0,000582751	0,010863718
CCDC113	TC16000501.hg.1	0,001165501	0,015073094
SCAPER	TC15001688.hg.1	0,004079254	0,031319232
AVL9	TC07000207.hg.1	0,006993007	0,044547453
METTL13	TC01001496.hg.1	0,006993007	0,044547453
DPH5	TC01002924.hg.1	0,002331002	0,022134595
GNG11	TC07000556.hg.1	0,017482517	0,079419358
KIAA2013	TC01002207.hg.1	0,006993007	0,044547453
MKNK1	TC01005450.hg.1	0,002331002	0,022134595
GTDC1	TC02002398.hg.1	0,011072261	0,058833921
TCEANC2	TC01000661.hg.1	0,017482517	0,079419358
ERCC8	TC05001394.hg.1	0,011072261	0,058833921
MYO5A	TC15001456.hg.1	0,001165501	0,015073094
NAA15	TC04000683.hg.1	0,006993007	0,044547453
MBTPS1	TC16001307.hg.1	0,006993007	0,044547453
IQSEC1	TC03001187.hg.1	0,001165501	0,015073094
EHMT2	TC06001550.hg.1	0,000582751	0,010863718
PDK2	TC17000655.hg.1	0,000582751	0,010863718
TP53BP1	TC15001278.hg.1	0,000582751	0,010863718
TAMM41	TC03001167.hg.1	0,001165501	0,015073094
SMARCAL1	TC02001278.hg.1	0,002331002	0,022134595
AGPAT4	TC06002284.hg.1	0,007035599	0,044614821
RNF34	TC12000958.hg.1	0,006993007	0,044547453
ZMYM1	TC01004312.hg.1	0,002331002	0,022134595
METTL2B	TC07003294.hg.1	0,004079254	0,031319232
LIFR	TC05001289.hg.1	0,006993007	0,044547453
ARHGDIA	TC17001961.hg.1	0,002331002	0,022134595
C1orf85	TC01003335.hg.1	0,004079254	0,031319232
AKAP1	TC17000704.hg.1	0,000582751	0,010863718
RABGAP1	TC09000627.hg.1	0,006993007	0,044547453
GDE1	TC16000918.hg.1	0,011072261	0,058833921
VTI1B	TC14002327.hg.1	0,017482517	0,079419358
BAX	TC19002194.hg.1	0,011072261	0,058833921
TNRC6A	TC16000264.hg.1	0,001165501	0,015073094
AKAP8L	TC19001254.hg.1	0,000582751	0,010863718
TFAP4	TC16001756.hg.1	0,000582751	0,010863718
URI1	TC19000417.hg.1	0,002331002	0,022134595
TIMP2	TC17002807.hg.1	0,017482517	0,079419358
SPPL3	TC12002055.hg.1	0,006993007	0,044547453
NOP9	TC14002194.hg.1	0,002331002	0,022134595
ZNF546	TC19000558.hg.1	0,011072261	0,058833921
TSC1	TC09001678.hg.1	0,011072261	0,058833921
LDB1	TC10001617.hg.1	0,004079254	0,031319232
EBAG9	TC08000669.hg.1	0,010517951	0,058833921
FOXO1	TC13001719.hg.1	0,011072261	0,058833921
NDUFV2	TC18000041.hg.1	0,011072261	0,058833921
TIMMDC1	TC03000609.hg.1	0,011072261	0,058833921

APEH	TC03000297.hg.1	0,006993007	0,044547453
ACAD9	TC03000684.hg.1	0,017482517	0,079419358
L2HGDH	TC14001117.hg.1	0,000582751	0,010863718
NADK2	TC05001270.hg.1	0,011072261	0,058833921
METTL25	TC12000677.hg.1	0,017482517	0,079419358
SETDB2	TC13000198.hg.1	0,000582751	0,010863718
ZFP64	TC20000947.hg.1	0,000582751	0,010863718
SF3B3	TC16000579.hg.1	0,004079254	0,031319232
SEC14L1	TC17000884.hg.1	0,000582751	0,010863718
FAM161A	TC02004263.hg.1	0,002331002	0,022134595
PPP2R2A	TC08002591.hg.1	0,000582751	0,010863718
CHD6	TC20000850.hg.1	0,015082035	0,079010584
UCHL5	TC01003650.hg.1	0,017482517	0,079419358
APH1A	TC01003201.hg.1	0,017482517	0,079419358
ZNF346	TC05001008.hg.1	0,002331002	0,022134595
C4orf29	TC04000647.hg.1	0,006993007	0,044547453
TMEM19	TC12003254.hg.1	0,011072261	0,058833921
SLC44A1	TC09000535.hg.1	0,017482517	0,079419358
KLHL8	TC04001362.hg.1	0,006993007	0,044547453
CLCN6	TC01000149.hg.1	0,010263769	0,058833921
HIBADH	TC07001230.hg.1	0,017482517	0,079419358
MYO5A	TC15002540.hg.1	0,006993007	0,044547453
C12orf10	TC12000436.hg.1	0,017482517	0,079419358
NACC1	TC19000236.hg.1	0,002331002	0,022134595
MFF	TC02001362.hg.1	0,004079254	0,031319232
SSBP2	TC05001545.hg.1	0,010517951	0,058833921
UBR2	TC06000581.hg.1	0,004079254	0,031319232
RCOR3	TC01001757.hg.1	0,000582751	0,010863718
UBE3B	TC12000848.hg.1	0,017482517	0,079419358
PAXBP1	TC21000401.hg.1	0,004079254	0,031319232
TDRD3	TC13000240.hg.1	0,000582751	0,010863718
MORC2	TC22000660.hg.1	0,006993007	0,044547453
ALMS1	TC02000443.hg.1	0,000582751	0,010863718
POLR1C	TC06000603.hg.1	0,006993007	0,044547453
CCDC125	TC05001437.hg.1	0,001165501	0,015073094
GTF3C4	TC09000760.hg.1	0,006993007	0,044547453
PPP1R10	TC06001502.hg.1	0,000582751	0,010863718
C10orf25	TC10001217.hg.1	0,002331002	0,022134595
RNF167	TC17000057.hg.1	0,006993007	0,044547453
PPP1R9B	TC17001679.hg.1	0,004079254	0,031319232
IDE	TC10001531.hg.1	0,004079254	0,031319232
SLC16A13	TC17000084.hg.1	0,004079254	0,031319232
NFX1	TC09000140.hg.1	0,000582751	0,010863718
AMPD2	TC01000950.hg.1	0,000582751	0,010863718
PTPMT1	TC11003447.hg.1	0,011072261	0,058833921
MEPCE	TC07000620.hg.1	0,000582751	0,010863718
TMCC1	TC03003085.hg.1	0,006993007	0,044547453
DISP1	TC01006341.hg.1	0,002331002	0,022134595
SEPN1	TC01006315.hg.1	0,006993007	0,044547453
APH1A	TC01005811.hg.1	0,011072261	0,058833921

C2CD2	TC21000479.hg.1	0,011072261	0,058833921
NOL9	TC01002154.hg.1	0,017482517	0,079419358
PRPF6	TC20000531.hg.1	0,001165501	0,015073094
XRCC6BP1	TC12000547.hg.1	0,000582751	0,010863718
ALAS1	TC03000329.hg.1	0,017482517	0,079419358
RRP36	TC06000593.hg.1	0,011072261	0,058833921
SFXN3	TC10000731.hg.1	0,006993007	0,044547453
DGCR2	TC22001208.hg.1	0,006993007	0,044547453
EARS2	TC16000965.hg.1	0,000582751	0,010863718
MTA2	TC11001870.hg.1	0,004079254	0,031319232
DDX31	TC09001675.hg.1	0,004000668	0,031319232
PATZ1	TC22000668.hg.1	0,002331002	0,022134595
CEP85	TC01000339.hg.1	0,011072261	0,058833921
SMARCA2	TC09001792.hg.1	0,002331002	0,022134595
IFT88	TC13000025.hg.1	0,004079254	0,031319232
TGFBR2	TC03000149.hg.1	0,000582751	0,010863718
ALG6	TC01000719.hg.1	0,008734438	0,054888112
ATPAF1	TC01006355.hg.1	0,004079254	0,031319232
UCHL5	TC01006015.hg.1	0,017482517	0,079419358
ZNF174	TC16000123.hg.1	0,017482517	0,079419358
LARP4	TC12002357.hg.1	0,001165501	0,015073094
FAM188A	TC10001065.hg.1	0,001165501	0,015073094
PGS1	TC17000902.hg.1	0,000582751	0,010863718
MANBA	TC04001430.hg.1	0,006993007	0,044547453
RRP15	TC01005030.hg.1	0,006993007	0,044547453
XYLT2	TC17000663.hg.1	0,000582751	0,010863718
CCDC111	TC04000897.hg.1	0,002331002	0,022134595
TUBA4A	TC02002793.hg.1	0,001165501	0,015073094
TTC21B	TC02002495.hg.1	0,004079254	0,031319232
STX3	TC11000499.hg.1	0,017482517	0,079419358
NOL6	TC09001013.hg.1	0,004079254	0,031319232
PRDX6	TC01001510.hg.1	0,011072261	0,058833921
DCAF8	TC01005888.hg.1	0,007226222	0,045616039
ZZZ3	TC01002799.hg.1	0,000582751	0,010863718
REV3L	TC06002017.hg.1	0,006993007	0,044547453
ARHGAP1	TC11001723.hg.1	0,002331002	0,022134595
ACOX1	TC17001890.hg.1	0,011072261	0,058833921
MUM1	TC19000036.hg.1	0,002331002	0,022134595
MTIF3	TC13000510.hg.1	0,004079254	0,031319232
IL10RB	TC21001059.hg.1	0,000582751	0,010863718
AK2	TC01002479.hg.1	0,006993007	0,044547453
ANKMY2	TC07001161.hg.1	0,001165501	0,015073094
AP1B1	TC22000634.hg.1	0,004079254	0,031319232
JARID2	TC06000104.hg.1	0,006993007	0,044547453
TRMT1L	TC01003627.hg.1	0,004079254	0,031319232
STAU2	TC08002586.hg.1	0,004079254	0,031319232
TEX264	TC03002339.hg.1	0,004079254	0,031319232
GLDC	TC09000899.hg.1	0,011072261	0,058833921
TOP1	TC20000302.hg.1	0,000582751	0,010863718
ALG1	TC16000139.hg.1	0,006993007	0,044547453

PEX11B	TC01001094.hg.1	0,010263769	0,058833921
PAXBP1	TC21000925.hg.1	0,002331002	0,022134595
DDX10	TC11000981.hg.1	0,017482517	0,079419358
GATA4	TC08000089.hg.1	0,011072261	0,058833921
SCAI	TC09001585.hg.1	0,001165501	0,015073094
DDX55	TC12000989.hg.1	0,00214071	0,022134595
DTD2	TC14002319.hg.1	0,000582751	0,010863718
GCOM1	TC15000439.hg.1	0,001165501	0,015073094
MYZAP	TC15000439.hg.1	0,001165501	0,015073094
ASH2L	TC08000283.hg.1	0,001165501	0,015073094
UBIAD1	TC01000141.hg.1	0,004079254	0,031319232
PVRL3	TC03000561.hg.1	0,004079254	0,031319232
HSPA4	TC05000649.hg.1	0,000582751	0,010863718
LARP4	TC12000392.hg.1	0,001165501	0,015073094
SRSF9	TC12002045.hg.1	0,000582751	0,010863718
SHC1	TC01006381.hg.1	0,001165501	0,015073094
VPS13B	TC08000608.hg.1	0,017482517	0,079419358
SMARCD2	TC17001787.hg.1	0,002331002	0,022134595
DNAJA3	TC16000130.hg.1	0,011072261	0,058833921
FBXO31	TC16002016.hg.1	0,007226222	0,045616039
SNRNP70	TC19002196.hg.1	0,004079254	0,031319232
FDXACB1	TC11003342.hg.1	0,017482517	0,079419358
RAB11A	TC15000609.hg.1	0,000582751	0,010863718
EXOC2	TC06001207.hg.1	0,001165501	0,015073094
KIAA0753	TC17001064.hg.1	0,017482517	0,079419358
OSBPL1A	TC18000418.hg.1	0,001165501	0,015073094
MRP63	TC13000031.hg.1	0,011072261	0,058833921
RREB1	TC06000056.hg.1	0,000582751	0,010863718
NUP214	TC09000746.hg.1	0,000582751	0,010863718
HNRNPUL2	TC11003488.hg.1	0,001165501	0,015073094
KMT2A	TC11001064.hg.1	0,011072261	0,058833921
TOP1	TC20001221.hg.1	0,000582751	0,010863718
TBC1D5	TC03003337.hg.1	0,002331002	0,022134595
IMPACT	TC18000109.hg.1	0,001165501	0,015073094
MBOAT7	TC19001837.hg.1	0,004079254	0,031319232
ZNF70	TC22000566.hg.1	0,002331002	0,022134595
C14orf1	TC14001324.hg.1	0,011072261	0,058833921
UNG	TC12000843.hg.1	0,000582751	0,010863718
NUP62	TC19002608.hg.1	0,002331002	0,022134595
MRPS27	TC05001463.hg.1	0,002331002	0,022134595
VPS33A	TC12003287.hg.1	0,011072261	0,058833921
CDC25B	TC20000040.hg.1	0,017482517	0,079419358
ZBTB25	TC14001217.hg.1	0,000582751	0,010863718
ZFYVE21	TC14000807.hg.1	0,00214071	0,022134595
DDX18	TC02000742.hg.1	0,004079254	0,031319232
PHACTR4	TC01000379.hg.1	0,000582751	0,010863718
SEC24C	TC10000468.hg.1	0,006993007	0,044547453
EPN2	TC17000234.hg.1	0,001165501	0,015073094
TMEM218	TC11002416.hg.1	0,004000668	0,031319232
TMEM106B	TC07000093.hg.1	0,001165501	0,015073094

MBTPS1	TC16001988.hg.1	0,017482517	0,079419358
SLC2A1	TC01002578.hg.1	0,011072261	0,058833921
SFXN4	TC10001701.hg.1	0,001165501	0,015073094
INO80C	TC18000455.hg.1	0,001165501	0,015073094
SERPINH1	TC11000809.hg.1	0,006993007	0,044547453
NRP1	TC10001166.hg.1	0,000582751	0,010863718
C4orf27	TC04001726.hg.1	0,001165501	0,015073094
MGST2	TC04000687.hg.1	0,006993007	0,044547453
PIAS3	TC01001097.hg.1	0,000582751	0,010863718
KCNC4	TC01004613.hg.1	0,017482517	0,079419358
ARHGAP22	TC10001263.hg.1	0,000582751	0,010863718
CDC27	TC10001263.hg.1	0,000582751	0,010863718
TFAP4	TC16000827.hg.1	0,000582751	0,010863718
WBSCR16	TC07000467.hg.1	0,011072261	0,058833921
URB2	TC01001905.hg.1	0,002331002	0,022134595
THUMPD3	TC03000037.hg.1	0,006993007	0,044547453
BSCL2	TC11003186.hg.1	0,002331002	0,022134595
THEM4	TC01003238.hg.1	0,000582751	0,010863718
GLRX	TC05001608.hg.1	0,000582751	0,010863718
MRPL15	TC08000374.hg.1	0,000582751	0,010863718
APC	TC05003398.hg.1	0,004079254	0,031319232
NOL11	TC17000795.hg.1	0,017482517	0,079419358
HELZ	TC17001812.hg.1	0,000582751	0,010863718
DHTKD1	TC10000089.hg.1	0,001165501	0,015073094
MTR	TC01001956.hg.1	0,004079254	0,031319232
SYNRG	TC17001408.hg.1	0,002331002	0,022134595
PHLDB2	TC03003362.hg.1	0,002331002	0,022134595
PLCG1	TC20000303.hg.1	0,002331002	0,022134595
NSUN6	TC10002961.hg.1	0,011072261	0,058833921
ARHGAP17	TC16000972.hg.1	0,011072261	0,058833921
AMZ2	TC17000801.hg.1	0,001165501	0,015073094
RCBTB1	TC13000661.hg.1	0,011072261	0,058833921
SLC25A37	TC08000188.hg.1	0,000582751	0,010863718
U2AF2	TC19000906.hg.1	0,017482517	0,079419358
SLC43A1	TC11001793.hg.1	0,002331002	0,022134595
NR1D1	TC17001459.hg.1	0,000582751	0,010863718
GUF1	TC04000268.hg.1	0,017482517	0,079419358
DHX33	TC17001057.hg.1	0,002331002	0,022134595
DGKA	TC12000495.hg.1	0,00214071	0,022134595
SH3BP5	TC03001207.hg.1	0,000582751	0,010863718
MIA3	TC01001829.hg.1	0,000582751	0,010863718
TTF2	TC01001026.hg.1	0,006993007	0,044547453
EHD2	TC19000681.hg.1	0,004079254	0,031319232
RMND1	TC06002235.hg.1	0,001165501	0,015073094
PGRMC2	TC04001537.hg.1	0,000582751	0,010863718
CLUAP1	TC16000125.hg.1	0,006993007	0,044547453
GDF11	TC12000490.hg.1	0,006993007	0,044547453
REPS1	TC06002164.hg.1	0,000582751	0,010863718
FAM86A	TC16000842.hg.1	0,002331002	0,022134595
LSG1	TC03002137.hg.1	0,002331002	0,022134595

NBAS	TC02001592.hg.1	0,011072261	0,058833921
GCA	TC02000978.hg.1	0,003260294	0,030697427
LMLN	TC03002771.hg.1	0,011072261	0,058833921
ADSS	TC01004046.hg.1	0,000582751	0,010863718
THUMPD2	TC02001768.hg.1	0,001165501	0,015073094
TGFBR2	TC03002268.hg.1	0,000582751	0,010863718
TGM2	TC20000833.hg.1	0,011072261	0,058833921
RORA	TC15001507.hg.1	0,011072261	0,058833921
ZNF507	TC19000425.hg.1	0,011072261	0,058833921
SLC25A15	TC13000140.hg.1	0,001165501	0,015073094
CETN3	TC05003139.hg.1	0,006993007	0,044547453
ALKBH2	TC12001947.hg.1	0,002331002	0,022134595
RRP15	TC01001804.hg.1	0,004079254	0,031319232
GPAM	TC10001663.hg.1	0,004079254	0,031319232
VPRBP	TC03001436.hg.1	0,006993007	0,044547453
AAK1	TC02001950.hg.1	0,001165501	0,015073094
ATP6V0A1	TC17000525.hg.1	0,017482517	0,079419358
LYRM5	TC12000252.hg.1	0,004079254	0,031319232
FARSB	TC02002813.hg.1	0,011072261	0,058833921
RALGDS	TC09002922.hg.1	0,000582751	0,010863718
SNX17	TC02000173.hg.1	0,021307711	0,094939392
STAT2	TC12001602.hg.1	0,001165501	0,015073094
HDDC3	TC15001855.hg.1	0,002331002	0,022134595
INTS9	TC08001094.hg.1	0,001165501	0,015073094
ZFP36L1	TC14001253.hg.1	0,017482517	0,079419358
GNL3	TC03000339.hg.1	0,002331002	0,022134595
SDCCAG8	TC01001981.hg.1	0,006993007	0,044547453
CES2	TC16000524.hg.1	0,001165501	0,015073094
TTPAL	TC20000333.hg.1	0,011072261	0,058833921
PDCD2L	TC19000443.hg.1	0,001165501	0,015073094
MGME1	TC20000114.hg.1	0,011072261	0,058833921
FBXW8	TC12000920.hg.1	0,002331002	0,022134595
WDFY3	TC04001355.hg.1	0,004079254	0,031319232
PIGM	TC01003395.hg.1	0,002331002	0,022134595
FOCAD	TC09000099.hg.1	0,00214071	0,022134595
ZNF77	TC19001039.hg.1	0,006993007	0,044547453
MSI2	TC17000705.hg.1	0,004079254	0,031319232
PRKCDBP	TC11001354.hg.1	0,004079254	0,031319232
TDRKH	TC01003229.hg.1	0,000582751	0,010863718
RNF214	TC11001050.hg.1	0,001165501	0,015073094
RPTOR	TC17000921.hg.1	0,001165501	0,015073094
ZSCAN30	TC18000452.hg.1	0,000582751	0,010863718
NAV1	TC01001661.hg.1	0,002331002	0,022134595
RNF138	TC18000133.hg.1	0,004079254	0,031319232
PSIP1	TC09000925.hg.1	0,001165501	0,015073094
DHX36	TC03001919.hg.1	0,001165501	0,015073094
MUM1	TC19001933.hg.1	0,006993007	0,044547453
ZNF608	TC05001731.hg.1	0,004079254	0,031319232
ICMT	TC01002147.hg.1	0,006993007	0,044547453
DMXL2	TC15001441.hg.1	0,002331002	0,022134595

VPS18	TC15000297.hg.1	0,002331002	0,022134595
VPS36	TC13000689.hg.1	0,000582751	0,010863718
SHANK3	TC22000435.hg.1	0,006993007	0,044547453
PHKB	TC16000427.hg.1	0,000582751	0,010863718
FASTKD2	TC02001221.hg.1	0,017482517	0,079419358
PNPT1	TC02001866.hg.1	0,000582751	0,010863718
PREX1	TC20000920.hg.1	0,011072261	0,058833921
ITSN1	TC21000131.hg.1	0,000582751	0,010863718
LRRC1	TC06000668.hg.1	0,000582751	0,010863718
INTS8	TC08000578.hg.1	0,004079254	0,031319232
ARHGAP29	TC01002884.hg.1	0,000582751	0,010863718
TAF15	TC17000402.hg.1	0,000582751	0,010863718
HEBP1	TC12001259.hg.1	0,001165501	0,015073094
LRP8	TC01002669.hg.1	0,011072261	0,058833921
MAVS	TC20000043.hg.1	0,011072261	0,058833921
NUFIP1	TC13000626.hg.1	0,000582751	0,010863718
GIT2	TC12001959.hg.1	0,000582751	0,010863718
DYSF	TC02000432.hg.1	0,017482517	0,079419358
NOP56	TC20000026.hg.1	0,002331002	0,022134595
MAGOHB	TC12001225.hg.1	0,000582751	0,010863718
GM2A	TC05000839.hg.1	0,001165501	0,015073094
PHF15	TC05000664.hg.1	0,000582751	0,010863718
MDM1	TC12001699.hg.1	0,000582751	0,010863718
GMCL1	TC02000409.hg.1	0,000582751	0,010863718
PCID2	TC13000892.hg.1	0,000582751	0,010863718
FAM84B	TC08001623.hg.1	0,000582751	0,010863718
RCHY1	TC04001298.hg.1	0,006993007	0,044547453
ARHGEF3	TC03001488.hg.1	0,000582751	0,010863718
HELZ	TC17002755.hg.1	0,000582751	0,010863718
GOLGA1	TC09001583.hg.1	0,000582751	0,010863718
AASDH	TC04001219.hg.1	0,003260294	0,030697427
DPF3	TC14001279.hg.1	0,000582751	0,010863718
SHANK3	TC22001183.hg.1	0,004079254	0,031319232
PFKM	TC12000346.hg.1	0,000582751	0,010863718
CSTF1	TC20000428.hg.1	0,006993007	0,044547453
POLA2	TC11000622.hg.1	0,011072261	0,058833921
ACAD11	TC03003341.hg.1	0,000582751	0,010863718
LRBA	TC04001634.hg.1	0,006993007	0,044547453
APH1B	TC15000582.hg.1	0,000582751	0,010863718
CES2	TC16001604.hg.1	0,001165501	0,015073094
CETN3	TC05001584.hg.1	0,004079254	0,031319232
TBC1D5	TC03002832.hg.1	0,002331002	0,022134595
DYNLL2	TC17000707.hg.1	0,002331002	0,022134595
NOP2	TC12001142.hg.1	0,006993007	0,044547453
MTHFR	TC01002202.hg.1	0,000582751	0,010863718
PIK3R3	TC01002616.hg.1	0,000582751	0,010863718
TTLL5	TC14002304.hg.1	0,000582751	0,010863718
MYO5A	TC15002203.hg.1	0,004079254	0,031319232
TOM1L2	TC17001194.hg.1	0,000582751	0,010863718
LONP2	TC16000430.hg.1	0,004079254	0,031319232

VPS11	TC11001082.hg.1	0,006993007	0,044547453
COG7	TC16000960.hg.1	0,004079254	0,031319232
RBMS2	TC12000514.hg.1	0,001165501	0,015073094
EXOC7	TC17002788.hg.1	0,017482517	0,079419358
GNPDA1	TC05001879.hg.1	0,001165501	0,015073094
HDHD2	TC18000492.hg.1	0,002331002	0,022134595
PARP1	TC01003876.hg.1	0,004079254	0,031319232
CUTC	TC10002946.hg.1	0,000582751	0,010863718
CTC1	TC17001111.hg.1	0,000582751	0,010863718
ANKFY1	TC17001019.hg.1	0,000582751	0,010863718
FJX1	TC11000337.hg.1	0,000582751	0,010863718
NAPEPLD	TC07001715.hg.1	0,00214071	0,022134595
USP41	TC22000519.hg.1	0,000582751	0,010863718
LBR	TC01003887.hg.1	0,015082035	0,079010584
FXN	TC09000318.hg.1	0,000582751	0,010863718
LSS	TC21000554.hg.1	0,011072261	0,058833921
MAVS	TC20001069.hg.1	0,002331002	0,022134595
NARS2	TC11002138.hg.1	0,001165501	0,015073094
IKBKAP	TC09001455.hg.1	0,001165501	0,015073094
PREPL	TC02001798.hg.1	0,000582751	0,010863718
METTL2A	TC17000756.hg.1	0,006993007	0,044547453
RRP1B	TC21000212.hg.1	0,017482517	0,079419358
LACTB2	TC08001313.hg.1	0,017482517	0,079419358
PLA2G12A	TC04001465.hg.1	0,006993007	0,044547453
SLC30A4	TC15001318.hg.1	0,006993007	0,044547453
DDX24	TC14001462.hg.1	0,004079254	0,031319232
ZNF462	TC09002163.hg.1	0,001165501	0,015073094
ARL15	TC05001345.hg.1	0,017482517	0,079419358
POLI	TC18000186.hg.1	0,001165501	0,015073094
WDR36	TC05000519.hg.1	0,004079254	0,031319232
IVD	TC15000284.hg.1	0,000582751	0,010863718
ENOPH1	TC04000454.hg.1	0,001165501	0,015073094
XPO6	TC16000983.hg.1	0,017482517	0,079419358
ABHD11	TC07003019.hg.1	0,000582751	0,010863718
SERPINB1	TC06001217.hg.1	0,011072261	0,058833921
DGKE	TC17000699.hg.1	0,005956016	0,044547453
MANSC1	TC12001253.hg.1	0,000582751	0,010863718
PTPN13	TC04000469.hg.1	0,004000668	0,031319232
EPAS1	TC02000281.hg.1	0,011072261	0,058833921
COL4A3BP	TC05001491.hg.1	0,004079254	0,031319232
KDM4C	TC09002929.hg.1	0,000582751	0,010863718
GTF3C1	TC16000979.hg.1	0,001165501	0,015073094
PARP9	TC03001705.hg.1	0,000582751	0,010863718
COG4	TC16001239.hg.1	0,006993007	0,044547453
SPG11	TC15001296.hg.1	0,000582751	0,010863718
DZIP1	TC13000804.hg.1	0,017482517	0,079419358
ORC2	TC02002669.hg.1	0,000582751	0,010863718
TANGO6	TC16000561.hg.1	0,000582751	0,010863718
RBM26	TC13000758.hg.1	0,000582751	0,010863718
DZIP3	TC03000548.hg.1	0,002331002	0,022134595

TYW1	TC07000414.hg.1	0,001165501	0,015073094
TUBGCP5	TC15000027.hg.1	0,000582751	0,010863718
C21orf59	TC21001072.hg.1	0,000582751	0,010863718
EIF2AK1	TC07002778.hg.1	0,001165501	0,015073094
COX15	TC10001584.hg.1	0,006993007	0,044547453
USP32	TC17001761.hg.1	0,002331002	0,022134595
DPH6	TC15001205.hg.1	0,017482517	0,079419358
SRSF1	TC17001723.hg.1	0,002331002	0,022134595
RBM26	TC13001592.hg.1	0,004079254	0,031319232
GCN1L1	TC12002034.hg.1	0,004079254	0,031319232
ORC5	TC07001724.hg.1	0,004079254	0,031319232
RPRD2	TC01001186.hg.1	0,000582751	0,010863718
ZNF462	TC09000544.hg.1	0,001165501	0,015073094
CHST15	TC10001725.hg.1	0,002331002	0,022134595
ELAC2	TC17001147.hg.1	0,000582751	0,010863718
CAMK2D	TC04002680.hg.1	0,004079254	0,031319232
ADAT2	TC06002186.hg.1	0,000582751	0,010863718
NBR1	TC17000548.hg.1	0,000582751	0,010863718
NUFIP1	TC13001465.hg.1	0,001165501	0,015073094
ELP4	TC11000303.hg.1	0,006993007	0,044547453
TBC1D8	TC02002143.hg.1	0,002331002	0,022134595
METTL23	TC17000879.hg.1	0,000582751	0,010863718
MON1B	TC16002059.hg.1	0,006993007	0,044547453
DDX24	TC14002132.hg.1	0,011072261	0,058833921
PEX1	TC07001601.hg.1	0,000582751	0,010863718
SLC23A2	TC20000588.hg.1	0,006993007	0,044547453
SMARCA2	TC09000013.hg.1	0,000582751	0,010863718
SYT11	TC01001306.hg.1	0,000582751	0,010863718
GMPR2	TC14000165.hg.1	0,004000668	0,031319232
RSBN1L	TC07000497.hg.1	0,00214071	0,022134595
TMEM107	TC17002476.hg.1	0,006993007	0,044547453
ATF6	TC01004799.hg.1	0,021307711	0,094939392
TTC28	TC22000624.hg.1	0,00214071	0,022134595
KMT2A	TC11002900.hg.1	0,002331002	0,022134595
NOTCH1	TC09001734.hg.1	0,000582751	0,010863718
ADAMTSL1	TC09000086.hg.1	0,011072261	0,058833921
THUMPD2	TC02004177.hg.1	0,002331002	0,022134595
EXOSC2	TC09000740.hg.1	0,004079254	0,031319232
ANAPC1	TC02002211.hg.1	0,000582751	0,010863718
AHNAK	TC11001867.hg.1	0,000582751	0,010863718
CCDC88C	TC14001436.hg.1	0,000582751	0,010863718
DPY19L3	TC19000426.hg.1	0,004079254	0,031319232
INSIG2	TC02000748.hg.1	0,000582751	0,010863718
COG1	TC17000822.hg.1	0,000582751	0,010863718
DGKE	TC17002290.hg.1	0,006993007	0,044547453
KAT2A	TC17001524.hg.1	0,000582751	0,010863718
USP5	TC12000095.hg.1	0,017482517	0,079419358
RPA1	TC17000016.hg.1	0,004079254	0,031319232
ATIC	TC02001260.hg.1	0,000582751	0,010863718
ESYT1	TC12000504.hg.1	0,002331002	0,022134595

C1QBP	TC17002457.hg.1	0,017482517	0,079419358
NMRK1	TC09001220.hg.1	0,011072261	0,058833921
KSR2	TC12003111.hg.1	0,002331002	0,022134595
RCHY1	TC04002599.hg.1	0,000582751	0,010863718
HPS3	TC03000803.hg.1	0,002331002	0,022134595
PTPN1	TC20000403.hg.1	0,000582751	0,010863718
PLIN3	TC19001072.hg.1	0,015082035	0,079010584
GCN1L1	TC12003120.hg.1	0,004079254	0,031319232
DHX29	TC05001362.hg.1	0,000582751	0,010863718
INTS10	TC08000148.hg.1	0,000582751	0,010863718
PRMT3	TC11000257.hg.1	0,005956016	0,044547453
OSGEPL1	TC02002615.hg.1	0,001165501	0,015073094
KIAA0586	TC14000351.hg.1	0,00214071	0,022134595
ANP32A	TC15001594.hg.1	0,000582751	0,010863718
MRPS14	TC01003543.hg.1	0,011072261	0,058833921
ZHX3	TC20000848.hg.1	0,000582751	0,010863718
TUBGCP4	TC15000328.hg.1	0,006993007	0,044547453
NOP56	TC20001062.hg.1	0,001165501	0,015073094
DAPK1	TC09000398.hg.1	0,000582751	0,010863718
RBM43	TC02002424.hg.1	0,000582751	0,010863718
ANAPC1	TC02000530.hg.1	0,001165501	0,015073094
MMAA	TC04002245.hg.1	0,002331002	0,022134595
PNPO	TC17000617.hg.1	0,002331002	0,022134595
MPHOSPH6	TC16001302.hg.1	0,002331002	0,022134595
NUDT16	TC03000710.hg.1	0,000582751	0,010863718
PRKD2	TC19001653.hg.1	0,000582751	0,010863718
TMEM14A	TC06000658.hg.1	0,011072261	0,058833921
VWA5A	TC11001132.hg.1	0,000582751	0,010863718
CHD9	TC16001564.hg.1	0,011072261	0,058833921
THBD	TC20000702.hg.1	0,002331002	0,022134595
PTPN13	TC04002094.hg.1	0,002331002	0,022134595
GCNT1	TC09000350.hg.1	0,000582751	0,010863718
PUS7	TC07001734.hg.1	0,000582751	0,010863718
EXOSC5	TC19001555.hg.1	0,011072261	0,058833921
NET1	TC10000047.hg.1	0,017482517	0,079419358
TWISTNB	TC07001177.hg.1	0,001165501	0,015073094
ZNF215	TC11000142.hg.1	0,000582751	0,010863718
ATP8B1	TC18000538.hg.1	0,017482517	0,079419358
CCND1	TC11000718.hg.1	0,000582751	0,010863718
TIPIN	TC15001576.hg.1	0,017482517	0,079419358
FUT10	TC08002582.hg.1	0,000582751	0,010863718
MTAP	TC09002880.hg.1	0,004079254	0,031319232
ITSN1	TC21000681.hg.1	0,000582751	0,010863718
ATF7IP	TC12002262.hg.1	0,000582751	0,010863718
FAXDC2	TC05001973.hg.1	0,00214071	0,022134595
ABHD2	TC15002352.hg.1	0,004079254	0,031319232
ST6GAL1	TC03001028.hg.1	0,002331002	0,022134595
MBLAC2	TC05001585.hg.1	0,000582751	0,010863718
ORC2	TC02004778.hg.1	0,000582751	0,010863718
LRRC8D	TC01000841.hg.1	0,000582751	0,010863718

SH3D19	TC04002781.hg.1	0,000582751	0,010863718
TEX2	TC17001797.hg.1	0,000582751	0,010863718
PRUNE2	TC09001224.hg.1	0,002331002	0,022134595
HMGA2	TC12000587.hg.1	0,001165501	0,015073094
POLR3B	TC12000823.hg.1	0,001165501	0,015073094
LSM10	TC01002507.hg.1	0,000582751	0,010863718
MBD5	TC02000925.hg.1	0,004079254	0,031319232
S1PR1	TC01000909.hg.1	0,000582751	0,010863718
SESN3	TC11002205.hg.1	0,006993007	0,044547453
TNRC6B	TC22001471.hg.1	0,000582751	0,010863718
WDR3	TC01001033.hg.1	0,006993007	0,044547453
NR1D1	TC17002615.hg.1	0,011072261	0,058833921
ARHGAP12	TC10001159.hg.1	0,001165501	0,015073094
ABHD2	TC15000844.hg.1	0,004079254	0,031319232
MPZL3	TC11002345.hg.1	0,004079254	0,031319232
METTL23	TC17002377.hg.1	0,000582751	0,010863718
KIF13B	TC08001097.hg.1	0,000582751	0,010863718
ATM	TC11000980.hg.1	0,001165501	0,015073094
SETBP1	TC18000160.hg.1	0,002331002	0,022134595
PGPEP1	TC19000355.hg.1	0,000582751	0,010863718
KLF9	TC09001201.hg.1	0,011072261	0,058833921
LPIN2	TC18000803.hg.1	0,000582751	0,010863718
SCD5	TC04001338.hg.1	0,000582751	0,010863718
IMP3	TC15001669.hg.1	0,001165501	0,015073094
PIP4K2B	TC17001433.hg.1	0,000582751	0,010863718
ALDH3A2	TC17000246.hg.1	0,006993007	0,044547453
KBTBD6	TC13000599.hg.1	0,001165501	0,015073094
ZNF148	TC03003060.hg.1	0,017482517	0,079419358
CSGALNACT1	TC08001022.hg.1	0,002331002	0,022134595
SLX4IP	TC20000083.hg.1	0,000582751	0,010863718
WSCD1	TC17000069.hg.1	0,002331002	0,022134595
LPIN2	TC18000285.hg.1	0,001165501	0,015073094
MKL2	TC16000171.hg.1	0,000582751	0,010863718
MTR	TC01005133.hg.1	0,000582751	0,010863718
AMD1	TC06000883.hg.1	0,004079254	0,031319232
NMRK1	TC09002604.hg.1	0,006993007	0,044547453
HELZ	TC17002754.hg.1	0,006993007	0,044547453
ATR	TC03001854.hg.1	0,000582751	0,010863718
FIG4	TC06000879.hg.1	0,000582751	0,010863718
MDN1	TC06001944.hg.1	0,002068779	0,022134595
PTCD2	TC05000338.hg.1	0,000582751	0,010863718
SLC1A1	TC09000029.hg.1	0,011072261	0,058833921
RRS1	TC08002603.hg.1	0,000582751	0,010863718
PIEZO2	TC18000324.hg.1	0,017482517	0,079419358
PPAT	TC04001220.hg.1	0,002331002	0,022134595
SEMA6B	TC19001066.hg.1	0,001165501	0,015073094
MEIS2	TC15001211.hg.1	0,004079254	0,031319232
POLR1B	TC02000707.hg.1	0,00214071	0,022134595
ATM	TC11002857.hg.1	0,000582751	0,010863718
RALGAPA2	TC20001464.hg.1	0,006993007	0,044547453

USP18	TC22000029.hg.1	0,000582751	0,010863718
FLRT2	TC14000514.hg.1	0,002331002	0,022134595
PGAP1	TC02002644.hg.1	0,006993007	0,044547453
ABHD10	TC03000566.hg.1	0,012618408	0,06685837
FASN	TC17001973.hg.1	0,002331002	0,022134595
INTS9	TC08002267.hg.1	0,002331002	0,022134595
MAPK14	TC06000523.hg.1	0,000582751	0,010863718
RALGAPA2	TC20000685.hg.1	0,006993007	0,044547453
NR5A2	TC01004937.hg.1	0,011072261	0,058833921
ITSN1	TC21000683.hg.1	0,002331002	0,022134595
GGA2	TC16000963.hg.1	0,000582751	0,010863718
C21orf59	TC21000924.hg.1	0,000582751	0,010863718
GLCE	TC15000633.hg.1	0,000582751	0,010863718
CSTF2T	TC10001297.hg.1	0,001165501	0,015073094
NR2F2	TC15000936.hg.1	0,000582751	0,010863718
MRPL17	TC11001364.hg.1	0,002331002	0,022134595
RIN2	TC20000134.hg.1	0,000582751	0,010863718
FAM63B	TC15002216.hg.1	0,001165501	0,015073094
GPR4	TC19002713.hg.1	0,001165501	0,015073094
STEAP1	TC07000536.hg.1	0,006993007	0,044547453
KIAA1919	TC06000890.hg.1	0,000582751	0,010863718
RIN2	TC20001131.hg.1	0,000582751	0,010863718
SSH2	TC17001322.hg.1	0,000582751	0,010863718
RPL31	TC02004436.hg.1	0,004079254	0,031319232
CAMK2D	TC04001486.hg.1	0,004079254	0,031319232
FAM63B	TC15000445.hg.1	0,000582751	0,010863718
TOB1	TC17001698.hg.1	0,000582751	0,010863718
RORA	TC15002563.hg.1	0,002331002	0,022134595
ADM	TC11000182.hg.1	0,000582751	0,010863718
EIF2AK4	TC15000272.hg.1	0,000582751	0,010863718
FRMD3	TC09001278.hg.1	0,001165501	0,015073094
MGAT4A	TC02002127.hg.1	0,000582751	0,010863718
RAB11A	TC15002244.hg.1	0,000582751	0,010863718
RANBP6	TC09000896.hg.1	0,002331002	0,022134595
DHCR24	TC01002694.hg.1	0,011072261	0,058833921
ATIC	TC02003807.hg.1	0,000582751	0,010863718
NAMPT	TC07001738.hg.1	0,000582751	0,010863718
CITED2	TC06002168.hg.1	0,000582751	0,010863718
HMG20A	TC15000712.hg.1	0,000582751	0,010863718
OCLN	TC05000304.hg.1	0,002331002	0,022134595
SESN3	TC11003298.hg.1	0,002331002	0,022134595
BCL2L1	TC20000756.hg.1	0,000582751	0,010863718
SUOX	TC12000498.hg.1	0,000582751	0,010863718
LGR4	TC11001509.hg.1	0,001165501	0,015073094
TSHZ1	TC18000252.hg.1	0,000582751	0,010863718
ARHGAP18	TC06004137.hg.1	0,000582751	0,010863718
EDNRB	TC13001585.hg.1	0,004079254	0,031319232
KNOP1	TC16000920.hg.1	0,000582751	0,010863718
SLC20A1	TC02000711.hg.1	0,000582751	0,010863718
MGARP	TC04002948.hg.1	0,017482517	0,079419358

MTMR4	TC17001734.hg.1	0,000582751	0,010863718
ARHGAP29	TC01005638.hg.1	0,004079254	0,031319232
FLI1	TC11001185.hg.1	0,000582751	0,010863718
EDNRB	TC13000748.hg.1	0,006993007	0,044547453
SH3D19	TC04001637.hg.1	0,000582751	0,010863718
AHNAK2	TC14001562.hg.1	0,000582751	0,010863718
CARD8	TC19001677.hg.1	0,002331002	0,022134595
SLC25A30	TC13000634.hg.1	0,000582751	0,010863718
RAPGEF5	TC07001185.hg.1	0,000582751	0,010863718
EXOC6	TC10000661.hg.1	0,000582751	0,010863718
NFIB	TC09000916.hg.1	0,000582751	0,010863718
TGFBRAP1	TC02002166.hg.1	0,000582751	0,010863718
CARD8	TC19002534.hg.1	0,006993007	0,044547453
AHNAK2	TC14002183.hg.1	0,000582751	0,010863718
S1PR1	TC01005668.hg.1	0,006993007	0,044547453
CYYR1	TC21000344.hg.1	0,004079254	0,031319232
PREX2	TC08000452.hg.1	0,000582751	0,010863718
NUDT16	TC03002549.hg.1	0,000582751	0,010863718
MKL2	TC16001421.hg.1	0,001165501	0,015073094
PDE3A	TC12002281.hg.1	0,017482517	0,079419358
STAT1	TC02002624.hg.1	0,001165501	0,015073094
CGNL1	TC15000437.hg.1	0,004079254	0,031319232
PARP4	TC13000491.hg.1	0,000582751	0,010863718
PDE3A	TC12000227.hg.1	0,006993007	0,044547453
PLK2	TC05001385.hg.1	0,000582751	0,010863718
DUSP6	TC12002493.hg.1	0,001165501	0,015073094
NFIA	TC01000702.hg.1	0,000582751	0,010863718
ACACA	TC17002601.hg.1	0,000582751	0,010863718
STYK1	TC12001226.hg.1	0,000582751	0,010863718
PRICKLE1	TC12001405.hg.1	0,000582751	0,010863718
ZFP36L2	TC02001790.hg.1	0,000582751	0,010863718
ACACA	TC17001406.hg.1	0,000582751	0,010863718
MLKL	TC16001270.hg.1	0,001165501	0,015073094
ETV1	TC07001152.hg.1	0,000582751	0,010863718
SETBP1	TC18000692.hg.1	0,000582751	0,010863718
PRKCH	TC14000371.hg.1	0,000582751	0,010863718
TRAM2	TC06001800.hg.1	0,000582751	0,010863718
OCLN	TC05001458.hg.1	0,006993007	0,044547453
DUSP4	TC08001099.hg.1	0,000582751	0,010863718
CSGALNACT1	TC08002245.hg.1	0,001165501	0,015073094
HMGA2	TC12002424.hg.1	0,000582751	0,010863718
BCL2L1	TC20001531.hg.1	0,000582751	0,010863718
NR5A2	TC01001645.hg.1	0,001165501	0,015073094
TRIM25	TC17001716.hg.1	0,000582751	0,010863718
PLSCR4	TC03001867.hg.1	0,001165501	0,015073094
DUSP6	TC12001796.hg.1	0,000582751	0,010863718
CDC42EP3	TC02001746.hg.1	0,000582751	0,010863718
ZMYND8	TC20000908.hg.1	0,000582751	0,010863718
MTUS1	TC08001013.hg.1	0,000582751	0,010863718
PRICKLE1	TC12002841.hg.1	0,000582751	0,010863718

PIK3CG	TC07000687.hg.1	0,000582751	0,010863718
GIMAP7	TC07002674.hg.1	0,000582751	0,010863718
GIMAP7	TC07001003.hg.1	0,000582751	0,010863718
BMP4	TC14001148.hg.1	0,000582751	0,010863718
SDPR	TC02002627.hg.1	0,000582751	0,010863718
DUSP4	TC08002271.hg.1	0,000582751	0,010863718

Supplementary Table 10a. Hypomethylated DMRs BAV-D (fc 10%), and associated genes				
**Region**	**DMR start**	**DMR end**	**Chr**	**Gene**
R.9	1294140	1295077	1	MXRA8 (-694)
R.10	1296671	1297576	1	MXRA8 (-3209)
R.13	1369793	1369948	1	VWA1 (-1180)
R.16	1397524	1398028	1	ATAD3B (-9373), ATAD3C (+12707)
R.18	1566351	1566687	1	MMP23B (-955)
R.19	1713944	1714140	1	NADK (-4133)
R.21	1897728	1897959	1	GABRD (-52936), TMEM52 (-47132)
R.23	2106222	2106400	1	C1orf86 (+19902), PRKCZ (+124402)
R.24	2138953	2139905	1	SKI (-20705), C1orf86 (-13216)
R.25	2144873	2145276	1	C1orf86 (-18862), SKI (-15059)
R.28	2254199	2254582	1	RER1 (-68881), SKI (+94257)
R.29	2262232	2262474	1	RER1 (-60919), SKI (+102219)
R.30	2284501	2285178	1	RER1 (-38432), SKI (+124706)
R.31	2349734	2350197	1	PLCH2 (-48932), PEX10 (-5997)
R.32	2375010	2375627	1	PEX10 (-31350), PLCH2 (-23579)
R.34	2435087	2435827	1	PANK4 (+22582), PLCH2 (+36559)
R.35	2436214	2437200	1	PANK4 (+21332), PLCH2 (+37809)
R.40	2792637	2792744	1	MMEL1 (-228262), ACTRT2 (-145355)
R.43	2839179	2839247	1	MMEL1 (-274784), ACTRT2 (-98833)
R.45	2844168	2844871	1	MMEL1 (-280091), ACTRT2 (-93526)
R.47	2896587	2896834	1	MMEL1 (-332282), ACTRT2 (-41335)
R.50	2983926	2984245	1	PRDM16 (-1689)
R.51	2999586	2999941	1	ARHGEF16 (-371226), PRDM16 (+13989)
R.52	3010896	3011304	1	ARHGEF16 (-359890), PRDM16 (+25325)
R.53	3017674	3018024	1	ARHGEF16 (-353141), PRDM16 (+32074)
R.55	3036168	3037102	1	ARHGEF16 (-334355), PRDM16 (+50860)
R.57	3044741	3044804	1	ARHGEF16 (-326217), PRDM16 (+58998)
R.58	3058521	3059137	1	ARHGEF16 (-312161), PRDM16 (+73054)
R.61	3077798	3078524	1	ARHGEF16 (-292829), PRDM16 (+92386)
R.63	3103048	3104042	1	ARHGEF16 (-267445), PRDM16 (+117770)
R.64	3104999	3105252	1	ARHGEF16 (-265864), PRDM16 (+119351)
R.65	3120245	3121019	1	ARHGEF16 (-250358), PRDM16 (+134857)
R.66	3133763	3134420	1	ARHGEF16 (-236898), PRDM16 (+148317)
R.67	3135083	3135836	1	ARHGEF16 (-235530), PRDM16 (+149685)
R.68	3141992	3142925	1	ARHGEF16 (-228531), PRDM16 (+156684)
R.69	3143018	3143682	1	ARHGEF16 (-227640), PRDM16 (+157575)
R.70	3154700	3155418	1	ARHGEF16 (-215931), PRDM16 (+169284)
R.71	3157287	3158200	1	ARHGEF16 (-213246), PRDM16 (+171969)
R.74	3191660	3192542	1	ARHGEF16 (-178889), PRDM16 (+206326)
R.75	3193118	3193944	1	ARHGEF16 (-177459), PRDM16 (+207756)
R.76	3199650	3200586	1	ARHGEF16 (-170872), PRDM16 (+214343)
R.77	3228966	3229195	1	ARHGEF16 (-141909), PRDM16 (+243306)
R.78	3230250	3230424	1	ARHGEF16 (-140653), PRDM16 (+244562)
R.79	3265739	3266440	1	ARHGEF16 (-104900), PRDM16 (+280315)
R.80	3269252	3269478	1	ARHGEF16 (-101625), PRDM16 (+283590)
R.81	3272131	3272732	1	ARHGEF16 (-98558), PRDM16 (+286657)
R.82	3282757	3283488	1	ARHGEF16 (-87867), PRDM16 (+297348)
R.86	3339870	3340625	1	ARHGEF16 (-30742), PRDM16 (+354473)

R.88	3459949	3460268	1	MEGF6 (+67950), ARHGEF16 (+89119)
R.89	3466683	3467219	1	MEGF6 (+61108), ARHGEF16 (+95961)
R.90	3473665	3474202	1	MEGF6 (+54125), ARHGEF16 (+102944)
R.93	3600735	3600879	1	SMIM1 (-88545), TP73 (+31723)
R.95	3623859	3624292	1	SMIM1 (-65276), TP73 (+54992)
R.96	3649250	3649771	1	SMIM1 (-39841), TP73 (+80427)
R.98	4000125	4000600	1	AJAP1 (-714742), C1orf174 (-183514)
R.99	6187633	6188120	1	CHD5 (+52306), KCNAB2 (+81896)
R.102	6514605	6514781	1	TNFRSF25 (+11542), ESPN (+29845)
R.106	6558085	6558497	1	PLEKHG5 (-1135)
R.107	6579837	6580525	1	PLEKHG5 (-23025), NOL9 (+34414)
R.108	6615264	6615604	1	NOL9 (-839), TAS1R1 (+193)
R.113	7844450	7844895	1	PER3 (-90)
R.115	8403631	8404216	1	SLC45A1 (+26038), RERE (+473600)
R.116	8878173	8878225	1	RERE (-675)
R.117	9129646	9129791	1	SLC2A5 (+56)
R.120	9409560	9410333	1	SLC25A33 (-189594), SPSB1 (+57008)
R.128	10695686	10696066	1	CASZ1 (+160829), PEX14 (+160932)
R.129	11248185	11249057	1	ANGPTL7 (-777)
R.130	11322728	11322809	1	MTOR (-205)
R.131	11714218	11714254	1	FBXO44 (-678), FBXO2 (+503)
R.132	11760856	11761296	1	AGTRAP (-35136), DRAXIN (+9290)
R.133	11795905	11795976	1	AGTRAP (-271)
R.137	12538341	12538678	1	DHRS3 (+139227), TNFRSF1B (+311450)
R.138	12600225	12600744	1	DHRS3 (+77252), TNFRSF1B (+373425)
R.139	12703864	12704007	1	AADACL4 (-630)
R.142	15271830	15272326	1	TMEM51 (-206950), KAZN (+346878)
R.143	15541182	15541349	1	EFHD2 (-195125), TMEM51 (+62238)
R.145	16062361	16063122	1	SLC25A34 (-158)
R.146	16090718	16091599	1	FBLIM1 (+165)
R.149	16268457	16268969	1	ZBTB17 (+33908), SPEN (+94354)
R.150	16345293	16346120	1	CLCNKA (-2840), HSPB7 (-422)
R.151	16347995	16348752	1	HSPB7 (-3089), CLCNKA (-173)
R.152	16369406	16370265	1	CLCNKB (-436)
R.153	17025723	17026406	1	CROCC (-222380), NBPF1 (-86083)
R.154	17091946	17092279	1	CROCC (-156332), NBPF1 (-152131)
R.156	17307215	17308032	1	MFAP2 (-294)
R.159	17865653	17865737	1	ARHGEF10L (-635)
R.161	17944356	17944660	1	ACTL8 (-137300), ARHGEF10L (+78178)
R.166	19639308	19639494	1	AKR7A2 (-761), PQLC2 (+579)
R.167	19745731	19746564	1	CAPZB (+65831), PQLC2 (+107326)
R.168	19764480	19764821	1	CAPZB (+47328), PQLC2 (+125829)
R.169	19992167	19992771	1	HTR6 (+689)
R.171	20396560	20396750	1	PLA2G5 (-46)
R.172	21586831	21587174	1	EIF4G3 (-83660), ECE1 (+29979)
R.173	22778527	22778738	1	ZBTB40 (+289)
R.181	23697506	23698143	1	ZNF436 (-1890)
R.182	23879671	23880481	1	E2F2 (-22364), ID3 (+6209)
R.185	24229232	24229575	1	FUCA1 (-34620), CNR2 (+56145)
R.186	24438607	24438781	1	MYOM3 (-29)

R.187	24469564	24469791	1	IL22RA1 (-67)
R.190	25240865	25240938	1	RUNX3 (+50599), CLIC4 (+169054)
R.191	25257505	25257566	1	RUNX3 (+33965), CLIC4 (+185688)
R.192	25257599	25258332	1	RUNX3 (+33535), CLIC4 (+186118)
R.196	26233538	26233709	1	STMN1 (-667)
R.197	26346812	26347541	1	EXTL1 (-1094)
R.199	26394009	26394593	1	TRIM63 (-177)
R.202	27240365	27240669	1	NR0B2 (-60)
R.204	27675934	27676652	1	SYTL1 (+4929), MAP3K6 (+17090)
R.206	27693782	27693840	1	MAP3K6 (-428)
R.207	27709771	27709791	1	CD164L2 (+12)
R.208	27718221	27718277	1	GPR3 (-899)
R.209	27729053	27729992	1	GPR3 (+10375), WASF2 (+87146)
R.210	27849102	27849295	1	WASF2 (-32530), AHDC1 (+80944)
R.211	27901698	27902555	1	WASF2 (-85458), AHDC1 (+28016)
R.213	32041507	32041898	1	TINAGL1 (-433)
R.214	32042157	32042921	1	TINAGL1 (+403)
R.215	32052246	32053005	1	HCRTR1 (-30675), TINAGL1 (+10490)
R.216	32054510	32055006	1	HCRTR1 (-28543), TINAGL1 (+12622)
R.217	32573942	32574126	1	KPNA6 (+395)
R.218	32665450	32665584	1	CCDC28B (-470)
R.221	32828016	32828191	1	TSSK3 (+306)
R.225	33813321	33814006	1	A3GALT2 (-26965), PHC2 (+27530)
R.226	34632573	34632588	1	CSMD2 (-1138)
R.227	36916605	36916744	1	OSCP1 (-623)
R.230	38022586	38022718	1	SNIP1 (-2749), DNALI1 (+132)
R.231	38200920	38201123	1	EPHA10 (+29716), CDCA8 (+42932)
R.232	38461540	38461896	1	SF3A3 (-5125), FHL3 (+9459)
R.233	38513318	38513641	1	POU3F1 (-1030)
R.234	39407387	39407851	1	AKIRIN1 (-49276), RHBDL2 (-15110)
R.235	39546511	39546656	1	MACF1 (-2450)
R.236	39873077	39874025	1	BMP8A (-83767), MACF1 (+324517)
R.238	40149381	40149650	1	NT5C1A (-11806), HPCAL4 (+7845)
R.239	40598455	40598779	1	PPT1 (-35242), RLF (-28428)
R.240	40780093	40780905	1	COL9A2 (+2467), ZMPSTE24 (+56720)
R.241	42384310	42385160	1	HIVEP3 (-566)
R.243	43814306	43815035	1	CDC20 (-10013), MPL (+11193)
R.244	44445539	44446092	1	B4GALT2 (+157)
R.245	44497352	44497595	1	KLF17 (-87048), SLC6A9 (-14477)
R.247	44884109	44884150	1	RNF220 (+13264), TMEM53 (+256097)
R.248	45082704	45083278	1	TMEM53 (+57236), RNF220 (+212125)
R.249	45272255	45272637	1	TCTEX1D4 (+511)
R.250	45274513	45275340	1	TCTEX1D4 (-1970)
R.252	46088336	46089045	1	NASP (+38959), GPBP1L1 (+63611)
R.253	46639632	46640255	1	TSPAN1 (-815)
R.255	47779661	47779775	1	STIL (+94)
R.257	47900256	47900320	1	FOXD2 (-1401)
R.260	48452465	48452740	1	TRABD2B (+9964), FOXD2 (+550914)
R.262	50886920	50886969	1	DMRTA2 (+2227), ELAVL4 (+314981)
R.264	51887613	51888574	1	TTC39A (-77306), EPS15 (+96906)

R.269	54619655	54619895	1	CDCP2 (-332)
R.270	55246867	55247408	1	PARS2 (-16951), DHCR24 (+105753)
R.271	59042931	59043070	1	TACSTD2 (+165)
R.272	60539362	60539671	1	C1orf87 (-75)
R.273	61545546	61546247	1	NFIA (-1637)
R.275	67217673	67217886	1	INSL5 (+49159), SGIP1 (+217815)
R.276	71172023	71172486	1	CTH (+295300), PTGER3 (+341216)
R.277	74663297	74663636	1	TNNI3K (-480), FPGT (-459)
R.279	84767878	84768702	1	DNASE2B (-95925), PRKACB (+158336)
R.280	85464110	85464261	1	MCOLN2 (-1563)
R.281	85665444	85665902	1	MCOLN3 (-151544), SYDE2 (+1056)
R.282	85725654	85725931	1	SYDE2 (-59064), BCL10 (+16980)
R.283	86047433	86047911	1	CYR61 (+1228), ZNHIT6 (+126429)
R.284	86048479	86048923	1	CYR61 (+2257), ZNHIT6 (+125400)
R.285	87793364	87793510	1	LMO4 (-714)
R.286	88928647	88928839	1	PKN2 (-221162)
R.287	92946132	92947035	1	GLMN (-182051), GFI1 (+2927)
R.288	95698378	95699097	1	RWDD3 (-973)
R.289	99729460	99729769	1	ENSG00000117600 (+106)
R.290	100111387	100111949	1	PALMD (+169)
R.291	103574469	103574619	1	COL11A1 (-497)
R.292	109941060	109941201	1	SORT1 (-558)
R.293	110035670	110036417	1	CYB561D1 (-692)
R.295	110166563	110166664	1	GSTM4 (-32089), AMPD2 (+3338)
R.296	110452372	110452616	1	CSF1 (-761)
R.299	110933248	110933767	1	SLC16A4 (+196)
R.300	111218079	111218287	1	KCNA3 (-528)
R.303	113261711	113262512	1	PPM1J (-4013), FAM19A3 (-929)
R.306	116021925	116022091	1	VANGL1 (-162566), NGF (-141151)
R.308	117213980	117214228	1	IGSF3 (-3790)
R.309	117451543	117452320	1	PTGFRN (-747)
R.312	119528638	119529219	1	SPAG17 (-801083), TBX15 (+3250)
R.313	119531857	119532093	1	TBX15 (+204)
R.314	119542159	119542295	1	TBX15 (-10048), WARS2 (+141067)
R.315	119548527	119549145	1	TBX15 (-16657), WARS2 (+134458)
R.316	120838644	120839380	1	NOTCH2 (-226772), FCGR1B (+96925)
R.317	120905306	120906047	1	NOTCH2 (-293437), FCGR1B (+30260)
R.318	144533354	144534187	1	NBPF9 (-277977), PPIAL4B (-169525)
R.319	145092330	145092576	1	ENSG00000255168 (-116692), PDE4DIP (-97431)
R.320	145412774	145413193	1	HFE2 (-294)
R.321	145470946	145471727	1	POLR3GL (-950), ANKRD34A (+829)
R.322	147012684	147012802	1	BCL9 (-439)
R.324	147781827	147782558	1	NBPF24 (-182644), PPIAL4A (+173226)
R.325	147789747	147789973	1	NBPF24 (-190311), PPIAL4A (+165559)
R.326	149230829	149231118	1	PPIAL4C (-322029), NBPF16 (+491531)
R.327	149287547	149288143	1	PPIAL4C (-265158), NBPF16 (+548402)
R.328	149399174	149399911	1	PPIAL4C (-153460), NBPF16 (+660100)
R.329	149672849	149673689	1	FCGR1A (-80977), PPIAL4C (+120266)
R.330	149890522	149890655	1	SV2A (-1155)
R.332	149908085	149908313	1	MTMR11 (+592)

R.333	149908606	149909548	1	MTMR11 (-286)
R.335	150255137	150255241	1	APH1A (-13790), MRPS21 (-11151)
R.337	150522266	150523231	1	ADAMTSL4 (+851)
R.339	150980214	150980411	1	PRUNE (-640), FAM63A (+538)
R.342	151137274	151137676	1	SCNM1 (-1043), LYSMD1 (+920)
R.343	151344806	151345248	1	SELENBP1 (+182)
R.344	151967021	151967697	1	S100A10 (-493)
R.346	153174654	153175262	1	LOR (-57218), SPRR2G (-51613)
R.349	153413641	153413940	1	S100A7L2 (-1366)
R.350	153514008	153514482	1	S100A5 (-4)
R.351	153520801	153521758	1	S100A4 (-2919), S100A3 (+568)
R.353	153746211	153746899	1	SLC27A3 (-1213)
R.354	153762201	153762244	1	SLC27A3 (+14455), GATAD2B (+133228)
R.358	154839813	154839983	1	ADAR (-259216), KCNN3 (+2858)
R.359	154929734	154929791	1	PBXIP1 (-1183)
R.360	154942509	154943349	1	CKS1B (-4246), SHC1 (+294)
R.363	155177237	155178215	1	MTX1 (-764), THBS3 (-38)
R.364	155290813	155291345	1	RUSC1 (+361)
R.366	155910958	155911238	1	RXFP4 (-382)
R.370	156217178	156218113	1	PAQR6 (+197)
R.371	156261200	156261841	1	C1orf85 (+3942), TMEM79 (+7451)
R.372	156338775	156338826	1	RHBG (-202)
R.374	156631235	156631248	1	NES (+15947), BCAN (+19502)
R.382	159869960	159870086	1	CCDC19 (-70)
R.383	160050948	160051377	1	KCNJ9 (-197)
R.385	160084263	160085132	1	ATP1A2 (-865)
R.386	160085433	160085581	1	ATP1A2 (-56)
R.388	160159672	160160312	1	CASQ1 (-354)
R.391	160765349	160765919	1	LY9 (-314)
R.393	161088188	161089118	1	PFDN2 (-752), NIT1 (+762)
R.394	161167745	161168451	1	NDUFS2 (-1007), ADAMTS4 (+748)
R.398	162601334	162602295	1	DDR2 (-440)
R.399	162792092	162792246	1	RGS4 (-246396), HSD17B7 (+31633)
R.400	163172649	163173040	1	RGS5 (-152)
R.402	164544748	164545699	1	PBX1 (+16352), LMX1A (+780728)
R.403	164545783	164546143	1	PBX1 (+17091), LMX1A (+779989)
R.405	165513318	165513361	1	LRRC52 (+96)
R.406	166890380	166890924	1	TADA1 (-45088), ILDR2 (+53909)
R.407	167063581	167064091	1	GPA33 (-3968), DUSP27 (+554)
R.408	167408509	167408841	1	CD247 (+79100), POU2F1 (+218609)
R.411	167689891	167690788	1	MPZL1 (-847)
R.412	169555944	169556657	1	F5 (-475)
R.413	170253405	170254397	1	GORAB (-247369), METTL11B (+138759)
R.414	170501141	170501645	1	GORAB (+123)
R.415	171621279	171621941	1	MYOC (+201)
R.416	172113506	172114419	1	PIGC (+299263), DNM3 (+303342)
R.418	173572181	173572694	1	SLC9C2 (-205)
R.419	174967663	174968089	1	CACYBP (-993)
R.420	175013154	175013920	1	TNN (-23457), MRPS14 (-20976)
R.421	175474437	175474533	1	TNR (+238421), TNN (+437491)

R.423	177140609	177140812	1	FAM5B (+78)
R.425	178310534	178310650	1	TEX35 (-171620), RASAL2 (+247316)
R.426	178455912	178456270	1	TEX35 (-26121), RASAL2 (+392815)
R.427	179199424	179200312	1	ABL2 (-1132)
R.429	184943911	184944153	1	FAM129A (-350)
R.431	193155273	193155806	1	B3GALT2 (+244)
R.432	199717229	199717464	1	NR5A2 (-279383)
R.434	200842282	200842756	1	GPR25 (+436)
R.438	201708500	201708718	1	IPO9 (-89660), NAV1 (+91232)
R.439	201777257	201777623	1	IPO9 (-20829), NAV1 (+160063)
R.440	201856763	201857621	1	SHISA4 (-644)
R.441	201978949	201979128	1	GPR37L1 (-112947), ELF3 (+1966)
R.442	202090924	202091880	1	GPR37L1 (-584)
R.443	202091933	202092005	1	GPR37L1 (-17)
R.446	202181869	202182642	1	LGR6 (+19227), UBE2T (+128852)
R.449	203444000	203444945	1	PRELP (-483)
R.450	204121899	204121925	1	ETNK2 (-781)
R.452	205497713	205498551	1	MFSD4 (-39973), CDK18 (+24406)
R.453	206786170	206786180	1	EIF2D (-271)
R.454	207070825	207071109	1	IL24 (+179)
R.459	207991122	207991597	1	CD46 (+65958), CD34 (+93387)
R.460	207995752	207996459	1	CD46 (+70704), CD34 (+88641)
R.461	209798721	209799353	1	LAMB3 (+25642), CAMK1G (+41975)
R.463	210407677	210407973	1	HHAT (-94425), SYT14 (+296287)
R.466	212687842	212688447	1	ATF3 (-93867), NENF (+81916)
R.473	214477063	214477280	1	SMYD2 (+22596), PTPN14 (+247394)
R.474	215178658	215179151	1	KCNK2 (-77674), CENPF (+402367)
R.479	221057558	221058198	1	HLX (+6179), DUSP10 (+857640)
R.480	221068834	221069136	1	HLX (+17286), DUSP10 (+846533)
R.481	223888869	223889141	1	CAPN8 (-35657), CAPN2 (-11029)
R.482	224363449	224363575	1	TP53BP2 (-329838), DEGS1 (-7361)
R.483	226128626	226129561	1	LEFTY2 (+95)
R.484	226296868	226297335	1	H3F3A (+45424), ACBD3 (+77329)
R.485	226924257	226924846	1	ITPKB (+2312), C1orf95 (+188051)
R.488	227962723	227963296	1	PRSS38 (-40384), SNAP47 (+40313)
R.489	228337311	228337379	1	GJC2 (-208)
R.492	228870454	228870637	1	RHOU (-278)
R.495	230272542	230272656	1	GALNT2 (+69643), PGBD5 (+240792)
R.496	231298651	231299396	1	TRIM67 (+308)
R.497	231761688	231761845	1	DISC1 (-794)
R.498	234667087	234667549	1	TARBP1 (-52469), IRF2BP2 (+77953)
R.499	234852797	234852892	1	IRF2BP2 (-107574), TOMM20 (+439406)
R.500	235256497	235257141	1	IRF2BP2 (-511548), TOMM20 (+35432)
R.501	236304341	236304503	1	GPR137B (-1410)
R.506	241912510	241912768	1	OPN3 (-108976), EXO1 (-98843)
R.507	242686709	242687412	1	PLD5 (+759)
R.508	243646402	243647204	1	SDCCAG8 (+227445), AKT3 (+366627)
R.509	244213358	244214206	1	ZBTB18 (-803)
R.511	247611842	247611931	1	OR2B11 (+3421), NLRP3 (+30536)
R.513	247802802	247803033	1	OR13G1 (+33447), OR2G3 (+34062)

R.514	47319	47716	2	FAM110C (-1133)
R.515	193256	193509	2	FAM110C (-146998), SH3YL1 (+70682)
R.523	660692	660856	2	FAM150B (-372478), TMEM18 (+16665)
R.524	691862	692026	2	SNTG2 (-254610), TMEM18 (-14505)
R.525	1286216	1286397	2	TPO (-130926), SNTG2 (+339753)
R.526	1287787	1287978	2	TPO (-129350), SNTG2 (+341329)
R.528	1565328	1565960	2	TPO (+148411), PXDN (+182634)
R.529	1595734	1595933	2	PXDN (+152444), TPO (+178601)
R.531	1647185	1647260	2	PXDN (+101055), TPO (+229990)
R.532	1656557	1657483	2	PXDN (+91258), TPO (+239787)
R.534	1711759	1712527	2	PXDN (+36135), TPO (+294910)
R.536	1820169	1821101	2	PXDN (-72357), MYT1L (+514253)
R.537	1821733	1822412	2	PXDN (-73795), MYT1L (+512815)
R.539	3173204	3173292	2	MYT1L (-838360), TRAPPC12 (-210209)
R.543	3641709	3642634	2	COLEC11 (-7324), RPS7 (+19377)
R.544	3642710	3642867	2	COLEC11 (-6707), RPS7 (+19994)
R.549	6072283	6072981	2	SOX11 (+239833), CMPK2 (+933195)
R.552	8833307	8833764	2	ID2 (+14561), KIDINS220 (+144219)
R.553	9458925	9459386	2	ITGB1BP1 (+104202), ASAP2 (+112262)
R.556	10686963	10687583	2	ODC1 (-98643), NOL10 (+142820)
R.558	11118387	11118564	2	PQLC3 (-177022), KCNF1 (+66413)
R.559	11809858	11810183	2	NTSR2 (+269)
R.560	11924984	11925224	2	TRIB2 (-931911), LPIN1 (+107383)
R.561	11990297	11990493	2	TRIB2 (-866620), LPIN1 (+172674)
R.562	14773457	14774086	2	NBAS (+927682)
R.563	16124662	16124940	2	MYCN (+44115), FAM49A (+722301)
R.565	19550754	19551750	2	NT5C1B-RDH14 (-780424), OSR1 (+5425)
R.566	19551789	19551944	2	NT5C1B-RDH14 (-781039), OSR1 (+4810)
R.567	19553237	19554237	2	NT5C1B-RDH14 (-782909), OSR1 (+2940)
R.568	21266500	21266995	2	APOB (+197)
R.569	24398427	24398859	2	PFN4 (-52296), ITSN2 (+184940)
R.570	25475726	25475916	2	POMC (-84049), DNMT3A (+89638)
R.571	25552259	25552751	2	POMC (-160733), DNMT3A (+12954)
R.572	26523139	26523591	2	GPR113 (+18552), HADHB (+55602)
R.573	26700908	26700976	2	DRC1 (+76158), OTOF (+80624)
R.575	26785891	26786046	2	OTOF (-4403)
R.576	26915207	26915355	2	KCNK3 (-338)
R.577	26916149	26916575	2	KCNK3 (+743)
R.578	27300240	27301195	2	EMILIN1 (-717)
R.579	27301252	27301943	2	EMILIN1 (+163)
R.580	27371818	27371891	2	TCF23 (-17)
R.581	27530829	27531535	2	UCN (+131)
R.582	27603886	27604131	2	ZNF513 (-417)
R.583	27665017	27665711	2	KRTCAP3 (+131)
R.584	27718710	27719544	2	FNDC4 (-1015), GCKR (-582)
R.586	28618328	28619094	2	PLB1 (-100227), FOSL2 (+2986)
R.587	29204117	29204695	2	WDR43 (+86897), C2orf71 (+92721)
R.589	31805915	31806042	2	XDH (-168398), MEMO1 (+429647)
R.590	33359198	33359529	2	RASGRP3 (-341931), LTBP1 (+187325)
R.591	36582038	36582398	2	CRIM1 (-851)

R.593	38357299	38358147	2	CYP1B1 (-54400), ATL2 (+246681)
R.595	39892124	39892612	2	TMEM178A (-691)
R.596	40658342	40658918	2	THUMPD2 (-652223), SLC8A1 (+80871)
R.597	42276988	42277840	2	EML4 (-119076), PKDCC (+2254)
R.598	42794680	42795486	2	MTA3 (-574)
R.599	43327937	43328536	2	HAAO (-308505), ZFP36L2 (+125511)
R.610	46612964	46613544	2	TMEM247 (-93471), EPAS1 (+88713)
R.612	54086854	54087517	2	GPR75 (-60), GPR75-ASB3 (-16), ENSG00000270898 (+111)
R.613	54858240	54858444	2	EML6 (-93337), SPTBN1 (+174920)
R.617	61406491	61407414	2	AHSA2 (+2289), USP34 (+290951)
R.622	64834106	64834430	2	SERTAD2 (+46779), AFTPH (+82803)
R.623	65085243	65085764	2	SERTAD2 (-204457), SLC1A4 (-131031)
R.624	66298197	66298242	2	SPRED2 (-638909), MEIS1 (-364312)
R.626	68545960	68546038	2	PPP3R1 (-66336), PLEK (-46306)
R.630	69665094	69665232	2	NFU1 (-403)
R.633	71131201	71131748	2	ATP6V1B1 (-31537), VAX2 (+3755)
R.634	71787663	71787827	2	DYSF (+93913), CYP26B1 (+587422)
R.635	72078638	72079609	2	CYP26B1 (+296043), DYSF (+385292)
R.636	73495839	73496203	2	EGR4 (+24808), CCT7 (+34655)
R.638	74119781	74120274	2	ACTG2 (-107)
R.640	74601431	74602278	2	ENSG00000264324 (-11351), DCTN1 (+5627)
R.641	74607470	74608183	2	DCTN1 (-345)
R.642	74668072	74668976	2	RTKN (+502)
R.643	74669349	74669516	2	RTKN (-407)
R.644	74742786	74743243	2	TLX2 (+1404), DQX1 (+10448)
R.645	75136185	75136552	2	POLE4 (-49250), HK2 (+75261)
R.649	85822152	85822407	2	RNF181 (-577)
R.650	85999498	85999873	2	ATOH8 (+18669), ST3GAL5 (+116451)
R.651	86038423	86038589	2	ATOH8 (+57489), ST3GAL5 (+77631)
R.654	88583529	88583805	2	TEX37 (-240502), THNSL2 (+112688)
R.655	97166001	97166422	2	ARID5A (-36274), NCAPH (+164687)
R.656	97215100	97215848	2	ARID5A (+12988), KANSL3 (+88572)
R.657	97524767	97525009	2	ANKRD39 (-1056)
R.662	99438903	99439883	2	MGAT4A (-91804), TSGA10 (+331987)
R.663	99953016	99953048	2	EIF5B (-784), TXNDC9 (-107)
R.664	100720179	100720529	2	REV1 (-613857), AFF3 (+1691)
R.665	100722022	100722584	2	AFF3 (-258)
R.666	100723110	100723417	2	AFF3 (-1219)
R.670	102313069	102313242	2	MAP4K4 (-1387)
R.673	103236553	103237268	2	SLC9A2 (+745)
R.675	105697589	105697777	2	GPR45 (-160517), MRPS9 (+43242)
R.676	106054920	106055458	2	NCK2 (-306165), FHL2 (-39508)
R.678	106681831	106681983	2	C2orf40 (-205)
R.680	108993602	108994528	2	SULT1C4 (-403)
R.681	109203501	109204230	2	LIMS1 (-67627), GCC2 (+138849)
R.682	109622481	109622566	2	SH3RF3 (-123473), EDAR (-16696)
R.683	109746735	109747264	2	SH3RF3 (+1003), SEPT10 (+624783)
R.684	109788188	109788778	2	SH3RF3 (+42486), SEPT10 (+583300)
R.685	109934728	109934860	2	SH3RF3 (+188797), SEPT10 (+436989)

R.689	113956531	113957041	2	PSD4 (+25238), PAX8 (+79741)
R.693	121371842	121372064	2	GLI2 (-178032), INHBB (+268234)
R.694	121625208	121625735	2	GLI2 (+75487), TFCP2L1 (+417311)
R.695	121669723	121670320	2	GLI2 (+120037), TFCP2L1 (+372761)
R.696	127951364	127951465	2	BIN1 (-86484), CYP27C1 (+11928)
R.698	128410029	128410803	2	GPR17 (+6977), LIMS2 (+11704)
R.699	128422224	128422717	2	LIMS2 (-351)
R.700	128438738	128439345	2	SFT2D3 (-19555), LIMS2 (-16922)
R.701	128453108	128453335	2	LIMS2 (-31102), SFT2D3 (-5375)
R.703	130634806	130635421	2	RAB6C (-102121)
R.704	131089926	131090049	2	TUBA3E (-133954), IMP4 (-9810)
R.707	132152814	132152960	2	TUBA3D (-80779), PLEKHB2 (+290467)
R.708	134326017	134326172	2	NCKAP5 (-64)
R.709	135808369	135808617	2	RAB3GAP1 (-1375)
R.710	139258740	139259034	2	SPOPL (-484)
R.711	142889367	142889392	2	LRP1B (-110)
R.714	149401448	149401942	2	EPC2 (-858)
R.716	152830189	152830521	2	ARL5A (-145353), CACNB4 (+125238)
R.717	154728210	154729059	2	GALNT13 (+209)
R.718	157292018	157292127	2	GPD2 (+120)
R.719	158113471	158114268	2	GALNT5 (-240)
R.721	161126857	161127564	2	ITGB6 (-70399), RBMS1 (+223094)
R.722	161127827	161128184	2	ITGB6 (-71194), RBMS1 (+222299)
R.723	164204628	164204915	2	KCNH7 (-509644), FIGN (+387745)
R.724	165811982	165812159	2	SLC38A11 (-75)
R.726	169658981	169659121	2	NOSTRIN (+4390), SPC25 (+87904)
R.733	172950171	172950397	2	DLX1 (+330)
R.734	172952136	172952883	2	DLX1 (+2556), DLX2 (+15118)
R.735	172953032	172953270	2	DLX1 (+3197), DLX2 (+14477)
R.736	172965046	172965280	2	DLX2 (+2465), DLX1 (+15209)
R.739	173686002	173686677	2	ENSG00000091436 (-254347), RAPGEF4 (+85771)
R.740	173792545	173792879	2	ENSG00000091436 (-147975), RAPGEF4 (+192143)
R.741	173940203	173940277	2	ENSG00000091436 (-447)
R.743	176867156	176867187	2	HOXD10 (-114135), HOXD11 (-104842), HOXD12 (-97286), HOXD13 (-90447), KIAA1715 (-99), EVX2 (+81469)
R.745	176972041	176972113	2	HOXD10 (-9230), HOXD11 (+63)
R.746	176981064	176981336	2	HOXD10 (-107)
R.749	181843183	181843895	2	UBE2E3 (-1794)
R.750	190446181	190446792	2	SLC40A1 (-874)
R.751	196520930	196521354	2	SLC39A10 (-329)
R.752	196933852	196934154	2	DNAH7 (-467)
R.753	198540518	198540621	2	RFTN2 (+149)
R.756	202484020	202484098	2	TMEM237 (+24181), STRADB (+167667)
R.757	203036093	203036235	2	SUMO1 (+67167), FZD7 (+136854)
R.758	204103863	204104259	2	CYP20A1 (+398)
R.761	207507163	207507528	2	DYTN (+75774), ADAM23 (+199083)
R.762	215675429	215675637	2	BARD1 (-1105)
R.763	216299448	216299856	2	FN1 (+1243), ATIC (+123112)
R.765	218621486	218621497	2	TNP1 (-896705), TNS1 (+187359)

R.766	218842585	218843504	2	RUFY4 (-90933), TNS1 (-34194)
R.773	219267198	219268015	2	VIL1 (-16208), CTDSP1 (+3129)
R.776	220253653	220253668	2	DNPEP (-924)
R.777	220282165	220282281	2	DES (-876)
R.778	220308747	220309687	2	GMPPA (-54406), SPEG (+9649)
R.779	220324649	220325375	2	GMPPA (-38611), SPEG (+25444)
R.781	220436894	220436936	2	OBSL1 (-904), INHA (-2)
R.783	223916502	223916952	2	KCNE4 (-135)
R.784	223917577	223918000	2	KCNE4 (+927)
R.786	228735722	228736145	2	DAW1 (-389)
R.788	231688289	231688923	2	ITM2C (-41009), CAB39 (+111046)
R.791	233251770	233252706	2	ALPPL2 (-19315), ALPP (+8994)
R.792	233284402	233284934	2	ALPI (-36165), ALPPL2 (+13115)
R.793	233390712	233390859	2	CHRND (-108)
R.796	234296642	234297039	2	DGKD (+33688), USP40 (+177395)
R.798	234369962	234370070	2	USP40 (+104220), DGKD (+106863)
R.799	235883549	235883680	2	SH3BP4 (-3714)
R.801	235934945	235935754	2	AGAP1 (-467401), SH3BP4 (+48021)
R.803	237078733	237079122	2	GBX2 (-1916)
R.804	237081650	237082073	2	GBX2 (-4850)
R.805	237172796	237173121	2	ASB18 (+29)
R.806	237993279	237993660	2	COPS8 (-485)
R.807	238322274	238322972	2	COL6A3 (+395)
R.808	238394933	238395125	2	MLPH (-782)
R.812	238647755	238647913	2	RBM44 (-59554), LRRFIP1 (+46852)
R.815	239046879	239047774	2	FAM132B (-20322), SCLY (+77797)
R.816	239169537	239169696	2	HES6 (-20852), PER2 (+27633)
R.817	239195537	239196219	2	HES6 (-47113), PER2 (+1372)
R.820	240061601	240062353	2	HDAC4 (+260666), TWIST2 (+305304)
R.824	240212763	240213173	2	HDAC4 (+109675), TWIST2 (+456295)
R.830	241458886	241459847	2	ANKMY1 (+38013), GPC1 (+84279)
R.831	241533694	241534607	2	GPR35 (-10711), CAPN10 (+8018)
R.832	241535685	241536284	2	GPR35 (-8877), CAPN10 (+9852)
R.834	241866245	241866549	2	SNED1 (-71858), ENSG00000226321 (+4411)
R.835	241936844	241937034	2	SNED1 (-1316)
R.836	241974917	241975756	2	SNED1 (+37082), MTERFD2 (+66410)
R.838	242127836	242127948	2	ANO7 (-32)
R.841	242448557	242448802	2	STK25 (-573)
R.842	242499464	242499542	2	BOK (+1367), THAP4 (+77361)
R.844	242598543	242598843	2	ATG4B (+21666), DTYMK (+27713)
R.845	242751796	242752475	2	NEU4 (+41)
R.848	242810748	242811149	2	CXXC11 (-932)
R.852	1134005	1134730	3	CNTN6 (+108)
R.853	2141937	2142391	3	CNTN4 (-138349)
R.854	2553085	2553187	3	CNTN4 (+272623), IL5RA (+598922)
R.859	9791044	9791144	3	OGG1 (-534)
R.864	11178593	11178798	3	HRH1 (-115689), SLC6A1 (+144286)
R.865	11610138	11610613	3	VGLL4 (+75022), ATG7 (+296274)
R.866	12199836	12200835	3	TIMP4 (+515)
R.867	13060741	13060974	3	IQSEC1 (-51690), NUP210 (+400951)

R.868	13113959	13114441	3	IQSEC1 (-105032), NUP210 (+347609)
R.870	13664480	13665022	3	FBLN2 (+74120), WNT7A (+256867)
R.871	14339534	14339700	3	XPC (-119334), SLC6A6 (-104459)
R.872	14443428	14443694	3	SLC6A6 (-515)
R.873	14581937	14582410	3	C3orf20 (-134432), SLC6A6 (+138098)
R.875	16216094	16216606	3	GALNT15 (+194)
R.876	24537792	24538388	3	THRB (-1824)
R.877	25469914	25469925	3	RARB (+118)
R.879	32509030	32509212	3	CMTM6 (+35779), CMTM7 (+75958)
R.881	37904034	37904475	3	CTDSPL (+590)
R.886	42694144	42695035	3	ZBTB47 (-586)
R.887	42700203	42701042	3	KLHL40 (-26388), ZBTB47 (+5447)
R.888	42947263	42947385	3	ZNF662 (-334)
R.891	44596360	44596512	3	ZKSCAN7 (-264)
R.893	45067564	45067788	3	CLEC3B (+1)
R.901	47952098	47952841	3	DHX30 (+108052), MAP4 (+178292)
R.903	48456686	48456733	3	SPINK8 (-86879), PLXNB1 (+14162)
R.904	48471575	48471901	3	PLXNB1 (-866)
R.907	48884897	48885314	3	PRKAR2A (+173)
R.909	49459855	49460177	3	AMT (+170)
R.910	49757438	49757476	3	AMIGO3 (+3892), RNF123 (+30525)
R.911	49967462	49967713	3	MON1A (-10)
R.913	50303757	50304463	3	LSMEM2 (-12348), GNAI2 (+30726)
R.914	50306107	50307037	3	LSMEM2 (-9886), GNAI2 (+33188)
R.917	50315748	50316384	3	LSMEM2 (-392)
R.919	50397107	50397379	3	TMEM115 (-202)
R.920	50487955	50488230	3	TMEM115 (-91052), CACNA2D2 (+52761)
R.921	50654374	50654499	3	DOCK3 (-58235), MAPKAPK3 (+5098)
R.922	51740741	51741473	3	GRM2 (+21)
R.923	51989369	51989764	3	GPR62 (+237)
R.924	52351680	52351963	3	DNAH1 (+1487), BAP1 (+92544)
R.925	52488070	52488229	3	NISCH (-1374), TNNC1 (-64)
R.926	52552868	52553167	3	NT5DC2 (+14775), STAB1 (+23664)
R.931	52864659	52864816	3	ITIH4 (+17)
R.932	52869266	52869521	3	ITIH4 (-4639), MUSTN1 (-159)
R.936	55693182	55693631	3	WNT5A (-169434), ERC2 (+808984)
R.937	62358234	62358379	3	FEZF2 (+883)
R.939	64211328	64211994	3	PRICKLE2 (-530)
R.940	64547108	64547373	3	PRICKLE2 (-336110), ADAMTS9 (+126435)
R.941	66454856	66455738	3	LRIG1 (+96059), SLC25A26 (+336012)
R.942	69061575	69061827	3	FAM19A4 (-79940), EOGT (+1073)
R.943	69914738	69915449	3	MITF (+126508)
R.944	71293694	71294341	3	FOXP1 (-114030), EIF4E3 (+480508)
R.945	73672063	73672998	3	PDZRN3 (+1560), PPP4R2 (+626595)
R.946	73674074	73674146	3	PDZRN3 (-19)
R.947	73674166	73674363	3	PDZRN3 (-174)
R.949	75668627	75668924	3	FRG2C (-44705)
R.950	75704498	75705108	3	FRG2C (-8678)
R.951	79815718	79815848	3	ROBO1 (+1182)
R.952	81811322	81812214	3	GBE1 (-456)

R.953	100712058	100712345	3	ABI3BP (+157)
R.956	111577968	111577981	3	PHLDB2 (-52)
R.958	111804830	111805005	3	C3orf52 (-264)
R.959	112359488	112359652	3	CCDC80 (+546)
R.960	113160071	113160637	3	SPICE1 (+73649), BOC (+230025)
R.961	113417997	113418063	3	KIAA2018 (-2537)
R.962	114343068	114343779	3	GAP43 (-998933), ZBTB20 (-240936)
R.963	114790243	114790596	3	ZBTB20 (-687932), GAP43 (-551937)
R.964	119379220	119379792	3	POPDC2 (-69)
R.965	120170383	120170646	3	FSTL1 (-415)
R.966	121312127	121312241	3	FBXO40 (+218)
R.967	121612956	121613703	3	SLC15A2 (+394)
R.968	122514541	122514814	3	HSPBAP1 (-2007), DIRC2 (+1036)
R.969	122712138	122712271	3	PDIA5 (-73712), SEMA5B (-17224)
R.970	122785328	122785650	3	PDIA5 (-428)
R.971	123123576	123124018	3	ADCY5 (+44808), SEC22A (+196628)
R.972	123165872	123166774	3	ADCY5 (+2282), SEC22A (+239154)
R.973	123339334	123340306	3	PTPLB (-35788), MYLK (+263329)
R.974	123699208	123699389	3	KALRN (-114229), CCDC14 (-18735)
R.975	124103560	124103696	3	UMPS (-345585), KALRN (+290100)
R.976	124931956	124932002	3	SLC12A8 (-1736)
R.977	125820484	125821264	3	SLC41A3 (-17727), ALDH1L1 (+79155)
R.978	125900065	125900108	3	ALDH1L1 (-58)
R.979	126721131	126721586	3	PLXNA1 (+13922), TPRA1 (+588253)
R.987	128722515	128723007	3	GP9 (-56849), EFCC1 (+2289)
R.989	132756721	132756991	3	TMEM108 (-379)
R.990	135684107	135684163	3	PPP2R3A (-380)
R.997	139257713	139257907	3	RBP1 (+861)
R.998	139346734	139346818	3	RBP1 (-88105), NMNAT3 (+50083)
R.1000	140813769	140814133	3	ACPL2 (-136719), SPSB4 (+43707)
R.1001	141087187	141087363	3	ZBTB38 (-19379), ACPL2 (+136605)
R.1002	141161580	141161911	3	RASA2 (-44145), ZBTB38 (+55092)
R.1003	141495400	141496003	3	GRK7 (-1288)
R.1006	147125942	147125962	3	ZIC4 (-3881), ZIC1 (-1219)
R.1007	149095283	149095743	3	TM4SF1 (+139)
R.1008	149374066	149374914	3	WWTR1 (+46570), TM4SF4 (+182729)
R.1009	149687002	149687773	3	PFN2 (+1508), RNF13 (+156831)
R.1010	151177285	151177940	3	IGSF10 (-1116)
R.1011	151178727	151178904	3	IGSF10 (-2319)
R.1012	153840536	153840718	3	ARHGEF26 (+1835), DHX36 (+201659)
R.1013	155394109	155394292	3	PLCH1 (-373)
R.1014	155463133	155463719	3	PLCH1 (-69598), C3orf33 (+60629)
R.1016	158450550	158450833	3	RARRES1 (-207)
R.1017	158518888	158519410	3	MFSD1 (-505)
R.1018	169377725	169377946	3	MECOM (-513743), ACTRT3 (+109847)
R.1019	169483122	169483295	3	MECOM (-619116), ACTRT3 (+4474)
R.1020	169683976	169684239	3	SEC62 (-445)
R.1021	170136159	170136327	3	CLDN11 (-410)
R.1022	171561060	171561201	3	TMEM212 (-8)
R.1023	178275476	178276460	3	KCNMB2 (-520)

R.1026	184037459	184038317	3	FAM131A (-17352), EIF4G1 (+4692)
R.1027	184055123	184055379	3	FAM131A (+11)
R.1028	184056553	184056843	3	FAM131A (+1458), CLCN2 (+22741)
R.1030	187387555	187387866	3	SST (+476)
R.1032	187870929	187871034	3	BCL6 (-407467), LPP (-59739)
R.1033	187896277	187897193	3	BCL6 (-433220), LPP (-33986)
R.1034	187929286	187929592	3	LPP (-1282)
R.1035	187930521	187930704	3	LPP (-108)
R.1036	188664887	188664993	3	TPRG1 (-224823), LPP (+734219)
R.1037	188665466	188666022	3	TPRG1 (-224019), LPP (+735023)
R.1039	191048308	191048439	3	UTS2B (-49)
R.1040	192444985	192445202	3	HRASLS (-513820), FGF12 (-318256)
R.1041	192445533	192445582	3	HRASLS (-513356), FGF12 (-318720)
R.1043	193310532	193310749	3	OPA1 (-292)
R.1045	193987426	193987651	3	CPN2 (+84518), HES1 (+133605)
R.1046	194014481	194014530	3	CPN2 (+57551), HES1 (+160572)
R.1051	195530091	195530532	3	MUC4 (+8836), MUC20 (+82559)
R.1052	195589996	195590056	3	MUC4 (-50878), TNK2 (+32406)
R.1057	196705629	196705898	3	PIGZ (-10060), MFI2 (+50923)
R.1059	106750	107725	4	ZNF595 (+54028), ZNF732 (+191872)
R.1060	206282	206442	4	ZNF732 (+92748), ZNF595 (+153152)
R.1061	298926	299121	4	ZNF732 (+86)
R.1062	379900	380396	4	ZNF141 (+48524), ZNF721 (+112797)
R.1064	646577	647322	4	ATP5I (+21177), PDE6B (+27555)
R.1069	961505	962124	4	DGKQ (+5529), TMEM175 (+35640)
R.1070	1027885	1028067	4	FGFRL1 (+24209), RNF212 (+79338)
R.1071	1029410	1029654	4	FGFRL1 (+25765), RNF212 (+77782)
R.1072	1043540	1043631	4	FGFRL1 (+39819), RNF212 (+63728)
R.1073	1148456	1148569	4	RNF212 (-41199), SPON2 (+18148)
R.1076	1201881	1202599	4	SPON2 (-35579), CTBP1 (+40685)
R.1078	1204956	1205914	4	SPON2 (-38774), CTBP1 (+37490)
R.1079	1206004	1206150	4	SPON2 (-39416), CTBP1 (+36848)
R.1080	1303710	1304048	4	UVSSA (-37225), MAEA (+20240)
R.1082	1398383	1398798	4	NKX1-1 (+1528), CRIPAK (+13251)
R.1083	1595294	1595515	4	NKX1-1 (-195286), FAM53A (+90583)
R.1086	2627014	2627194	4	FAM193A (-55)
R.1090	3043199	3043752	4	HTT (-32932), GRK4 (+78021)
R.1094	3485263	3486039	4	DOK7 (+20613), LRPAP1 (+48635)
R.1095	3773815	3773996	4	ADRA2C (+5781), OTOP1 (+454710)
R.1096	4228637	4228650	4	OTOP1 (-28)
R.1097	4341397	4341544	4	ENSG00000168824 (-8396), ZBTB49 (+49493)
R.1108	5710411	5711301	4	EVC (-2068), EVC2 (-562)
R.1109	5990265	5990638	4	CRMP1 (-95667), JAKMIP1 (+211830)
R.1110	6223973	6224223	4	WFS1 (-47479), JAKMIP1 (-21816)
R.1111	6247571	6247830	4	JAKMIP1 (-45419), WFS1 (-23876)
R.1114	6955660	6955953	4	TADA2B (-89311), TBC1D14 (+44235)
R.1116	7046066	7046407	4	TADA2B (+1119), GRPEL1 (+23687)
R.1117	7252323	7252587	4	SORCS2 (+58190), PSAPL1 (+184245)
R.1119	7657359	7657708	4	PSAPL1 (-220834), AFAP1 (+284119)
R.1120	7762705	7763703	4	PSAPL1 (-326504), AFAP1 (+178449)

R.1121	7768357	7768660	4	PSAPL1 (-331809), AFAP1 (+173144)
R.1126	8345155	8346138	4	HTRA3 (+74155), ACOX3 (+96791)
R.1127	8395941	8396502	4	ACOX3 (+46216), HTRA3 (+124730)
R.1129	8582227	8582287	4	GPR78 (-36)
R.1132	9783192	9783398	4	DRD5 (+37)
R.1134	11369007	11369730	4	CLNK (-682880), HS3ST1 (+62020)
R.1135	11652441	11652505	4	HS3ST1 (-221084)
R.1137	15376116	15376135	4	CC2D2A (-95363), C1QTNF7 (+34566)
R.1138	15428750	15429572	4	CC2D2A (-42328), C1QTNF7 (+87601)
R.1139	15683206	15683224	4	FAM200B (-214)
R.1140	23892515	23892621	4	PPARGC1A (-868)
R.1141	24795830	24796753	4	SOD3 (-793)
R.1142	24796919	24797176	4	SOD3 (-37)
R.1143	25090597	25090665	4	LGI2 (-58130), SEPSECS (+71433)
R.1147	26491963	26492206	4	CCKAR (-1)
R.1148	30723983	30724915	4	PCDH7 (+1404)
R.1151	39448432	39449053	4	RPL9 (+11765), KLB (+40270)
R.1152	40752838	40753247	4	NSUN7 (+1129), APBB2 (+463506)
R.1153	40858965	40859095	4	NSUN7 (+107116), APBB2 (+357519)
R.1156	41614397	41614958	4	PHOX2B (+136309), LIMCH1 (+251874)
R.1159	53523482	53524296	4	USP46 (+1613), SPATA18 (+606392)
R.1160	53727714	53727979	4	RASL11B (-610)
R.1161	53728923	53729461	4	RASL11B (+735)
R.1169	66534326	66535145	4	EPHA5 (+1325)
R.1170	74718399	74719172	4	PF4V1 (-120)
R.1171	74864239	74864596	4	CXCL5 (+78)
R.1172	75895596	75895858	4	THAP6 (-543956), PARM1 (+37401)
R.1174	77356175	77356416	4	SHROOM3 (+43)
R.1175	79471927	79472643	4	ANXA3 (-388)
R.1176	79971177	79971408	4	PAQR3 (-110701), NAA11 (+275911)
R.1178	80885348	80886264	4	GK2 (-556434), ANTXR2 (+107911)
R.1179	81106663	81106857	4	PRDM8 (+336)
R.1180	81109888	81110459	4	FGF5 (-77579), PRDM8 (+3750)
R.1181	81111177	81111985	4	FGF5 (-76172), PRDM8 (+5157)
R.1182	81122314	81123306	4	FGF5 (-64943), PRDM8 (+16386)
R.1183	81128234	81128690	4	FGF5 (-59291), PRDM8 (+22038)
R.1184	83674602	83675306	4	TMEM150C (-191587), SCD5 (+45056)
R.1185	86748944	86749040	4	ARHGAP24 (+352725), MAPK10 (+532224)
R.1186	87770089	87770782	4	SLC10A6 (-20)
R.1187	88450506	88451496	4	SPARCL1 (-346)
R.1189	89978251	89978566	4	FAM13A (-86)
R.1193	100576610	100576985	4	DAPP1 (-161205), MTTP (+91558)
R.1195	103682690	103683140	4	MANBA (-764)
R.1196	111396631	111397155	4	ENPEP (-336)
R.1200	114213914	114214449	4	ANK2 (+243350), CAMK2D (+468042)
R.1201	114900050	114900431	4	ARSJ (+616)
R.1202	119948360	119948505	4	MYOZ2 (-108506), SYNPO2 (+138437)
R.1203	120056829	120057818	4	MYOZ2 (+385)
R.1205	123747447	123747672	4	FGF2 (-303)
R.1208	145566200	145566595	4	HHIP (-775)

R.1210	148401828	148401958	4	EDNRA (-191)
R.1211	149362435	149362830	4	NR3C2 (+1039), ARHGAP10 (+709419)
R.1218	157996959	157997086	4	GLRB (-186)
R.1219	159094213	159095085	4	FAM198B (-447)
R.1220	160188727	160189254	4	RAPGEF2 (+102)
R.1221	165304531	165304855	4	MARCH1 (+509)
R.1222	166130172	166131163	4	KLHL2 (-377)
R.1223	166299786	166299976	4	CPE (-213)
R.1224	166301076	166301256	4	TLL1 (-493244), CPE (+1072)
R.1228	174442087	174442812	4	SCRG1 (-121763), HAND2 (+8930)
R.1229	174444166	174445082	4	SCRG1 (-123937), HAND2 (+6756)
R.1230	174445349	174446201	4	SCRG1 (-125088), HAND2 (+5605)
R.1231	174452985	174453287	4	HAND2 (-1756)
R.1234	183247132	183247234	4	TENM3 (+82601), DCTD (+591349)
R.1235	184908739	184909018	4	STOX2 (+82370), ENPP6 (+230235)
R.1237	186658766	186659155	4	PDLIM3 (-202299), SORBS2 (+218845)
R.1238	186732936	186733428	4	PDLIM3 (-276520), SORBS2 (+144624)
R.1239	186741362	186742139	4	PDLIM3 (-285089), SORBS2 (+136055)
R.1240	186960904	186961192	4	SORBS2 (-83242), TLR3 (-29258)
R.1241	187148430	187148688	4	KLKB1 (+3)
R.1242	187590495	187590579	4	MTNR1A (-113816), FAT1 (+54472)
R.1243	187621221	187621327	4	MTNR1A (-144553), FAT1 (+23735)
R.1244	188916709	188916814	4	ZFP42 (-163)
R.1246	190731443	190731709	4	FRG1 (-130367)
R.1248	191127	191492	5	SDHA (-27046), PLEKHG4B (+50937)
R.1250	345850	346247	5	AHRR (+41758), C5orf55 (+97209)
R.1254	493448	493746	5	SLC9A3 (+30850), EXOC3 (+50324)
R.1255	669397	669837	5	TPPP (+23893), CEP72 (+57230)
R.1256	969899	969939	5	BRD9 (-76980), NKD2 (-39025)
R.1258	1016279	1016768	5	NKD2 (+7580), SLC12A7 (+95626)
R.1260	1033518	1033714	5	NKD2 (+24672), SLC12A7 (+78534)
R.1262	1201359	1201639	5	SLC6A19 (-211)
R.1263	1446165	1446443	5	SLC6A3 (-759)
R.1266	1552259	1553224	5	LPCAT1 (-28650), MRPL36 (+247267)
R.1274	1948875	1949480	5	IRX4 (-61846), IRX2 (+802598)
R.1276	2007611	2008270	5	IRX4 (-120609), IRX2 (+743835)
R.1277	2066634	2066711	5	IRX4 (-179341), IRX2 (+685103)
R.1278	2128976	2129941	5	IRX4 (-242127), IRX2 (+622317)
R.1280	2387826	2388038	5	IRX4 (-500600), IRX2 (+363844)
R.1282	3854704	3855077	5	IRX1 (+258723)
R.1284	5140003	5140406	5	ADAMTS16 (-238)
R.1285	5652065	5652129	5	ADAMTS16 (+511654), MED10 (+726610)
R.1287	6753739	6754116	5	ADCY2 (-642393), PAPD7 (+39210)
R.1288	6755439	6755843	5	ADCY2 (-640680), PAPD7 (+40923)
R.1289	9547468	9548098	5	SEMA5A (-1596)
R.1290	14406021	14406732	5	FAM105B (-258480), TRIO (+262566)
R.1291	14410252	14410363	5	FAM105B (-254549), TRIO (+266497)
R.1292	14440492	14441261	5	FAM105B (-223980), TRIO (+297066)
R.1293	16179210	16179660	5	MARCH11 (+449)
R.1294	16508920	16509123	5	ZNF622 (-43121), FAM134B (+108145)

R.1295	27038707	27038836	5	CDH9 (-79)
R.1296	31638951	31639699	5	PDZD2 (-192)
R.1299	34042976	34043509	5	C1QTNF3 (+694)
R.1300	35230728	35231272	5	PRLR (-309)
R.1301	36302644	36303314	5	RANBP3L (-977)
R.1302	37249567	37250092	5	C5orf42 (-300)
R.1304	37838290	37838741	5	GDNF (-2587)
R.1305	38445563	38446192	5	LIFR (+149628), EGFLAM (+187367)
R.1309	41870875	41871058	5	OXCT1 (-346)
R.1310	42924215	42924552	5	ZNF131 (-197316), SEPP1 (-112203)
R.1311	42943857	42944457	5	ZNF131 (-177543), SEPP1 (-131976)
R.1313	43000890	43001210	5	SEPP1 (-188869), ZNF131 (-120650)
R.1315	43034043	43034529	5	SEPP1 (-222105), ZNF131 (-87414)
R.1318	45695643	45696206	5	HCN1 (+328)
R.1325	53814625	53815303	5	SNX18 (+1371), ESM1 (+466527)
R.1326	54281198	54281362	5	ESM1 (+211)
R.1328	59783035	59783906	5	PDE4D (-593846), DEPDC1B (+212517)
R.1329	63461216	63461305	5	RNF180 (-448)
R.1332	71014900	71015162	5	CARTPT (+41)
R.1333	71475111	71475356	5	MAP1B (+72173), MRPS27 (+140854)
R.1341	75013592	75014259	5	POC5 (-620)
R.1342	75919188	75919850	5	F2RL2 (-260)
R.1346	77945219	77945739	5	LHFPL2 (-831)
R.1347	78985425	78986160	5	CMYA5 (+93)
R.1348	80528609	80529340	5	CKMT2 (-168)
R.1351	83016630	83017553	5	HAPLN1 (+340)
R.1352	92909968	92910534	5	NR2F1 (-8792)
R.1353	92930530	92931173	5	NR2F1 (+11809), POU5F2 (+146491)
R.1355	95295740	95296150	5	GLRX (-137527), ELL2 (+1830)
R.1357	102200701	102201421	5	PAM (-653)
R.1358	111077837	111078579	5	STARD4 (-230000), NREP (+234420)
R.1359	112042851	112042966	5	APC (-309)
R.1360	112824700	112824765	5	MCC (-206)
R.1361	115152785	115152938	5	CDO1 (-211)
R.1362	115697214	115697696	5	SEMA6A (+213175), COMMD10 (+276767)
R.1363	121646553	121647308	5	SNCAIP (-833)
R.1365	122620929	122621186	5	CEP120 (+137939), PRDM6 (+196242)
R.1367	126626348	126626362	5	MEGF10 (-196)
R.1368	127871927	127872329	5	FBN2 (+122750), SLC12A2 (+452670)
R.1372	131592959	131593106	5	PDLIM4 (-331)
R.1373	131593413	131594335	5	PDLIM4 (+510)
R.1374	132113601	132113725	5	SEPT8 (-596)
R.1376	132158560	132159003	5	SEPT8 (-45715), SHROOM1 (+7808)
R.1377	132209089	132209201	5	LEAP2 (+1131), AFF4 (+90181)
R.1385	134735613	134735654	5	H2AFY (-322)
R.1393	135364970	135365012	5	TGFBI (+407)
R.1395	137071788	137072504	5	KLHL3 (-367)
R.1396	137802252	137803197	5	EGR1 (+1546), ETF1 (+76264)
R.1399	139040055	139040849	5	PSD2 (-134954), CXXC5 (+12575)
R.1400	139056667	139057496	5	PSD2 (-118324), CXXC5 (+29205)

R.1405	140480597	140480872	5	PCDHB3 (+501)
R.1406	140554081	140554819	5	PCDHB8 (-2921)
R.1407	140595072	140595722	5	PCDHB14 (-7681), PCDHB13 (+1888)
R.1408	140683367	140683632	5	SLC25A2 (+112)
R.1409	140743927	140744449	5	PCDHGA5 (+290)
R.1410	140772408	140772681	5	PCDHGA8 (+164)
R.1411	140789247	140789745	5	PCDHGA10 (-3247)
R.1412	140798971	140799305	5	PCDHGA11 (-1624)
R.1413	140807225	140808068	5	PCDHGA12 (-2538)
R.1414	140811312	140811642	5	PCDHGC3 (-44103), PCDHGA12 (+1292)
R.1416	140864020	140864474	5	PCDHGC5 (-4561), PCDHGC4 (-494)
R.1418	141339748	141339777	5	PCDH12 (-1136)
R.1419	142065539	142066245	5	ARHGAP26 (-84057), FGF1 (-64985)
R.1420	142077529	142077896	5	FGF1 (-76806), ARHGAP26 (-72236)
R.1422	145713754	145714097	5	POU4F3 (-4661)
R.1423	146888445	146888890	5	DPYSL3 (+951)
R.1425	148758791	148759090	5	IL17B (-102)
R.1426	148785250	148785846	5	IL17B (-26709), CSNK1A1 (+144979)
R.1427	149867577	149868359	5	RPS14 (-38658), NDST1 (-19706)
R.1428	149887008	149887787	5	NDST1 (-276)
R.1429	149980486	149980682	5	SYNPO (-58)
R.1430	149997153	149997694	5	MYOZ3 (-43020), SYNPO (+16782)
R.1431	149999033	149999249	5	MYOZ3 (-41303), SYNPO (+18499)
R.1432	150004773	150004918	5	MYOZ3 (-35598), SYNPO (+24204)
R.1433	150018914	150019603	5	MYOZ3 (-21185), SYNPO (+38617)
R.1434	150020212	150020338	5	MYOZ3 (-20169), SYNPO (+39633)
R.1435	150028553	150029342	5	MYOZ3 (-11496), SYNPO (+48306)
R.1437	150284302	150284600	5	ZNF300 (+94)
R.1439	151066662	151067341	5	SPARC (-276)
R.1440	154071076	154071247	5	HAND1 (-213338), LARP1 (-21300)
R.1441	155753616	155754265	5	SGCD (+174)
R.1443	157003181	157003983	5	ADAM19 (-799)
R.1445	159797762	159797918	5	C1QTNF2 (-192)
R.1446	169128937	169129494	5	FOXI1 (-403685), DOCK2 (+64965)
R.1447	169407439	169407753	5	FOXI1 (-125305), DOCK2 (+343345)
R.1454	171094783	171095097	5	C5orf50 (-117936), FGF18 (+248280)
R.1455	171190008	171190154	5	C5orf50 (-22795), FGF18 (+343421)
R.1456	172175321	172175765	5	DUSP1 (+22655), NEURL1B (+107274)
R.1457	172260464	172260799	5	ERGIC1 (-646)
R.1458	172305889	172306112	5	RPL26L1 (-79895), ERGIC1 (+44723)
R.1459	172331791	172332685	5	RPL26L1 (-53658), ERGIC1 (+70960)
R.1460	172447063	172447822	5	CREBRF (-35912), ATP6V0E1 (+36683)
R.1465	172750923	172751331	5	NKX2-5 (-88767), STC2 (+5379)
R.1466	173316748	173317342	5	ENSG00000170091 (-155562), CPEB4 (+1762)
R.1468	175108269	175109114	5	CPLX2 (-114621), HRH2 (+23659)
R.1469	176559334	176559563	5	NSD1 (-1477)
R.1471	176793941	176794405	5	SLC34A1 (-17272), RGS14 (+9335)
R.1473	176827082	176827671	5	PFN3 (+260)
R.1474	176829112	176829557	5	PFN3 (-1698)
R.1475	176901453	176901844	5	DBN1 (-1450)

R.1476	176918190	176918920	5	DBN1 (-18356), PDLIM7 (+6029)
R.1477	176919234	176919739	5	DBN1 (-19288), PDLIM7 (+5097)
R.1478	176922906	176923805	5	DBN1 (-23157), PDLIM7 (+1228)
R.1481	178157956	178158086	5	ZNF354A (-318)
R.1482	178368253	178368620	5	ZNF454 (+245)
R.1483	178692691	178692806	5	ADAMTS2 (+79682), ZNF354C (+205333)
R.1484	178741004	178741374	5	ADAMTS2 (+31242), ZNF354C (+253773)
R.1487	180045822	180046528	5	SCGB3A1 (-27635), FLT4 (+30449)
R.1488	391664	391743	6	IRF4 (-35)
R.1489	475353	475429	6	IRF4 (+83652), HUS1B (+181572)
R.1491	901264	901310	6	FOXQ1 (-411388), EXOC2 (-208176)
R.1496	1601229	1601509	6	FOXC1 (-9312), FOXF2 (+211300)
R.1497	1603115	1604081	6	FOXC1 (-7083), FOXF2 (+213529)
R.1498	1604134	1604697	6	FOXC1 (-6265), FOXF2 (+214347)
R.1499	1615843	1616109	6	FOXC1 (+5295), GMDS (+629950)
R.1500	1616792	1617619	6	FOXC1 (+6525), GMDS (+628720)
R.1501	1618814	1619445	6	FOXC1 (+8449), GMDS (+626796)
R.1502	1620889	1621807	6	FOXC1 (+10667), GMDS (+624578)
R.1503	1635611	1635808	6	FOXC1 (+25029), GMDS (+610216)
R.1504	1766046	1766316	6	FOXC1 (+155500), GMDS (+479745)
R.1505	2382649	2382777	6	GMDS (-136787), MYLK4 (+368487)
R.1506	2515297	2515337	6	GMDS (-269391), MYLK4 (+235883)
R.1508	3076190	3077041	6	BPHL (-42310), RIPK1 (+7958)
R.1509	3258655	3258989	6	TUBB2B (-30853), SLC22A23 (+197971)
R.1512	3724407	3724690	6	SLC22A23 (-267756), PXDC1 (+27711)
R.1513	3737830	3737956	6	SLC22A23 (-281100), PXDC1 (+14367)
R.1515	5026164	5026435	6	PPP1R3G (-59420), RPP40 (-22019)
R.1517	6725157	6725372	6	RREB1 (-382776), LY86 (+136924)
R.1518	8436218	8436296	6	SLC35B3 (-541)
R.1519	10415653	10415687	6	TFAP2A (-200)
R.1520	10555808	10556523	6	GCNT6 (-77827), GCNT2 (+27577)
R.1521	10585683	10586167	6	GCNT6 (-48068), GCNT2 (+57336)
R.1522	11044877	11044894	6	ELOVL2 (-339)
R.1524	11279656	11279727	6	TMEM170B (-258819), NEDD9 (-46777)
R.1526	13014491	13015221	6	PHACTR1 (+296314), TBC1D7 (+312288)
R.1529	15504030	15504923	6	DTNBP1 (+158796), JARID2 (+257950)
R.1530	15505345	15505460	6	DTNBP1 (+157870), JARID2 (+258876)
R.1531	17282700	17283113	6	CAP2 (-110540), RBM24 (+1330)
R.1532	17393563	17393647	6	CAP2 (+158)
R.1533	17599948	17600327	6	FAM8A1 (-448)
R.1534	17985408	17986167	6	NUP153 (-279170), KIF13A (+2012)
R.1535	18387360	18387465	6	RNF144B (-168)
R.1536	21664652	21665155	6	SOX4 (+70932), PRL (+632826)
R.1539	25652381	25652407	6	SCGN (-70)
R.1540	26045788	26046341	6	HIST1H2BB (-2180), HIST1H3C (+426)
R.1541	26225258	26225539	6	HIST1H3E (+16)
R.1548	28554680	28554774	6	SCAND3 (+385)
R.1550	29520698	29521046	6	MAS1L (-65134), UBD (+6830)
R.1551	29521228	29521356	6	MAS1L (-65554), UBD (+6410)
R.1552	29521781	29521788	6	MAS1L (-66047), UBD (+5917)

R.1558	29595194	29595349	6	GABBR1 (+5690), OR2H2 (+39589)
R.1560	29601398	29601705	6	GABBR1 (-590)
R.1561	29623646	29623821	6	MOG (-1253)
R.1562	29638918	29639793	6	ZFP57 (+5575), MOG (+14369)
R.1566	30041727	30042204	6	RNF39 (+1698), PPP1R11 (+7101)
R.1567	30042260	30042295	6	RNF39 (+1386), PPP1R11 (+7413)
R.1569	30094960	30095258	6	TRIM31 (-14226), TRIM40 (-9678)
R.1570	30095269	30095295	6	TRIM31 (-14399), TRIM40 (-9505)
R.1571	30095495	30095546	6	TRIM31 (-14638), TRIM40 (-9266)
R.1573	30122544	30122593	6	TRIM10 (+6142), TRIM40 (+17782)
R.1578	30174190	30174845	6	TRIM26 (+6625), TRIM15 (+43525)
R.1579	30175092	30175327	6	TRIM26 (+5933), TRIM15 (+44217)
R.1586	30522404	30522674	6	PRR3 (-2124)
R.1587	30522855	30523215	6	PRR3 (-1628)
R.1589	30579440	30579650	6	PPP1R10 (+5476), ABCF1 (+40392)
R.1591	30614329	30614422	6	C6orf136 (-440)
R.1594	30655720	30655896	6	PPP1R18 (-136)
R.1595	30687707	30687946	6	MDC1 (-2161), TUBB (-151)
R.1597	30691895	30691984	6	TUBB (+3962), FLOT1 (+18570)
R.1598	30698734	30698784	6	TUBB (+10781), FLOT1 (+11751)
R.1603	30720080	30720263	6	DDR1 (-131689), IER3 (-7841)
R.1604	30796199	30796243	6	IER3 (-83890), DDR1 (-55640)
R.1605	30850868	30850913	6	DDR1 (-970)
R.1606	30851417	30851624	6	DDR1 (-340)
R.1607	30852823	30853073	6	GTF2H4 (-23013), DDR1 (+1087)
R.1610	30881464	30881579	6	VARS2 (-586)
R.1611	30883203	30884124	6	VARS2 (+1556), SFTA2 (+16288)
R.1614	31082534	31082835	6	C6orf15 (-2349)
R.1616	31090929	31091383	6	CDSN (-2933)
R.1617	31110639	31111270	6	PSORS1C2 (-3828)
R.1619	31275148	31275881	6	HLA-C (-35652), HLA-B (+49449)
R.1621	31496644	31496752	6	MCCD1 (+204)
R.1630	31590576	31590640	6	PRRC2A (+2106), BAG6 (+29869)
R.1636	31631706	31632171	6	C6orf47 (-3390), ENSG00000263020 (-1940), CSNK2B (-1902)
R.1640	31683109	31683352	6	LY6G6D (+98)
R.1643	31696161	31696729	6	DDAH2 (+1124), C6orf25 (+5284)
R.1644	31704705	31704774	6	MSH5 (-3058), MSH5-SAPCD1 (-3057), CLIC1 (+355)
R.1647	31740787	31740978	6	VWA7 (+4188), MSH5 (+33085)
R.1648	31767033	31767750	6	VARS (-3662)
R.1650	31838724	31838973	6	NEU1 (-8166), SLC44A4 (+7929)
R.1654	31856706	31856773	6	SLC44A4 (-9962), EHMT2 (+8721)
R.1658	31868965	31869120	6	EHMT2 (-3582), ZBTB12 (+726)
R.1659	31913250	31913323	6	CFB (-199)
R.1663	31938751	31938861	6	STK19 (-915)
R.1664	31939059	31939106	6	STK19 (-638), DOM3Z (+984)
R.1668	32016214	32016247	6	TNXB (-2326)
R.1669	32026147	32026891	6	TNXB (-12614), ATF6B (+69498)
R.1670	32038177	32039176	6	TNXB (-24772), ATF6B (+57340)
R.1671	32043739	32044496	6	TNXB (-30213), ATF6B (+51899)

R.1672	32048679	32049263	6	TNXB (-35066), ATF6B (+47046)
R.1673	32049516	32049699	6	TNXB (-35703), ATF6B (+46409)
R.1674	32050332	32050905	6	TNXB (-36714), ATF6B (+45398)
R.1677	32059166	32059605	6	TNXB (-45481), ATF6B (+36631)
R.1678	32060681	32061478	6	TNXB (-47175), ATF6B (+34937)
R.1680	32073590	32074397	6	TNXB (-60089), ATF6B (+22023)
R.1681	32076417	32076592	6	TNXB (-62600), ATF6B (+19512)
R.1682	32078398	32078624	6	TNXB (-64606), ATF6B (+17506)
R.1685	32086893	32087190	6	TNXB (-73137), ATF6B (+8975)
R.1686	32095128	32095229	6	ATF6B (+838)
R.1689	32118811	32119041	6	PPT2-EGFL8 (-3073), PPT2 (-2374), PRRT1 (+803)
R.1690	32120077	32120324	6	PPT2-EGFL8 (-1798), PPT2 (-1099), PRRT1 (-472)
R.1691	32120895	32121130	6	PRRT1 (-1284), PPT2-EGFL8 (-986), PPT2 (-287)
R.1692	32121156	32121259	6	PRRT1 (-1479), PPT2-EGFL8 (-791), PPT2 (-92)
R.1693	32121355	32121433	6	PRRT1 (-1665), PPT2-EGFL8 (-605), PPT2 (+94)
R.1696	32136431	32137210	6	EGFL8 (+4439), AGPAT1 (+8036)
R.1698	32150063	32150585	6	AGER (+1777), RNF5 (+4193)
R.1700	32156100	32156294	6	AGER (-4096)
R.1702	32164585	32164801	6	GPSM3 (-4010)
R.1703	32165089	32165237	6	GPSM3 (-4480)
R.1705	32729563	32729596	6	HLA-DQB2 (+1687), HLA-DQA2 (+20461)
R.1716	32847811	32847845	6	PSMB9 (+25890), HLA-DMB (+61019)
R.1723	32945423	32945906	6	BRD2 (+6987), HLA-DOA (+31724)
R.1724	33048254	33048809	6	HLA-DPA1 (-45)
R.1725	33085063	33085521	6	HLA-DPA1 (-36805), COL11A2 (+74984)
R.1727	33128825	33129024	6	HLA-DPA1 (-80438), COL11A2 (+31351)
R.1729	33161211	33161336	6	COL11A2 (-998)
R.1730	33161438	33161589	6	COL11A2 (-1238)
R.1732	33163202	33163347	6	COL11A2 (-2999)
R.1734	33174761	33175225	6	RING1 (-1279)
R.1742	33265267	33265322	6	WDR46 (-7991), RGL2 (+1806)
R.1744	33280052	33280159	6	RGL2 (-13005), TAPBP (+1883)
R.1745	33282867	33282896	6	TAPBP (-893)
R.1746	33283064	33283189	6	TAPBP (-1138)
R.1751	33386985	33387089	6	CUTA (-943), SYNGAP1 (-904)
R.1752	33400543	33401542	6	ZBTB9 (-21313), SYNGAP1 (+13102)
R.1754	34984836	34984953	6	TCP11 (+124529), ANKS1A (+127853)
R.1755	35027147	35027324	6	TCP11 (+82188), ANKS1A (+170194)
R.1756	35108061	35108605	6	TCP11 (+1091), ANKS1A (+251291)
R.1757	35285967	35286360	6	PPARD (-24227), DEF6 (+20535)
R.1759	36390970	36391250	6	ETV7 (-35550), KCTD20 (-19434)
R.1760	36645100	36645886	6	CDKN1A (-994)
R.1762	39760647	39761595	6	DAAM2 (+327)
R.1763	39902533	39903199	6	MOCS1 (-7411), LRFN2 (+652338)
R.1765	41020847	41021161	6	APOBEC2 (-39)
R.1774	41472032	41472416	6	FOXP4 (-41940), NCR2 (+168697)
R.1775	41516162	41517062	6	MDFI (-89573), FOXP4 (+2448)
R.1778	42788563	42789053	6	TBCC (-74250), RPL7L1 (-58565)
R.1779	43214396	43214590	6	SLC22A7 (-51509), TTBK1 (+3075)
R.1781	43736807	43737136	6	VEGFA (-1472)

R.1788	46293571	46294097	6	ENPP5 (-155126), RCAN2 (+165265)
R.1789	46703468	46703670	6	PLA2G7 (-490)
R.1790	47444699	47445060	6	CD2AP (-645)
R.1791	50680997	50681715	6	TFAP2D (-185)
R.1792	52440429	52441305	6	TRAM2 (+846)
R.1794	52858837	52859107	6	GSTA3 (-84489), GSTA4 (+1204)
R.1795	56112548	56112696	6	COL21A1 (-78)
R.1797	56878397	56878665	6	DST (-370737), KIAA1586 (-32816)
R.1798	70500634	70501081	6	LMBRD1 (+6145)
R.1799	70506758	70507230	6	LMBRD1 (+9)
R.1800	70576168	70576502	6	COL19A1 (-128)
R.1801	72130755	72131020	6	RIMS1 (-465839), OGFRL1 (+132382)
R.1802	74008821	74009455	6	KHDC1 (+10800), KCNQ5 (+677618)
R.1803	74404526	74405048	6	CD109 (-1040)
R.1804	75794800	75795067	6	COL12A1 (+120833)
R.1805	76059726	76059773	6	SENP6 (-252013), TMEM30A (-65066)
R.1806	76203530	76204308	6	TMEM30A (-209235), SENP6 (-107844)
R.1807	84742998	84743033	6	MRAP2 (-459)
R.1808	85824168	85824216	6	TBX18 (-349955), NT5E (-335617)
R.1809	88031886	88032061	6	SMIM8 (-332)
R.1811	89828097	89828674	6	SRSF12 (-586)
R.1813	91006647	91007625	6	BACH2 (-675)
R.1820	109777592	109778433	6	MICAL1 (-823)
R.1822	111197245	111197834	6	GTF3C6 (-82223), AMD1 (+1567)
R.1825	112575705	112575966	6	LAMA4 (+81)
R.1826	116381609	116382179	6	FRK (+27)
R.1827	116575894	116576566	6	TSPYL4 (-969)
R.1828	116691382	116691685	6	DSE (+13)
R.1831	132271361	132271588	6	CTGF (+1038), ENPP1 (+142319)
R.1832	132272790	132273157	6	CTGF (-461)
R.1840	138866865	138867125	6	ECT2L (-250068), HEBP2 (+141639)
R.1841	138892911	138893677	6	ECT2L (-223769), HEBP2 (+167938)
R.1842	139117274	139117543	6	ECT2L (+346)
R.1844	143928514	143929511	6	FUCA2 (-96186), PHACTR2 (-70089)
R.1845	144329887	144330162	6	PLAGL1 (-39910), SF3B5 (+86729)
R.1846	149772707	149772892	6	ZC3H12D (+33397), SUMO4 (+51305)
R.1849	151346268	151346409	6	AKAP12 (-214795), MTHFD1L (+159654)
R.1850	151560927	151560992	6	AKAP12 (-174)
R.1851	152126736	152126938	6	ESR1 (+115206), SYNE1 (+831697)
R.1852	152702330	152702660	6	SYNE1 (+256039), ESR1 (+690864)
R.1854	156717406	156718398	6	NOX3 (-940865), ARID1B (-381161)
R.1855	157505432	157505996	6	TMEM242 (+238919), ARID1B (+406651)
R.1857	158375684	158375814	6	SYNJ2 (-27170), SNX9 (+131453)
R.1858	158464398	158464852	6	SYNJ2 (+61706), SERAC1 (+124677)
R.1859	158733508	158734420	6	TULP4 (+272)
R.1861	160023626	160023927	6	SOD2 (+90583), FNDC1 (+433348)
R.1862	160182184	160182447	6	ACAT2 (+956)
R.1864	160541976	160542770	6	SLC22A1 (-448)
R.1865	160680034	160680851	6	SLC22A2 (-395)
R.1866	163147737	163148022	6	PACRG (-284), PARK2 (+923)

R.1875	167104601	167104926	6	MPC1 (-308278), RPS6KA2 (+171275)
R.1881	168045556	168045888	6	TCP10 (-247768), C6orf123 (+151817)
R.1887	168782541	168782650	6	DACT2 (-62194), SMOC2 (-59235)
R.1888	168840641	168841399	6	SMOC2 (-811)
R.1889	168842035	168842940	6	SMOC2 (+657)
R.1891	168936523	168936684	6	SMOC2 (+94773), THBS2 (+717535)
R.1892	169001953	169002120	6	SMOC2 (+160206), THBS2 (+652102)
R.1893	169050956	169051431	6	SMOC2 (+209363), THBS2 (+602945)
R.1894	169558175	169559061	6	THBS2 (+95521), SMOC2 (+716787)
R.1895	169629714	169630013	6	THBS2 (+24275), SMOC2 (+788033)
R.1897	169852497	169852806	6	C6orf120 (-249605), THBS2 (-198513)
R.1899	170065891	170066646	6	THBS2 (-412130), C6orf120 (-35988)
R.1900	170191057	170191082	6	C6orf70 (+39349), DLL1 (+408491)
R.1902	170411557	170411953	6	DLL1 (+187806), C6orf70 (+260034)
R.1904	170449417	170449587	6	DLL1 (+150059), C6orf70 (+297781)
R.1910	142966	143220	7	FAM20C (-49876)
R.1911	544525	544924	7	PDGFA (+14306), FAM20C (+351756)
R.1912	553693	553810	7	PDGFA (+5279), FAM20C (+360783)
R.1914	597568	597596	7	PDGFA (-38551), PRKAR1B (+155262)
R.1915	608975	609820	7	PDGFA (-50367), PRKAR1B (+143446)
R.1917	640338	641028	7	PDGFA (-81652), PRKAR1B (+112161)
R.1918	788061	788989	7	SUN1 (-83613), HEATR2 (+22187)
R.1919	810958	811786	7	SUN1 (-60766), HEATR2 (+45034)
R.1920	871436	872208	7	SUN1 (-316)
R.1921	905795	906779	7	GET4 (-9902), SUN1 (+34149)
R.1924	1022675	1023156	7	CYP2W1 (+81)
R.1925	1025760	1026363	7	GPR146 (-68859), CYP2W1 (+3227)
R.1927	1066650	1067447	7	GPR146 (-27872), CYP2W1 (+44214)
R.1928	1094263	1095005	7	GPR146 (-287)
R.1930	1135190	1136023	7	GPER (+9164), ZFAND2A (+64207)
R.1931	1163456	1163642	7	ZFAND2A (+36265), GPER (+37106)
R.1932	1250038	1250126	7	ZFAND2A (-50268), UNCX (-22461)
R.1933	1250258	1250425	7	ZFAND2A (-50528), UNCX (-22201)
R.1934	1250756	1251032	7	ZFAND2A (-51080), UNCX (-21649)
R.1938	1329387	1329462	7	UNCX (+56882), MICALL2 (+169713)
R.1944	1576174	1577016	7	MAFK (+6245), TMEM184A (+19471)
R.1945	1687007	1687852	7	TMEM184A (-91364), ELFN1 (-40325)
R.1950	2077057	2077345	7	MAD1L1 (+195677), ELFN1 (+349446)
R.1955	2499915	2499975	7	LFNG (-59531), CHST12 (+56722)
R.1957	2607139	2607424	7	TTYH3 (-64303), IQCE (+8650)
R.1958	2646478	2646955	7	TTYH3 (-24868), IQCE (+48085)
R.1959	2677986	2678263	7	AMZ1 (-41031), TTYH3 (+6540)
R.1960	2756996	2757616	7	AMZ1 (+38150), GNA12 (+126652)
R.1961	2772710	2773127	7	AMZ1 (+53763), GNA12 (+111039)
R.1963	4065931	4066127	7	FOXK1 (-655911), SDK1 (+724949)
R.1967	4729417	4729920	7	AP5Z1 (-85584), FOXK1 (+7729)
R.1970	4859229	4859734	7	PAPOLB (+42143), AP5Z1 (+44229)
R.1971	4885521	4885643	7	PAPOLB (+16043), AP5Z1 (+70329)
R.1972	4923615	4924010	7	RADIL (-463)
R.1976	5534951	5535934	7	TNRC18 (-72266), ACTB (+34897)

R.1977	5552460	5552734	7	TNRC18 (-89420), ACTB (+17743)
R.1978	5645051	5646023	7	FSCN1 (+13083), RNF216 (+175833)
R.1979	6199980	6200140	7	USP42 (+55545), CYTH3 (+112215)
R.1980	6204774	6204983	7	USP42 (+60364), CYTH3 (+107396)
R.1982	16505094	16505664	7	SOSTDC1 (+95)
R.1984	18126994	18127503	7	HDAC9 (-408677), PRPS1L1 (-59763)
R.1988	25702709	25703644	7	NFE2L3 (-488683), NPVF (-435072)
R.1990	25989524	25989735	7	NPVF (-721525), NFE2L3 (-202230)
R.1992	27150262	27150598	7	HOXA2 (-8000), HOXA3 (+8784)
R.1993	27153212	27153847	7	HOXA2 (-11100), HOXA3 (+5684)
R.1994	27163331	27164285	7	HOXA3 (-4594)
R.1995	27169136	27169957	7	HOXA4 (+871)
R.1996	27170388	27170880	7	HOXA4 (-216)
R.1997	27171203	27171528	7	HOXA4 (-948)
R.1998	27178861	27179432	7	HOXA4 (-8729), HOXA5 (+4140)
R.1999	27180888	27181671	7	HOXA4 (-10862), HOXA5 (+2007)
R.2000	27182493	27183133	7	HOXA5 (+474)
R.2001	27183369	27183436	7	HOXA5 (-116)
R.2002	27183643	27183694	7	HOXA5 (-382)
R.2003	27184188	27184369	7	HOXA5 (-992)
R.2004	27184441	27184667	7	HOXA5 (-1267)
R.2005	27184737	27185282	7	HOXA5 (-1723)
R.2006	27186554	27186993	7	HOXA5 (-3487), HOXA6 (+619)
R.2013	27497452	27497483	7	HIBADH (+205146), EVX1 (+215304)
R.2014	28319343	28319594	7	CREB5 (-132675), JAZF1 (-99107)
R.2015	28338625	28338985	7	JAZF1 (-118443), CREB5 (-113339)
R.2016	28452098	28452289	7	CREB5 (+50)
R.2017	29519323	29519656	7	CPVL (-284598), PRR15 (-83937)
R.2018	29923883	29924746	7	WIPF3 (+49974), SCRN1 (+105590)
R.2020	30791871	30792301	7	INMT-FAM188B (+333), INMT (+335)
R.2021	30950813	30951272	7	AQP1 (-427)
R.2022	30951709	30951801	7	AQP1 (+285)
R.2023	32111062	32111068	7	PDE1C (+227876), PPP1R17 (+384736)
R.2024	35292896	35293130	7	TBX20 (+745)
R.2029	36193854	36194394	7	ANLN (-235291), EEPD1 (+1366)
R.2033	41745737	41746034	7	INHBA (-3180)
R.2035	44104860	44105166	7	PGAM2 (+173)
R.2036	44105434	44105764	7	PGAM2 (-413)
R.2037	44795713	44796036	7	PPIA (-40404), ZMIZ2 (+7345)
R.2039	45002287	45002919	7	PURB (-77643), MYO1G (+16094)
R.2047	50861592	50861654	7	GRB10 (-61573), COBL (+522873)
R.2049	55089770	55090707	7	LANCL2 (-342902), EGFR (+3445)
R.2051	56160409	56161020	7	PHKG1 (-71)
R.2054	64349915	64350876	7	ZNF273 (-13229), ZNF138 (+95593)
R.2056	65196614	65196881	7	VKORC1L1 (-141506), ZNF92 (+358036)
R.2058	65970924	65971710	7	KCTD7 (-234326), TPST1 (+301131)
R.2059	70061455	70061616	7	WBSCR17 (-535619), AUTS2 (+997217)
R.2060	70096029	70096791	7	WBSCR17 (-500745)
R.2061	70597091	70597599	7	WBSCR17 (+190)
R.2063	73389575	73389660	7	ELN (-52501), WBSCR28 (+114129)

R.2064	73392697	73393148	7	ELN (-49196), WBSCR28 (+117434)
R.2065	73441057	73441834	7	ELN (-673)
R.2066	73442487	73443113	7	ELN (+681)
R.2068	73894884	73895061	7	GTF2I (-177038), GTF2IRD1 (+26673)
R.2071	75596021	75596055	7	POR (+51641), STYXL1 (+81283)
R.2073	75624427	75624699	7	STYXL1 (+52758), POR (+80166)
R.2074	75779470	75779779	7	HSPB1 (-152236), MDH2 (+102240)
R.2075	76026556	76026648	7	YWHAG (-38254), SRCRB4D (+12410)
R.2076	89841435	89841767	7	STEAP2 (+601)
R.2077	90895466	90896329	7	FZD1 (+2115), MTERF (+614136)
R.2078	92238086	92238248	7	RBM48 (+80080), CDK6 (+225064)
R.2079	92672812	92673176	7	CDK6 (-209763), SAMD9 (+74342)
R.2082	97663876	97663940	7	LMTK2 (-72289), OCM2 (-44400)
R.2083	97839870	97840113	7	BHLHA15 (-1574)
R.2084	97911938	97912641	7	BRI3 (+1303), BAIAP2L1 (+118090)
R.2085	98246633	98247587	7	NPTX2 (+501)
R.2086	98424185	98424445	7	TMEM130 (+43174), NPTX2 (+177706)
R.2088	98739496	98739782	7	SMURF1 (+2084), TRRAP (+263526)
R.2089	98990265	98991138	7	BUD31 (-15562), ARPC1B (+18349)
R.2090	99063876	99063916	7	ATP5J2-PTCD1 (-109), ATP5J2 (+58)
R.2091	99691127	99691611	7	AP4M1 (-7811), COPS6 (+4792)
R.2093	99775422	99775521	7	GPC2 (-482), STAG3 (+286)
R.2096	100239307	100240094	7	TFR2 (+643)
R.2097	100253792	100253914	7	ACTL6B (+231)
R.2099	100304425	100304693	7	POP7 (+883)
R.2101	100880751	100881286	7	PLOD3 (-20065), CLDN15 (+1082)
R.2103	101558400	101558698	7	SH2B2 (-369856), CUX1 (+99258)
R.2104	101579003	101579936	7	SH2B2 (-348935), CUX1 (+120179)
R.2106	101768610	101768874	7	SH2B2 (-159663), CUX1 (+309451)
R.2108	101936318	101936527	7	PRKRIP1 (-67921), SH2B2 (+8018)
R.2109	101943990	101944556	7	PRKRIP1 (-60071), SH2B2 (+15868)
R.2110	101961796	101962123	7	PRKRIP1 (-42384), SH2B2 (+33555)
R.2111	102105641	102105781	7	ALKBH4 (-388), LRWD1 (+335)
R.2112	102574105	102574504	7	ARMC10 (-141023), LRRC17 (+20867)
R.2114	105319437	105319558	7	CDHR3 (-284187), EFCAB10 (-97555)
R.2115	105319679	105320151	7	CDHR3 (-283770), EFCAB10 (-97972)
R.2117	107220040	107220262	7	BCAP29 (-1053)
R.2118	110730805	110731527	7	LRRN3 (+104)
R.2119	112120799	112121036	7	LSMEM1 (-128)
R.2120	113727753	113728506	7	FOXP2 (-326922), PPP1R3A (-169010)
R.2121	114055074	114055419	7	FOXP2 (+195)
R.2122	114561804	114562063	7	MDFIC (-275)
R.2123	116138462	116139180	7	CAV2 (-623)
R.2124	116592956	116593113	7	ST7 (-346)
R.2125	116660172	116660240	7	ST7 (+66825), WNT2 (+303137)
R.2128	127228532	127228839	7	GCC1 (-3025), ARF5 (+287)
R.2129	127880932	127881269	7	LEP (-236)
R.2130	127910927	127911367	7	LEP (+29810), RBM28 (+72815)
R.2131	128432231	128432457	7	CCDC136 (+245)
R.2134	129780421	129781152	7	TMEM209 (+64551), KLHDC10 (+70437)

R.2136	130019736	130020130	7	CPA1 (-279)
R.2137	130606228	130606499	7	MKLN1 (-406255), KLF14 (-187476)
R.2138	132299913	132300110	7	PLXNA4 (-37952), CHCHD3 (+466830)
R.2139	134250149	134250389	7	BPGM (-81314), AKR1B15 (+16381)
R.2140	134463017	134463703	7	CALD1 (-1041)
R.2141	134464123	134464411	7	CALD1 (-134)
R.2142	134574947	134575811	7	AGBL3 (-95880), CALD1 (+110978)
R.2143	134856562	134856681	7	C7orf49 (-1175)
R.2144	135433353	135433790	7	FAM180A (+22)
R.2146	138720898	138720989	7	KIAA1549 (-54880), ZC3HAV1 (+73450)
R.2148	142554132	142554834	7	EPHB6 (+1691), TRPV6 (+29024)
R.2149	142582859	142583731	7	TRPV6 (+212)
R.2153	150038502	150038898	7	RARRES2 (+32)
R.2158	150778340	150778926	7	FASTK (-702)
R.2159	150822006	150822987	7	AGAP3 (+38668), GBX1 (+42138)
R.2163	151442371	151442967	7	RHEB (-225659), PRKAG2 (+131541)
R.2164	151453891	151454795	7	RHEB (-237333), PRKAG2 (+119867)
R.2167	152591896	152592038	7	ACTR3B (+135133)
R.2168	153584416	153584839	7	DPP6 (-165137)
R.2175	156259194	156259219	7	SHH (-654240), C7orf13 (+174141)
R.2176	156433317	156433350	7	SHH (-828367), RNF32 (-144), C7orf13 (+14)
R.2177	156735383	156735656	7	LMBR1 (-49596), NOM1 (-6897)
R.2178	157178981	157179830	7	DNAJB6 (+49746)
R.2180	157322117	157322652	7	DNAJB6 (+192725)
R.2182	157348161	157348266	7	DNAJB6 (+218554)
R.2184	157453775	157454207	7	DNAJB6 (+324331), PTPRN2 (+926380)
R.2186	157633252	157633985	7	DNAJB6 (+503959), PTPRN2 (+746752)
R.2191	157667762	157667935	7	DNAJB6 (+538189), PTPRN2 (+712522)
R.2192	157776346	157776679	7	PTPRN2 (+603858), DNAJB6 (+646853)
R.2193	157809235	157809385	7	PTPRN2 (+571061), DNAJB6 (+679650)
R.2195	158533877	158533927	7	NCAPG2 (-36407), ESYT2 (+88417)
R.2196	158575681	158575934	7	NCAPG2 (-78313), ESYT2 (+46511)
R.2199	158807362	158808009	7	VIPR2 (+129963), WDR60 (+158417)
R.2200	158886120	158886269	7	VIPR2 (+51454), WDR60 (+236926)
R.2202	637468	638208	8	DLGAP2 (-811694), TDRP (-142057)
R.2205	1080058	1080669	8	TDRP (-584583), DLGAP2 (-369168)
R.2210	1707330	1707553	8	CLN8 (-4486)
R.2213	1777477	1778042	8	MYOM2 (-215395), ARHGEF10 (+5618)
R.2214	1796429	1796568	8	MYOM2 (-196656), ARHGEF10 (+24357)
R.2217	1893977	1894814	8	MYOM2 (-98759), ARHGEF10 (+122254)
R.2218	1895007	1895558	8	MYOM2 (-97872), ARHGEF10 (+123141)
R.2219	1906312	1906498	8	MYOM2 (-86750), ARHGEF10 (+134263)
R.2228	4849522	4850180	8	CSMD1 (+2087)
R.2229	6420858	6421171	8	ANGPT2 (-85)
R.2230	6693367	6693540	8	DEFB1 (+42090), AGPAT5 (+127576)
R.2232	8084610	8085544	8	ENSG00000182319 (+158931), ZNF705B (+283933)
R.2233	8749919	8750314	8	ERI1 (-109540), CLDN23 (+190669)
R.2235	10530053	10530148	8	C8orf74 (-46)
R.2242	12958083	12958533	8	KIAA1456 (+155122), DLC1 (+414087)
R.2243	13372483	13373141	8	DLC1 (-417)

R.2246	19459672	19460243	8	CSGALNACT1 (+48)
R.2249	21882439	21882942	8	FGF17 (-17246), NPM2 (+1046)
R.2250	21915510	21916355	8	DMTN (-784)
R.2251	21916635	21916853	8	DMTN (+27)
R.2252	22084861	22085227	8	POLR3D (-17704), BMP1 (+62795)
R.2253	22446721	22447181	8	ENSG00000248235 (+164)
R.2254	22458046	22458182	8	KIAA1967 (-4031), C8orf58 (+954)
R.2255	22547688	22548546	8	BIN3 (-21484), EGR3 (+2698)
R.2256	22548828	22549103	8	BIN3 (-22333), EGR3 (+1849)
R.2257	22735111	22735333	8	EGR3 (-184407), PEBP4 (+50199)
R.2258	22785257	22786190	8	PEBP4 (-303)
R.2265	24772309	24772344	8	DOCK5 (-270053), NEFM (+1802)
R.2271	27347372	27348177	8	EPHX2 (-521)
R.2272	27468684	27469001	8	CLU (+3705), EPHX2 (+120547)
R.2273	27472446	27473422	8	CLU (-386)
R.2275	27850175	27850215	8	SCARA5 (+49)
R.2277	28244386	28244769	8	ZNF395 (-595)
R.2278	28928948	28929689	8	HMBOX1 (+181398), KIF13B (+191271)
R.2280	30240920	30241167	8	RBPMS (-983)
R.2281	30413794	30413919	8	SMIM18 (-82260), RBPMS (+171830)
R.2283	36641771	36641924	8	KCNU1 (-44)
R.2287	37698900	37699507	8	BRF2 (+8218), GPR124 (+44430)
R.2288	37823979	37824340	8	ADRB3 (+323)
R.2289	38386235	38386476	8	TACC1 (-258366), FGFR1 (-60993)
R.2291	38964944	38965014	8	ADAM32 (-189)
R.2292	41166738	41167087	8	SFRP1 (+103)
R.2294	41517301	41518139	8	NKX6-3 (-12842), ANK1 (+236560)
R.2296	42064673	42065244	8	PLAT (+283)
R.2298	48648112	48649086	8	CEBPD (+3049), SPIDR (+475432)
R.2300	49340641	49341022	8	EFCAB1 (+306959), UBE2V2 (+419872)
R.2301	49467831	49468341	8	EFCAB1 (+179705), UBE2V2 (+547126)
R.2302	49493865	49494724	8	EFCAB1 (+153496), UBE2V2 (+573335)
R.2303	49647717	49647809	8	EFCAB1 (+28)
R.2304	49783056	49783652	8	EFCAB1 (-135563), SNAI2 (+50945)
R.2305	49984564	49985021	8	C8orf22 (-120)
R.2306	52721657	52722539	8	PXDNL (-93)
R.2308	57358240	57358505	8	PENK (+216)
R.2309	57360586	57360711	8	PENK (-2060)
R.2310	61835620	61836533	8	CLVS1 (-364437), CHD7 (+244740)
R.2311	67038272	67039155	8	TRIM55 (-417)
R.2312	67405405	67405742	8	ADHFE1 (+60853), MYBL1 (+119910)
R.2313	67454546	67454667	8	MYBL1 (+70877), ADHFE1 (+109886)
R.2315	71316696	71316869	8	NCOA2 (-743)
R.2316	71520941	71520989	8	TRAM1 (-361)
R.2319	75735552	75736301	8	PI15 (-845)
R.2322	93074929	93075561	8	RUNX1T1 (+32461), SLC26A7 (+813710)
R.2324	95962352	95962463	8	TP53INP1 (-769)
R.2328	97658076	97658774	8	CPQ (+970)
R.2329	98290359	98290372	8	TSPYL5 (-190)
R.2331	101117949	101118083	8	RGS22 (+328)

R.2332	101348456	101348501	8	RNF19A (-32992), ANKRD46 (+223491)
R.2333	101661991	101662246	8	SNX31 (-226)
R.2334	105479215	105479318	8	DPYS (+14)
R.2335	105599835	105600724	8	LRP12 (+954)
R.2336	107782513	107782632	8	ABRA (-100)
R.2337	110374563	110374585	8	PKHD1L1 (-132)
R.2338	110656096	110656245	8	SYBU (+47849), EBAG9 (+103765)
R.2339	119086580	119086762	8	EXT1 (+37421), MED30 (+553719)
R.2341	120220484	120220882	8	MAL2 (+73)
R.2342	120685317	120685686	8	ENPP2 (-34432), TAF2 (+159601)
R.2343	121138133	121138317	8	COL14A1 (+878)
R.2346	130995990	130996123	8	FAM49B (-43979), ASAP1 (+418160)
R.2347	132052843	132052887	8	ASAP1 (-638648), ADCY8 (+1807)
R.2349	134114834	134115661	8	WISP1 (-88034), SLA (-42645)
R.2350	134202337	134203304	8	WISP1 (-461)
R.2351	134203339	134203841	8	WISP1 (+308)
R.2352	134580580	134581043	8	NDRG1 (-270983), ST3GAL1 (+3371)
R.2353	141559367	141559946	8	CHRAC1 (+38260), AGO2 (+86061)
R.2355	141599208	141599436	8	AGO2 (+46396), CHRAC1 (+77925)
R.2357	142368026	142368332	8	SLC45A4 (-129506), GPR20 (+9188)
R.2365	143823898	143824001	8	SLURP1 (-121)
R.2366	143851287	143852085	8	LYPD2 (-17734), LYNX1 (+7954)
R.2367	143879727	143880283	8	GML (-36212), LY6D (-11997)
R.2368	144120484	144120706	8	LY6E (+20650), LY6H (+121533)
R.2374	144610522	144610644	8	MAFA (-98007), ZC3H3 (+13040)
R.2376	144649510	144650176	8	NAPRT1 (+10940), GSDMD (+14466)
R.2377	144652042	144652930	8	NAPRT1 (+8297), GSDMD (+17109)
R.2380	144789823	144790772	8	MAPK15 (-8131), ZNF707 (+23620)
R.2381	144924893	144925333	8	NRBP2 (-1967)
R.2382	144935734	144935835	8	NRBP2 (-12639), EPPK1 (+16847)
R.2383	144948315	144948781	8	NRBP2 (-25402), EPPK1 (+4084)
R.2388	145018816	145019116	8	EPPK1 (-66334), PLEC (+6078)
R.2389	145024929	145025123	8	PLEC (+18)
R.2390	145027741	145028103	8	PLEC (-2878)
R.2391	145033104	145033743	8	PLEC (-8380), PARP10 (+27198)
R.2392	145048137	145048363	8	PLEC (-23206), PARP10 (+12372)
R.2394	145562760	145563658	8	SCRT1 (-3266)
R.2395	145577929	145578141	8	SLC52A2 (-4218), TMEM249 (+539)
R.2396	145725708	145726688	8	GPT (-3267)
R.2397	145727318	145728285	8	GPT (-1663)
R.2398	145728490	145729106	8	GPT (-667)
R.2399	145755615	145755804	8	LRRC24 (-3308), C8orf82 (-1194)
R.2401	3180374	3181007	9	KIAA0020 (-336450), RFX3 (+345292)
R.2403	34457129	34457500	9	DNAI1 (-1518)
R.2404	34458891	34459568	9	DNAI1 (+397)
R.2405	35563312	35564052	9	TESK1 (-41685), RUSC2 (+25053)
R.2406	35649906	35650561	9	SIT1 (+703)
R.2407	35791475	35792342	9	NPR2 (-242)
R.2408	36166097	36166258	9	CCIN (-3211)
R.2410	85677921	85678251	9	RASEF (-43)

R.2412	94183343	94184131	9	AUH (-59542), NFIL3 (+2407)
R.2413	97847913	97848736	9	FANCC (+231659), C9orf3 (+326259)
R.2415	98811152	98811640	9	ERCC6L2 (+173413), HSD17B3 (+253038)
R.2416	101568975	101569334	9	GALNT12 (-826)
R.2417	101705792	101705939	9	COL15A1 (-55)
R.2418	117068159	117068532	9	ORM1 (-16990), COL27A1 (+150506)
R.2419	117156701	117157614	9	AKNA (-473)
R.2421	123655887	123656764	9	PHF19 (-16720), TRAF1 (+34721)
R.2422	124029872	124030048	9	RAB14 (-65813), GSN (-32111)
R.2423	124988720	124989337	9	NDUFA8 (-66931), LHX6 (+2095)
R.2426	129261375	129262204	9	LMX1B (-114932), MVB12B (+172662)
R.2427	129433779	129434406	9	ZBTB43 (-133201), LMX1B (+57371)
R.2428	129884026	129884457	9	ANGPTL2 (+920)
R.2429	130516695	130517095	9	TOR2A (-19299), SH2D3C (+24125)
R.2431	130700442	130700923	9	DPM2 (+80)
R.2433	131038362	131038602	9	GOLGA2 (-214), SWI5 (+57)
R.2435	131965610	131965921	9	NTMT1 (-405397), PPP2R4 (+92169)
R.2438	133759967	133760878	9	QRFP (+8802), ABL1 (+171090)
R.2440	136324577	136324795	9	CACFD1 (-426)
R.2442	138987290	138987845	9	NACC2 (-45142), C9orf69 (+23141)
R.2443	139092321	139092974	9	C9orf69 (-81939), LHX3 (+2356)
R.2447	139715701	139716215	9	PHPT1 (-27588), RABL6 (+13584)
R.2448	139744532	139745116	9	MAMDC4 (-1995)
R.2450	140342917	140343854	9	ENTPD8 (-7485), NSMF (+10400)
R.2451	140352188	140353134	9	ENTPD8 (-16760), NSMF (+1125)
R.2453	140473680	140473875	9	DPH7 (-391)
R.2455	375248	375830	10	ZMYND11 (+195115), DIP2C (+360067)
R.2458	415359	416315	10	ZMYND11 (+235413), DIP2C (+319769)
R.2459	435953	436626	10	ZMYND11 (+255866), DIP2C (+299316)
R.2461	555957	556762	10	DIP2C (+179246), ZMYND11 (+375936)
R.2462	560323	560669	10	DIP2C (+175110), ZMYND11 (+380072)
R.2464	664719	664994	10	DIP2C (+70749), ZMYND11 (+484433)
R.2465	678920	679221	10	DIP2C (+56535), ZMYND11 (+498647)
R.2466	695844	696063	10	DIP2C (+39652), ZMYND11 (+515530)
R.2467	729479	729916	10	DIP2C (+5908), ZMYND11 (+549274)
R.2472	2776888	2777128	10	ADARB2 (-997338), PFKP (-332704)
R.2473	3149673	3149852	10	PFKP (+40051), PITRM1 (+65240)
R.2475	3280494	3281074	10	PITRM1 (-65781), KLF6 (+546683)
R.2477	3581101	3581233	10	PITRM1 (-366164), KLF6 (+246300)
R.2478	3789537	3789974	10	PITRM1 (-574753), KLF6 (+37711)
R.2483	6233666	6233774	10	PFKFB3 (-11174), RBM17 (+102411)
R.2485	6264090	6264776	10	PFKFB3 (+19539), PRKCQ (+357830)
R.2486	6274873	6275077	10	PFKFB3 (+30081), PRKCQ (+347288)
R.2488	11866199	11866971	10	ECHDC3 (+82220), UPF2 (+211311)
R.2490	13388442	13388567	10	PHYH (-46404), SEPHS1 (+1792)
R.2491	14051636	14051821	10	FRMD4A (+321154), PRPF18 (+422802)
R.2492	18629497	18629677	10	CACNB2 (+199981), NSUN6 (+310964)
R.2493	21804624	21805402	10	NEBL (-618482), SKIDA1 (+9598)
R.2494	22623213	22624094	10	SPAG6 (-10745), BMI1 (+13514)
R.2495	22843607	22843938	10	PIP4K2A (+159711), SPAG6 (+209374)

R.2496	24496598	24496943	10	KIAA1217 (-1322)
R.2498	26855907	26856128	10	PDSS1 (-130570), APBB1IP (+128886)
R.2499	29759227	29759454	10	LYZL1 (+181351), SVIL (+265389)
R.2502	29923736	29924258	10	SVIL (+100733), LYZL1 (+346007)
R.2503	30316933	30317688	10	SVIL (-292581), KIAA1462 (+31142)
R.2504	30348709	30349405	10	KIAA1462 (-604)
R.2506	31107995	31108190	10	LYZL2 (-189402), ZNF438 (+212773)
R.2507	31610093	31610754	10	ZEB1 (+2323), ARHGAP12 (+607311)
R.2508	32216031	32216359	10	ARHGAP12 (+1540), ZEB1 (+608094)
R.2511	44068714	44069360	10	ZNF485 (-32818), ZNF239 (-5130)
R.2515	45406644	45406764	10	TMEM72 (+56)
R.2516	45675577	45675680	10	C10orf25 (-179293), OR13A1 (+135427)
R.2517	46970584	46970625	10	SYT15 (-90)
R.2519	49674458	49674883	10	ARHGAP22 (+138326), MAPK8 (+159938)
R.2524	50323825	50323832	10	VSTM4 (-275)
R.2526	50506748	50507003	10	C10orf128 (-110472), DRGX (+93031)
R.2527	50887731	50887934	10	CHAT (+65750), OGDHL (+82536)
R.2528	56560945	56561134	10	PCDH15 (+11)
R.2530	63212206	63212496	10	TMEM26 (+857)
R.2531	63213385	63213784	10	TMEM26 (-377)
R.2532	63422254	63422841	10	ARID5B (-238511), TMEM26 (-209340)
R.2534	70979777	70980112	10	HKDC1 (-114)
R.2535	71176501	71176853	10	TACR2 (-54)
R.2537	72311914	72312093	10	PRF1 (+50527), PALD1 (+73427)
R.2541	73769127	73769828	10	CHST3 (+45355), SPOCK2 (+79053)
R.2543	75407634	75407991	10	MYOZ1 (-6298), SYNPO2L (+8017)
R.2544	75415704	75415875	10	SYNPO2L (+40)
R.2545	76573155	76573506	10	KAT6B (-13052), ADK (+662344)
R.2546	77164227	77165167	10	ZNF503 (-3033)
R.2547	77165293	77165905	10	ZNF503 (-3935)
R.2548	77352231	77352376	10	ZNF503 (-190640), C10orf11 (-190215)
R.2549	77542314	77542488	10	C10orf11 (-118)
R.2550	77794591	77794696	10	C10orf11 (+252125)
R.2551	78870177	78870221	10	KCNMA1 (+527201)
R.2552	78943401	78943634	10	KCNMA1 (+453882)
R.2553	80853937	80854035	10	PPIF (-253248), ZMIZ1 (+25194)
R.2555	81966990	81967666	10	ANXA11 (-2000)
R.2560	88295210	88295591	10	OPN4 (-118913), WAPAL (-13829)
R.2561	88427400	88428295	10	LDB3 (-358)
R.2562	88716646	88717364	10	SNCG (-1408), MMRN2 (+367)
R.2563	88718164	88718393	10	MMRN2 (-907), SNCG (-134)
R.2564	88727732	88728128	10	ADIRF (-19)
R.2565	88729315	88729861	10	ADIRF (+1639), GLUD1 (+125035)
R.2566	90712478	90712748	10	FAS (-37801), STAMBPL1 (+73122)
R.2567	93805441	93805682	10	BTAF1 (+122036), CPEB3 (+245282)
R.2568	94351376	94351614	10	KIF11 (-1548)
R.2569	94455543	94455895	10	EXOC6 (-152558), HHEX (+7774)
R.2570	94819989	94820376	10	CYP26C1 (-838)
R.2571	95516908	95517463	10	LGI1 (-456)
R.2573	97175272	97175479	10	PDLIM1 (-124595), SORBS1 (+145759)

R.2574	98031125	98031261	10	BLNK (+104)
R.2577	99477939	99478719	10	ZFYVE27 (-19905), MARVELD1 (+4848)
R.2579	99734513	99734805	10	CRTAC1 (+55926), GOLGA7B (+124663)
R.2583	102278918	102279791	10	SEC31B (+236)
R.2584	102642531	102642752	10	SEMA4G (-89950), PAX2 (+137174)
R.2585	102760724	102760970	10	LZTS2 (+1614), PDZD7 (+30043)
R.2586	102821565	102821684	10	KAZALD1 (+27)
R.2595	104535792	104536035	10	WBP1L (+32187), CYP17A1 (+61376)
R.2597	105420831	105421480	10	NEURL (+167420), SH3PXD2A (+194008)
R.2599	106093778	106094166	10	ITPRIP (-309)
R.2600	111765673	111766049	10	ADD3 (-1859)
R.2604	112404738	112404997	10	RBM20 (+713)
R.2605	112630726	112630835	10	PDCD4 (-784)
R.2606	118765255	118765597	10	KIAA1598 (-338)
R.2608	118899288	118899407	10	VAX1 (-1781)
R.2613	120354081	120354785	10	PRLHR (+727)
R.2614	120355419	120355756	10	PRLHR (-428)
R.2615	121138105	121138206	10	RGS10 (+164064), GRK5 (+171055)
R.2616	121171859	121172463	10	RGS10 (+130059), GRK5 (+205060)
R.2621	123781519	123781602	10	PLEKHA1 (-352651), TACC2 (+32852)
R.2622	123908897	123909426	10	PLEKHA1 (-225050), TACC2 (+160453)
R.2624	124222331	124222884	10	DMBT1 (-97573), HTRA1 (+1567)
R.2627	125651621	125651726	10	CPXM2 (-344)
R.2629	126106555	126106614	10	OAT (+920)
R.2630	126278272	126278502	10	LHPP (+127983), FAM53B (+154232)
R.2632	126686658	126686891	10	CTBP2 (+29684), ZRANB1 (+56083)
R.2634	128077307	128077369	10	ADAM12 (-314)
R.2635	128994603	128994644	10	NPS (-352989), DOCK1 (+400646)
R.2645	130830901	130831421	10	MKI67 (-906512), MGMT (-434287)
R.2646	131005696	131005778	10	MGMT (-259711)
R.2648	131264597	131264840	10	MGMT (-729)
R.2652	131810674	131811642	10	GLRX3 (-123505), EBF3 (-49053)
R.2653	131989161	131989387	10	GLRX3 (+54611)
R.2654	132003854	132004214	10	GLRX3 (+69371)
R.2655	133048362	133048930	10	PPP2R2D (-699309)
R.2661	134096650	134096880	10	STK32C (+24679), DPYSL4 (+96361)
R.2668	134421030	134421801	10	INPP5A (+70092), NKX6-2 (+178140)
R.2671	134598352	134599151	10	NKX6-2 (+804)
R.2672	134600463	134600600	10	NKX6-2 (-976)
R.2675	134801360	134801824	10	NKX6-2 (-202036), GPR123 (-99817)
R.2680	134980303	134980924	10	UTF1 (-63164), KNDC1 (+6663)
R.2681	135050326	135050345	10	VENTX (-572)
R.2682	135051044	135051270	10	VENTX (+249)
R.2683	135051581	135052004	10	VENTX (+885)
R.2684	135059097	135059289	10	VENTX (+8285), ADAM8 (+31179)
R.2685	135191613	135191639	10	ECHS1 (-4433), PAOX (-1112)
R.2692	368384	368588	11	B4GALNT4 (-1318)
R.2695	504918	504937	11	RNH1 (-1358)
R.2697	818752	818892	11	PNPLA2 (-80)
R.2698	843897	844085	11	POLR2L (-1446), TSPAN4 (-455)

R.2700	980039	980634	11	AP2A2 (+54456), MUC6 (+56369)
R.2704	1463541	1463935	11	BRSK2 (+31356), MOB2 (+44238)
R.2705	1506936	1507347	11	MOB2 (+834)
R.2706	1542394	1543275	11	MOB2 (-34859), DUSP8 (+50315)
R.2708	1769289	1769522	11	IFITM10 (+2415), KRTAP5-6 (+50981)
R.2709	1785631	1785701	11	CTSD (-444)
R.2710	1848742	1849486	11	CTSD (-63892), SYT8 (-6560)
R.2714	1891872	1892180	11	TNNT3 (-48766), LSP1 (+5631)
R.2715	1929115	1929491	11	TNNT3 (-11489), LSP1 (+42908)
R.2721	2011971	2012332	11	MRPL23 (+43644), IGF2 (+150316)
R.2734	2292890	2293048	11	ASCL2 (-787)
R.2735	2293164	2293201	11	ASCL2 (-1001)
R.2740	2470969	2471097	11	KCNQ1 (+4812), CDKN1C (+436078)
R.2742	2828364	2828778	11	CDKN1C (+78540), KCNQ1 (+362350)
R.2743	2847462	2848310	11	CDKN1C (+59225), KCNQ1 (+381665)
R.2746	2904591	2904951	11	CDKN1C (+2340), KCNQ1 (+438550)
R.2747	2907670	2907754	11	CDKN1C (-601)
R.2749	2930322	2930995	11	SLC22A18AS (-5689), PHLDA2 (+20026)
R.2750	3014056	3014102	11	NAP1L4 (-472)
R.2752	3167827	3168788	11	CARS (-89641), OSBPL5 (+18276)
R.2757	3253769	3254065	11	MRGPRE (-301)
R.2759	3819306	3820252	11	NUP98 (-757)
R.2762	6462391	6462828	11	HPX (-316)
R.2764	7597814	7598673	11	PPFIBP2 (+63243), CYB5R2 (+97195)
R.2765	8361190	8361530	11	LMO1 (-75955), STK33 (+136011)
R.2767	8832111	8833047	11	ST5 (+99919), RPL27A (+128621)
R.2769	10715175	10715715	11	MRVI1 (-146)
R.2770	12031266	12031508	11	DKK3 (-758)
R.2771	12307924	12308645	11	MICALCL (-162)
R.2772	13983893	13984067	11	FAR1 (+293763), RRAS2 (+396750)
R.2773	14993642	14993818	11	CALCA (+170)
R.2776	15959602	15959856	11	SOX6 (+538206), INSC (+825759)
R.2777	16761290	16761533	11	C11orf58 (+1464), PLEKHA7 (+274547)
R.2779	17740887	17740977	11	MYOD1 (-183)
R.2781	18067256	18067927	11	TPH1 (-4720)
R.2783	18344237	18344427	11	HPS5 (-611), GTF2H1 (+189)
R.2784	18433745	18434015	11	LDHC (+26)
R.2785	19736253	19737237	11	NAV2 (+1602), DBX1 (+445125)
R.2786	20043971	20044639	11	DBX1 (+137565), NAV2 (+309162)
R.2789	27015592	27015991	11	FIBIN (+164)
R.2790	30038310	30038672	11	KCNA4 (+79)
R.2794	32008961	32009163	11	PAX6 (-169553), RCN1 (-103388)
R.2796	32421752	32421845	11	WT1 (+35377), RCN1 (+309349)
R.2804	43568922	43569269	11	HSD17B12 (-133163), API5 (+235557)
R.2806	44332340	44332385	11	ALX4 (-647)
R.2807	44642868	44642932	11	TSPAN18 (-143076), CD82 (+55759)
R.2809	44978644	44979029	11	PRDM11 (-190057), TP53I11 (-7126)
R.2811	45230868	45231545	11	PRDM11 (+62313), SYT13 (+76663)
R.2817	47376519	47376985	11	MYBPC3 (-2499)
R.2822	47470768	47470803	11	RAPSN (-56)

R.2823	47516584	47517381	11	RAPSN (-46253), CELF1 (+28557)
R.2824	47574990	47575283	11	CELF1 (-29597), PTPMT1 (-12016)
R.2825	47607760	47608047	11	FAM180B (-294)
R.2826	47611236	47612070	11	FAM180B (+3455), C1QTNF4 (+4558)
R.2827	49579997	49580310	11	OR4C13 (-393789), FOLH1 (-349932)
R.2830	57089366	57090226	11	TNKS1BP1 (-125)
R.2831	57105339	57105814	11	SSRP1 (-2226), P2RX3 (-414)
R.2832	57195025	57195540	11	SLC43A3 (-865)
R.2833	57267101	57267679	11	SLC43A1 (+15869), RTN4RL2 (+39368)
R.2834	57407842	57408751	11	YPEL4 (+9120), SERPING1 (+43306)
R.2835	58673453	58673577	11	GLYATL2 (-61258), GLYATL1 (-37207)
R.2841	60775715	60776399	11	CD5 (-93810), CD6 (+36905)
R.2842	61323284	61323667	11	SYT7 (+24822), PPP1R32 (+74884)
R.2843	61525216	61525304	11	MYRF (+5139), TMEM258 (+34822)
R.2844	61717191	61717334	11	BEST1 (-579)
R.2846	62314874	62315320	11	AHNAK (-790)
R.2847	62342512	62342730	11	EEF1G (-1176)
R.2848	62414563	62414786	11	GANAB (-588)
R.2850	62688748	62689122	11	CHRM1 (+218)
R.2852	63272554	63272909	11	LGALS12 (-1103)
R.2854	63529520	63530432	11	MARK2 (-76424), RTN3 (+81021)
R.2856	63687223	63687937	11	RCOR2 (-3264)
R.2861	64058550	64058878	11	KCNK4 (-1417)
R.2864	64374819	64375765	11	SLC22A12 (+17010), NRXN2 (+115368)
R.2865	64397333	64398015	11	SLC22A12 (+39392), NRXN2 (+92986)
R.2866	64405346	64405993	11	SLC22A12 (+47388), NRXN2 (+84990)
R.2868	64527189	64527417	11	PYGM (+466)
R.2870	64532839	64532971	11	PYGM (-5136), SF1 (+13036)
R.2876	64702921	64703510	11	GPHA2 (+144)
R.2877	65123374	65123609	11	TIGD3 (+1254), SLC25A45 (+26650)
R.2878	65246411	65247138	11	SCYL1 (-45773), FRMD8 (+92705)
R.2879	65374663	65375067	11	KCNK7 (-11398), MAP3K11 (+6848)
R.2882	65684313	65685262	11	DRAP1 (-1940)
R.2883	65816463	65816935	11	SF3B2 (-3108), GAL3ST3 (-48)
R.2884	66034681	66034922	11	RAB1B (-1202)
R.2885	66104115	66104485	11	RIN1 (-300)
R.2886	66326652	66327000	11	ZDHHC24 (-13117), CTSF (+9241)
R.2887	66410467	66411184	11	RBM4 (+4420), RBM4B (+34393)
R.2888	66649832	66650560	11	LRFN4 (+25223), PC (+75651)
R.2891	67071064	67071646	11	SSH3 (+436)
R.2893	67325980	67326225	11	CABP2 (-35236), GSTP1 (-24963)
R.2894	67373083	67373841	11	NDUFV1 (-861)
R.2895	67383802	67384040	11	NDUFV1 (+9598), NUDT8 (+13471)
R.2896	67478704	67479369	11	ALDH3B2 (-36935), UNC93B1 (+292556)
R.2900	68081226	68081607	11	PPP6R3 (-146821), LRP5 (+1340)
R.2902	68564292	68564477	11	MTL5 (-45353), CPT1A (+44999)
R.2905	68779553	68780136	11	MRGPRD (-31390), MRGPRF (+1032)
R.2906	68781279	68782211	11	MRGPRF (-868)
R.2909	68855823	68856414	11	CCND1 (-599736), TPCN2 (+39754)
R.2910	68924751	68925191	11	CCND1 (-530884), TPCN2 (+108606)

R.2915	69634296	69634372	11	FGF3 (-542)
R.2917	69924724	69925268	11	ANO1 (+588)
R.2918	70256556	70256681	11	CTTN (+11972), SHANK2 (+601753)
R.2920	70282745	70282789	11	CTTN (+38120), SHANK2 (+575605)
R.2925	70557445	70557881	11	SHANK2 (+300709), CTTN (+313016)
R.2928	71724287	71724677	11	LRTOMT (-66900), IL18BP (+14373)
R.2929	71725340	71725590	11	LRTOMT (-65917), IL18BP (+15356)
R.2930	72532555	72533487	11	ATG16L2 (+7668), FCHSD2 (+320285)
R.2931	72975404	72975702	11	P2RY6 (-7694), P2RY2 (+46209)
R.2932	73053649	73054401	11	RELT (-33284), ARHGEF17 (+34691)
R.2933	73357164	73357194	11	PLEKHB1 (-44)
R.2934	75237636	75237935	11	GDPD5 (-838)
R.2935	76155921	76156042	11	C11orf30 (-2001)
R.2936	76381040	76381081	11	LRRC32 (-17)
R.2937	76493489	76493876	11	LRRC32 (-112639), TSKU (-9113)
R.2939	76838749	76839476	11	MYO7A (-197)
R.2940	76849101	76849451	11	MYO7A (+9966), GDPD4 (+149187)
R.2942	77774337	77775044	11	THRSP (-216)
R.2944	82612683	82612766	11	PRCP (-1253), C11orf82 (-44)
R.2945	85397109	85397684	11	CCDC89 (-77)
R.2946	85645743	85646125	11	SYTL2 (-208423), PICALM (+134037)
R.2947	86085932	86086101	11	C11orf73 (+72752), ME3 (+297661)
R.2948	86383761	86383809	11	ME3 (-107)
R.2949	86663341	86663418	11	FZD4 (+3053), PRSS23 (+152098)
R.2950	92702628	92702653	11	MTNR1B (-245)
R.2951	94245224	94245440	11	FUT4 (-31685), MRE11A (-18258)
R.2952	94277826	94278457	11	PIWIL4 (-22332), FUT4 (+1125)
R.2953	94502804	94502879	11	AMOTL1 (+1305), CWC15 (+203934)
R.2954	94965469	94965610	11	SESN3 (-1179)
R.2955	101454683	101454765	11	TRPC6 (-65)
R.2957	102638669	102638778	11	MMP8 (-43039), MMP10 (+12635)
R.2960	107582818	107582884	11	SLN (+7568), ELMOD1 (+120895)
R.2963	111169427	111169473	11	ARHGAP20 (-585999), C11orf92 (+6320)
R.2965	111410935	111411076	11	LAYN (-378)
R.2966	111782016	111782670	11	HSPB2-C11orf52 (-1117), ENSG00000170276 (-623)
R.2967	111847475	111847747	11	DLAT (-47927), DIXDC1 (+39684)
R.2968	113258210	113258277	11	ANKK1 (-269)
R.2969	113258444	113258750	11	ANKK1 (+84)
R.2971	113929164	113929287	11	ZBTB16 (-1089)
R.2972	113947036	113947203	11	NNMT (-181433), ZBTB16 (+16805)
R.2973	114127924	114128517	11	NNMT (-332)
R.2974	114166440	114166636	11	RBM7 (-104214), NNMT (+37985)
R.2977	116969404	116969870	11	SIK3 (-644)
R.2978	117069780	117070485	11	TAGLN (-2456)
R.2980	117747934	117748236	11	FXYD6 (-188)
R.2984	118505096	118505740	11	PHLDB1 (+28263), TREH (+44963)
R.2985	118773555	118774009	11	BCL9L (+7831), CXCR5 (+19307)
R.2986	118781633	118781778	11	BCL9L (-93)
R.2987	119205209	119205373	11	RNF26 (+54)
R.2990	120110812	120110887	11	TMEM136 (-85155), POU2F3 (+3501)

R.2991	120434710	120434979	11	TBCEL (-459985), GRIK4 (+52377)
R.2993	121297557	121297737	11	SORL1 (-25265), SC5D (+134485)
R.2994	121970285	121971092	11	BLID (+16234), SORL1 (+647777)
R.2995	121973013	121973805	11	BLID (+13514), SORL1 (+650497)
R.2996	122051193	122051844	11	UBASH3B (-474864), BLID (-64596)
R.2997	122074011	122074433	11	UBASH3B (-452161), BLID (-87299)
R.2998	123396299	123396593	11	GRAMD1B (-84)
R.2999	123525662	123525669	11	SCN3B (-354)
R.3001	124609941	124610713	11	NRGN (+486)
R.3002	124621829	124622815	11	VSIG2 (-188)
R.3003	124628888	124629752	11	VSIG2 (-7186), ESAM (+2860)
R.3005	125366499	125366590	11	FEZ1 (-339)
R.3008	125932701	125933215	11	CDON (+229)
R.3009	126310409	126311095	11	ST3GAL4 (+35075), KIRREL3 (+559903)
R.3010	128321662	128321880	11	ETS1 (+135666)
R.3011	128457400	128457682	11	ETS1 (-104)
R.3013	128559244	128560240	11	FLI1 (-4148)
R.3014	128560328	128561007	11	FLI1 (-3222)
R.3015	128646953	128647107	11	KCNJ1 (+65399), FLI1 (+83140)
R.3016	128693801	128694679	11	KCNJ1 (+18189), FLI1 (+130350)
R.3017	129183160	129183282	11	ARHGAP32 (-121128), BARX2 (-62614)
R.3018	129685597	129685751	11	TMEM45B (-40)
R.3020	129991445	129992368	11	ST14 (-37550), APLP2 (+52106)
R.3024	130298854	130299333	11	ADAMTS8 (-206)
R.3025	131239703	131240660	11	NTM (-541174), SNX19 (-453778)
R.3027	131780357	131780499	11	NTM (-928)
R.3030	132662455	132662963	11	OPCML (+150857), NTM (+881353)
R.3031	133820837	133821252	11	SPATA19 (-105649), IGSF9B (+5835)
R.3032	133823125	133823862	11	SPATA19 (-108098), IGSF9B (+3386)
R.3037	248296	249286	12	IQSEC3 (+72860), SLC6A12 (+74898)
R.3039	371916	372246	12	SLC6A13 (-79)
R.3047	2045722	2045810	12	CACNA2D4 (-17764), DCP1B (+67911)
R.3050	2944310	2944480	12	NRIP2 (-205)
R.3052	3371110	3371908	12	PRMT8 (-228893), TSPAN9 (+184966)
R.3054	4416614	4417170	12	C12orf5 (-13479), CCND2 (+33954)
R.3055	4488749	4488893	12	FGF23 (+73)
R.3056	4671207	4671597	12	DYRK4 (-27842), RAD51AP1 (+23346)
R.3057	5152546	5152766	12	KCNA5 (-429)
R.3063	6661647	6662581	12	IFFO1 (+3125), GAPDH (+19021)
R.3064	6691577	6691805	12	NOP2 (-14247), CHD4 (+24860)
R.3065	6756751	6757257	12	ACRBP (-378)
R.3068	6959518	6960042	12	USP5 (-1512), CDCA3 (+1450)
R.3075	8833355	8834151	12	MFAP5 (-18345), RIMKLB (-16765)
R.3076	9217510	9217769	12	A2M (+51113), KLRG1 (+75503)
R.3077	10095902	10096112	12	CLEC2A (-11027), CLEC12A (-7908)
R.3078	12418005	12418943	12	LRP6 (+1272), BCL2L14 (+194601)
R.3079	13043502	13043755	12	GPRC5A (-87)
R.3080	13068699	13069409	12	GPRC5A (+25338), GPRC5D (+34264)
R.3081	13080282	13080422	12	GPRC5D (+22966), GPRC5A (+36636)
R.3082	14996272	14996776	12	ART4 (-95)

R.3083	19936477	19936663	12	PDE3A (-585609), AEBP2 (+343962)
R.3085	24717001	24717178	12	SOX5 (-614453), BCAT1 (+338919)
R.3087	25801455	25801621	12	RASSF8 (-310453), IFLTD1 (-95321)
R.3088	26347596	26348117	12	SSPN (-572)
R.3090	33030976	33031216	12	YARS2 (-122260), PKP2 (+18594)
R.3091	41221639	41221855	12	PDZRN4 (-360503), CNTN1 (+135393)
R.3092	42981814	42982649	12	PRICKLE1 (-104435), ADAMTS20 (+963492)
R.3093	45685719	45686426	12	ARID2 (-437375), ANO6 (+76205)
R.3096	47219626	47219737	12	SLC38A4 (+98)
R.3097	47219920	47220092	12	SLC38A4 (-226)
R.3099	48690915	48691036	12	OR10AD1 (-93806), H1FNT (-31787)
R.3103	49688316	49688980	12	TROAP (-28414), PRPH (+1163)
R.3104	49690254	49691064	12	TROAP (-26403), PRPH (+3174)
R.3106	50497732	50497827	12	GPD1 (+178)
R.3107	51610688	51611065	12	POU6F1 (+600)
R.3109	52437299	52437571	12	C12orf44 (-26320), NR4A1 (+20819)
R.3111	52652462	52652742	12	KRT7 (+25704), KRT81 (+32716)
R.3116	52994933	52995295	12	KRT72 (+208)
R.3119	53443057	53443911	12	TENC1 (+751)
R.3120	53591398	53591756	12	ITGB7 (+9514), ZNF740 (+17093)
R.3121	53612551	53612734	12	ITGB7 (-11552), RARG (+13393)
R.3122	53886067	53886622	12	MAP3K12 (+1305), PCBP2 (+40432)
R.3123	54071090	54071672	12	ATP5G2 (-1272)
R.3127	54133447	54133653	12	HOXC13 (-198985), CALCOCO1 (-12326)
R.3135	54785003	54785055	12	ZNF385A (+53)
R.3140	56096884	56097787	12	ITGA7 (+4149), METTL7B (+22006)
R.3141	56105991	56106698	12	ITGA7 (-4860), BLOC1S1 (-3476)
R.3142	56113808	56114269	12	RDH5 (-143)
R.3144	56359210	56360065	12	CDK2 (-915), PMEL (+168)
R.3145	56414442	56415271	12	RPS26 (-20780), IKZF4 (+13414)
R.3146	56694490	56694667	12	CS (-403)
R.3147	57569262	57569787	12	NXPH4 (-41053), LRP1 (+47243)
R.3151	57915613	57915636	12	DDIT3 (-1325), MBD6 (-902)
R.3152	58002603	58003385	12	ARHGEF25 (-969)
R.3153	58003774	58004448	12	ARHGEF25 (+148)
R.3154	58011837	58011875	12	SLC26A10 (-1837)
R.3158	58119915	58120635	12	AGAP2 (+11754), OS9 (+32371)
R.3159	58129407	58130183	12	AGAP2 (+2234), OS9 (+41891)
R.3160	58130410	58131345	12	AGAP2 (+1151), OS9 (+42974)
R.3161	58131681	58132558	12	AGAP2 (-91)
R.3163	65014995	65015547	12	RASSF3 (+10978), GNS (+137956)
R.3164	65671664	65672052	12	MSRB3 (-565)
R.3165	67072769	67073062	12	GRIP1 (-163)
R.3168	71551503	71552457	12	LGR5 (-281570), PTPRR (-237357)
R.3169	72666264	72666976	12	TRHDE (+157)
R.3172	87232511	87232821	12	MGAT4C (-582621)
R.3174	91572142	91572276	12	LUM (-66601), DCN (+4691)
R.3176	94580426	94580931	12	PLXNC1 (+38180), CCDC41 (+273049)
R.3178	99548329	99548950	12	APAF1 (+509721), ANKS1B (+829375)
R.3180	107974296	107974554	12	PWP1 (-105084), BTBD11 (+262235)

R.3184	109569084	109569180	12	ACACB (+14732), FOXN4 (+177893)
R.3185	110149962	110150261	12	MMAB (-138433), TRPV4 (+102584)
R.3186	111358470	111358639	12	MYL2 (-174)
R.3188	111473641	111474016	12	CUX2 (+2001), FAM109A (+333096)
R.3191	111840792	111841024	12	SH2B3 (-2844)
R.3192	112074044	112074158	12	ATXN2 (-36621), BRAP (+49689)
R.3193	112194269	112195089	12	ALDH2 (-10047), ENSG00000257767 (+2985)
R.3195	113013363	113013494	12	RPH3A (-216031), PTPN11 (+156711)
R.3196	113916473	113916664	12	LHX5 (-6692), RBM19 (+487542)
R.3197	114232702	114232905	12	LHX5 (-322927), RBM19 (+171307)
R.3198	114234083	114234200	12	LHX5 (-324265), RBM19 (+169969)
R.3219	116586872	116587387	12	MED13L (+128013)
R.3220	116756285	116756948	12	MAP1LC3B2 (-240569), MED13L (-41474)
R.3222	117036972	117037408	12	MAP1LC3B2 (+40004), C12orf49 (+138676)
R.3223	117626777	117627450	12	TESC (-89840), FBXO21 (+1147)
R.3224	118500366	118501232	12	WSB2 (-1804)
R.3225	118588556	118589026	12	PEBP1 (+15128), TAOK3 (+221959)
R.3226	120241287	120242156	12	CIT (+73373), PRKAB1 (+136164)
R.3227	120242330	120242513	12	CIT (+72673), PRKAB1 (+136864)
R.3228	120538681	120539137	12	RAB35 (+15734), CCDC64 (+111236)
R.3229	121416315	121416796	12	HNF1A (+210)
R.3230	121973425	121974305	12	KDM2B (+45024), RNF34 (+135943)
R.3231	122235169	122235560	12	RHOF (-3197)
R.3233	122296950	122297408	12	HPD (-414)
R.3234	122355599	122356056	12	WDR66 (+60)
R.3238	123258347	123258742	12	CCDC62 (-329)
R.3241	123757018	123757413	12	CDK2AP1 (-529)
R.3242	123868728	123869502	12	SETD8 (+795)
R.3243	124196011	124196328	12	ATP6V0A2 (-695)
R.3245	124773668	124774078	12	NCOR2 (+205925), ZNF664 (+316203)
R.3247	124812999	124813340	12	NCOR2 (+166628), ZNF664 (+355500)
R.3248	124864528	124865130	12	NCOR2 (+114969), ZNF664 (+407159)
R.3249	124907105	124907367	12	NCOR2 (+72562), ZNF664 (+449566)
R.3250	124908112	124908617	12	NCOR2 (+71433), ZNF664 (+450695)
R.3252	124938573	124939526	12	NCOR2 (+40748), ZNF664 (+481380)
R.3255	125002007	125002940	12	NCOR2 (-22676), SCARB1 (+345919)
R.3256	125003064	125003558	12	NCOR2 (-23513), SCARB1 (+345082)
R.3257	125028295	125028339	12	NCOR2 (-48519), SCARB1 (+320076)
R.3258	125033803	125034283	12	NCOR2 (-54245), SCARB1 (+314350)
R.3259	125139714	125140249	12	NCOR2 (-160184), SCARB1 (+208411)
R.3262	126676048	126676790	12	TMEM132B (+865257)
R.3263	127211262	127212034	12	NONE
R.3265	129127968	129128018	12	SLC15A4 (+180535), TMEM132C (+376045)
R.3266	129298690	129299332	12	SLC15A4 (+9517), TMEM132C (+547063)
R.3267	129309207	129309376	12	SLC15A4 (-764)
R.3270	130502003	130502468	12	FZD10 (-144768), TMEM132D (-114025)
R.3272	130647213	130647580	12	FZD10 (+393)
R.3278	132690643	132690782	12	GALNT9 (-140)
R.3282	132886849	132887175	12	GALNT9 (-196439), MUC8 (+163714)
R.3285	133007675	133008035	12	GALNT9 (-317282), MUC8 (+42871)

R.3294	133414255	133414571	12	GOLGA3 (-9125), CHFR (+49781)
R.3297	20751679	20751945	13	GJA3 (-16624), GJB2 (+12107)
R.3299	21287237	21288062	13	IL17D (+10168), N6AMT2 (+60438)
R.3304	29157934	29158364	13	FLT1 (-88917), POMP (-75092)
R.3305	31019673	31020046	13	KATNAL1 (-138239), HMGB1 (+171874)
R.3306	31306018	31306303	13	ALOX5AP (-3484)
R.3307	31479935	31480273	13	MEDAG (-224)
R.3308	31480942	31481184	13	MEDAG (+735)
R.3309	31506685	31507139	13	MEDAG (+26584), HSPH1 (+229152)
R.3310	34184932	34185311	13	STARD13 (-325230), RFC3 (-207064)
R.3312	37004721	37004747	13	CCNA1 (-1761)
R.3313	37494744	37495632	13	SMAD9 (-779)
R.3315	44359415	44359836	13	ENOX1 (+1418), DNAJC15 (+762287)
R.3316	44595496	44595905	13	ENOX1 (-234657), SMIM2 (+139692)
R.3318	46425904	46426263	13	SIAH3 (-213)
R.3319	47471264	47471705	13	HTR2A (-1312)
R.3321	51417846	51417929	13	RNASEH2B (-65926), DLEU1 (+761581)
R.3324	53775035	53775410	13	OLFM4 (+172329)
R.3326	73356430	73356572	13	DIS3 (-430), PIBF1 (+304)
R.3327	76430089	76430722	13	LMO7 (+95609)
R.3328	77461279	77461426	13	KCTD12 (-828)
R.3329	80910763	80911692	13	SPRY2 (+2566), NDFIP2 (+855941)
R.3332	95253641	95253676	13	GPR180 (-498)
R.3333	95826930	95827029	13	SOX21 (-462591), ABCC4 (+126703)
R.3334	96744126	96744289	13	HS6ST3 (+1115), OXGR1 (+902396)
R.3337	99135543	99135625	13	RNF113B (-306065), STK24 (+38668)
R.3338	99405102	99405124	13	SLC15A1 (-205)
R.3339	99629970	99630583	13	DOCK9 (-33)
R.3342	100578585	100578831	13	ZIC5 (+45455), CLYBL (+319785)
R.3344	102104707	102105440	13	ITGBL1 (+108)
R.3348	107568171	107568872	13	ARGLU1 (-348010), FAM155A (+950561)
R.3349	107569368	107569408	13	ARGLU1 (-348876), FAM155A (+949695)
R.3350	108866993	108867154	13	ABHD13 (-3653), LIG4 (+56)
R.3351	110319562	110319607	13	IRS2 (+119330)
R.3352	110521956	110522265	13	IRS2 (-83196), COL4A1 (+437385)
R.3353	110874267	110874326	13	IRS2 (-435382), COL4A1 (+85199)
R.3354	110885015	110885926	13	IRS2 (-446556), COL4A1 (+74025)
R.3355	110959650	110960177	13	COL4A1 (-418), COL4A2 (+300)
R.3356	110961404	110961824	13	COL4A1 (-2118), COL4A2 (+2000)
R.3359	111107075	111107905	13	RAB20 (+106590), COL4A2 (+147876)
R.3360	111281037	111281479	13	CARKD (+13250), CARS2 (+77246)
R.3362	111297400	111297551	13	CARKD (+29468), CARS2 (+61028)
R.3365	111522755	111522985	13	ARHGEF7 (-244754), ING1 (+155541)
R.3366	111837676	111838481	13	TEX29 (-134936), ARHGEF7 (+70455)
R.3367	111839104	111839335	13	TEX29 (-133795), ARHGEF7 (+71596)
R.3369	111935412	111936059	13	TEX29 (-37279), ARHGEF7 (+168112)
R.3370	112063695	112064147	13	SOX1 (-657992), TEX29 (+90906)
R.3371	112191122	112191272	13	SOX1 (-530716), TEX29 (+218182)
R.3372	112609986	112610059	13	SOX1 (-111890), TEX29 (+637008)
R.3373	112709538	112710428	13	SOX1 (-11930), TEX29 (+736968)

R.3374	112722333	112723034	13	SOX1 (+771)
R.3376	112979998	112980703	13	SPACA7 (-50282), SOX1 (+258438)
R.3381	113365389	113366074	13	MCF2L (-257803), ATP11A (+21089)
R.3384	113496400	113497324	13	MCF2L (-126673), ATP11A (+152219)
R.3386	113540400	113540631	13	MCF2L (-83019), ATP11A (+195873)
R.3391	113655622	113656424	13	F7 (-104098), MCF2L (+32488)
R.3393	113689422	113689955	13	F7 (-70432), MCF2L (+66154)
R.3398	113776873	113777160	13	F10 (-115)
R.3400	114067943	114068181	13	GRTP1 (-49621), ADPRHL1 (+39777)
R.3402	114149099	114150035	13	TFDP1 (-89489), TMCO3 (+4257)
R.3403	114187104	114188051	13	TFDP1 (-51478), TMCO3 (+42268)
R.3404	114200981	114201796	13	TFDP1 (-37667), TMCO3 (+56079)
R.3405	114202067	114202683	13	TFDP1 (-36681), TMCO3 (+57065)
R.3409	114792210	114793125	13	GAS6 (-225628), RASA3 (+105418)
R.3410	114797349	114797865	13	GAS6 (-230567), RASA3 (+100479)
R.3412	114812177	114812302	13	GAS6 (-245200), RASA3 (+85846)
R.3418	20903410	20903611	14	TEP1 (-21931), OSGEP (+19753)
R.3420	21466565	21467316	14	SLC39A2 (-508)
R.3421	21494071	21494161	14	TPPP2 (-4269), NDRG2 (-993)
R.3423	23305780	23305957	14	MMP14 (+103)
R.3424	23351487	23352029	14	REM2 (-616)
R.3425	23447078	23447792	14	HAUS4 (-21072), ENSG00000259132 (+4032)
R.3426	23525003	23525720	14	CDH24 (+1385), PSMB11 (+13986)
R.3427	23573850	23574656	14	ACIN1 (-9430), CEBPE (+14572)
R.3428	23623480	23623935	14	CEBPE (-34883), SLC7A8 (+29141)
R.3429	23707002	23707320	14	HOMEZ (+48165), C14orf164 (+52636)
R.3430	23835595	23836012	14	EFS (-962)
R.3432	23904930	23905273	14	MYH7 (-175)
R.3433	24540452	24541377	14	CPNE6 (+869)
R.3434	24641021	24641183	14	REC8 (+40)
R.3435	24641194	24641852	14	REC8 (+461)
R.3439	24867164	24868082	14	NYNRIN (-369)
R.3441	31677445	31677716	14	HECTD1 (-892)
R.3443	36987722	36988446	14	MBIP (-198202), NKX2-1 (+1349)
R.3449	38080446	38080904	14	SSTR1 (-596529), FOXA1 (-16436)
R.3450	45603067	45603518	14	FANCM (-1857), FKBP3 (+1229)
R.3451	51290136	51290662	14	SAV1 (-155350), NIN (+7440)
R.3453	53419080	53419701	14	FERMT2 (-1576)
R.3454	54430529	54430831	14	CDKN3 (-432993), BMP4 (-7151)
R.3456	59932289	59933130	14	GPR135 (-650)
R.3461	61748243	61748440	14	TMEM30B (+216)
R.3463	65005951	65006071	14	HSPA2 (-1175)
R.3464	65289728	65289994	14	SPTB (+5)
R.3465	68830813	68831210	14	ZFP36L1 (+428945), RAD51B (+544487)
R.3468	70826997	70827336	14	COX16 (-719)
R.3471	74185294	74185993	14	PNMA1 (-4516)
R.3472	75447512	75447888	14	PGF (-25213), EIF2B2 (-21914)
R.3474	75893680	75894209	14	JDP2 (-4892)
R.3475	75897342	75897841	14	JDP2 (-1245)
R.3476	75936582	75936789	14	BATF (-52082), JDP2 (+37849)

R.3477	76445988	76446681	14	TGFB3 (+1199), TTLL5 (+318714)
R.3478	76447327	76447949	14	IFT43 (-4487), TGFB3 (-104)
R.3479	77041210	77041277	14	VASH1 (-187288), ESRRB (+167363)
R.3480	77737495	77737771	14	NGB (+22)
R.3481	77767644	77767800	14	NGB (-30067), GSTZ1 (-19505)
R.3485	91579859	91580142	14	C14orf159 (-713)
R.3489	93419016	93419554	14	CHGA (+29860), ITPK1 (+162863)
R.3490	94423192	94424156	14	PRIMA1 (-168847), ASB2 (+19463)
R.3491	94461913	94462337	14	OTUB2 (-30550), ASB2 (-18988)
R.3497	95403054	95403143	14	GSC (-166537), DICER1 (+221248)
R.3498	95693784	95693981	14	DICER1 (-69536), CLMN (+92360)
R.3500	97924509	97924890	14	VRK1 (+661059)
R.3501	98444417	98444533	14	NONE
R.3505	99786193	99786833	14	BCL11B (-48948), SETD3 (+160703)
R.3509	101157915	101158251	14	BEGAIN (-123676), DLK1 (-35081)
R.3510	101175123	101175970	14	BEGAIN (-141140), DLK1 (-17617)
R.3511	101192852	101192913	14	DLK1 (-281)
R.3514	101618194	101618406	14	DIO3 (-409388), ENSG00000269375 (+259035)
R.3516	102026258	102026294	14	DIO3 (-1412)
R.3517	102027514	102027797	14	DIO3 (-32)
R.3518	102226416	102226547	14	PPP2R5C (-1653)
R.3520	103415882	103416389	14	AMN (+27143), CDC42BPB (+107663)
R.3522	103593088	103593520	14	EIF5 (-207035), TNFAIP2 (+3506)
R.3525	104178670	104179160	14	ZFYVE21 (-3152), XRCC3 (-474)
R.3526	104190678	104190829	14	ZFYVE21 (+8687), PPP1R13B (+123173)
R.3530	104668772	104668845	14	C14orf144 (-41732), KIF26A (+63749)
R.3531	104940229	104940465	14	C14orf180 (-105674), C14orf144 (+229806)
R.3532	104979372	104979611	14	C14orf180 (-66529), C14orf144 (+268951)
R.3534	105045877	105046247	14	C14orf180 (+41)
R.3535	105052560	105053550	14	C14orf180 (+7034), TMEM179 (+18929)
R.3537	105147537	105147781	14	TMEM179 (-75675), INF2 (-8315)
R.3538	105154557	105154858	14	INF2 (-1266)
R.3539	105236538	105236654	14	SIVA1 (+17159), AKT1 (+23865)
R.3540	105251188	105251610	14	AKT1 (+9062), SIVA1 (+31962)
R.3541	105265453	105265909	14	ZBTB42 (-1569)
R.3543	105398575	105399321	14	PLD4 (+7788), AHNAK2 (+45746)
R.3548	105747540	105748448	14	PACS2 (-33087), BTBD6 (+33083)
R.3549	105779910	105780137	14	PACS2 (-1057)
R.3550	105789630	105790276	14	TEX22 (-74967), BRF1 (-8027)
R.3556	23085026	23085646	15	NIPA2 (-50928), NIPA1 (+1100)
R.3557	25919674	25920224	15	UBE3A (-266154), ATP10A (+188406)
R.3558	25962719	25963457	15	UBE3A (-309293), ATP10A (+145267)
R.3560	26047382	26047653	15	UBE3A (-393723), ATP10A (+60837)
R.3564	27212911	27213362	15	GABRG3 (-3292)
R.3565	29210444	29210759	15	APBA2 (+79482), NDNL2 (+351431)
R.3567	31516111	31516481	15	KLF13 (-102762), TRPM1 (-62820)
R.3568	32933661	32934185	15	SCG5 (+46)
R.3573	37388127	37388828	15	MEIS2 (+3608)
R.3574	37389384	37389951	15	MEIS2 (+2418)
R.3575	39871876	39872186	15	THBS1 (-1263)

R.3576	40583227	40583422	15	PLCB2 (+16798), PAK6 (+51704)
R.3578	40633124	40633202	15	PLCB2 (-33040), DISP2 (-17273)
R.3579	40674878	40674910	15	KNSTRN (-28)
R.3580	41061384	41061527	15	C15orf62 (-703)
R.3581	42173869	42174823	15	SPTBN5 (+11929), PLA2G4B (+44373)
R.3585	42749336	42749885	15	ZNF106 (+119)
R.3588	43809486	43809865	15	MAP1A (-147)
R.3590	45671148	45671708	15	GATM (-437)
R.3591	48935514	48936335	15	FBN1 (+2121), DUT (+312305)
R.3592	51387651	51387736	15	AP4E1 (+186824), CYP19A1 (+243113)
R.3593	51973661	51973920	15	SCG3 (+241)
R.3594	52030164	52030641	15	LYSMD2 (-69)
R.3596	57026014	57026064	15	ZNF280D (-252)
R.3597	59156878	59157579	15	SLTM (+68623), FAM63B (+93716)
R.3598	60285234	60285744	15	BNIP2 (-303756), FOXB1 (-10932)
R.3599	63333476	63333846	15	TPM1 (-1223)
R.3600	63889093	63889711	15	FBXL22 (-150)
R.3601	65068554	65068885	15	RBPMS2 (-934)
R.3602	65204370	65204428	15	ANKDD1A (+298)
R.3605	65503447	65503889	15	CILP (+158)
R.3608	66999484	67000045	15	SMAD3 (-358418), SMAD6 (+5199)
R.3609	67417899	67418428	15	SMAD3 (+59981), AAGAB (+128910)
R.3610	67457967	67458665	15	AAGAB (+88758), SMAD3 (+100133)
R.3611	69744390	69744684	15	RPLP1 (-586)
R.3612	70877866	70878049	15	TLE3 (-487443), UACA (+177974)
R.3613	70994435	70995014	15	TLE3 (-604210), UACA (+61207)
R.3614	72075835	72075962	15	SENP8 (-333293), THSD4 (+686608)
R.3615	72563412	72564263	15	PKM (-40154), PARP6 (+1124)
R.3617	74218418	74218921	15	LOXL1 (-129)
R.3618	74228839	74229811	15	LOXL1 (+10526), STOML1 (+55364)
R.3621	75499862	75500859	15	GOLGA6C (-50579), PPCDC (+184465)
R.3622	75500943	75501277	15	GOLGA6C (-49830), PPCDC (+185214)
R.3624	75918700	75919561	15	SNUPN (-1102)
R.3626	76005861	76006143	15	CSPG4 (-813)
R.3628	77287338	77287656	15	PSTPIP1 (+71)
R.3629	78110857	78111196	15	LINGO1 (-186195), TBC1D2B (+258967)
R.3630	78286905	78287690	15	LINGO1 (-362466), TBC1D2B (+82696)
R.3632	79164541	79164806	15	MORF4L1 (-482)
R.3635	79722776	79723655	15	KIAA1024 (-1642)
R.3636	81426610	81426820	15	MESDC2 (-144496), IL16 (-91026)
R.3639	85359250	85360217	15	ALPK3 (-177)
R.3640	85426911	85427743	15	SLC28A1 (-586)
R.3641	86315115	86315211	15	KLHL25 (+23098), AKAP13 (+391203)
R.3644	89920824	89920918	15	POLG (-42793), RHCG (+118973)
R.3645	90456387	90456924	15	C15orf38-AP3S2 (-542), C15orf38 (-468)
R.3647	90727783	90727995	15	IDH2 (-82153), SEMA4B (-16662)
R.3648	91447195	91447540	15	MAN2A2 (-52)
R.3649	91500193	91500893	15	RCCD1 (+2443), PRC1 (+37182)
R.3651	93277307	93277793	15	CHD2 (-165508), FAM174B (-78362)
R.3652	93361889	93362731	15	FAM174B (-163122), CHD2 (-80748)

R.3655	93613983	93614970	15	RGMA (+2615), CHD2 (+171419)
R.3661	96875210	96875656	15	NR2F2 (+1487)
R.3669	96904176	96904960	15	NR2F2 (+30622)
R.3670	96906354	96907209	15	NR2F2 (+32836)
R.3671	96909322	96910011	15	NR2F2 (+35721)
R.3672	98064827	98065822	15	ARRDC4 (-438603)
R.3673	99194474	99195143	15	IGF1R (+2609), PGPEP1L (+353982)
R.3675	99789622	99789855	15	MEF2A (-316166), SYNM (+144453)
R.3676	99978986	99979389	15	MEF2A (-126717), SYNM (+333902)
R.3677	100272053	100272751	15	LYSMD4 (+1224), MEF2A (+166497)
R.3679	101389974	101390259	15	ALDH1A3 (-29464), ASB7 (+247378)
R.3680	101513798	101514474	15	LRRK1 (+54716), CHSY1 (+278001)
R.3681	101661445	101661580	15	CHSY1 (+130624), LRRK1 (+202093)
R.3682	101728611	101729526	15	CHSY1 (+63068), LRRK1 (+269649)
R.3684	102157488	102157726	15	PCSK6 (-127734), TM2D3 (+34963)
R.3686	331727	332515	16	PDIA2 (-1031)
R.3689	615709	616220	16	PIGQ (-4053)
R.3691	834043	834399	16	CHTF18 (-4401), MSLNL (-1295)
R.3692	867542	867594	16	GNG13 (-16835), LMF1 (+153431)
R.3696	1026944	1027604	16	SOX8 (-4534)
R.3698	1032491	1033408	16	SSTR5 (-95831), SOX8 (+1142)
R.3699	1041053	1041686	16	SSTR5 (-87411), SOX8 (+9562)
R.3701	1069368	1069760	16	SSTR5 (-59217), SOX8 (+37756)
R.3702	1080388	1080808	16	SSTR5 (-48183), SOX8 (+48790)
R.3703	1121050	1122047	16	SSTR5 (-7232), SOX8 (+89741)
R.3704	1128689	1128865	16	SSTR5 (-4)
R.3705	1131266	1131466	16	SSTR5 (+2585), C1QTNF8 (+14878)
R.3707	1138344	1138697	16	C1QTNF8 (+7723), SSTR5 (+9740)
R.3708	1145964	1146545	16	C1QTNF8 (-11)
R.3709	1147214	1148056	16	C1QTNF8 (-1391)
R.3711	1209990	1210829	16	CACNA1H (+7169), TPSG1 (+64847)
R.3712	1310584	1310905	16	UBE2I (-48136), TPSD1 (+4611)
R.3713	1480790	1480992	16	C16orf91 (-1546)
R.3716	1583883	1583984	16	TMEM204 (+360)
R.3719	1600304	1600969	16	TMEM204 (+17063), IFT140 (+61474)
R.3721	1843697	1844152	16	IGFALS (-192)
R.3722	2004686	2005062	16	NDUFB10 (-4635), RPL3L (-147)
R.3723	2021941	2022090	16	TBL3 (-22)
R.3725	2030509	2030892	16	GFER (-3513), NOXO1 (+483)
R.3733	2867051	2867446	16	PRSS21 (+21)
R.3734	3016717	3017593	16	PAQR4 (-2091)
R.3735	3062349	3062426	16	CLDN9 (-69)
R.3736	3162397	3163017	16	ZNF205 (+146)
R.3740	3843012	3843415	16	TRAP1 (-75616), CREBBP (+87513)
R.3741	3998975	3999569	16	CREBBP (-68545), ADCY9 (+166914)
R.3742	4000474	4000652	16	CREBBP (-69836), ADCY9 (+165623)
R.3744	4056360	4056879	16	CREBBP (-125893), ADCY9 (+109566)
R.3745	4102293	4103225	16	CREBBP (-172032), ADCY9 (+63427)
R.3746	4163819	4164087	16	CREBBP (-233226), ADCY9 (+2233)
R.3747	4164891	4165515	16	ADCY9 (+983)

R.3750	4387370	4387567	16	PAM16 (+13904), GLIS2 (+22707)
R.3751	4421445	4421603	16	VASN (-325)
R.3752	4426265	4426960	16	VASN (+4764), CORO7-PAM16 (+39975)
R.3753	4431502	4432164	16	VASN (+9984), CORO7-PAM16 (+34755)
R.3754	4587038	4587862	16	MGRN1 (-87341), CDIP1 (-22340)
R.3755	4665298	4665704	16	CDIP1 (-100391), MGRN1 (-9290)
R.3758	8619531	8619943	16	TMEM114 (+2489)
R.3760	10912331	10912718	16	TVP23A (+117)
R.3763	11876111	11876426	16	ZC3H7A (+139)
R.3764	14397688	14397766	16	MKL2 (+224582), PARN (+326397)
R.3766	15237722	15238525	16	ENSG00000261130 (-251487), RRN3 (-49950)
R.3767	15239600	15240413	16	ENSG00000261130 (-249604), RRN3 (-51833)
R.3768	15818255	15818816	16	NDE1 (+81412), MYH11 (+132332)
R.3769	21171067	21171239	16	DNAH3 (-391)
R.3770	22103596	22104151	16	VWA3A (+11)
R.3771	22825843	22826243	16	HS3ST2 (+545)
R.3772	23724428	23724774	16	ERN2 (+220)
R.3773	27781069	27781794	16	KIAA0556 (+219959), GSG1L (+293398)
R.3774	27791167	27791248	16	KIAA0556 (+229735), GSG1L (+283622)
R.3775	28270238	28270754	16	XPO6 (-47306), SBK1 (-33344)
R.3777	28550171	28550637	16	NUPR1 (-75)
R.3778	28835558	28835698	16	ATXN2L (+1214), TUFM (+22101)
R.3781	29197753	29198393	16	LAT (+201926), NPIPB11 (+217277)
R.3782	29229650	29230149	16	NPIPB11 (+185450), LAT (+233753)
R.3783	29272727	29273115	16	NPIPB11 (+142429), LAT (+276774)
R.3787	29706151	29706381	16	QPRT (+15908), C16orf54 (+51061)
R.3789	29823868	29824599	16	PAGR1 (-3051), PRRT2 (+722)
R.3792	30124293	30124904	16	GDPD3 (+578)
R.3794	30485597	30485810	16	ITGAL (+1725), ZNF768 (+52206)
R.3795	30905861	30906273	16	CTF1 (-1861), BCL7C (+214)
R.3796	30907246	30907679	16	BCL7C (-1182), CTF1 (-465)
R.3798	31159623	31159920	16	PRSS8 (-12689), PRSS36 (+1643)
R.3801	31482618	31483137	16	TGFB1I1 (-502)
R.3802	31484199	31484618	16	SLC5A2 (-10030), TGFB1I1 (+1029)
R.3805	49686567	49686662	16	CBLN1 (-370873), ZNF423 (+170035)
R.3806	49732224	49733170	16	CBLN1 (-416955), ZNF423 (+123953)
R.3807	50003431	50003613	16	ZNF423 (-146872), CNEP1R1 (-54799)
R.3809	50583273	50583729	16	NKD1 (+1260), SNX20 (+131762)
R.3811	50745944	50746063	16	CYLD (-30667), NOD2 (+14954)
R.3813	54227790	54228582	16	IRX3 (+92489), FTO (+490311)
R.3815	54324187	54324358	16	IRX3 (-3598)
R.3824	55512806	55512868	16	MMP2 (-46)
R.3826	56456455	56457177	16	AMFR (+2634), GNAO1 (+230928)
R.3827	56622951	56623111	16	MT3 (-249)
R.3830	57406044	57406955	16	CX3CL1 (+130)
R.3831	57672401	57673258	16	GPR97 (-29269), GPR56 (+10411)
R.3832	58061220	58061473	16	MMP15 (+1877), C16orf80 (+102007)
R.3833	58718915	58718971	16	SLC38A7 (-316)
R.3834	58768477	58769104	16	GOT2 (-530)
R.3835	62067937	62068673	16	CDH8 (+2434)

R.3836	65104932	65105714	16	CDH11 (+50778)
R.3837	66399929	66400599	16	CDH5 (-269)
R.3838	66584228	66585210	16	CKLF (-1751), TK2 (-404)
R.3840	66959729	66959833	16	RRAD (-234)
R.3841	66969500	66969735	16	FAM96B (-1315), CES2 (+1255)
R.3842	67218787	67219218	16	EXOC3L1 (+5104), NOL3 (+11098)
R.3843	67231928	67232557	16	ELMO3 (-771)
R.3844	67233432	67233983	16	ELMO3 (+694)
R.3845	67428155	67428324	16	TPPP3 (-2384)
R.3846	67682005	67682810	16	RLTPR (+3586), ACD (+12238)
R.3847	67686832	67687754	16	ACD (+7353), RLTPR (+8471)
R.3848	67918484	67918965	16	NRN1L (+17)
R.3849	67977865	67978450	16	LCAT (-124)
R.3850	67997858	67998030	16	LCAT (-19910), SLC12A4 (+4653)
R.3851	67999966	68000829	16	LCAT (-22364), SLC12A4 (+2199)
R.3852	68003410	68003907	16	SLC12A4 (-1062)
R.3854	69958189	69958964	16	CLEC18A (-26506), WWP2 (+162368)
R.3855	69969137	69969618	16	CLEC18A (-15705), WWP2 (+173169)
R.3857	70680104	70680817	16	MTSS1L (+39508), IL34 (+66663)
R.3858	70688052	70688171	16	MTSS1L (+31857), IL34 (+74314)
R.3861	70720149	70720817	16	MTSS1L (-514)
R.3862	70759914	70760142	16	MTSS1L (-40059), VAC14 (+75036)
R.3863	71264290	71264579	16	HYDIN (+157)
R.3866	73125573	73126120	16	ZFHX3 (-43573)
R.3867	73205994	73206624	16	ZFHX3 (-124035)
R.3868	75271872	75272754	16	CTRB1 (+19415), BCAR1 (+27592)
R.3869	75273344	75273486	16	CTRB1 (+20517), BCAR1 (+26490)
R.3870	75282051	75282783	16	BCAR1 (+17488), CTRB1 (+29519)
R.3871	75301362	75301973	16	BCAR1 (-1763)
R.3872	75528459	75528920	16	CHST6 (+236)
R.3873	75568999	75569459	16	CHST5 (-161)
R.3874	78539954	78540172	16	WWOX (+406753)
R.3876	81271937	81272429	16	BCMO1 (+130)
R.3877	81520178	81520589	16	PLCG2 (-292479), CMIP (+41609)
R.3878	81678804	81678968	16	PLCG2 (-133977), CMIP (+200111)
R.3881	84346722	84346930	16	ATP2C2 (-55307), WFDC1 (+18574)
R.3883	84560555	84560950	16	TLDC1 (-22389), COTL1 (+90930)
R.3884	84828829	84829007	16	CRISPLD2 (-24672), USP10 (+95334)
R.3886	85062881	85063742	16	KIAA0513 (-33506), ZDHHC7 (-18204)
R.3888	85123342	85123778	16	GSE1 (-523262), KIAA0513 (+26742)
R.3890	85157762	85158047	16	GSE1 (-488917), KIAA0513 (+61087)
R.3891	85196116	85196491	16	GSE1 (-450518), KIAA0513 (+99486)
R.3894	85214676	85215140	16	GSE1 (-431914), KIAA0513 (+118090)
R.3895	85253980	85254209	16	GSE1 (-392727), KIAA0513 (+157277)
R.3896	85470485	85470674	16	GSE1 (-176242), KIAA0513 (+373762)
R.3897	85478769	85479301	16	GSE1 (-167787), KIAA0513 (+382217)
R.3899	85607562	85608556	16	GSE1 (-38763), KIAA0513 (+511241)
R.3900	85608659	85608934	16	GSE1 (-38025), KIAA0513 (+511979)
R.3902	85619974	85620872	16	GSE1 (-26399), KIAA0513 (+523605)
R.3904	85684468	85684853	16	GSE1 (+37839), GINS2 (+37944)

R.3906	85845716	85846184	16	IRF8 (-86459), COX4I1 (+12651)
R.3908	85878940	85879145	16	IRF8 (-53366), COX4I1 (+45744)
R.3914	86591940	86592652	16	MTHFSD (-3480)
R.3918	86714765	86715554	16	FOXL1 (+103045), FBXO31 (+702203)
R.3922	86965114	86965605	16	FOXL1 (+353245), FBXO31 (+452003)
R.3925	87158927	87159274	16	FBXO31 (+258262), FOXL1 (+546986)
R.3927	87574693	87575344	16	JPH3 (-61492), ZCCHC14 (-49368)
R.3928	87730147	87730600	16	KLHDC4 (+69181), JPH3 (+93863)
R.3930	88096564	88096995	16	ZNF469 (-397099), BANP (+93156)
R.3931	88134407	88134848	16	ZNF469 (-359251), BANP (+131004)
R.3933	88186048	88186669	16	ZNF469 (-307520), BANP (+182735)
R.3934	88217830	88218376	16	ZNF469 (-275776), BANP (+214479)
R.3935	88268339	88268771	16	ZNF469 (-225324), BANP (+264931)
R.3938	88539861	88540396	16	ZC3H18 (-96660), ZFPM1 (+20404)
R.3944	88802820	88803803	16	CTU2 (+30441), PIEZO1 (+48307)
R.3947	88895135	88895544	16	APRT (-16988), GALNS (+28034)
R.3949	88975259	88976160	16	PABPN1L (-42684), CBFA2T3 (+67902)
R.3955	89387014	89387569	16	ZNF778 (+103174), ANKRD11 (+169677)
R.3957	89445433	89445503	16	ANKRD11 (+111501), ZNF778 (+161350)
R.3959	89689811	89690262	16	SPATA33 (-34173), DPEP1 (+3037)
R.3965	790628	791324	17	GLOD4 (-105395), NXN (+92034)
R.3966	872762	873452	17	GLOD4 (-187526), NXN (+9903)
R.3967	900325	901284	17	TIMM22 (+448)
R.3968	923728	923829	17	TIMM22 (+23422), ABR (+159389)
R.3969	925669	926438	17	TIMM22 (+25697), ABR (+157114)
R.3970	932057	933047	17	TIMM22 (+32195), ABR (+150616)
R.3971	933118	933179	17	TIMM22 (+32792), ABR (+150019)
R.3972	934534	935017	17	TIMM22 (+34419), ABR (+148392)
R.3974	1090291	1090441	17	BHLHA9 (-83487), ABR (-7198)
R.3977	1182702	1183159	17	TUSC5 (-26)
R.3978	1395864	1396001	17	MYO1C (+173)
R.3981	1553415	1554059	17	SCARF1 (-4696), RILP (-366)
R.3982	1617102	1617375	17	TLCD2 (-3507)
R.3985	2324794	2325143	17	MNT (-20557), METTL16 (+90211)
R.3988	2886250	2886453	17	OR1D5 (+80549), RAP1GAP2 (+186620)
R.3989	2951689	2951736	17	OR1D5 (+15188), RAP1GAP2 (+251981)
R.3990	3416252	3416525	17	ASPA (+37093), TRPV3 (+44710)
R.3992	3563641	3563924	17	TAX1BP3 (+8193), CTNS (+24010)
R.3995	3824384	3824497	17	P2RX1 (-4647)
R.3996	3911336	3912065	17	ATP2A3 (-44116), ZZEF1 (+134613)
R.3999	4439394	4439623	17	MYBBP1A (+19172), SPNS2 (+37376)
R.4000	4648566	4649262	17	TM4SF5 (-26273), ZMYND15 (+5100)
R.4006	5018984	5019638	17	USP6 (-422)
R.4007	5026585	5026840	17	ZNF232 (-316)
R.4009	6358415	6358599	17	FAM64A (+10746), ACKR6 (+101307)
R.4010	6658198	6658728	17	XAF1 (-696)
R.4011	6796745	6797708	17	ALOX12 (-102157), TEKT1 (-62147)
R.4012	6899085	6899758	17	ALOX12 (+38)
R.4013	6921465	6922320	17	BCL6B (-4446)
R.4016	7111414	7111551	17	ASGR1 (-28600), ACADVL (-8961)

R.4019	7253189	7253720	17	KCTD11 (-1753)
R.4021	7491548	7492524	17	SOX15 (+1452), MPDU1 (+5071)
R.4023	7832479	7833237	17	CNTROB (-2615), KCNAB3 (-105)
R.4024	8127196	8127373	17	AURKB (-13369), CTC1 (+24077)
R.4025	8218709	8219095	17	ODF4 (-24280), ARHGEF15 (+5301)
R.4026	8295737	8296113	17	ENSG00000263809 (-9420), RNF222 (+5219)
R.4027	8370017	8370548	17	NDEL1 (+31104), MYH10 (+163796)
R.4028	8380278	8380905	17	NDEL1 (+41413), MYH10 (+153487)
R.4029	8382941	8383800	17	NDEL1 (+44192), MYH10 (+150708)
R.4031	8702486	8702896	17	MFSD6L (-24)
R.4036	9019086	9019336	17	NTN1 (+94352), STX8 (+460342)
R.4038	9862752	9862873	17	RCVRN (-53875), GAS7 (+239055)
R.4039	9929698	9929755	17	RCVRN (-120789), GAS7 (+172141)
R.4041	11144311	11145218	17	SHISA6 (+185)
R.4042	11501639	11501663	17	DNAH9 (-97)
R.4043	12453058	12453277	17	MYOCD (-116138), MAP2K4 (+529027)
R.4045	12567873	12568507	17	MYOCD (-1116)
R.4047	15168483	15168658	17	PMP22 (-2665)
R.4048	15244441	15244519	17	TEKT3 (+478)
R.4049	15522876	15523510	17	CDRT1 (-367)
R.4050	15689091	15689957	17	ADORA2B (-158707), TBC1D26 (+53933)
R.4051	16281514	16282410	17	UBB (-2151)
R.4052	16290151	16290349	17	TRPV2 (-28606), UBB (+6137)
R.4054	17109936	17110353	17	PLD6 (-516)
R.4059	18646874	18647524	17	TVP23B (-37109), TRIM16L (+45876)
R.4060	19290690	19290755	17	MFAP4 (-220)
R.4061	19314299	19314618	17	RNF112 (-48)
R.4062	19651653	19652056	17	ALDH3A1 (-3083)
R.4063	19771364	19771813	17	ULK2 (-359)
R.4065	21356245	21356368	17	UBBP4 (-374388), KCNJ12 (+76798)
R.4068	26697281	26697498	17	SARM1 (-1293), VTN (+453)
R.4069	26732120	26732836	17	SLC46A1 (+750)
R.4076	27899465	27899966	17	TP53I13 (+4054), GIT1 (+16851)
R.4078	29886885	29887049	17	RAB11FIP4 (+168325), COPRS (+299389)
R.4079	30347851	30348814	17	LRRC37B (+172)
R.4080	31254544	31254708	17	TMEM98 (-329)
R.4082	32483560	32483711	17	ASIC2 (-863630), CCL2 (-98668)
R.4083	32580869	32581466	17	CCL2 (-1136)
R.4085	33775917	33775963	17	SLFN13 (-84)
R.4086	33817136	33817217	17	SLFN12L (-2301)
R.4087	33822621	33823619	17	SLFN12L (-8244), SLFN14 (+61997)
R.4088	34079987	34080240	17	GAS2L2 (-217)
R.4091	35656660	35657046	17	C17orf78 (-76132), AATF (+350678)
R.4093	37331279	37331531	17	ARL5C (-8087), CACNB1 (+22551)
R.4094	37356328	37356554	17	CACNB1 (-2485), RPL19 (-95)
R.4096	37820515	37821481	17	PNMT (-3413), TCAP (+558)
R.4098	37855819	37856217	17	ERBB2 (-315)
R.4099	38084377	38085230	17	ORMDL3 (-1710)
R.4101	38249266	38249729	17	NR1D1 (+7480), THRA (+30435)
R.4102	38296481	38296698	17	CASC3 (+14)

R.4103	38334161	38334186	17	RAPGEFL1 (-68)
R.4104	38465281	38465393	17	RARA (-109)
R.4105	38472961	38473382	17	RARA (+7726), GJD3 (+46895)
R.4107	38599056	38599366	17	IGFBP4 (-502)
R.4108	38821420	38821487	17	KRT222 (-61)
R.4112	39791734	39792147	17	EIF1 (-53204), KRT17 (-11112)
R.4113	39969264	39969297	17	LEPREL4 (-426), FKBP10 (+98)
R.4115	40274703	40274811	17	KAT2A (-1381), HSPB9 (+1)
R.4119	40805868	40806193	17	TUBG2 (-5292), TUBG1 (+44337)
R.4120	40932199	40932359	17	WNK4 (-417)
R.4121	40932508	40932520	17	WNK4 (-182)
R.4123	41003155	41003399	17	AOC3 (+76)
R.4126	41477271	41477948	17	DHX8 (-83724), ARL4D (+1283)
R.4127	41738893	41739326	17	MEOX1 (+212)
R.4128	41835937	41836423	17	SOST (-24)
R.4131	42247910	42248358	17	ASB16 (+60)
R.4132	42275123	42275551	17	ATXN7L3 (+101)
R.4134	42635778	42636570	17	DBF4B (-149877), FZD2 (+1249)
R.4135	42785825	42785931	17	DBF4B (-173)
R.4137	42992567	42993050	17	GFAP (+53)
R.4138	42994008	42994269	17	GFAP (-1277)
R.4142	43213270	43213629	17	PLCD3 (-3559), ACBD4 (+3483)
R.4144	43318735	43319382	17	FMNL1 (+19903), SPATA32 (+20420)
R.4145	43472435	43472920	17	SPATA32 (-133199), ARHGAP27 (+30319)
R.4147	43506829	43507585	17	ARHGAP27 (-4210)
R.4149	45786313	45786512	17	TBX21 (-24197), TBKBP1 (+13783)
R.4150	45944339	45944464	17	SP2 (-29114), SP6 (-15843)
R.4154	46631910	46632154	17	HOXB3 (-748)
R.4155	46632965	46633726	17	HOXB3 (-2062)
R.4156	46641504	46641863	17	HOXB3 (-10400), HOXB4 (+15789)
R.4157	46648014	46648582	17	HOXB3 (-17014), HOXB4 (+9175)
R.4165	46681316	46681401	17	HOXB6 (+995)
R.4166	46682394	46683047	17	HOXB6 (-367)
R.4167	46697115	46697543	17	HOXB8 (-5028), HOXB9 (+6510)
R.4168	46698881	46699155	17	HOXB8 (-6717), HOXB9 (+4821)
R.4169	47072907	47073436	17	IGF2BP1 (-1602)
R.4172	48194635	48194939	17	PDK2 (+22148), PPP1R9B (+33090)
R.4174	48242743	48243707	17	SGCA (-141)
R.4175	48546193	48546297	17	CHAD (+82)
R.4176	48555514	48555610	17	RSAD1 (-599)
R.4177	48628397	48628573	17	CACNA1G (-10336), SPATA20 (+4035)
R.4178	48912265	48912952	17	WFIKKN2 (-162)
R.4180	49242847	49243257	17	NME1-NME2 (-873)
R.4182	53499747	53500432	17	MMD (-737)
R.4183	53800041	53800372	17	MMD (-300854), TMEM100 (+9275)
R.4184	55663225	55663284	17	ENSG00000166329 (+159418), MSI2 (+329360)
R.4186	56082885	56083757	17	VEZF1 (-17808), SRSF1 (+1386)
R.4188	56401623	56402238	17	MPO (-43635), BZRAP1 (+3514)
R.4190	57642749	57642763	17	DHX40 (-130)
R.4198	59553911	59554746	17	TBX4 (+20522), NACA2 (+114234)

R.4199	60757756	60758287	17	MRC2 (+53260), MARCH10 (+127673)
R.4200	60783168	60783915	17	MRC2 (+78780), MARCH10 (+102153)
R.4201	60885892	60886284	17	MARCH10 (-393)
R.4202	61926188	61926700	17	SMARCD2 (-6144), CSH2 (+24682)
R.4204	62075103	62075324	17	SCN4A (-24936), ICAM2 (+22780)
R.4207	62773704	62774590	17	SMURF2 (-115961), LRRC37A3 (+140756)
R.4208	63533152	63533778	17	AXIN2 (+24300), RGS9 (+399873)
R.4209	66194721	66194978	17	AMZ2 (-48865), KPNA2 (+162774)
R.4210	66287499	66287507	17	SLC16A6 (-246)
R.4211	66374702	66375195	17	SLC16A6 (-87692), WIPI1 (+78613)
R.4212	70536103	70536199	17	SOX9 (+418990), SLC39A11 (+552676)
R.4214	71188803	71189068	17	COG1 (-193)
R.4216	71772827	71772869	17	RPL38 (-426944), SDK2 (-132620)
R.4218	72321276	72321958	17	KIF19 (-734)
R.4219	72442179	72443179	17	CD300A (-19876), GPRC5C (+15627)
R.4221	72667378	72667709	17	CD300E (-47743), CD300LF (+41573)
R.4225	73285714	73285853	17	SLC25A19 (-2029)
R.4226	73498127	73498542	17	CASKIN2 (+13329), KIAA0195 (+45790)
R.4230	73893424	73893634	17	TRIM65 (-445)
R.4232	74070431	74070698	17	ZACN (-4698), SRP68 (-1831), GALR2 (-310)
R.4233	74073527	74074118	17	ZACN (-1440)
R.4234	74133754	74134059	17	EXOC7 (-34080), FOXJ1 (+3473)
R.4235	74381784	74382495	17	SPHK1 (+1409), UBE2O (+67148)
R.4236	74696737	74696872	17	ST6GALNAC1 (-56885), MXRA7 (+10232)
R.4239	75181254	75181836	17	SEPT9 (-95947), SEC14L1 (+96714)
R.4241	75283431	75283979	17	TNRC6C (-717432), SEPT9 (+6213)
R.4242	75315081	75315244	17	TNRC6C (-685974), SEPT9 (+37671)
R.4243	75373219	75373457	17	TNRC6C (-627799), SEPT9 (+95846)
R.4246	75473577	75474070	17	TNRC6C (-527313), SEPT9 (+196332)
R.4247	75880269	75881232	17	TNRC6C (-120386), SEPT9 (+603259)
R.4248	75905364	75905616	17	TNRC6C (-95647), SEPT9 (+627998)
R.4249	76036514	76037364	17	TNRC6C (+35802), TMC6 (+87839)
R.4258	76719591	76719943	17	DNAH17 (-146291), CYTH1 (+58609)
R.4262	76967757	76968215	17	TIMP2 (-46517), LGALS3BP (+8205)
R.4263	76970857	76970933	17	TIMP2 (-49426), LGALS3BP (+5296)
R.4265	77078650	77079031	17	ENGASE (+7814), RBFOX3 (+399722)
R.4266	77086350	77086810	17	ENGASE (+15553), RBFOX3 (+391983)
R.4268	77133777	77134159	17	ENGASE (+62941), RBFOX3 (+344595)
R.4269	77151165	77151243	17	ENGASE (+80177), RBFOX3 (+327359)
R.4270	77766421	77767242	17	CBX8 (+4083), CBX2 (+14901)
R.4272	77893704	77893850	17	CBX4 (-80549), TBC1D16 (+115870)
R.4276	77997198	77997997	17	CBX4 (-184370), TBC1D16 (+12049)
R.4279	78161110	78161706	17	CARD14 (+17597), SGSH (+32791)
R.4281	78194695	78195518	17	SGSH (-908), SLC26A11 (+878)
R.4284	78755379	78755841	17	CHMP6 (-210031), RPTOR (+236542)
R.4285	78800767	78800806	17	CHMP6 (-164854), RPTOR (+281719)
R.4289	78999347	78999895	17	BAIAP2 (-9341), CHMP6 (+33980)
R.4291	79010458	79011352	17	BAIAP2 (+1943), AATK (+128912)
R.4295	79138873	79139625	17	AATK (+568)
R.4296	79228937	79229385	17	C17orf89 (+16122), SLC38A10 (+39944)

R.4297	79251255	79251354	17	SLC38A10 (+17800), C17orf89 (+38266)
R.4299	79265454	79265759	17	SLC38A10 (+3498), C17orf89 (+52568)
R.4304	79377272	79377665	17	ENSG00000171282 (+3929), ACTG1 (+102338)
R.4305	79392689	79393682	17	ENSG00000171282 (+19646), ACTG1 (+86621)
R.4307	79484960	79485709	17	FSCN2 (-10223), ACTG1 (-5528)
R.4310	79869748	79869861	17	PCYT2 (-523)
R.4311	79881066	79882042	17	SIRT7 (-5484), MAFG (+4036)
R.4313	79949717	79950366	17	ASPSCR1 (+14616), STRA13 (+30752)
R.4314	79961522	79961769	17	STRA13 (+19148), ASPSCR1 (+26220)
R.4315	80066134	80066586	17	SLC16A3 (-125203), FASN (-10152)
R.4319	80200634	80201299	17	SLC16A3 (+9404), CSNK1D (+30640)
R.4320	80201957	80202426	17	SLC16A3 (+10629), CSNK1D (+29415)
R.4321	80205093	80205481	17	SLC16A3 (+13724), CSNK1D (+26320)
R.4323	80266884	80267030	17	CSNK1D (-35350), CD7 (+8521)
R.4324	80273290	80274000	17	CSNK1D (-42038), CD7 (+1833)
R.4339	80970159	80970504	17	ZNF750 (-171878), B3GNTL1 (+39354)
R.4341	81014667	81015507	17	METRNL (-22480), B3GNTL1 (-5401)
R.4342	81045495	81045863	17	METRNL (+8112)
R.4343	3219966	3220532	18	MYOM1 (-143)
R.4345	10453624	10454085	18	APCDD1 (-770)
R.4346	11947875	11948154	18	MPPE1 (-38793), IMPA2 (-33412)
R.4347	12287275	12287561	18	TUBB6 (-20640), CIDEA (+33100)
R.4348	14747661	14747928	18	ANKRD30B (-444)
R.4349	19752292	19753208	18	GATA6 (+3346), CTAGE1 (+245128)
R.4350	19757468	19758221	18	GATA6 (+8441), CTAGE1 (+240033)
R.4351	19780587	19780878	18	GATA6 (+31329), CTAGE1 (+217145)
R.4352	20713759	20714332	18	CABLES1 (-1681)
R.4353	20716991	20717693	18	CABLES1 (+1615), TMEM241 (+300527)
R.4354	20735005	20735693	18	CABLES1 (+19622), TMEM241 (+282520)
R.4356	21572622	21572748	18	CABYR (-146257), LAMA3 (+303278)
R.4359	28896580	28897214	18	DSG1 (-1155)
R.4362	43303777	43304079	18	SLC14A1 (-2762)
R.4363	43355414	43355989	18	SIGLEC15 (-49775), SLC14A1 (+49012)
R.4364	43417389	43418215	18	SIGLEC15 (+12325), EPG5 (+129438)
R.4365	43418556	43419102	18	SIGLEC15 (+13352), EPG5 (+128411)
R.4367	44773985	44774923	18	IER3IP1 (-71709), SKOR2 (+1100)
R.4368	44789949	44790514	18	SKOR2 (-14678), SMAD2 (+667280)
R.4369	46064816	46065067	18	CTIF (-587)
R.4370	46501202	46501625	18	SMAD7 (-24333), DYM (+485758)
R.4373	49866371	49867117	18	DCC (+202)
R.4374	55815677	55816677	18	NEDD4L (+104567), ALPK2 (+480012)
R.4376	60984656	60985380	18	BCL2 (+2343), PHLPP1 (+602335)
R.4378	74240460	74240582	18	ZNF236 (-295595), ZNF516 (-33375)
R.4379	74799250	74799572	18	MBP (+45314), ZNF236 (+263295)
R.4382	76740262	76740962	18	SALL3 (+337)
R.4383	77280171	77280586	18	CTDP1 (-159422), NFATC1 (+120043)
R.4384	77286052	77286260	18	CTDP1 (-153645), NFATC1 (+125820)
R.4385	345306	345778	19	MIER2 (-749)
R.4388	707791	708568	19	PALM (-921)
R.4389	710823	711001	19	MISP (-40214), PALM (+1811)

R.4390	750790	751241	19	MISP (-110)
R.4394	851650	852311	19	ELANE (+967)
R.4398	1070986	1071622	19	HMHA1 (+5382), POLR2E (+24294)
R.4399	1112327	1113155	19	GPX4 (+8805), SBNO2 (+61541)
R.4400	1155030	1155738	19	SBNO2 (+18898), GPX4 (+51448)
R.4402	1265302	1265999	19	C19orf24 (-9375), CIRBP (+6267)
R.4403	1271275	1271371	19	C19orf24 (-3703)
R.4404	1313502	1313642	19	NDUFS7 (-70058), EFNA2 (+27419)
R.4405	1356269	1356304	19	NDUFS7 (-27343), EFNA2 (+70134)
R.4406	1360208	1360981	19	NDUFS7 (-23035), EFNA2 (+74442)
R.4409	1650236	1650452	19	UQCR11 (-44864), TCF3 (+2260)
R.4410	1775211	1775422	19	ONECUT3 (+22945), ATP8B3 (+36958)
R.4411	1826373	1826957	19	ATP8B3 (-14390), REXO1 (+21787)
R.4414	2041905	2041985	19	BTBD2 (-26226), MKNK2 (+9298)
R.4418	2291373	2291872	19	LINGO3 (+400)
R.4419	2294887	2295092	19	LINGO3 (-2967)
R.4420	2494443	2494595	19	GADD45B (+18394), GNG7 (+208188)
R.4421	2525231	2525634	19	GADD45B (+49308), GNG7 (+177274)
R.4422	2540907	2541104	19	GADD45B (+64881), GNG7 (+161701)
R.4423	2588241	2588629	19	GADD45B (+112310), GNG7 (+114272)
R.4424	2613894	2614039	19	GNG7 (+88740), GADD45B (+137842)
R.4428	3364892	3365201	19	NFIC (-1537)
R.4429	3369759	3370244	19	NFIC (+3418), C19orf77 (+110538)
R.4430	3387811	3388047	19	NFIC (+21345), C19orf77 (+92611)
R.4431	3398706	3398810	19	NFIC (+32174), C19orf77 (+81782)
R.4432	3463788	3464765	19	C19orf77 (+16263), NFIC (+97693)
R.4433	3464875	3465714	19	C19orf77 (+15245), NFIC (+98711)
R.4434	3480363	3480561	19	C19orf77 (+78)
R.4436	3671903	3672411	19	CACTIN (-45344), PIP5K1C (+28320)
R.4438	3969522	3969950	19	DAPK3 (+1332), NMRK2 (+36635)
R.4439	4153671	4153713	19	CREB3L3 (+94)
R.4440	4305072	4305239	19	TMIGD2 (-2728), FSD1 (+559)
R.4441	4474970	4475216	19	ENSG00000167674 (+2809), PLIN4 (+42623)
R.4443	4639397	4639510	19	SEMA6B (-79634), C19orf10 (+30928)
R.4446	5250376	5251120	19	PTPRS (+90066), KDM4B (+281616)
R.4447	5821791	5822369	19	NRTN (-1733)
R.4448	5851255	5851504	19	FUT3 (+102)
R.4450	6271786	6271968	19	MLLT1 (+8082), ACSBG2 (+136192)
R.4451	6376549	6376585	19	PSPN (-707)
R.4452	6459035	6459352	19	SLC25A23 (+552)
R.4453	6475456	6475902	19	DENND1C (+6140), CRB3 (+11385)
R.4454	6476756	6477198	19	DENND1C (+4842), CRB3 (+12683)
R.4456	6659899	6660075	19	CD70 (-68824), TNFSF14 (+10612)
R.4457	6738762	6739268	19	GPR108 (-1401), TRIP10 (-676)
R.4458	6744898	6745717	19	TRIP10 (+5617), SH2D3A (+22291)
R.4460	7580209	7580275	19	ZNF358 (-762)
R.4461	8212234	8212675	19	FBN3 (+2275), CCL25 (+94571)
R.4462	8509471	8509791	19	HNRNPM (-211)
R.4463	8590567	8591513	19	ZNF414 (-11996), MYO1F (+51421)
R.4464	9609395	9609422	19	ZNF560 (-126)

R.4465	10172825	10172899	19	ANGPTL6 (+40610), RDH8 (+48937)
R.4467	10231566	10231711	19	EIF3G (-1061)
R.4468	10444815	10445516	19	RAVER1 (-850)
R.4470	10463831	10464320	19	ICAM3 (-13577), TYK2 (+27276)
R.4471	10697800	10698020	19	AP1M2 (+80)
R.4472	10736006	10736448	19	SLC44A2 (+293)
R.4474	11280822	11281758	19	SPC24 (-14806), KANK2 (+26953)
R.4476	11308876	11309639	19	KANK2 (-1015)
R.4477	11517079	11517268	19	EPOR (-22186), RGL3 (+12830)
R.4478	11649122	11649571	19	CNN1 (-185)
R.4481	12831659	12832077	19	FBXW9 (-24411), TNPO2 (+2942)
R.4482	12936719	12936802	19	RTBDN (+9469), RNASEH2A (+19367)
R.4483	12958803	12958913	19	MAST1 (+9510), DNASE2 (+33424)
R.4485	13080409	13080743	19	DAND5 (+280)
R.4486	13107252	13108217	19	NFIX (+1313), LYL1 (+105946)
R.4487	13112283	13112808	19	NFIX (+6124), LYL1 (+101135)
R.4488	13113455	13113893	19	NFIX (+7252), LYL1 (+100007)
R.4489	13127389	13127873	19	NFIX (+21209), LYL1 (+86050)
R.4493	14073064	14073848	19	DCAF15 (+10152), RFX1 (+43641)
R.4496	14376360	14376960	19	CD97 (-115596), LPHN1 (-59661)
R.4497	14517152	14517447	19	DDX39A (+12879), CD97 (+25044)
R.4499	14607597	14608092	19	GIPC1 (-901)
R.4500	15121385	15121591	19	CCDC105 (-68)
R.4501	15375283	15375374	19	EPHX3 (-32086), BRD4 (+15933)
R.4502	15391832	15391946	19	BRD4 (-627)
R.4503	15528530	15529452	19	AKAP8L (+808)
R.4504	15550742	15550869	19	AKAP8L (-21007), WIZ (+9956)
R.4505	15559119	15559628	19	AKAP8L (-29575), WIZ (+1388)
R.4506	15563870	15564105	19	WIZ (-3226)
R.4507	15568360	15568935	19	WIZ (-7886), RASAL3 (+6729)
R.4509	16178091	16178426	19	TPM4 (-89)
R.4510	16198843	16199792	19	RAB8A (-23121), TPM4 (+20970)
R.4512	16296192	16296301	19	FAM32A (+18)
R.4513	16830613	16830749	19	NWD1 (-110)
R.4516	17357587	17357641	19	NR2F6 (-865)
R.4519	17504632	17504972	19	PLVAP (-16643), BST2 (+11655)
R.4522	17877419	17877733	19	B3GNT3 (-28343), FCHO1 (+19049)
R.4523	17942025	17942411	19	INSL3 (-9835), JAK3 (+16623)
R.4524	17952399	17953157	19	INSL3 (-20395), JAK3 (+6063)
R.4527	18385244	18385930	19	KIAA1683 (-268)
R.4528	18544350	18545062	19	ISYNA1 (+4405), SSBP4 (+14467)
R.4531	18761132	18761792	19	CRTC1 (-33026), TMEM59L (+43222)
R.4532	18873037	18873268	19	COMP (+28970), CRTC1 (+78665)
R.4533	19639553	19639596	19	TSSK6 (-12737), CILP2 (-9482)
R.4534	22805801	22806448	19	ZNF98 (-200977), ZNF492 (-11001)
R.4535	30020162	30020886	19	POP4 (-74400), VSTM2B (+3118)
R.4537	33716258	33716915	19	SLC7A10 (+169)
R.4538	33764284	33764550	19	SLC7A10 (-47661), CEBPA (+29053)
R.4540	35629022	35629791	19	FXYD7 (-4747), LGI4 (-3303), FXYD1 (-1519)
R.4541	35630106	35630305	19	LGI4 (-4102), FXYD7 (-3948), FXYD1 (-720)

R.4542	35630355	35630474	19	LGI4 (-4311), FXYD7 (-3739), FXYD1 (-511)
R.4544	35800743	35801014	19	CD22 (-19211), MAG (+17851)
R.4550	36246402	36246882	19	HSPB6 (+2338), LIN37 (+7380)
R.4551	36247523	36247869	19	HSPB6 (+1284), LIN37 (+8434)
R.4553	36523945	36524454	19	CLIP3 (-405)
R.4554	36642720	36643572	19	COX7A1 (+625)
R.4555	37825307	37825406	19	HKR1 (-223)
R.4556	38746615	38746749	19	PPP1R14A (+549)
R.4557	38755588	38756382	19	SPINT2 (+887)
R.4559	39086923	39087186	19	MAP4K1 (+21509), RYR1 (+162585)
R.4567	41129636	41130041	19	LTBP4 (+26698), NUMBL (+66717)
R.4568	41882327	41882741	19	ENSG00000255730 (-128), TMEM91 (+405)
R.4570	42437245	42437425	19	RABAC1 (+26207), ARHGEF1 (+48820)
R.4573	44039727	44039870	19	ZNF575 (+2443), XRCC1 (+40359)
R.4574	44277951	44278628	19	SMG9 (-19148), KCNN4 (+7119)
R.4576	44306685	44307056	19	LYPD5 (-258)
R.4577	45150513	45150725	19	CEACAM19 (-24375), PVR (+3521)
R.4578	45260501	45261004	19	CBLC (-20373), BCL3 (+8949)
R.4580	45737483	45737880	19	EXOC3L2 (-213)
R.4581	45809373	45809725	19	CKM (+16686), MARK4 (+55033)
R.4582	46032431	46032854	19	VASP (+21969), OPA3 (+55457)
R.4583	46270244	46270700	19	SIX5 (+2012), ENSG00000237452 (+33963)
R.4585	46284048	46284986	19	DMPK (-656)
R.4586	46286064	46286683	19	DMPK (-2513)
R.4587	46288852	46289559	19	ENSG00000268434 (-235)
R.4589	46915351	46915776	19	CCDC8 (+1277), PPP5C (+65313)
R.4590	46999055	46999366	19	CCDC8 (-82370), PPP5D1 (+105246)
R.4595	47633739	47633827	19	SAE1 (-337)
R.4596	48324309	48325153	19	CRX (-366)
R.4600	49220102	49220485	19	MAMSTR (+2684), FUT2 (+21062)
R.4601	49222892	49223814	19	MAMSTR (-375)
R.4602	49575475	49576200	19	KCNA7 (+360)
R.4603	49685846	49686254	19	TRPM4 (+25052), SLC6A16 (+142432)
R.4604	49699879	49700021	19	TRPM4 (+38952), SLC6A16 (+128532)
R.4605	49841517	49842188	19	CD37 (+3196), TEAD2 (+22899)
R.4606	49842655	49843565	19	CD37 (+4453), TEAD2 (+21642)
R.4609	50192739	50193437	19	CPT1C (-1285)
R.4610	50706577	50706737	19	MYH14 (-245)
R.4612	51017855	51018014	19	JOSD2 (-3325), ASPDH (-793)
R.4617	51815359	51815658	19	IGLON5 (+407)
R.4618	52195291	52196034	19	SIGLEC14 (-45609), HAS1 (+31558)
R.4619	52222446	52223099	19	SIGLEC14 (-72719), HAS1 (+4448)
R.4620	52227967	52228048	19	HAS1 (-787)
R.4621	52675203	52675321	19	ZNF836 (-366)
R.4622	52772305	52772371	19	ZNF766 (-486)
R.4623	52956821	52957409	19	ZNF578 (+286)
R.4624	52995647	52996617	19	ZNF808 (-34774), ZNF578 (+39303)
R.4626	54485321	54485460	19	CACNG6 (-10151), CACNG8 (+19097)
R.4627	54727193	54728037	19	LILRB3 (-765)
R.4635	55791817	55792075	19	BRSK1 (-3588), HSPBP1 (-218)

R.4637	55889013	55889387	19	TMEM190 (+996)
R.4638	55997780	55998732	19	SSC5D (-1678)
R.4639	56000201	56000944	19	SSC5D (+639)
R.4640	56061106	56061348	19	ENSG00000231274 (-4318)
R.4642	56158830	56159199	19	CCDC106 (-494)
R.4643	56187810	56188459	19	EPN1 (+144)
R.4644	57154302	57154862	19	SMIM17 (+69)
R.4646	57337421	57337648	19	ZNF835 (-154407), PEG3 (+14529)
R.4647	57610536	57610699	19	ZIM2 (-258521), USP29 (-20793)
R.4651	58545333	58545728	19	ZSCAN1 (+97)
R.4652	58570454	58570491	19	ZNF135 (-134)
R.4653	58629901	58629975	19	ZSCAN18 (-145)
R.4654	58715251	58716004	19	ZNF544 (-24442), ZNF274 (+21232)
R.4655	58868256	58869004	19	A1BG (-3765)
R.4657	821854	822788	20	FAM110A (+7901), ANGPT4 (+74639)
R.4658	825415	825634	20	FAM110A (+11105), ANGPT4 (+71435)
R.4665	2801711	2801995	20	VPS16 (-19496), TMEM239 (+4874)
R.4667	3190202	3190600	20	ITPA (+395)
R.4670	3661910	3662546	20	ADAM33 (+665)
R.4674	4804581	4804718	20	RASSF2 (-359)
R.4675	5892091	5892599	20	CHGB (+269)
R.4676	11871375	11871396	20	NONE
R.4677	11898478	11898557	20	NONE
R.4678	13975853	13976143	20	SEL1L2 (-4736), MACROD2 (-17)
R.4680	17595355	17595472	20	DSTN (+44906), RRBP1 (+67514)
R.4681	17674195	17674250	20	BANF2 (-94)
R.4682	19866743	19867145	20	RIN2 (-221)
R.4683	19869398	19870109	20	NAA20 (-128006), RIN2 (+2589)
R.4687	21377671	21378072	20	NKX2-4 (+794)
R.4688	23015908	23015936	20	SSTR4 (-135)
R.4689	30060868	30061002	20	REM1 (-2161), DEFB124 (-119)
R.4690	30406997	30407388	20	MYLK2 (+82)
R.4691	30433405	30433673	20	FOXS1 (-119)
R.4695	31407775	31408213	20	MAPRE1 (+295)
R.4696	31446444	31446967	20	EFCAB8 (-23)
R.4698	33585040	33585435	20	MYH7B (+22032), TRPC4AP (+95436)
R.4699	34025927	34026478	20	GDF5OS (+5376), GDF5 (+16365)
R.4700	35168979	35169380	20	MYL9 (-707)
R.4702	35490815	35491355	20	DSN1 (-88931), SOGA1 (+1004)
R.4703	36147549	36148182	20	NNAT (-1751)
R.4708	36153865	36154292	20	BLCAP (+2254), NNAT (+4462)
R.4710	36889364	36889389	20	TGM2 (-95603), BPI (-43148)
R.4717	42545022	42545443	20	TOX2 (+1741), JPH2 (+270985)
R.4718	42839593	42839882	20	JPH2 (-23520), FITM2 (+100071)
R.4723	44657685	44657798	20	SLC12A5 (+7413), NCOA5 (+60849)
R.4724	45313200	45313808	20	SLC13A3 (-33425), TP53RK (+4914)
R.4725	46415688	46415859	20	SULF2 (-444)
R.4729	50108912	50109375	20	KCNG1 (-469478), NFATC2 (+50114)
R.4734	56239154	56239706	20	ZBP1 (-43901), PMEPA1 (+45528)
R.4735	56247302	56248127	20	ZBP1 (-52186), PMEPA1 (+37243)

R.4736	56287004	56287532	20	PMEPA1 (-2310)
R.4741	57471654	57471672	20	NELFCD (-84600), GNAS (+43894)
R.4744	57875545	57876201	20	EDN3 (+391)
R.4746	60470081	60470267	20	TAF4 (+170692), CDH4 (+642692)
R.4747	60639404	60639721	20	TAF4 (+1303), CDH4 (+812081)
R.4748	60882647	60882801	20	ADRM1 (+4663), LAMA5 (+59644)
R.4750	61447038	61447174	20	COL9A3 (-1308)
R.4751	61508481	61508590	20	TCFL5 (-15421), DIDO1 (+60738)
R.4754	61977221	61978070	20	CHRNA4 (+15093), COL20A1 (+53108)
R.4755	62092443	62092878	20	CHRNA4 (-99922), KCNQ2 (+11330)
R.4756	62153067	62153431	20	PPDPF (+1172), PTK6 (+15446)
R.4761	62405935	62406721	20	SLC2A4RG (+35114), ZBTB46 (+56239)
R.4763	62679255	62679713	20	ZNF512B (+629)
R.4765	62693971	62694005	20	TCEA2 (-509)
R.4770	33749529	33749986	21	URB1 (+15577), MRAP (+85634)
R.4771	35444367	35445342	21	SLC5A3 (-1015), MRPS6 (-968)
R.4772	35552652	35553467	21	KCNE2 (-183263), SLC5A3 (+107190)
R.4774	38362727	38362754	21	HLCS (-23968), RIPPLY3 (-16122)
R.4776	40194735	40195074	21	ETS2 (+17674), PSMG1 (+360872)
R.4778	42218551	42219193	21	DSCAM (+193)
R.4786	44898090	44898206	21	SIK1 (-51140), HSF2BP (+181226)
R.4787	45177538	45178331	21	CSTB (+18391), PDXK (+38942)
R.4788	45232587	45232601	21	AGPAT3 (-112295), RRP1 (+23200)
R.4789	45705429	45706101	21	AIRE (+2)
R.4792	45789122	45789379	21	LRRC3 (-86118), TRPM2 (+16112)
R.4794	46367939	46368090	21	ADARB1 (-126478), C21orf67 (-8255)
R.4795	46847581	46847898	21	POFUT2 (-139927), COL18A1 (-27663)
R.4797	46875142	46875417	21	COL18A1 (-123)
R.4798	46890907	46891093	21	COL18A1 (+15597), SLC19A1 (+71385)
R.4799	46897181	46898137	21	COL18A1 (+22256), SLC19A1 (+64726)
R.4804	17680477	17680578	22	CECR5 (-40361), CECR1 (+22351)
R.4805	18043043	18043745	22	SLC25A18 (+255)
R.4808	18231406	18231825	22	BID (+25166), BCL2L13 (+110260)
R.4815	19949901	19950166	22	TXNRD2 (-20701), ARVCF (+54297)
R.4821	24577288	24577448	22	SUSD2 (+141)
R.4823	24979254	24979964	22	GGT1 (-19559), SNRPD3 (+28138)
R.4825	25160308	25160406	22	PIWIL3 (+10326), GGT1 (+161189)
R.4826	25758455	25758737	22	LRP5L (+18948), CRYBB2 (+143107)
R.4829	26137980	26137997	22	MYO18B (-131)
R.4830	27152635	27153015	22	CRYBA4 (+134897)
R.4831	27834439	27834629	22	MN1 (+362952), CRYBA4 (+816606)
R.4833	28191222	28191914	22	MN1 (+5918)
R.4835	29426511	29426832	22	KREMEN1 (-42434), ZNRF3 (+147092)
R.4838	29706704	29707585	22	GAS2L1 (+4114), RASL10A (+4600)
R.4839	29707782	29708246	22	RASL10A (+3731), GAS2L1 (+4983)
R.4840	30476281	30476452	22	HORMAD2 (+204)
R.4843	31518188	31518963	22	INPP5J (-404)
R.4844	31536540	31537064	22	PLA2G3 (-209)
R.4845	31643997	31644190	22	LIMK2 (-379)
R.4847	35695066	35695301	22	TOM1 (-613)

R.4850	36682843	36682870	22	APOL1 (+33733), MYH9 (+101206)
R.4852	36806001	36806443	22	MYH9 (-22159), TXN2 (+71855)
R.4858	37584018	37584441	22	C1QTNF6 (+99)
R.4859	37608037	37608819	22	SSTR3 (-66)
R.4861	37955927	37956207	22	CDC42EP1 (-387)
R.4863	38714395	38714466	22	CSNK1E (-1018)
R.4866	39687344	39688265	22	PDGFB (-47049), RPL3 (+27971)
R.4867	39918625	39919237	22	ATF4 (+2362), RPS19BP1 (+9929)
R.4869	41697184	41697410	22	ZC3H7B (-229)
R.4872	42347991	42348061	22	CENPM (-4858)
R.4873	42470063	42470123	22	NAGA (-3259), FAM109B (-162)
R.4874	42474656	42475135	22	SMDT1 (-803)
R.4875	42685734	42686055	22	TCF20 (-74447), NFAM1 (+142506)
R.4876	42763955	42764390	22	TCF20 (-152725), NFAM1 (+64228)
R.4877	43042879	43043270	22	CYB5R3 (-2560)
R.4879	43739629	43739992	22	SCUBE1 (-417)
R.4886	45124555	45125480	22	ARHGAP8 (+26636), PHF21B (+280563)
R.4887	45608345	45608686	22	UPK3A (-72347), NUP50 (+48794)
R.4888	45899292	45899736	22	FBLN1 (+743)
R.4891	46458783	46459327	22	PPARA (-87444), WNT7B (-86046)
R.4892	46472568	46473343	22	WNT7B (-99947), PPARA (-73543)
R.4895	46516291	46516503	22	WNT7B (-143388), PPARA (-30102)
R.4896	46932642	46932833	22	CELSR1 (+329)
R.4902	50025915	50026188	22	BRD1 (+192400)
R.4906	50493570	50494436	22	TTLL8 (+1361), PIM3 (+139842)
R.4907	50705948	50706248	22	MAPK12 (-5844), MAPK11 (+2724)
R.4911	50985681	50986031	22	TYMP (-17412), SYCE3 (+15478)
R.4912	51016501	51017166	22	CPT1B (+262)
R.4913	51158550	51159147	22	ACR (-17775), SHANK3 (+45779)

Supplementary Table 10b. Hypermethylated DMRs BAV-D (fc 10%), and associated genes				
**Region**	**DMR start**	**DMR end**	**Chr**	**Gene**
R.2	901449	901725	1	PLEKHN1 (-290)
R.26	2231925	2232166	1	RER1 (-91226), SKI (+71912)
R.101	6419606	6419906	1	GPR153 (-98721), ACOT7 (+26117)
R.112	7692321	7692367	1	VAMP3 (-138985), CAMTA1 (+846960)
R.114	8271918	8272277	1	ERRFI1 (-185730), SLC45A1 (-105788)
R.121	9599256	9599380	1	SLC25A33 (-223)
R.122	9600345	9600721	1	SLC25A33 (+992)
R.124	9714280	9714810	1	PIK3CD (+2742), CLSTN1 (+170039)
R.134	12203571	12204096	1	TNFRSF1B (-23226), TNFRSF8 (+80400)
R.135	12226913	12226947	1	TNFRSF1B (-130)
R.180	23495997	23496648	1	LUZP1 (+7978), KDM1A (+150337)
R.183	24126017	24126076	1	GALE (+845)
R.189	24882153	24882207	1	NCMAP (-422)
R.195	25349006	25349681	1	RUNX3 (-57843), SYF2 (+209640)
R.198	26373132	26373407	1	SLC30A2 (-646)
R.254	46958475	46959349	1	DMBX1 (-13757), FAAH (+98975)
R.301	111415232	111416181	1	CD53 (-69)
R.323	147245168	147245626	1	GJA5 (+87)
R.336	150480534	150480856	1	ECM1 (+143)
R.340	151103642	151104186	1	SEMA6C (+15019), GABPB2 (+60834)
R.375	156646065	156646485	1	NES (+914)
R.377	156863643	156864023	1	PEAR1 (+321)
R.381	159796231	159796658	1	SLAMF8 (-95)
R.384	160053689	160053947	1	KCNJ9 (+2458), IGSF8 (+14661)
R.401	164290179	164290241	1	PBX1 (-238662), NUF2 (+998466)
R.410	167598521	167599124	1	RCSD1 (-507)
R.430	186649985	186650479	1	PTGS2 (-673)
R.435	200992902	200993069	1	KIF21B (-158)
R.436	201368946	201369031	1	LAD1 (-253)
R.437	201476557	201476775	1	PHLDA3 (-38354), CSRP1 (+1918)
R.468	213090116	213090240	1	VASH2 (-33799), FLVCR1 (+58581)
R.469	214152076	214153036	1	PROX1 (-8730), RPS6KC1 (+927959)
R.472	214158373	214158686	1	PROX1 (-2756)
R.476	217313699	217314284	1	ESRRG (-417197), GPATCH2 (+490432)
R.502	236557182	236557758	1	EDARADD (-108)
R.510	247578859	247579608	1	NLRP3 (-2117)
R.601	45028225	45028269	2	SIX3 (-140655), CAMKMT (+439144)
R.669	102091115	102091656	2	RFX8 (-221)
R.674	105484508	105484823	2	MRPS9 (-169775), POU3F3 (+12697)
R.686	113404519	113404678	2	NT5DC4 (-74464), SLC20A1 (+1165)
R.720	158184192	158184543	2	ERMN (-143)
R.727	171569132	171570003	2	SP5 (-2293)
R.728	171573891	171574592	2	GAD1 (-98830), SP5 (+2381)
R.729	171669275	171670134	2	GAD1 (-3367)
R.730	171670878	171671795	2	GAD1 (-1735)
R.731	171676306	171676925	2	GORASP2 (-108420), GAD1 (+3544)
R.732	171678751	171679402	2	GORASP2 (-105959), GAD1 (+6005)
R.737	172967819	172968752	2	DLX2 (-658)

R.747	176989349	176989586	2	HOXD8 (-5000)
R.748	177503592	177503639	2	HNRNPA3 (-573675), MTX2 (+369469)
R.754	198650985	198651347	2	BOLL (+132)
R.775	220117599	220117945	2	TUBA4A (+982)
R.810	238581924	238582364	2	RAB17 (-82408), LRRFIP1 (-18838)
R.895	45208900	45209252	3	CDCP1 (-21162), TMEM158 (+58694)
R.896	46249795	46250518	3	CCR1 (-270)
R.897	46411447	46411541	3	CCR5 (-139)
R.898	46448084	46448963	3	ACKR5 (-525)
R.908	49057259	49057884	3	DALRD3 (-1568), NDUFAF3 (-872)
R.912	50284010	50284305	3	LSMEM2 (-32300), GNAI2 (+10774)
R.915	50310766	50311213	3	LSMEM2 (-5468), GNAI2 (+37606)
R.934	55517101	55518091	3	LRTM1 (-555495), WNT5A (+6377)
R.938	62360674	62361449	3	FEZF2 (-1872)
R.1004	141516232	141516291	3	ATP1B3 (-79194), GRK7 (+19272)
R.1015	157824217	157824510	3	RSRC1 (-3477), SHOX2 (-415)
R.1044	193587490	193587939	3	HES1 (-266219), OPA1 (+276782)
R.1048	194406190	194406362	3	LSG1 (-13070), XXYLT1 (+585620)
R.1050	195384528	195384742	3	APOD (-73559), MUC20 (-63118)
R.1067	779230	779568	4	CPLX1 (+40587), PCGF3 (+79845)
R.1074	1166528	1166767	4	SPON2 (+13)
R.1100	4855629	4856510	4	STX18 (-312315), MSX1 (-5323)
R.1105	4868723	4869172	4	MSX1 (+7555), CYTL1 (+152251)
R.1106	4870486	4871426	4	MSX1 (+9563), CYTL1 (+150243)
R.1107	4873966	4874548	4	MSX1 (+12864), CYTL1 (+146942)
R.1112	6690702	6691093	4	S100P (-3898)
R.1123	8193123	8193353	4	ABLIM2 (-32802), SH3TC1 (-7856)
R.1144	25656514	25656865	4	SLC34A2 (-776)
R.1150	38859706	38859770	4	TLR6 (-1301)
R.1158	43900946	43901084	4	KCTD8 (+549809)
R.1167	55990582	55991418	4	KDR (+756)
R.1173	76944785	76945459	4	CXCL10 (-472)
R.1191	99850664	99851281	4	EIF4E (-689)
R.1197	111554385	111554966	4	PITX2 (-10417)
R.1198	111558602	111559134	4	PITX2 (-14609)
R.1199	111562513	111563111	4	PITX2 (-18553)
R.1215	155413392	155413789	4	DCHS2 (-101142), PLRG1 (+57996)
R.1217	155662438	155662795	4	LRAT (-2506)
R.1225	169798931	169799308	4	CBR4 (+132289), PALLD (+380865)
R.1226	174415431	174415868	4	SCRG1 (-94963), HAND2 (+35730)
R.1233	183061955	183062576	4	TENM3 (-102316)
R.1251	368804	369088	5	AHRR (+64655), C5orf55 (+74312)
R.1268	1594021	1594330	5	LPCAT1 (-70084), MRPL36 (+205833)
R.1269	1594808	1594863	5	LPCAT1 (-70744), MRPL36 (+205173)
R.1307	40680550	40681444	5	PTGER4 (+1397), PRKAA1 (+117479)
R.1308	40681893	40682333	5	PTGER4 (+2513), PRKAA1 (+116363)
R.1312	42992998	42993450	5	SEPP1 (-181043), ZNF131 (-128476)
R.1314	43017351	43017982	5	SEPP1 (-205486), ZNF131 (-104033)
R.1317	43040438	43040580	5	SEPP1 (-228328), ZNF131 (-81191)
R.1319	49737236	49737349	5	EMB (-92)

R.1320	50262177	50262796	5	ISL1 (-416434), PARP8 (+299715)
R.1321	50263500	50264156	5	ISL1 (-415093), PARP8 (+301056)
R.1322	50264516	50264836	5	ISL1 (-414245), PARP8 (+301904)
R.1323	50673808	50674203	5	ISL1 (-4915)
R.1324	50683847	50684720	5	ISL1 (+5363)
R.1336	72526137	72526379	5	TMEM174 (+57236), FOXD1 (+218094)
R.1340	72731905	72732265	5	FOXD1 (+12267), TMEM174 (+263063)
R.1344	76145809	76146148	5	S100Z (+55)
R.1345	76373022	76373803	5	ZBED3 (+9735), AGGF1 (+47203)
R.1354	95159365	95159639	5	GLRX (-1084)
R.1366	126205009	126205081	5	LMNB1 (+92205), MARCH3 (+161455)
R.1371	131408382	131409352	5	CSF2 (-616)
R.1375	132155330	132155460	5	SEPT8 (-42328), SHROOM1 (+11195)
R.1378	133451237	133452188	5	TCF7 (+1311), SKP1 (+61016)
R.1379	134362967	134363877	5	PITX1 (+6566), CATSPER3 (+59826)
R.1380	134363973	134364717	5	PITX1 (+5643), CATSPER3 (+60749)
R.1381	134365520	134366377	5	PITX1 (+4039), CATSPER3 (+62353)
R.1382	134366534	134367394	5	PITX1 (+3024), CATSPER3 (+63368)
R.1383	134376247	134377058	5	PITX1 (-6665), H2AFY (+358659)
R.1384	134385984	134386369	5	PITX1 (-16189), H2AFY (+349135)
R.1390	134870506	134870977	5	NEUROG1 (+897)
R.1391	134880436	134880895	5	NEUROG1 (-9027), CXCL14 (+34303)
R.1424	148442694	148442890	5	SH3TC2 (-105)
R.1436	150051778	150052349	5	MYOZ3 (+11620), RBM22 (+28605)
R.1453	170877840	170878209	5	C5orf50 (-334851), FGF18 (+31365)
R.1461	172655841	172655948	5	NKX2-5 (+6465), BNIP1 (+84450)
R.1462	172656713	172657465	5	NKX2-5 (+5271), BNIP1 (+85644)
R.1463	172658730	172659730	5	NKX2-5 (+3130), BNIP1 (+87785)
R.1464	172672684	172673349	5	NKX2-5 (-10657), STC2 (+83489)
R.1470	176784715	176785296	5	RGS14 (+168)
R.1493	1391265	1392250	6	FOXC1 (-218923), FOXF2 (+1689)
R.1494	1392624	1393336	6	FOXC1 (-217701), FOXF2 (+2911)
R.1495	1394071	1395061	6	FOXC1 (-216115), FOXF2 (+4497)
R.1516	5996893	5997199	6	NRN1 (+10154), FARS2 (+735769)
R.1537	24908452	24909127	6	FAM65B (+2405), GMNN (+133624)
R.1542	27107670	27107756	6	HIST1H4I (+637)
R.1543	27791727	27791876	6	HIST1H4J (-82)
R.1544	27841633	27842098	6	HIST1H3I (-1767), HIST1H4L (-577)
R.1564	29942951	29943209	6	ZNRD1 (-85951), HLA-A (+34043)
R.1568	30068832	30068899	6	RNF39 (-25202), TRIM31 (+12017)
R.1583	30510294	30510459	6	PRR3 (-14286), HLA-E (+53133)
R.1593	30650844	30651563	6	DHX16 (-10390), PPP1R18 (+4468)
R.1596	30689710	30689865	6	MDC1 (-4122), TUBB (+1810)
R.1599	30710898	30711863	6	FLOT1 (-871), IER3 (+950)
R.1600	30711904	30712060	6	FLOT1 (-1472), IER3 (+349)
R.1601	30712282	30712338	6	FLOT1 (-1800), IER3 (+21)
R.1602	30712499	30712680	6	FLOT1 (-2080), IER3 (-259)
R.1622	31527889	31528239	6	LTA (-11767), NFKBIL1 (+12706)
R.1645	31705273	31705988	6	MSH5 (-2167), MSH5-SAPCD1 (-2166), CLIC1 (-536)
R.1676	32054441	32054867	6	TNXB (-40749), ATF6B (+41363)

R.1679	32064206	32065011	6	TNXB (-50704), ATF6B (+31408)
R.1708	32765281	32765402	6	HLA-DQB2 (-34075), HLA-DOB (+19483)
R.1770	41340058	41340668	6	FOXP4 (-173801), NCR2 (+36836)
R.1771	41342202	41342944	6	FOXP4 (-171591), NCR2 (+39046)
R.1772	41343647	41343830	6	FOXP4 (-170425), NCR2 (+40212)
R.1777	42110740	42110867	6	GUCA1A (-12380), TAF8 (+92542)
R.1787	45645147	45645497	6	SUPT3H (-299652), CLIC5 (+402810)
R.1796	56820778	56820869	6	DST (-313030), KIAA1586 (-90523)
R.1819	108492271	108492769	6	NR2E1 (+5258), SNX3 (+89944)
R.1823	111927480	111928234	6	TRAF3IP2 (-408)
R.1834	133561649	133562056	6	EYA4 (-636)
R.1837	136914796	136915556	6	MAP7 (-67566), MAP3K5 (+198480)
R.1843	139351113	139351353	6	HECA (-105016), REPS1 (-41835)
R.1873	166901788	166901854	6	MPC1 (-105335), RPS6KA2 (+374218)
R.1916	633050	634037	7	PDGFA (-74513), PRKAR1B (+119300)
R.1936	1265635	1266616	7	ZFAND2A (-66312), UNCX (-6417)
R.1937	1283896	1284732	7	UNCX (+11771), MICALL2 (+214824)
R.1940	1362621	1363124	7	UNCX (+90330), MICALL2 (+136265)
R.1986	22767384	22767571	7	IL6 (+1975), TOMM7 (+94992)
R.1991	26193109	26193805	7	NFE2L3 (+1597), HNRNPA2B1 (+46909)
R.2009	27260102	27260466	7	EVX1 (-21880), HOXA13 (-20559)
R.2010	27280585	27281216	7	EVX1 (-1263)
R.2012	27291695	27292614	7	EVX1 (+9991), HIBADH (+410459)
R.2030	36764019	36764205	7	AOAH (+41)
R.2032	39170497	39170763	7	RALA (-492452), POU6F2 (+153032)
R.2053	64348873	64349706	7	ZNF273 (-14335), ZNF138 (+94487)
R.2062	71799177	71799982	7	CALN1 (+77778)
R.2094	100091181	100091786	7	AGFG2 (-45364), NYAP1 (+9934)
R.2095	100199952	100200009	7	PCOLCE (+181)
R.2105	101596218	101596404	7	SH2B2 (-332094), CUX1 (+137020)
R.2150	148394542	148394994	7	CUL1 (-1236)
R.2151	149322908	149323101	7	ZNF746 (-128107), KRBA1 (-89143)
R.2171	155079250	155079356	7	INSIG1 (-10183), HTR5A (+217269)
R.2172	155165891	155166290	7	EN2 (-84733), INSIG1 (+76605)
R.2189	157648386	157648749	7	DNAJB6 (+518908), PTPRN2 (+731803)
R.2211	1765309	1765387	8	ARHGEF10 (-6794), CLN8 (+53420)
R.2223	1992746	1993528	8	MYOM2 (-18)
R.2238	11558169	11559156	8	GATA4 (-3050)
R.2259	23559839	23560590	8	NKX3-1 (-19775), NKX2-6 (+3896)
R.2260	23562918	23563572	8	NKX2-6 (+866)
R.2261	23566799	23567747	8	NKX2-6 (-3162)
R.2262	23571706	23571740	8	NKX2-6 (-7612), STC1 (+140597)
R.2263	23583662	23584098	8	NKX2-6 (-19769), STC1 (+128440)
R.2264	23584746	23585186	8	NKX2-6 (-20855), STC1 (+127354)
R.2267	24859074	24859537	8	DOCK5 (-183074), NEFM (+88781)
R.2268	25897201	25898191	8	EBF2 (+5217), CDCA2 (+581183)
R.2269	25898220	25898839	8	EBF2 (+4383), CDCA2 (+582017)
R.2270	25899257	25900020	8	EBF2 (+3274), CDCA2 (+583126)
R.2284	37378355	37379119	8	ZNF703 (-174532), KCNU1 (+736845)
R.2297	42623718	42623946	8	CHRNA6 (+97)

R.2348	133773093	133773115	8	TMEM71 (-276)
R.2364	143781340	143781406	8	LY6K (-156)
R.2375	144640306	144640397	8	GSDMD (+4975), NAPRT1 (+20431)
R.2400	969473	969544	9	DMRT3 (-7455), DMRT1 (+127819)
R.2402	14348844	14349430	9	NFIB (-35471), ZDHHC21 (+344332)
R.2409	79631737	79632225	9	FOXB2 (-2590)
R.2414	98784802	98785110	9	ERCC6L2 (+146973), HSD17B3 (+279478)
R.2430	130616110	130616917	9	ENG (+521)
R.2446	139652837	139653003	9	LCN8 (-242)
R.2482	6214016	6214079	10	PFKFB3 (-30846), RBM17 (+82739)
R.2525	50396425	50396833	10	C10orf128 (-225)
R.2536	72254314	72254335	10	PALD1 (+15748), PRF1 (+108206)
R.2557	88021787	88022744	10	GRID1 (+103969)
R.2559	88124063	88124965	10	GRID1 (+1721)
R.2580	100993553	100993965	10	HPS1 (-787076), HPSE2 (+1860)
R.2581	100996347	100996747	10	HPSE2 (-928)
R.2582	101292477	101292583	10	NKX2-3 (-160)
R.2587	102892131	102892787	10	TLX1 (+3202), LBX1 (+97092)
R.2588	102894441	102895133	10	TLX1 (+5530), LBX1 (+94764)
R.2589	102895708	102896640	10	TLX1 (+6917), LBX1 (+93377)
R.2590	102898409	102899285	10	TLX1 (+9590), LBX1 (+90704)
R.2591	102995440	102995987	10	BTRC (-118106), LBX1 (-6163)
R.2592	102998662	102998762	10	BTRC (-115108), LBX1 (-9161)
R.2603	112402954	112403495	10	RBM20 (-930)
R.2607	118890977	118891266	10	KIAA1598 (-126034), VAX1 (+6445)
R.2611	119305798	119306659	10	EMX2 (+4274), RAB11FIP2 (+499885)
R.2612	119310053	119310404	10	EMX2 (+8274), RAB11FIP2 (+495885)
R.2618	122708898	122709814	10	WDR11 (+98669), FGFR2 (+648561)
R.2628	125853232	125853748	10	CHST15 (-1520)
R.2637	129979292	129979375	10	MKI67 (-54685)
R.2638	130063978	130064296	10	MKI67 (-139488)
R.2650	131682386	131682509	10	EBF3 (+79657), MGMT (+417000)
R.2651	131764852	131764932	10	EBF3 (-2787)
R.2669	134498830	134498960	10	NKX6-2 (+100661), INPP5A (+147571)
R.2679	134872087	134872413	10	NKX6-2 (-272694), GPR123 (-29159)
R.2687	268923	269468	11	NLRP6 (-9374), PSMD13 (+32219)
R.2689	314044	314341	11	IFITM1 (+340)
R.2693	414951	415457	11	SIGIRR (-228)
R.2727	2162183	2162406	11	IGF2 (+173)
R.2728	2162445	2162616	11	IGF2 (-63)
R.2729	2165656	2165961	11	IGF2 (-3341)
R.2731	2211939	2212225	11	TH (-19047), ASCL2 (+80100)
R.2739	2466788	2466909	11	KCNQ1 (+628)
R.2745	2889602	2890587	11	CDKN1C (+17016), KCNQ1 (+423874)
R.2748	2919798	2920350	11	SLC22A18 (-3576)
R.2761	6342214	6342357	11	PRKCDBP (-409)
R.2774	14994561	14994989	11	CALCA (-875)
R.2782	18270324	18270548	11	SAA2 (-254)
R.2788	20384886	20385170	11	HTATIP2 (-261)
R.2792	31837144	31837429	11	PAX6 (+2222), ELP4 (+305990)

R.2797	32450244	32450692	11	WT1 (+6708), RCN1 (+338018)
R.2798	32454216	32455192	11	WT1 (+2472), RCN1 (+342254)
R.2799	32459626	32460587	11	WT1 (-2931)
R.2800	32460656	32461240	11	WT1 (-3772)
R.2801	33744366	33744977	11	CD59 (+13313), KIAA1549L (+180851)
R.2805	44324759	44325258	11	ALX4 (+6707), EXT2 (+207262)
R.2837	60145275	60146078	11	MS4A7 (-278)
R.2838	60623144	60623639	11	PTGDR2 (+52)
R.2859	63974123	63974153	11	FERMT3 (-68)
R.2860	63974229	63975026	11	FERMT3 (+422)
R.2901	68147880	68148079	11	PPP6R3 (-80258), LRP5 (+67903)
R.2912	69454823	69455028	11	CCND1 (-929)
R.2913	69455111	69455217	11	CCND1 (-691)
R.2943	82443149	82443614	11	PRCP (+168090)
R.2956	102139392	102139524	11	BIRC3 (-48768), YAP1 (+158266)
R.2958	104905834	104906381	11	CASP1 (-232)
R.2959	104915759	104916331	11	CARD16 (-3)
R.2976	115376098	115376699	11	CADM1 (-1266)
R.2988	119294283	119294408	11	THY1 (+492)
R.3004	124630919	124631666	11	ESAM (+887)
R.3012	128554939	128555317	11	ETS1 (-97691), FLI1 (-8762)
R.3026	131779255	131779604	11	NTM (-1926)
R.3028	131941027	131941989	11	NTM (+160152), OPCML (+872058)
R.3066	6898217	6898975	12	CD4 (-42)
R.3072	7070562	7070732	12	PHB2 (+9341), PTPN6 (+10213)
R.3074	8088741	8089207	12	SLC2A3 (-103)
R.3084	22486029	22486484	12	ST8SIA1 (+1391), CMAS (+287149)
R.3100	49371182	49371751	12	WNT1 (-931)
R.3101	49374710	49375669	12	WNT1 (+2792), DDN (+17902)
R.3105	49726661	49727110	12	C1QL4 (+4085), TROAP (+9824)
R.3125	54090254	54090519	12	ATP5G2 (-20278), CALCOCO1 (+30837)
R.3128	54360460	54360836	12	HOXC11 (-6262), HOXC12 (+12030)
R.3129	54389891	54389982	12	HOXC9 (-3966)
R.3130	54398561	54398809	12	HOXC8 (-4147)
R.3132	54410576	54410632	12	HOXC6 (-11538), HOXC8 (+7772)
R.3133	54427884	54428592	12	HOXC4 (-19423), HOXC5 (+1601)
R.3171	85671811	85672623	12	ALX1 (-1668)
R.3175	93967225	93967580	12	CRADD (-104090), SOCS2 (+3813)
R.3179	106978920	106979430	12	RFX4 (-15740), POLR3B (+227739)
R.3181	108991991	108992114	12	TMEM119 (-153)
R.3199	114833043	114833950	12	RBM19 (-429386), TBX5 (+12750)
R.3200	114834201	114835179	12	RBM19 (-430579), TBX5 (+11557)
R.3205	114847043	114847641	12	TBX5 (-1095)
R.3206	114851495	114852359	12	TBX5 (-5680), TBX3 (+269468)
R.3207	114877063	114877421	12	TBX5 (-30995), TBX3 (+244153)
R.3208	114887095	114887847	12	TBX5 (-41224), TBX3 (+233924)
R.3213	115130124	115130994	12	TBX3 (-9164)
R.3216	115138445	115138766	12	TBX3 (-17211)
R.3217	115174628	115175155	12	TBX3 (-53497)
R.3218	116043958	116044032	12	TBX3 (-922600), MED13L (+671148)

R.3236	122519119	122519951	12	MLXIP (+2907), IL31 (+139211)
R.3260	125197665	125198295	12	NCOR2 (-218182), SCARB1 (+150413)
R.3261	125397564	125398157	12	SCARB1 (-49468), UBC (+2033)
R.3271	130529673	130530121	12	TMEM132D (-141686), FZD10 (-117107)
R.3296	20735967	20736075	13	GJA3 (-833)
R.3298	20805094	20805895	13	GJB2 (-41576), GJB6 (+1039)
R.3301	25085301	25085776	13	PARP4 (+1409), C1QTNF9 (+204235)
R.3311	36049335	36050158	13	MAB21L1 (+1085), NBEA (+533323)
R.3330	88326635	88326918	13	SLITRK5 (+1907)
R.3341	100085073	100085455	13	GPR183 (-125605), TM9SF2 (-68407)
R.3345	105791890	105792346	13	DAOA (-326474)
R.3357	111061859	111062287	13	COL4A2 (+102459), RAB20 (+152007)
R.3364	111521981	111522314	13	ARHGEF7 (-245476), ING1 (+154819)
R.3378	112999748	113000664	13	SPACA7 (-30427), SOX1 (+278293)
R.3379	113000801	113001355	13	SPACA7 (-29555), SOX1 (+279165)
R.3414	114880761	114881194	13	GAS6 (-313938), RASA3 (+17108)
R.3442	36985063	36985905	14	MBIP (-195602), NKX2-1 (+3949)
R.3444	37053664	37054509	14	NKX2-8 (-2275)
R.3445	37128011	37128790	14	PAX9 (-2669)
R.3446	37130212	37130313	14	PAX9 (-807)
R.3448	38057752	38058243	14	FOXA1 (+6241), MIPOL1 (+390843)
R.3457	61103667	61104329	14	SIX1 (+12182), SIX6 (+128329)
R.3458	61120234	61120783	14	SIX1 (-4329)
R.3460	61122774	61123405	14	SIX1 (-6910), SIX4 (+67762)
R.3462	64761409	64761548	14	ESR2 (-401)
R.3467	70347833	70348461	14	SMOC1 (+2014), SLC8A3 (+307640)
R.3482	81893829	81894271	14	STON2 (-302)
R.3493	95233665	95234658	14	GSC (+2400), SERPINA3 (+155434)
R.3494	95234822	95235127	14	GSC (+1587), SERPINA3 (+156247)
R.3495	95237386	95238156	14	GSC (-1209)
R.3496	95238671	95239570	14	GSC (-2559)
R.3504	99728984	99729088	14	BCL11B (+8529)
R.3528	104372267	104372322	14	PPP1R13B (-58368), C14orf2 (+15543)
R.3536	105126510	105126697	14	TMEM179 (-54620), INF2 (-29370)
R.3553	105952171	105953092	14	CRIP1 (-22)
R.3554	105953244	105954118	14	TMEM121 (-39259), CRIP1 (+1027)
R.3569	33359788	33360485	15	FMN1 (-52)
R.3570	37172274	37173017	15	MEIS2 (+219440)
R.3571	37174376	37175029	15	MEIS2 (+217383)
R.3589	45018591	45018905	15	TRIM69 (-2438)
R.3595	53083532	53083978	15	ONECUT1 (-1546)
R.3603	65341703	65341995	15	SLC51B (+4141), RASL12 (+18601)
R.3638	83953690	83954395	15	BNC1 (-577)
R.3646	90547692	90548043	15	ZNF710 (+3244), IDH2 (+97868)
R.3653	93579332	93579395	15	RGMA (+37728), CHD2 (+136306)
R.3658	93652578	93652766	15	RGMA (-35580)
R.3660	96868971	96869027	15	NR2F2 (-4947)
R.3662	96877194	96877855	15	NR2F2 (+3579)
R.3666	96887867	96888433	15	NR2F2 (+14204)
R.3700	1047460	1047820	16	SSTR5 (-81141), SOX8 (+15832)

R.3718	1592816	1593766	16	TMEM204 (+9717), IFT140 (+68820)
R.3761	10970801	10971105	16	CIITA (-102)
R.3784	29296614	29296797	16	NPIPB11 (+118644), LAT (+300559)
R.3799	31227497	31227884	16	PYDC1 (+989)
R.3800	31366118	31366536	16	ITGAX (-128)
R.3823	55365815	55366021	16	MMP2 (-146965), IRX6 (+8246)
R.3828	56641101	56642024	16	MT2A (-548)
R.3865	73097364	73098356	16	ZFHX3 (-15586)
R.3879	84328104	84328208	16	WFDC1 (-96)
R.3893	85205741	85206442	16	GSE1 (-440730), KIAA0513 (+109274)
R.3910	86011988	86012573	16	FOXF1 (-531852), IRF8 (+79872)
R.3912	86231823	86232457	16	FOXF1 (-311993), IRF8 (+299731)
R.3915	86599111	86599745	16	FOXC2 (-1429)
R.3916	86609422	86610230	16	FOXL1 (-2289)
R.3917	86612312	86613094	16	FOXL1 (+588)
R.3921	86959156	86960025	16	FOXL1 (+347476), FBXO31 (+457772)
R.3940	88700622	88701212	16	IL17C (-4084)
R.3941	88717755	88717989	16	CYBA (-312)
R.3946	88849599	88850218	16	PIEZO1 (+1710), CTU2 (+77038)
R.3951	89034099	89034292	16	PABPN1L (-101170), CBFA2T3 (+9416)
R.3952	89043281	89043707	16	CBFA2T3 (+118)
R.3962	183931	184833	17	DOC2B (-152955), RPH3AL (+18216)
R.3997	4337082	4337231	17	SPNS3 (+174)
R.4002	4802813	4802906	17	CHRNE (+3509), MINK1 (+66177)
R.4003	4806344	4806972	17	CHRNE (-289)
R.4015	7036834	7037098	17	ASGR2 (-18838), ASGR1 (+45917)
R.4058	17740387	17740468	17	SREBF1 (-127)
R.4064	21281017	21281507	17	UBBP4 (-449433), KCNJ12 (+1753)
R.4066	25958267	25958673	17	LGALS9 (+1646), NOS2 (+169055)
R.4071	27038886	27039058	17	PROCA1 (-100)
R.4074	27369447	27370117	17	PIPOX (-136)
R.4077	28804347	28804679	17	GOSR1 (+133)
R.4084	33390736	33390854	17	RFFL (+25543), LIG3 (+83282)
R.4090	34344416	34345172	17	CCL23 (+211)
R.4092	36719518	36720382	17	SRCIN1 (+42233), ARHGAP23 (+135288)
R.4097	37823631	37824238	17	PNMT (-476)
R.4117	40489569	40489785	17	STAT5A (+50112), STAT3 (+50909)
R.4122	40935998	40936820	17	WNK4 (+3713), COA3 (+14313)
R.4130	41923945	41924123	17	CD300LG (-510)
R.4136	42988784	42989147	17	GFAP (+3896), CCDC103 (+11824)
R.4139	43037114	43037706	17	KIF18B (-12331), C1QL1 (+8029)
R.4148	43974869	43975063	17	STH (-101650), MAPT (+3218)
R.4152	46604235	46604554	17	SKAP1 (-96843), HOXB1 (+3964)
R.4158	46656543	46657274	17	HOXB4 (+564)
R.4159	46657555	46658170	17	HOXB4 (-390)
R.4160	46663648	46664647	17	HOXB4 (-6675), HOXB5 (+7175)
R.4162	46669485	46670029	17	HOXB4 (-12284), HOXB5 (+1566)
R.4163	46673625	46674514	17	HOXB5 (-2747)
R.4164	46676099	46676375	17	HOXB5 (-4914)
R.4170	47286719	47287577	17	ABI3 (-441), GNGT2 (-386)

R.4191	57918600	57918682	17	TUBD1 (+51655), VMP1 (+133815)
R.4193	59475396	59475917	17	TBX2 (-1600)
R.4194	59478953	59479306	17	TBX4 (-54677), TBX2 (+1873)
R.4195	59480470	59481280	17	TBX4 (-52932), TBX2 (+3618)
R.4206	62208049	62208434	17	ERN1 (-757)
R.4213	70723227	70724035	17	SLC39A11 (+365196), SOX9 (+606470)
R.4215	71361857	71361901	17	CDC42EP4 (-53565), SDK2 (+278349)
R.4217	72231318	72231448	17	DNAI2 (-46268), TTYH2 (+21730)
R.4227	73583839	73584070	17	MYO15B (-14643), LLGL2 (+62135)
R.4237	74868561	74869001	17	MGAT5B (+52)
R.4240	75282040	75282140	17	TNRC6C (-719047), SEPT9 (+4598)
R.4244	75446431	75446661	17	TNRC6C (-554591), SEPT9 (+169054)
R.4254	76183438	76183551	17	TK1 (-181), AFMID (+52)
R.4257	76588634	76588724	17	DNAH17 (-15203), CYTH1 (+189697)
R.4286	78865514	78865755	17	CHMP6 (-100006), RPTOR (+346567)
R.4288	78991965	78992323	17	BAIAP2 (-16818), CHMP6 (+26503)
R.4298	79259283	79259699	17	SLC38A10 (+9614), C17orf89 (+46452)
R.4301	79361621	79361742	17	TMEM105 (-57208), ENSG00000171282 (-11858)
R.4302	79370518	79371256	17	ENSG00000171282 (-2653)
R.4303	79371681	79372528	17	ENSG00000171282 (-1435)
R.4306	79479815	79479834	17	ACTG1 (-18)
R.4318	80196719	80197412	17	SLC16A3 (+5503), CSNK1D (+34541)
R.4322	80254862	80255457	17	CSNK1D (-23553), CD7 (+20318)
R.4340	81006485	81006524	17	ZNF750 (-208051), B3GNTL1 (+3181)
R.4360	29598296	29598433	18	RNF125 (+30)
R.4361	30353125	30353699	18	KLHL14 (-387)
R.4371	47794231	47794995	18	CCDC11 (-1721)
R.4397	1068487	1069320	19	HMHA1 (+2982), POLR2E (+26694)
R.4417	2282207	2282729	19	LINGO3 (+9555), OAZ1 (+12943)
R.4455	6481540	6482169	19	DENND1C (-36)
R.4490	13266390	13266738	19	IER2 (+3775), CACNA1A (+350474)
R.4491	13318873	13319777	19	IER2 (+56536), CACNA1A (+297713)
R.4511	16254474	16254516	19	HSH2D (-10)
R.4514	16999093	16999768	19	F2RL3 (-240)
R.4515	17346089	17346507	19	NR2F6 (+10451), USE1 (+20130)
R.4518	17487896	17488281	19	PLVAP (+70)
R.4525	17958647	17959082	19	JAK3 (-24)
R.4530	18721531	18721840	19	CRLF1 (-4026), TMEM59L (+3446)
R.4543	35786580	35786961	19	CD22 (-33319), MAG (+3743)
R.4564	41025339	41025959	19	SHKBP1 (-57108), SPTBN4 (+53501)
R.4566	41111297	41111757	19	LTBP4 (+8386), NUMBL (+85029)
R.4575	44285333	44285594	19	KCNN4 (-55)
R.4591	47137238	47137863	19	GNG8 (+391)
R.4592	47139338	47139761	19	GNG8 (-1608)
R.4593	47288039	47288263	19	STRN4 (-38013), SLC1A5 (+3700)
R.4594	47289410	47289611	19	STRN4 (-39373), SLC1A5 (+2340)
R.4597	48823408	48823427	19	CCDC114 (-86)
R.4599	48917816	48918205	19	GRWD1 (-31019), GRIN2D (+19879)
R.4615	51670251	51670666	19	CD33 (-57895), SIGLEC7 (+24901)
R.4650	58280801	58280927	19	ZNF586 (-174)

R.4663	2795194	2795601	20	TMEM239 (-1581), C20orf141 (-259)
R.4686	20433062	20433328	20	INSM1 (+84430), RALGAPA2 (+260071)
R.4701	35274595	35274655	20	SLA2 (-6)
R.4713	39311637	39312602	20	MAFB (+5760)
R.4715	39320926	39321729	20	MAFB (-3448)
R.4721	43883172	43883746	20	SLPI (-254)
R.4726	48598634	48599292	20	SNAI1 (-573)
R.4731	52199594	52199778	20	ZNF217 (+10692), TSHZ2 (+610740)
R.4745	58630315	58631001	20	CDH26 (+97176)
R.4752	61583686	61583830	20	SLC17A9 (-294)
R.4753	61866960	61867178	20	BIRC7 (-166)
R.4758	62184363	62185074	20	SRMS (-5862), HELZ2 (+19089)
R.4766	62738880	62739073	20	NPBWR2 (-453)
R.4773	36421467	36421955	21	RUNX1 (-70)
R.4780	42733729	42734266	21	MX2 (+128)
R.4782	43346184	43347094	21	PRDM15 (-47048), C2CD2 (+27360)
R.4784	44782434	44782872	21	SIK1 (+64355), CRYAA (+193535)
R.4810	18894280	18894389	22	DGCR6 (+599)
R.4813	19879176	19879696	22	COMT (-49694), GNB1L (-36974)
R.4819	22598937	22599332	22	VPREB1 (+48)
R.4848	36044226	36044540	22	APOL6 (-59)
R.4856	37403802	37404577	22	TST (+11318), CSF2RB (+94520)
R.4857	37545924	37546124	22	IL2RB (+6)
R.4883	44576654	44577265	22	PARVG (+8124), KIAA1644 (+131771)
R.4890	46276479	46276979	22	WNT7B (+96280), ATXN10 (+209050)
R.4909	50747040	50747878	22	PLXNB2 (-1403)
